# PI3K-driven HER2 expression is a potential therapeutic target in colorectal cancer stem cells

**DOI:** 10.1136/gutjnl-2020-323553

**Published:** 2021-01-12

**Authors:** Laura Rosa Mangiapane, Annalisa Nicotra, Alice Turdo, Miriam Gaggianesi, Paola Bianca, Simone Di Franco, Davide Stefano Sardina, Veronica Veschi, Michele Signore, Sven Beyes, Luca Fagnocchi, Micol Eleonora Fiori, Maria Rita Bongiorno, Melania Lo Iacono, Irene Pillitteri, Gloria Ganduscio, Gaspare Gulotta, Jan Paul Medema, Alessio Zippo, Matilde Todaro, Ruggero De Maria, Giorgio Stassi

**Affiliations:** 1 Department of Surgical, Oncological and Stomatological Sciences, Università degli Studi di Palermo, Palermo, Italy; 2 Department of Health Promotion Sciences, Internal Medicine and Medical Specialties, Università degli Studi di Palermo, Palermo, Italy; 3 Core Facilities, Istituto Superiore di Sanità, Roma, Italy; 4 Department of Cellular, Computational, and Integrative Biology (CIBIO), University of Trento, Trento, Italy; 5 Department of Oncology and Molecular Medicine, Istituto Superiore di Sanita, Roma, Italy; 6 Laboratory for Experimental Oncology and Radiobiology, Center for Experimental and Molecular Medicine, University of Amsterdam, Amsterdam, Noord-Holland, The Netherlands; 7 Oncode Institute, University of Amsterdam, Amsterdam, Noord-Holland, The Netherlands; 8 Institute of General Pathology, Universita Cattolica del Sacro Cuore Facolta di Medicina e Chirurgia, Roma, Italy; 9 Policlinico A Gemelli, Roma, Lazio, Italy

**Keywords:** colorectal cancer, stem cells, drug resistance, antibody targeted therapy

## Abstract

**Objective:**

Cancer stem cells are responsible for tumour spreading and relapse. Human epidermal growth factor receptor 2 (HER2) expression is a negative prognostic factor in colorectal cancer (CRC) and a potential target in tumours carrying the gene amplification. Our aim was to define the expression of HER2 in colorectal cancer stem cells (CR-CSCs) and its possible role as therapeutic target in CRC resistant to anti- epidermal growth factor receptor (EGFR) therapy.

**Design:**

A collection of primary sphere cell cultures obtained from 60 CRC specimens was used to generate CR-CSC mouse avatars to preclinically validate therapeutic options. We also made use of the ChIP-seq analysis for transcriptional evaluation of HER2 activation and global RNA-seq to identify the mechanisms underlying therapy resistance.

**Results:**

Here we show that in CD44v6-positive CR-CSCs, high HER2 expression levels are associated with an activation of the phosphatidylinositol 3-kinase (PI3K)/AKT pathway, which promotes the acetylation at the regulatory elements of the Erbb2 gene. HER2 targeting in combination with phosphatidylinositol 3-kinase (PI3K) and mitogen-activated protein kinase kinase (MEK) inhibitors induces CR-CSC death and regression of tumour xenografts, including those carrying *Kras* and *Pik3ca* mutation. Requirement for the triple targeting is due to the presence of cancer-associated fibroblasts, which release cytokines able to confer CR-CSC resistance to PI3K/AKT inhibitors. In contrast, targeting of PI3K/AKT as monotherapy is sufficient to kill liver-disseminating CR-CSCs in a model of adjuvant therapy.

**Conclusions:**

While PI3K targeting kills liver-colonising CR-CSCs, the concomitant inhibition of PI3K, HER2 and MEK is required to induce regression of tumours resistant to anti-EGFR therapies. These data may provide a rationale for designing clinical trials in the adjuvant and metastatic setting.

**Significance of this study**

**What is already known on this subject?**

- Advanced colorectal cancer (CRC) remains essentially incurable, particularly in the presence of genomic alterations in the signalling pathway of rat sarcoma (RAS).
- Human epidermal growth factor receptor 2 (HER2) expression seems to correlate with the stage of disease and reduced survival in CRC.
- Targeting *Erbb2* amplification has a significant therapeutic activity in patients with CRC.
- The phosphatidylinositol 3-kinase (PI3K)/AKT pathway is constitutively activated in colorectal cancer stem cells (CR-CSCs) and sustains the expression of CD44v6, which drives the metastatic dissemination.

**What are the new findings?**

- The constitutive activation of PI3K/AKT is associated with high expression levels of HER2 in CD44v6-positive CR-CSCs.
- HER2, in combination with PI3K and mitogen-activated protein kinase kinase (MEK) inhibitors, leads to cancer stem cell death and tumour regression in CRC avatars resistant to anti-epidermal growth factor receptor (EGFR) therapy and to combinations of PI3K, BRAF and HER2 targeting.
- Liver disseminated CR-CSCs can be effectively killed by PI3K/AKT inhibitors in an experimental model of adjuvant therapy.
- Cytokines released by cancer-associated fibroblasts, particularly hepatocyte growth factor (HGF), stromal cell-derived factor-1 (SDF-1) and osteopontin (OPN), confer resistance to the targeting of the PI3K/AKT pathway and surrogate the protective effect of tumour microenvironment.

**How might it impact on clinical practice in the foreseeable future?**

- PI3K/AKT inhibitors could be effective in the adjuvant setting for CRC.
- Targeting HER2, MEK and PI3K may provide a valuable therapeutic strategy against anti-EGFR-resistant advanced CRCs.

## INTRODUCTION

Despite major advances in terms of prevention and treatment, colorectal cancer (CRC) is one of the major causes of cancer-related death worldwide.^1^ Diagnosis at stage IV and tumour progression after surgery are very often lethal and require a substantial improvement of current therapeutic regimens. Over the past decade, the scientific community focused on different mechanisms found to be responsible for the development of therapy resistance, such as genetic heterogeneity and activation of alternative survival pathways.^2^ Despite the availability of a large repertoire of new targeted therapeutics, there are not many options to treat patients with chemoresistant tumours, particularly if associated with activation of the signal pathway of rat sarcoma (RAS) or epidermal growth factor receptor (EGFR) resistance.

The expansion of resistant subclones due to tumour heterogeneity is now regarded as the major clinical hurdle in patient management.^3^ About 36%–40% of patients with CRC are characterised by *Kras*-activating mutation at codons 12, 13 and 61, while 8%–15% present mutations in the *Braf* gene. In advanced stages, the presence of either *Braf* or *Ras* mutations correlates with a particularly poor prognosis.^4^ Several studies have shown the predictive and prognostic roles of different gene mutations belonging to mitogen-activated protein kinase (MAPK) and phosphatidylinositol 3-kinase (PI3K)/AKT pathways, such as *Ras*, *Braf*, *Pik3ca* and *PTEN*.^5 6^ EGFRs are the most important actionable targets identified so far in CRC. Although the addition of EGFR-targeting antibodies to chemotherapy is the most effective current therapy for *Ras* wild-type (wt) metastatic CRC, the therapeutic response is temporary and restricted to a limited number of patients due to primary or acquired resistance.^7^ Repeated liquid biopsies accompanied by the analysis of tumour-associated genetic alterations would be needed to monitor the treatment responses and to adapt new targeted therapies.^8^ The heterogeneity in the clinical responses of these patients with *Kras*-wt CRC has pointed out the contribution of other genetic mutations or amplifications.^9^ For instance, beyond the *Ras* mutation, the activation of alternative or parallel downstream signalling inside or outside the MAPK pathways is involved in the anti-EGFR treatment inefficacy.^7 10^ Thus, simultaneous inhibition of the EGFR family members and alternative signalling pathways has been adopted to overcome EGFR therapy resistance determined by the amplification of receptor tyrosine kinases.^11 12^ In contrast to melanoma, CRCs harbouring *Braf* mutations are refractory to BRAF inhibitors, such as vemurafenib.^13^ CRC resistance to BRAF inhibitors remains a major obstacle in clinical settings.^14^ This unresponsiveness is sustained by the activation of a feedback loop involving EGFR and downstream pathways.^15^ Although a recent phase II clinical study showed that addition of vemurafenib and cetuximab to chemotherapy prolonged the progression-free survival of metastatic patients with *Braf*-mutated tumours by 2.4 months, the prognosis of these patients remains poor.^16^ Moreover, another recent study reported that the combined BRAF, EGFR and mitogen-activated protein kinase kinase (MEK) inhibition in patients with *Braf^V600E^*-mutant CRC could increase survival, even if patients experienced primary and acquired resistance due to a positive feedback regulation of MAPK pathway in tumour cells.^17^

Given that *Kras*-mutant CRCs display different resistance mechanisms to EGFR and MAPK inhibitors, many alternative strategies are currently under investigation.^18^ In addition to acquired genetic mutations, the activation of a positive human epidermal growth factor receptor 2 (HER2) and HER3 feedback loop seems to be one example of antitumour therapy escape.^19 20^ Accordingly, the combined treatment with MEK and EGFR/HER2 dual inhibitors has shown a synergistic activity in preclinical models based on CRC cell lines bearing *Kras* mutation.^21^ The investigation on EGFR family members in CRC has been recently focused on HER2. *Erbb2* amplification occurs in approximately 3%–10% of patients with CRC and may promote resistance to EGFR inhibitors.^22 23^ Moreover, HER2 expression appears as a negative prognostic factor that correlates with the stage and survival in patients with CRC.^24^ This hypothesis has been recently validated by the clinical trial Heracles, which showed that the combination of trastuzumab and lapatinib in *Erbb2*-amplified patients with CRC can induce the regression of tumours resistant to anti-EGFR therapies.^25^ Constitutive expression of HER2 can be also driven by the degree of its enhancer and promoter activities.^26^

Myc overexpression may contribute to promote therapy resistance in *Kras*-mutant CRCs.^27^ While the MAPK effector promotes Myc stabilisation, its proteosomal degradation is mediated by GSK3β.^28 29^ The role of Myc in the tumourigenesis programme is mediated by the upregulation of the miR-17–92 cluster, which is associated with a poor prognosis.^30^

Colorectal cancer stem cells (CR-CSCs) are responsible for tumour development, spreading and resistance to chemotherapy.^31 32^ We have created a large collection of primary CRC cells growing as spheroids and able to reproduce the patients’ tumour in mouse avatars. We have recently shown that CR-CSCs express CD44v6 and depend on the PI3K/AKT pathway for survival and spreading.^31 33^ Herein, we have studied the molecular pathways that should be targeted to kill CR-CSCs both in the adjuvant and metastatic settings. While targeting the PI3K/AKT pathway is sufficient to kill disseminating CR-CSCs, we found that HER2 is constitutively expressed in CR-CSCs and that the simultaneous targeting of HER2, PI3K and MEK neutralises the protective effect of tumour stroma and induces tumour regression, even in the presence of aggressive mutational backgrounds.

## METHODS

A detailed description of the methods can be found in online supplemental information.

10.1136/gutjnl-2020-323553.supp1 Supplementary data

10.1136/gutjnl-2020-323553.supp9 Supplementary data

## RESULTS

### CD44V6-positive CRC cells express high levels of HER2 and are cetuximab resistant

**Figure 1:**
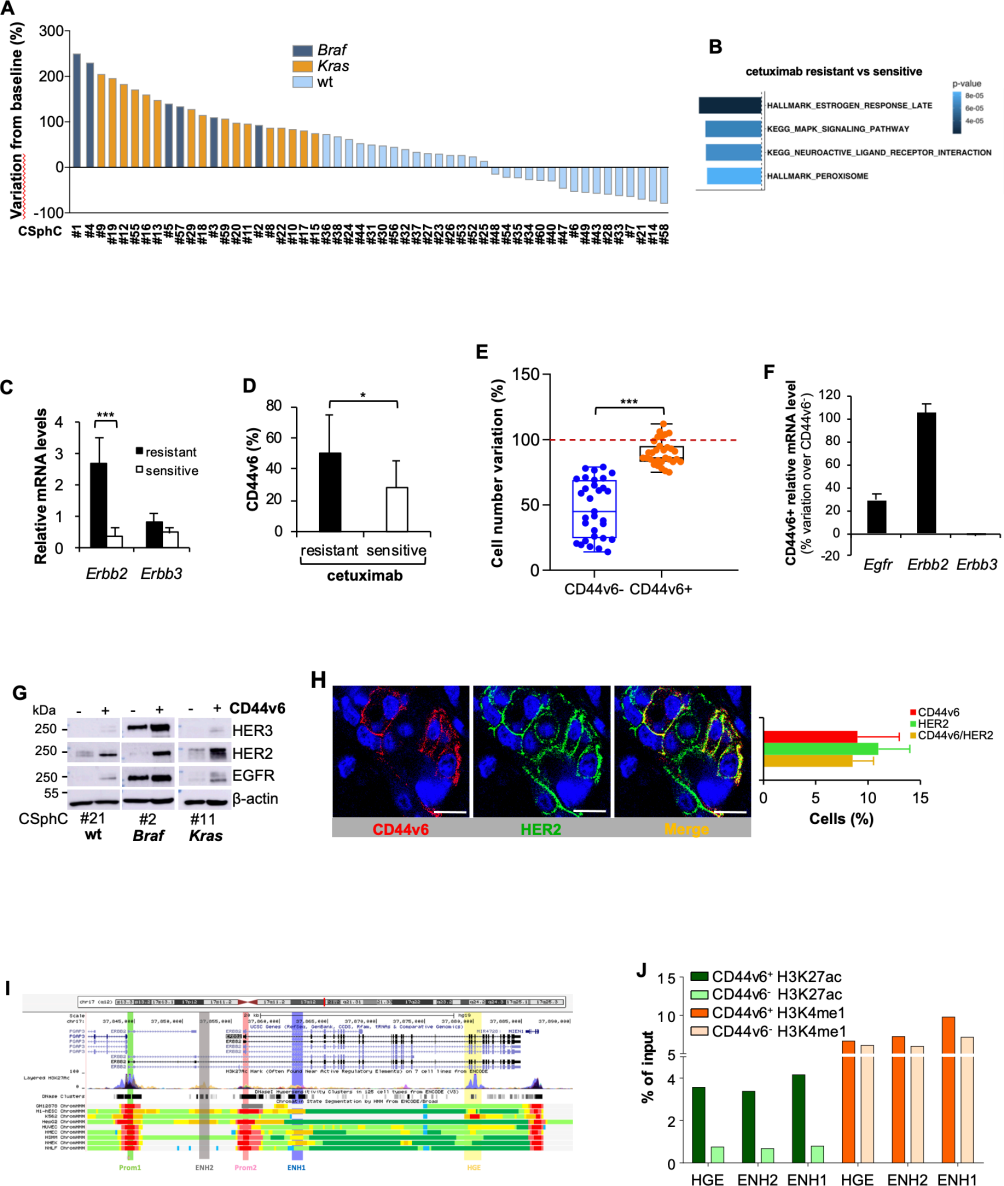
High expression of HER2 confers resistance to anti-epidermal growth factor receptor (EGFR) treatment in CD44v6-positive CR-CSCs.(A) Waterfall plot of cetuximab response in *Ras/Braf*-wt, *Braf*-mutant and *Kras*-mutant CSphC lines following 72 hours of treatment. (B) Top four significantly enriched gene sets in hallmark, canonical pathways MSigDB collections (false discovery rate (FDR) Q-value≤0.05) identified through the analysis of differentially expressed genes between cetuximab resistant versus sensitive *Ras/Braf*-wt sphere cells. P values related to each enriched gene set are indicated. (C) *Erbb2* and *Erbb3* mRNA expression levels in *Ras/Braf*-wt sphere cells resistant and sensitive to cetuximab. *Gapdh* amplification was used as endogenous control. Data are represented as ±SD of three experiments performed with 31 *Ras/Braf*-wt. (D) CD44v6 expression performed in cells as in (C). (E) Viable cell number variation in enriched CD44v6^−^ and CD44v6-positive *Ras/Braf*-wt treated with cetuximab for 72 hours and normalised with the values of cells treated with vehicle (indicated as 100%, red dotted line). Boxes and whiskers represent mean±SD of six experiments performed with 15 resistant and 16 sensitive *Ras/Braf*-wt sphere cells. (F) Variation of *Egfr*, *Erbb2* and *Erbb3* mRNA expression levels in CD44v6-positive versus CD44v6^−^ cells. *Gapdh* amplification was used as endogenous control. Data are represented as mean±SD of nine experiments performed with three *Ras/Braf*-wt (CSphC#14, 21 and 33), three *Braf*-mutants (CSphC#1, 2 and 5) and three *Kras*-mutants (CSphC#10, 11 and 16). (G) Immunoblot analysis of HER3, HER2 and EGFR on purified CD44v6^−^ and CD44v6-positive *Ras/Braf*-wt (CSphC#21), *Braf*-mutant (CSphC#2) and *Kras*-mutant (CSphC#11) CR-CSphC populations. β-Actin was used as loading control. (H, left panel) Representative immunofluorescence analysis of CD44v6 and HER2 on paraffin embedded sections from human CRC tissue specimen. Nuclei were counterstained with TOTO-3. Scale bars, 20 µm. Percentages of CD44v6, HER2 and CD44v6/HER2 positivity in eight human CRC tissues are shown on the right panel. Data are mean±SD of eight different samples. (I) Browser view of the *Erbb2* locus, showing different isoforms of *Erbb2* and chromatin states (ChromHMM tracks). Two promoters and three potential enhancers are highlighted (Prom1, Prom2, ENH1, ENH2 and HGE). (J) ChIP-qPCR for the histone marks H3K27ac and H3K4me1 at the indicated enhancer regions (ENH1, ENH2 and HGE) in *Braf*-mutant cells positive or negative for CD44v6. Enrichment is indicated as % relative to the input. CR-CSC, colorectal cancer stem cell; CSphC, colorectal cancer sphere cell; ENH1, intron 1 enhancer; ENH2, intron 2 enhancer; HER2, human epidermal growth factor receptor 2; HGE, *HER2* gene body enhancer; MSigDB, Molecular Signatures Database; wt, wild type. *indicates P<0.05 and ***indicates P<0.001.

EGFR inhibitors promote an effective therapeutic response in about 50%–55% of the patients with *Ras/Braf-*wt CRC, whereas *Braf*- and *Kras*-mutant CRC cells are completely resistant.^9^ In accordance with clinical data, treatment with cetuximab affected the cell viability of about a half of the *Ras/Braf-*wt primary colorectal cancer sphere cells (CSphCs) and delayed the outgrowth of tumour xenografts (figure 1A, online supplemental figure 1A, B and online supplemental table 1).

10.1136/gutjnl-2020-323553.supp2 Supplementary data

10.1136/gutjnl-2020-323553.supp3 Supplementary data

Primary resistance to the EGFR blockade is mostly due to a constitutive activation of the RAS-MAPK signalling network.^9^ Accordingly, a global RNA-Seq transcriptome analysis of cetuximab-resistant versus sensitive *Ras/Braf-*wt CSphCs showed 252 differentially expressed genes (DEGs) (online supplemental figure 1C and online supplemental table 2). The gene set enrichment analysis (GSEA) computed with the Molecular Signatures Database displayed the enrichment of genes associated with activation of the MAPK-signalling pathway, including the negative feedback regulator DUSP4^34^ (figure 1B and online supplemental figure 1D). Besides the activation of signalling pathway of MAPK, *Ras/Braf-*wt CSphCs resistant to cetuximab showed higher mRNA expression levels of *Erbb2* compared with those sensitive (figure 1C). In line with literature,^23^ our CSphC collection showed a 9.7% of *Erbb2* amplification (online supplemental table 1). We previously reported that while CD44v6 is a functional marker that identifies tumour-initiating CR-CSCs, the CD44v6-negative population represents the progenitor and differentiated fraction.^31 33^ A cohort of 31 out of 47 primary CSphC lines showed that the high percentage of CD44v6 expression levels resided in the *Ras/Braf-*wt cells resistant to cetuximab, even though these expression levels are similarly distributed between *Ras/Braf-*wt, *Braf*-mutated and *Kras-*mutated cell lines (figure 1D and online supplemental figure 1E). Because the CD44v6-positive population, within the *Ras/Braf-*wt cells, is resistant and increases after treatment with cetuximab (figure 1E and online supplemental figure 1F), we investigated whether the expression in signalling pathways associated with drug resistance may differ between the stem and differentiated cell compartments. Reverse phosphoproteomic analysis (RPPA) of CSphCs showed that, while MAPK pathways and HER2 are highly regulated, EGFR and HER3 are expressed in a lesser extent in the CD44v6-positive than in the CD44v6^−^ fraction (online supplemental figure 1G). The analysis at mRNA and protein levels confirmed an increased HER2 expression in the tumourigenic CD44v6-positive population of CRC cells, independently of the mutational background (figure 1F, G and online supplemental figure 1H, I). Accordingly, immunofluorescence analysis of patient tumour sections and tumour spheres indicated that the majority of CD44v6-positive cells coexpressed HER2 (figure 1H and online supplemental figure 1J).

10.1136/gutjnl-2020-323553.supp4 Supplementary data

We next investigated the transcriptional regulation of *Erbb2* expression in CD44v6-positive fraction by evaluating its 3′ regulatory elements. Interestingly, H3K27 acetylation (H3K27ac) was enriched at the analysed regions of *HER2* gene body enhancer (HGE), intron 1 enhancer (ENH1) and intron 2 enhancer in CD44v6-positive compartment, whereas H3K4me1 enrichment was equally found in both CD44v6^−^ and CD44v6-positive cell fractions at all three enhancer regions (figure 1I, J and online supplemental figure 1K). These data indicate that the enhancers are poised in both cellular fractions but only fully activated in the CD44v6-positive compartment, suggesting that the activation of *Erbb2* transcription mediated by the increased acetylation is restricted to the CD44v6-positive CRC cell compartment.

### PI3K/AKT pathway activation is associated with the transcriptional regulation of *Erbb2* in CD44v6-positive cells

**Figure 2:**
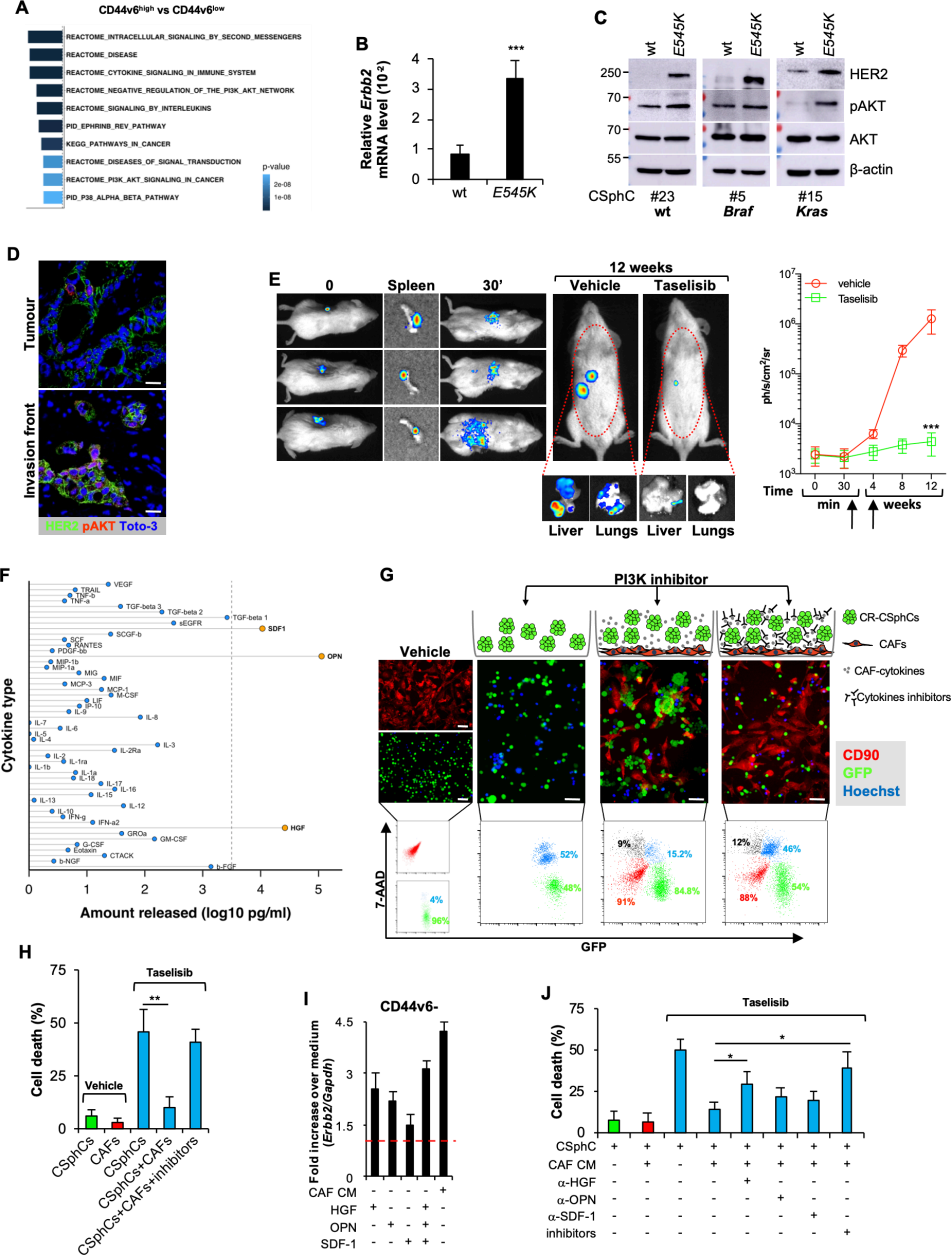
Activation of PI3K/AKT pathway is accompanied by elevated *Erbb2* expression levels in CD44v6-positive CRC cells. (A) Top 10 significantly enriched gene sets in hallmark, canonical pathways MSigDB collections (FDR Q-value≤0.05) computed by the analysis of differentially expressed genes between CD44v6^high^ and CD44v6^low^ cells. (B) mRNA relative levels of *Erbb2* in CSphCs and their corresponding CRISPR/Cas9-*Pik3ca* ^E545K^ cells. Data are represented as mean±SD of six independent experiments performed with *Ras/Braf*-wt (CSphC#23), *Braf*-mutant (CSphC#5) and *Kras-*mutant (CSphC#15) cells and their corresponding CRISPR/Cas9-*Pik3ca* ^E545K^ cells. (C) Immunoblot analysis of HER2, pAKT and AKT on *Ras/Braf*-wt (CSphC#23), *Braf*-mutant (CSphC#5) *Kras-*mutant (CSphC#15) cells. β-Actin was used as loading control. (D) Representative immunofluorescence analysis of HER2 and pAKT on paraffin-embedded sections from six human CRC tissue specimens. Nuclei were counterstained with TOTO-3. Scale bars, 20 µm. (E, left panels) In vivo whole-body imaging analysis of mice at 0 and 30 min and 12 weeks injected with sphere cells into the spleen. Five days after cell injection, mice were treated daily with taselisib for 3 weeks. Signal within the red dotted area represents the bioluminescence quantification. Kinetics of metastasis formation at the indicated time points (right panels). Black arrows indicate the start and end of treatment (from day 6 to week 4). Data are mean±SD of four independent experiments of six mice per group using *Kras*-mutant (CSphC#8 and 11) sphere cell lines. (F) Lollipop plot representing the amount of cytokines released by immortalised CAFs. Data are mean of six independent experiments using cells purified from six different patients. (G) Cell death (blue colour) evaluated by immunofluorescence (upper panels) and flow cytometry (lower panels) in sphere cells (CSphC#8) transduced with GFP (green colour) cocultured with CAFs CD90 positive (red colour) and treated with a PI3K inhibitor (taselisib) for 72 hours in the presence or absence of hepatocyte growth factor (HGF), stromal cell-derived factor-1 (SDF-1) and osteopontin (OPN) blocking antibodies (inhibitors). Scale bars, 40 µm. (H) Percentage of cell death in cells as in (G). Data are mean±SD of three independent experiments using *Ras/Braf*-wt (CSphC#14, 21 and 33), *Braf*-mutant (CSphC#1, 2 and 5) and *Kras*-mutant (CSphC#8, 10 and 11) sphere cell lines. (I) *Erbb2* mRNA expression levels in CD44v6^−^ enriched cells treated with CAF CM and the indicated cytokines. Data are mean±SD of three independent experiments performed with cells derived from *Ras/Braf*-wt (CSphC#14 and 33), *Braf*-mutant (CSphC#1 and 5) and *Kras-*mutant (CSphC#10 and 11) sphere cell lines. (J) Cell death in sphere cells exposed to CAF CM and treated with taselisib for 72 hours in the presence of cytokine neutralising antibodies as indicated. Data are mean±SD of three independent experiments performed with *Ras/Braf*-wt (CSphC#6, 14, 21 and 33), *Braf*-mutant (CSphC#1, 2, 4 and 5) and *Kras-*mutant (CSphC#8, 10, 11 and 17) sphere cell lines. CAF, cancer-associated fibroblast; CM, conditioned medium; CRC, colorectal cancer; CSphC, colorectal cancer sphere cell; HER2, human epidermal growth factor receptor 2; MSigDB, Molecular Signatures Database; wt, wild type. *indicates P<0.05, ** indicates P<0.01 and ***indicates P<0.001.

The transcriptomic analysis of CD44v6^high^ versus CD44v6^low^ cells highlighted the presence of 173 DEGs (online supplemental figure 2A and online supplemental table 3). In CD44v6^high^ cells, the GSEA enriched for genes related to the activation of PI3K/AKT signalling pathway, such as NOS3^35^ (figure 2A and online supplemental figure 2B). To investigate the role of PI3K in the transcriptional regulation of *Erbb2*, we induced an activating *Pik3ca* mutation into *Pik3ca-*wt low expressing HER2 CSphC lines by using CRISPR nuclease in combination with a specific donor DNA that introduced the *E545K* point mutation (online supplemental figure 2C and online supplemental table 1). The presence of a *Pik3ca^E545K^* is associated with an increased expression of HER2 and phospho-AKT (figure 2B, C and online supplemental figure 2D). Interestingly, immunofluorescence analysis of primary tumour sections indicated that HER2-positive cells were mainly prominent in the tumour invasion front and displayed activation of the PI3K/AKT pathway (figure 2D). The essential role of PI3K in the transcriptional regulation of *Erbb2* was further supported by ChIP-qPCR analysis showing that overexpression of the mostly represented *Pik3ca* activating mutation in breast cancer, the *Pik3ca^H1047R^*, in mammary IMEC-MYC cells enhances the transcriptional activity of both *Erbb2* promoters (prom 1 and prom 2) and ENH1 and HGE (online supplemental figure 2E). Since the inhibition of the PI3K/AKT pathway hampers the cell viability of CD44v6-positive cells,^33^ we evaluated whether the addition of a PI3K inhibitor to the combination therapy could affect the viability of both CD44v6-positive and CD44v6^−^ cells. To confirm the dependence of CR-CSCs on the PI3K activity, we tested an AKT (miransertib) and two PI3K (BKM120 and taselisib) inhibitors on several primary CSphC lines. Both miransertib and PI3K inhibitors reduced considerably the viability of CD44v6-positive cells in vitro regardless of the mutational background (online supplemental figure 2F–H), confirming that the PI3K/AKT pathway plays a key role on CR-CSC survival.

10.1136/gutjnl-2020-323553.supp5 Supplementary data

10.1136/gutjnl-2020-323553.supp6 Supplementary data

We previously showed that CRC development is sustained by cancer stem cells (CSCs), whose dissemination is responsible for CRC metastasisation,^33^ suggesting that targeting disseminated CR-CSCs may prevent tumour relapse and increase survival of patients with CRC.^31^ Thus, we investigated the ability of PI3K inhibitors to target disseminated sphere cells in the liver before they were able to make metastases in a model of adjuvant treatment. We found that the administration of taselisib in immunocompromised mice was able to prevent the formation of liver metastases after dissemination of CSphCs by spleen injection (figure 2E). These findings support the investigation of PI3K/AKT inhibitors in clinical trials aiming at killing disseminated metastasis-initiating CR-CSCs. The different survival properties of CD44v6 cells in vitro and in established tumours are likely due to the protective activity of the tumour microenvironment.^31^ Outside the protective tumour context, PI3K and AKT inhibitors can kill CR-CSCs. In contrast, the protective activity of cells and cytokines present in the tumour microenvironment may require the targeting of multiple pathways to overcome the enhanced survival of CR-CSCs. This hypothesis is supported by the significant therapeutic activity of PI3K inhibitors on micrometastases and small tumour lesions.^36^ In order to identify some possible soluble mediators of such protective activity, we then measured the release of cytokines from cancer-associated fibroblasts (CAFs). Among the cytokines more abundantly produced by CAFs, we selected HGF, SDF-1 and OPN (figure 2F) to further investigation, based on their ability to support PI3K/AKT activity and stemness properties in CSphCs.^33^ We next investigated whether the presence of CAFs would influence the survival of CSphCs exposed to the PI3K inhibitor. The coculture of GFP-labelled tumour spheres with CAFs protected cells from taselisib treatment (figure 2G), suggesting that CAFs could play a critical role in opposition to the killing activity of PI3K inhibitors in CRC. Moreover, neutralisation of HGF, SDF-1 and OPN completely prevented the protective activity of CAFs (figure 2H), indicating that these cytokines are responsible for delivering a survival signal in CR-CSCs that makes ineffective the PI3K targeting. Exposure of CSphCs, CD44v6-positive and CD44v6^−^ cell fractions to CAF-released cytokines enhanced the expression of *Erbb2* mRNA (figure 2I and online supplemental figure 2I). Interestingly, in the presence of tumour microenvironmental cytokines, *Erbb2* expression levels were not affected by the treatment with the PI3K inhibitor (online supplemental figure 2J–L). We also observed that HGF plays a major role in CAF-mediated protection of CSphCs treated with taselisib (figure 2J). Taken together, these data suggest that the tumour microenvironment protects CR-CSCs from the targeting of the PI3K/AKT pathway.

### MEK sustains CR-CSCs resistance to the triple targeting of HER2, BRAF and PI3K

**Figure 3:**
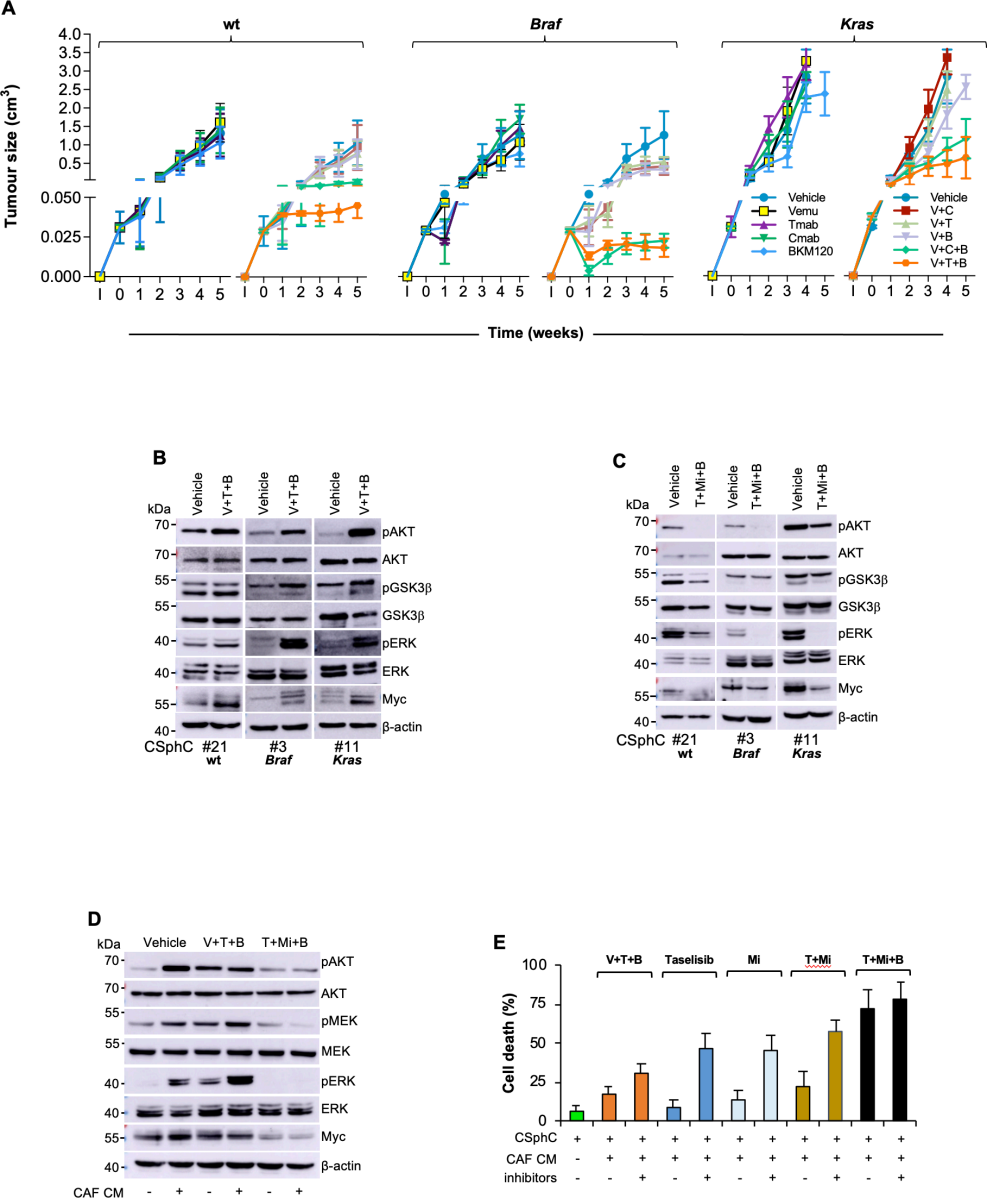
HER2/MEK/PI3K combinatorial targeting counteracts the protective effect of cytokines produced by CAF. (A) Size of xenograft tumours generated by subcutaneous injection of *Ras/Braf*-wt (CSphC#14, 21 and 33), *Braf-*mutant (CSphC#1, 2, 3 and 5) or *Kras-*mutant (CSphC#8, 11 and 16) sphere cells. Mice were treated for the first 4 weeks with vehicle (vehicle) or Vemu (or V), cetuximab (Cmab or C), Tmab (or T) and a PI3K inhibitor (B) alone or in combination as indicated. ‘I’ indicates the time of cell injection. Treatment was started at time 0. Data are mean values of six independent experiments (n=6 mice per group). (B) Immunoblot analysis of pAKT, AKT, pGSK3β, GSK3β, pERK, ERK and Myc on tumour xenograft-derived cells of mice injected with *RasBraf/Braf*-wt (CSphC#21), *Braf*-mutant (CSphC#3), *Kras*-mutant (CSphC#11) sphere cells. Mice were treated with vehicle or V in combination with T and B, and sacrificed 1 week after the treatment suspension (5 weeks). β-Actin was used as loading control. (C) Representative Western blot analysis of pAKT, AKT, pGSK3β, GSK3β, pERK, ERK and Myc in *Ras/Braf*-wt (CSphC#21), *Braf*-mutant (CSphC#3), *Kras*-mutant (CSphC#11) sphere cells treated for 24 hours with vehicle or T+Mi+B. β-Actin was used as loading control. (D) Immunoblot analysis of the indicated proteins in *Kras*-mutant (CSphC#9) sphere cells treated with vehicle or V in combination with T and B or T+Mi+B cultured in fetal bovine serum (FBS)-free Dulbecco’s modified eagle medium (DMEM) or CAF CM for 24 hours. (E) Cell death percentage in CSphCs exposed to hepatocyte growth factor (HGF), stromal cell-derived factor-1 (SDF-1) and osteopontin (OPN) blocking antibodies (inhibitors) and treated as indicated for 72 hours. Data are mean±SD of three independent experiments performed with *Ras/Braf*-wt (CSphC#14, 21 and 33), *Braf*-mutant (CSphC#1, 2 and 5) and *Kras-*mutant (CSphC#8, 10 and 11) sphere cell lines. B, BKM120; CAF, cancer-associated fibroblast; CSphC, colorectal cancer sphere cell; T, trastuzumab; T+Mi+B, trastuzumab in combination with MEKi and BKM120; V, vemurafenib; V+T+B, vemurafenib in combination with trastuzumab and BKM120; wt, wild type; CM, conditioned medium.

To further analyse the therapeutic potential of PI3K inhibitor in combination with MAPK pathway targeting, tumour xenografts, generated by the subcutaneous injection of CSphCs, were initially treated with either trastuzumab or cetuximab in combination with BRAF and PI3K inhibitors. These treatments were largely ineffective. We observed only a transient stabilisation of *Braf*-mutated tumours and a short delay in the disease progression of *Ras/Braf-*wt and *Kras*-mutated tumours (figure 3A and online supplemental figure 3A). These experiments allowed us to evaluate the potential mechanisms of acquired resistance to such triple combinations. CSphCs surviving the combinatorial treatment showed a significant phosphorylation of p235–236 S6 kinase (online supplemental figure 3B), which could follow the activation of RAS/ERK and mammalian target of rapamycin (mTOR) and result in the engagement of the Myc pathway.^37 38^ The activation of PI3K/AKT and MAPK pathways were confirmed by western blot in tumour specimen-derived subcutaneous xenograft treated with the triple combination (figure 3B and online supplemental figure 3C). This phenomenon was paralleled by a strong activation of the PI3K/AKT and MAPK pathways, particularly in the presence of Braf or *Kras* mutations (figure 3B). Moreover, cells surviving the combinatorial treatment showed high expression levels of the miR-17–92 cluster (online supplemental figure 3D), whose upregulation is associated with Myc expression.^39^ Altogether, these findings indicate that PI3K and MEK promote CR-CSC resistance to the targeting of BRAF, HER2 and PI3K signalling pathways.

10.1136/gutjnl-2020-323553.supp7 Supplementary data

10.1136/gutjnl-2020-323553.supp8 Supplementary data

Replacement of BRAF targeting with a MEK inhibitor caused a marked reduction of the PI3K/AKT and MAPK pathway activation and a decrease of phosphorylation of S6 kinase (figure 3C and online supplemental figure 3E, F). Viability of CSphCs was severely affected by the use of trametinib combination regardless of the mutational status and remarkably diminished the Myc-regulated miRNAs in cells previously exposed to vemurafenib-based combination (online supplemental figure 3G, H).

We then investigated whether the MEK inhibitor-based combination is also able to overcome the protective effect mediated by the tumour microenvironment. Beside PI3K, CAF-released cytokines boosted MAPK pathway activation, which persisted after the treatment of CSphCs with vemurafenib-based combination therapy (figure 3D). Conversely, pharmacological targeting of MEK, instead of BRAF, promoted a considerable cell death, paralleled with a marked reduction of MEK/ERK, AKT activation and Myc expression in CSphCs, independently of the presence of *Erbb2* amplification and the exposure to CAF conditioned medium (figure 3D, E and online supplemental figure 3I). Of note, sphere cells able to survive to a prolonged exposure to the vemurafenib-based treatment remain sensitive to the triplet containing trametinib (online supplemental figure 3J). Altogether, these data suggest that the tumour microenvironment confers therapy resistance mediated by Myc through the activation of MAPK and PI3K–AKT pathways.

### MEK inhibition-based therapy is synthetically lethal in CR-CSCs

**Figure 4:**
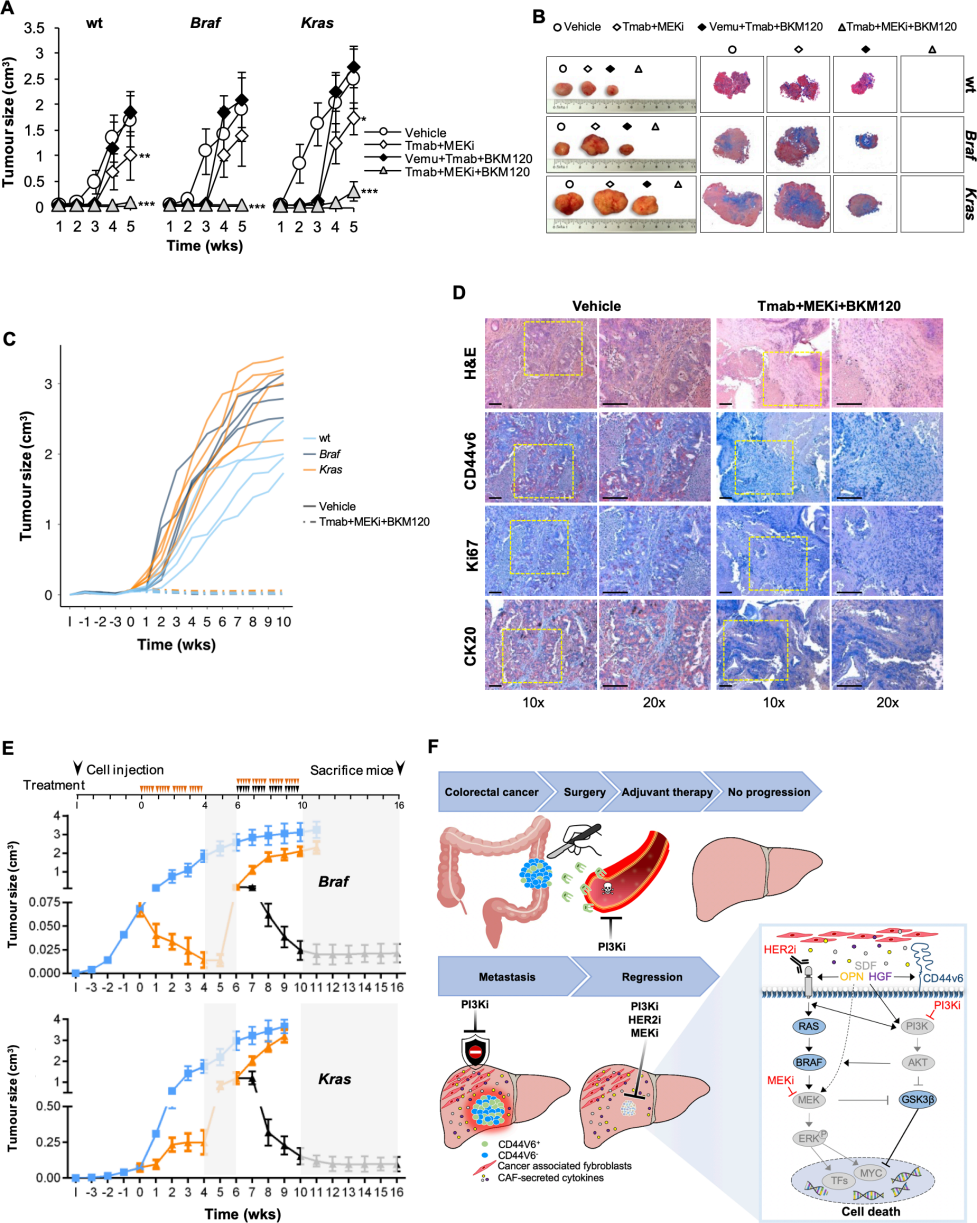
Therapeutic potential of HER2, PI3K and MEK targeting in CRC. (A) Size of tumours generated by subcutaneous injection of surviving *Ras/Braf*-wt (CSphC#14, 21 and 33), *Braf*-mutant (CSphC#1, 2 and 5) and *Kras*-mutant (CSphC#8, 11 and 16) sphere cells after 5 days of in vitro combination treatment as indicated. Data reported are mean±SD of tumour size for each cell lines (n=6 mice per group). (B) Representative macroscopic and Azan-Mallory analysis on tumour xenografts at 5 weeks treated as in (A). (C) Individual subcutaneous tumour volume plots of mice generated by the injection of four CSphC lines bearing the indicated different mutational background and treated for 4 weeks (0–4 weeks) with vehicle (vehicle) or Tmab plus MEKi plus BKM120. ‘I’ indicates the time of cell injection. Treatment was started at time 0. Data show kinetic growth of xenograft tumours generated by the injection of *Ras/Braf*-wt (CSphC#14, 21, 33 and 56), *Braf*-mutant (CSphC#1, 2, 3 and 5) and *Kras-*mutant (CSphC#8, 9, 11 and 16) CSphCs. (D) Representative H&E and immunohistochemical analysis of CD44v6, Ki67 and CK20 on tumour xenografts generated by the injection of *Kras*-mutant (CSphC#11) sphere cells treated as in (C) at the time of sacrifice (10 weeks). Scale bars, 200 µm. (E) Tumour size of mice xenografted with *Braf*-mutant (CSphC#1–5) and *Kras-*mutant (CSphC#8, 9, 11, 13 and 16) mutant sphere cells. Mice were treated with vehicle (vehicle, blue lines) or sequential treatments. A combination of Vemu, Tmab, BKM120 (Vemu+Tmab+BKM120, orange line) was used as first line (0–4 weeks, orange arrowheads) and after 2 weeks off-treatments, Tmab in combination with MEKi and BKM120 (Tmab+MEKi+BKM120, black lines and arrowheads) or the same Vemu combination used in the first 4 weeks (orange arrowheads) was administered from weeks 6 to 10. Off-treatments are highlighted with grey regions. ‘I’ indicates the time of cell injection. Data are expressed as mean±SD of subcutaneously implanted CSphC lines for each mutational status (n=6 mice per group). (F) Scheme of the signalling axis illustrating the site of action of the triple combination therapy. Surgery is the main treatment for primary CRC followed by adjuvant therapy. PI3Ki has shown efficacy in targeting disseminating CRC cells, impeding tumour progression (upper panel). However, PI3Ki as single agents are unable to counteract the TME protective influence in metastatic lesions. Triple combination treatment (PI3Ki, HER2i and MEKi) induces tumour regression by overcoming CAF-secreted cytokine effect (lower left panel). In CD44v6-positive CR-CSCs characterised by high PI3K pathway activity, TME-derived cytokines upregulate HER2 and CD44v6 expression levels, activate mitogen-activated protein kinase (MAPK) pathway and increase Myc protein levels, jeopardising the potential therapeutic efficacy of PI3Ki. The additional targeting of HER2 and the Myc upstream kinase MEK achieves a synthetic lethal effect in CR-CSCs (lower right panel). HER2, BRAF, PI3K and MEK inhibitors are indicated as I. CAF, cancer-associated fibroblast; CRC, colorectal cancer; CR-CSC, colorectal cancer cancer stem cell; CSphC, colorectal cancer sphere cell; HER2, human epidermal growth factor receptor 2; MEKi, trametinib; PI3Ki, PI3K inhibitors; TF, transcriptional factor; Tmab, trastuzumab; TME, tumour microenvironment; Vemu, vemurafenib; wt, wild type. *indicates P<0.05, ** indicates P<0.01 and ***indicates P<0.001.

In line with these results, we found that the replacement of vemurafenib with MEK inhibitors in the triple combination prevented the tumourigenic activity retained by sphere cells (figure 4A, B) and tumour progression when delivered in vivo, as indicated by the decrease in Ki67, CD44v6 and CK20 expression (figure 4C, D and online supplemental figure 4A–C). Of note, *Braf*-mutated or *Kras*-mutated xenograft tumours that recurred following the treatment with the vemurafenib-based triple combination, tumor xenografts resulted very sensitive to the trametinib-based combination therapy, as shown by the massive tumour regression and lack of regrowth even 6 weeks after treatment suspension (figure 4E). Next, we examined whether this MEK-targeted triplet was effective on a large number of primary CSphC cultures of different mutational backgrounds and their corresponding tumour xenografts. To confirm the effectiveness of this treatment, we tested other MEK and PI3K inhibitors (cobimetinib and taselisib) in combination with trastuzumab. Importantly, tumour size generated by the subcutaneous injection of primary sphere cells was significantly hampered by the treatment with either trametinib in combination with trastuzumab and BKM120 or cobimetinib plus trastuzumab and taselisib, independently of the mutational status (figure 4A and online supplemental figure 4D, E). Of note, this latter combination remarkably reduced the CD44v6 expression level on xenograft-derived CRC cells (online supplemental figure 4F). Consistently, cobimetinib plus trastuzumab and taselisib induced the death of a conspicuous number of cells that were substituted by fibrosis, resulting in a considerable decrease in the amount of Ki67-positive and CK20-positive cells (online supplemental figure 4G). Thus, simultaneous MEK/HER2/PI3K inhibition exerted a potent antitumour activity in CRC xenografts regardless of the mutational status. Altogether these data demonstrate that the combination treatment with HER2, PI3K and MEK inhibitors is synthetically lethal for CRC cells (figure 4F).

## DISCUSSION

The currently available targeted therapies for advanced CRC have a limited effect, particularly on the survival of patients carrying tumours with *Kras* mutation.^5^ We recently demonstrated that CD44v6-positive CR-CSCs are responsible for metastatic spreading and have a constitutive activation of the PI3K/AKT pathway that appears essential for their survival.^33^ Here, we demonstrate that CR-CSCs express high levels of HER2, which are associated with a constitutive activation of the PI3K/AKT pathway. Inhibition of HER2, MEK and PI3K kills CR-CSCs and promotes a long-lasting regression of all the tumour xenografts tested, regardless of their mutational background.

Among the attempts to target the actionable mutations in CRC, the treatment with anti-HER2 in patients carrying *Erbb2* amplification has been successful in clinical trials, whereas patients with *Braf^V600E^*-mutant CRC are poorly responsive to the administration of vemurafenib or dabrafenib.^13 40^

Although the existence of synthetic lethality between BRAF and EGFR in *Braf*-mutated CRC cells would predict the potential therapeutic effect of a combined targeting, we found that CR-CSCs are resistant to the combination of anti-EGFR or anti-HER2 and BRAF inhibitors due to the constitutive activation of the PI3K/AKT pathway. This could be the reason why vemurafenib, in combination with irinotecan and cetuximab, showed a weak therapeutic effect in patients with metastatic CRC.^41^

Here, we found that the regulatory elements of *Erbb2* transcription are acetylated in CD44v6-positive cells. While this can explain why CR-CSCs are remarked by high HER2 expression, the potential ability of PI3K to promote a transcriptional activation of *Erbb2* corroborates the hypothesis that both of these oncogenic pathways should be targeted simultaneously. Since HER2 expression is lost on CR-CSC differentiation, it is likely that the specific expression of HER2 in the CD44v6-positive cell compartment results from the considerable reduction of the PI3K/AKT signalling pathway and β-catenin activity observed in their differentiated progeny.^33^

Although sphere culture models mostly recapitulate the genetic landscape and the transcriptomic profile of parental tumour, representing valuable preliminary tools to identify potentially effective targeted therapies,^42 43^ it is fundamental to dissect the tumour microenvironment contribution in mediating resistance of cancer cells to therapeutic drugs.

According to our previous observation, we found that tumour microenvironmental cytokines produced by CAFs contribute to recapitulate a protective effect against antitumour drugs expanding the CD44v6-positive compartment expressing HER2. We showed that HGF and to a lesser extent OPN and SDF-1 make CR-CSCs resistant to the targeting of the PI3K/AKT pathway, possibly explaining the disappointing results obtained in the clinical trials that evaluated the therapeutic effects of PI3K inhibitors in metastatic patients.^44^ Such vulnerability of CR-CSCs in the absence of CAFs suggests that PI3K/AKT inhibitors can contribute to kill cells disseminated into the liver as part of adjuvant treatment due to the absence of a protective microenvironment. This hypothesis is strengthened by the observation that treatment with taselisib prevents the formation of liver metastases in mice receiving sphere cells by spleen injection.

In a subsequent set of experiments, we show that the addition of PI3K inhibitors to the combination of vemurafenib with trastuzumab or cetuximab induces a partial response of *Braf*-mutated tumours and a temporary stabilisation followed by a slower progression of *Ras/Braf-*wt and *Kras*-mutated tumours. Such transient therapeutic effect induces the rapid accumulation of tumour-initiating cells resistant to this triplet likely due to the presence of tumour microenvironmental cytokines.

The RPPA analysis in residual CSphCs spared by the HER2/BRAF/PI3K targeting allowed us to identify, through the regulation of S6 kinase phosphorylation, MEK and PI3K as major components of the resistance pathway. Accordingly, we observed increased levels of Myc in cells simultaneously exposed to agents targeting HER2, BRAF and PI3K. The concomitant activation of S6 kinase and MEK in sphere cells resistant to the vemurafenib-based triple combinations suggests that the failure to target both RAF and PI3K downstream pathways is responsible for maintaining activation of ERK and high Myc levels and promoting the pharmacological resistance of CR-CSCs to this triplet.

MEK is a key downstream element of the RAS-RAF pathway able to indirectly activate Myc.^15^ Replacement of vemurafenib with MEK inhibitors in the triple combination was able to significantly limit ERK activation and downregulate Myc expression while inducing a considerable therapeutic response in *Braf*-mutated and *Kras*-mutated tumours progressing after the vemurafenib-based combination. Of note, our data showed that MEK inhibition-based triplets were able to kill CR-CSCs in the presence of cytokines released by CAFs and to induce tumour regression in all CR-CSC-based xenografts tested, regardless of the mutational status and *Erbb2* amplification. Hence, HER2, PI3K and MEK appear as critical therapeutic targets in CR-CSCs, independently of the genomic abnormalities developed in patients’ tumours. This combination appears the most active both in tumour xenografts and in the in vitro experiments designed in the presence of the CAF-released cytokines.

The advent of targeted therapies and the study of the associated resistance mechanisms revealed the presence of clonal heterogeneity in CRC.^45^ Most of the current therapeutic strategies, including targeted combination treatments, affect differentiated cells and spare CSCs that eventually reinitiate tumour growth. It is therefore clear that the identification of the critical pathways responsible for the increase of survival and therapy resistance of CR-CSCs appears as a major priority to define possible effective treatments for patients with advanced CRC. This is particularly true for metastatic patients carrying oncogenic alterations in the RAS pathway, who have very limited therapeutic options. Our data show that MEK inhibition in association with PI3K and HER2 targeting can induce tumour regression even in tumours carrying mutations in the RAS pathway. Although targeted therapy is less toxic than standard chemotherapy, EGFR inhibitors are commonly associated with adverse events, including the inhibition of the MEK/ERK signal pathway, which compromises the epidermis cell differentiation leading to skin lesions.^46^ Given that HER2 inhibitors generally display minimal dermatological side effects as compared with those induced by EGFR inhibitors,^23^ as shown by current clinical studies for the treatment of advanced CRC,^25^ we foresee that triple targeting of HER2, MEK and PI3K may have a superior patient compliance and overall treatment outcome.

Here, we have shown that some biological features of CR-CSCs have the potential to be exploited in the clinics. The specific expression of HER2 in CR-CSCs, independently of gene amplification, suggests that HER2 should be regarded as key therapeutic target that deserves further preclinical and clinical investigations in CRC. The good therapeutic response, observed in clinical trial by HER2 targeting in patients with amplified tumours, increases the feasibility of this approach. Moreover, we provide evidence that targeting of the PI3K/AKT pathway could be exploited both in advanced disease and in the adjuvant setting. These findings may help define new therapeutic strategies based on CR-CSC targeting.

## Data Availability

Data are available in a public, open access repository. All data relevant to the study are included in the article or uploaded as supplementary information. The data that support the findings of this study are available from the corresponding author (GS) upon reasonable request. RNA sequencing data of CR-CSphCs have been deposited in a public, open access GEO repository, under accession number GSE162104 (link to data: https://www.ncbi.nlm.nih.gov/geo/query/acc.cgi?acc=GSE162104).
