# Supplementary material for: PI3K-driven HER2 expression is a potential therapeutic target in colorectal cancer stem cells
Source: Gut. 2021 Jan 12;71(1):119–28. doi: 10.1136/gutjnl-2020-323553 (PMC8666826; doi:10.1136/gutjnl-2020-323553)
Supplement: Supplementary data [file gutjnl-2020-323553supp001.pdf]

SUPPLEMENTAL INFORMATION

METHODS

CRC sphere cell culture

Purification and propagation of sphere cells was assessed as previously described[1] from 60 primary tumor specimens (age range 40–80 years) from CRC patients undergoing colon resection, in accordance with the ethical standards regarding Human Experimentation (authorization CE9/2015, Policlinico Paolo Giaccone, Palermo) (online supplemental table 1). CSphCs were cultured in serum-free stem cell medium (SCM) supplemented with EGF and b-FGF. Authentication of CSphC lines was routinely performed using a short tandem repeat DNA profiling kit (GlobalFiler™ PCR kit, Applied Biosystem) following the manufacturer's instructions and analyzed by ABI PRISM 3130 (Applied Biosystem). CSphC DNA profiles were matched with their relative patient tumor tissues. Mycoplasma presence was assessed with the MycoAlert™ Plus Mycoplasma Detection Kit (Lonza) every 3 months. To assess *Erbb2* copy number in CR-CSphCs we analyzed DNA samples using Digital Droplet PCR (Bio-Rad). After droplet generation and PCR amplification, plates were loaded in a Bio-Rad QX200 droplet reader and analyzed using the QuantaSoft v1.7.4 software (Bio-Rad) to evaluate droplets positive for *Erbb2* and/or *Rpp30*. Negative (no DNA) and positive (*Erbb2* amplified SKBR3 established cell line) controls were included in each plate. An *Erbb2*/*Rpp30* ratio < 1.25 identified non-amplified samples.[2, 3] The mutational profiles of the CSphCs were assessed by using a custom NGS panel and sequencing data were generated by MiSeq platform (Illumina).

CRC sphere cell treatment

CSphCs were treated with vemurafenib 1µM (S1267, Selleckchem), cetuximab 20 µg/ml and trastuzumab 10 µg/ml (kindly provided by the Department of Central Hospital Services- Policlinico Paolo Giaccone), taselisib 0.5 µM (GDC-0032, Chemietek), BKM120 1 µM (S2247, Selleckchem), trametinib 100 nM (S2673, Selleckchem), cobimetinib 0.5 µM (S8041, Selleckchem) or miransertib 2 µM (S8339, Selleckchem) . All the drugs were added in culture media every 48 hours.

The primary human CAF cell lines were isolated from CRC tissues obtained in accordance with the ethical policy of the University of Palermo Committee on Human Experimentation. For co-culture experiments, $1x10^5$ immortalized CAFs (CAFs) were plated as monolayer into 12-well plates for 48 hours, until confluence was reached and subsequently $6x10^5$ GFP-transduced CSphCs were seeded in presence or absence of HGF (1 µg/ml, MAB294, R&D), OPN (2 µg/ml, AF1433, R&D) and SDF-1 (50 µM, A5602, Sigma-Aldrich) neutralizing antibodies. After 48 hours cells were treated with 0.5 µM of taselisib for additional 72 hours. The preparation of CAF conditioned medium (CAF CM) was performed by culturing 1×10^6 cells in 5 ml of SCM for 48 hours. To analyze *Erbb2*

expression levels, CSphCs were exposed to CAF CM, HGF (100 ng/ml, PeproTech), OPN (1 μg/ml, Sigma-Aldrich) and SDF-1 (100 ng/ml, PeproTech) for 48 hours.

Cell survival

Cell proliferation and viability were evaluated by using CellTiter-Glo®Luminescent Cell Viability Assay Kit (Promega) according to the manufacturer's instructions and analyzed by Infinite® F500 (Tecan). Tripan blue exclusion test was accomplished to validate cell viability. CSphC death was assessed by adding Hoechst 33342 (Thermo Fisher Scientific) or 7-AAD (BD Pharmingen) and analyzed by a fluorescence microscope or flow cytometer, respectively.

Reverse-Phase Protein microArrays

The RPPA assay was carried out by the Functional Proteomics RPPA Core Facility by MD Anderson (Houston, TX, USA)[4] on a service basis (online supplemental table 4). The data analysis was performed by our group. Briefly, cell pellets were lysed for protein extraction by resuspension in a buffer containing T-PER reagent (Thermo Fisher Scientific), 300 mM NaCl (J.T.Baker; Avantor Performance Materials, Center Valley, PA), protease and phosphatase inhibitor cocktails (Sigma-Aldrich), followed by 20 min incubation on ice. Following protein extraction, samples underwent centrifugation at 10,000xg for 10 minutes and, subsequently, supernatants were transferred to fresh tubes for quantification of total protein concentration (Bradford reagent method, Thermo Fisher Scientific). Before shipping for RPPA assay service, lysates were diluted to a final concentration of 0.5 μg/μl using 2X sodium dodecyl sulfate (SDS) buffer (Thermo Fisher Scientific) and 2-mercaptoethanol was added to a final concentration of 2.5%. Analysis of pre-normalized RPPA data was performed by means of the "R3.6" software and the following packages: 'base', 'coin', 'exactRankTests', 'gridExtra', 'grid', 'tidyverse', 'plyr', 'dendextend' and 'ComplexHeatmap'.

Western blot

Cell were lysed in presence of ice-cold buffer containing Tris-HCL 10 mM (Sigma-Aldrich), NaCl 50 mM (Sigma-Aldrich), sodium pyruvate 30 mM (Sigma-Aldrich), NaF 50 nM (Sigma-Aldrich), $ZnCl_2$ 5 μM (Sigma-Aldrich), triton 1% (Bio-Rad), protease inhibitor cocktail (Sigma-Aldrich), phosphatase inhibitor cocktail 2 and 3 (Sigma-Aldrich), sodium orthovanadate 0.1 nM (Sigma-Aldrich), sodium butyrate 10 mM (Sigma-Aldrich) and PMSF 1 mM (Sigma-Aldrich). Proteins extracted were loaded in SDS-PAGE gels and blotted on nitrocellulose membranes. After incubation with blocking solution (0.1% Tween 20 and 5% non-fat dry milk in PBS) for 1 hour at room

temperature, membranes were exposed overnight at 4°C to HER3/ErbB3 XP (D22C5, rabbit IgG, CST), HER2/ErbB2 XP (D8F12, rabbit IgG, CST), EGF receptor XP (D38B1, rabbit IgG, CST), phospho-AKT XP (Ser473; D9E, rabbit, IgG, CST), AKT (rabbit polyclonal, CST), phospho-GSK3α/β (Ser21/9; D17D2, rabbit IgG, CST), GSK-3β (27C10, rabbit IgG, CST), phospho-ERK 1/2 (Thr202/Tyr204; rabbit polyclonal, CST), ERK 1/2 (137F5, rabbit IgG, CST), Myc (rabbit polyclonal, CST), phospho-MEK1/2 (Ser217/221; 41G9, rabbit IgG, CST) or MEK1/2 (rabbit polyclonal, CST). β-actin (8H10D10, mouse, CST) was used as loading control to normalize protein expression. Primary antibodies were revealed using anti-mouse or anti-rabbit HRP-conjugated (goat H+L, Thermo Fisher Scientific) for 1 hour at room temperature and detected with SuperSignal™ West Dura Extended Duration Substrate (Thermo Fisher Scientific) using Amersham imager 600 (GE Healthcare). Protein levels were calculated by densitometric analysis using ImageJ software.

Cytokines quantification

CAF-released cytokines involved in tumor inflammation, cell proliferation and immune response processes were quantified using the Bio-Plex Pro™ Human Cytokine 21-plex and 27-plex Assay (Bio-Rad). HGF, OPN, SDF-1 and TGF-β production was assessed by using the Human Cancer Biomarker Panel 1 16-plex #171AC500M, Bio-Plex Pro Human Cytokine SDF-1α Set #171B6019M, and Bio-Plex Pro™ TGF-β 3-plex Assay #171W4001M, respectively. Raw data were analyzed by Bio-Plex Software (Bio-Rad).

Flow cytometry and cell sorting

Cells were harvested, washed in PBS twice and stained for 1 hour at 4°C with conjugated antibodies CD44v6-APC (2F10, mouse IgG1, R&D systems) or isotype matched control (IC002A, mouse IgG1, R&D systems) and analyzed using both Accuri C6 Plus and FACSLyric (BD) flow cytometers. CD90 expression was assessed by using CD90 purified antibody (5E10, mouse IgG1k, BD) or isotype matched control (MOPC-21, mouse IgG1k, BD) and subsequent labeling with secondary antibodies Alexafluor 647 (goat anti-mouse IgG1, Thermo Fisher Scientific). Enrichment of CD44v6 or OFP expressing cells was accomplished by FACSMelody cell sorter. Prior to sorting, cells were washed with PBS containing 2% BSA and 2 mM EDTA and filtered with a 70 µM mesh to prevent clogging. Post-sorting acquisition was performed in order to verify the population purity. Dead cells were excluded on the basis of the uptake of 7-AAD (0.25 µg/1x106 cells, BD Biosciences).

RNA Extraction, Real-Time PCR and RNASeq

Total RNA of CSphCs was isolated by using Trizol Reagent (Thermo Fisher) and retro-transcribed with the high-capacity c-DNA reverse Transcription kit (Applied Biosystem). Real-time PCR was performed by using the rotor gene probe PCR master mix (Qiagen) and the following primers: *Erbb2* (Hs01001580_m1), *Erbb3* (Hs00176538_m1), *Egfr* (Hs01076078_m1) and *Gapdh* (Hs02786624_g1) (Applied Biosystem). The relative mRNA level was normalized to *Gapdh* and calculated by using the comparative Ct method (ΔΔCt).

To evaluate the miRNA expression profiles of CSphCs untreated and exposed to different treatments, total RNA samples were retro-transcribed and real time PCR were performed using Megaplex pools kit (Applied Biosystem) specific for a set of 384 microRNAs (TaqMan Human MicroRNA Array A) as recommended by manufacturer's instructions. Collected data were analyzed with the Thermo FisherCloud software.

NEBNext Ultra Directional RNA Library Prep Kit for Illumina was used for the preparation of RNASeq samples following the manufacturer's instructions. mRNA was purified using oligo-dT magnetic beads from total RNA. Retrotranscribed cDNA was used for ligation with adapters and PCR amplification. Clustering and DNA sequencing was assessed using the Illumina NextSeq 500. Analysis was performed by using the Illumina data analysis pipeline RTA v2.4.11 and Bcl2fastq v2.17. Raw sequencing reads were aligned to Ensembl release 84 (GRCh38 assembly) using the HISAT2 2.1.0 pipeline. Data were analyzed using the R version 3.5.0 and plotted by the pheatmap version 1.0.10, gtools 3.8.1 and ggplot2 3.0.0. Gene set enrichment analysis (GSEA) was assessed by selecting Hallmarks and canonical pathways within molecular signatures database (MSigDB) version 7.0. False Discovery Rate q value ≤ 0.05 was used to identify significantly enriched gene sets.

Chromatin immunoprecipitation (ChIP) and ChIP-qPCR

Chromatin was isolated from both CD44v6^{+} and CD44v6^{-} CSphCs, IMEC, IMEC-MYC and IMEC-MYC-PI3K cells. Cells were fixed adding formaldehyde directly to the cell culture media to reach a final concentration of 1%, then were incubated for 10 min at room temperature (RT). The reaction was quenched adding glycine to a final concentration of 125 mM and incubated 5 min at RT. The medium was removed and cells were washed 3 times with cold, sterile PBS plus protease inhibitor, then cells were collected by centrifugation at 4°C for 5 min at 1200 rpm. ChIP experiments were performed as previously described. [5, 6] Briefly, cell pellets were lysed in lysis buffer (50 mM Tris-HCl pH 8, 0.1% SDS, 10 mM EDTA pH 8, 1 mM PMSF (Sigma-Aldrich), protease inhibitor cocktail (Roche)) and chromatin was sonicated with a Bioruptor Pico sonicator

(Diagenode) for 25 cycles of 30 s, to reach an average fragment size of ~ 300 kb. Following quantification, 10 µg of sonicated chromatin were used in each immune-precipitation and incubated overnight at 4°C with 4 µg of indicated antibodies: anti- monomethyl histone H3 Lys4 (rabbit polyclonal, Abcam); anti-acethyl histone H3 Lys27 (rabbit polyclonal, Abcam). Concomitantly, protein G-coupled Dynabeads (Thermo Fisher Scientific) were blocked overnight at 4°C with 1 mg/ml sonicated salmon sperm DNA (Thermo Fisher Scientific) and 1 mg/ml BSA and then incubated with the ChIP reactions for 4 hours at 4°C. Magnetic beads were sequentially recovered and resuspended in RIPA buffer (10 mM Tris-HCl, pH 8, 0.1% SDS, 1 mM EDTA, pH 8, 140 mM NaCl, 1% DOC, 1% Triton, 1 mM PMSF, protease inhibitor cocktail), washed 5 times with ice-cold RIPA buffer, twice with ice-cold RIPA-500 buffer (10 mM Tris-HCl, pH 8, 0.1% SDS, 1 mM EDTA, pH 8, 500 mM NaCl, 1% DOC, 1% Triton, 1 mM PMSF, protease inhibitor cocktail), twice with ice-cold LiCl buffer (10 mM Tris-HCl, pH 8, 0.1% SDS, 1 mM EDTA, pH 8, 250 mM LiCl, 0.5% DOC, 0.5% NP-40, 1 mM PMSF, protease inhibitor cocktail) and once with TE buffer (10 mM Tris-HCl, pH 8, 1 mM EDTA, pH 8, 1 mM PMSF, protease inhibitor cocktail). Finally, the crosslinking was reversed in direct elution buffer (10 mM Tris-HCl, pH 8, 0.5% SDS, 5 mM EDTA, pH 8, 300 mM NaCl) at 65°C overnight and DNA was purified using Agencourt AMPure XP SPRI beads (Beckman, #A63882) and dissolved in 60 ml of Tris-HCl, pH 8. DNA was analyzed by quantitative PCR using the 2x SensiFAST SYBR No-ROX Mix (Bioline). The following oligos were used: Prom1 (fwd: CACCATCATGTGTCGCCAAG / rev: GCAGGTTGGAAGAGGCAAAA), ENH2 (fwd: CAGTTTGTGGCCTGGACATC / rev: TACCTACTTCACCAGCCAGC), Prom2 (fwd: GGCTTGGGATGGAGTAGGAT / rev: AAATTCCCTAGGCTGCCACT), ENH1 (fwd: GACCACCAGAGTCCAGAGAG / rev: TCTCCGAACAAAAGGGACCA), HGE (fwd: GATCCGGAAGTACACGATGC / rev: GGCTGGGAGGACTTCACC), control (fwd: GATCAAGTCAGGCTGAATACACG / rev: TCTGTGCTCCTAGCTTGTCCC). All experimental values were shown as percentage of input. To take into account background signals, we subtracted the values obtained with a non-immune serum to the relative ChIP signals.

CRISPR editing and lentiviral transduction

CSphCs were transfected with 1 µg of all-in-one OFP vector system for CRISPR-based genome editing (Thermo Fisher Scientific) and 1 µg of pMA-T plasmid containing customized donor DNA for $Pik3ca^{E545K}$ (Thermo Fisher Scientific), using the X-tremeGENE HP DNA Transfection Reagent (Roche) according to the manufacturer's instructions. DNA from control and transfected CSphCs was purified using the DNeasy Blood & Tissue Kit (Qiagen). The presence of $Pik3ca^{E545K}$ mutation

was assessed using the HotStarTaq Plus Master Mix Kit (Qiagen) using the following primer set: F-ATTGTTCACTACCATCCTC; R-TAATGTGCCAACTACCAATG. Amplified products were then purified using the MinElute PCR Purification Kit (Qiagen). Purification and base pair sequence were prepared using the BigDye Terminator v3.1 Cycle Sequencing Kit and BigDye X-Terminator Purification Kit (Applied Biosystems), respectively. Capillary electrophoresis was performed on ABI PRISM 3130 Genetic Analyzer.

Lentiviral particles were generated by transfecting HEK-293T packaging cells with p-TWEEN LUC-GFP plasmids together with psPAX2 (Addgene, 12260) and pMD2.G (Addgene, 12259) in DMEM 10% FBS supplemented with XtremeGENE HP DNA Transfection Reagent (Roche). For stable cell transduction, concentrated lentiviral particles were added to $1x10^5$ cells in culture medium in the presence of 8 μg/mL of polybrene (Sigma-Aldrich). hTERT-immortalized human mammary epithelial cells (IMEC) were transduced with pMXs-c-Myc, PGK-Pik3ca H1047R, pBABE-puro-Ras V12, and MSCV-p53DD-iGFP vector, respectively as previously described.[6] CAFs immortalization has been performed using a pLV-hTERT-IRES-hygro lentiviral plasmid (Addgene). Hygromycin (10 µg/ml; ant-hg-5, Invivogen) was used for selection of CAFs.

Animals and tumor models

6-8 weeks old male NOD/SCID mice were purchased by Charles River Laboratories and housed in the animal house at the Department of Biomedicine, Neuroscience and Advanced Diagnostics (Bi.N.D., University of Palermo). Mice were kept in a barrier facility for animals in a temperature-controlled system with a 12 hours dark/light cycle. Mice were given *ad libitum* access to pelleted chow [Special Diets Services-811900 VRF1 (P), Essex, UK] and to 0.45 mm filtered tap water in sterile drinking bottles. Each cage (1284L, Tecniplast S.p.A., Italy) provided with radiation-sterilized bedding (SAWI Research Bedding, JELU-WERK, Germany) has been used to house a maximum of 6 mice. Enrichment material such as soft paper and small red plastic houses (The Mouse House, ACRE011, Tecniplast) was used to meet animal welfare. All the surgical procedures have been performed during daytime, in accordance with the animal care committee guidelines of the University of Palermo (Italian Ministry of Health authorization n. 154/2017-PR). Subcutaneous xenografts were generated by injecting $2.5x10^5$ CSphCs in the flank of NOD/SCID mice, in 150 µl of 1:1 SCM/Matrigel (BD) solution. After tumor appearance (0.03-0.06 cm^3), mice were randomized in control and treatment groups (6 mice/group). Mice were treated for 5 days/week, for 4 weeks, with vehicle, taselisib (5 mg/Kg, once-daily, oral gavage), vemurafenib (20 mg/Kg, twice-daily, oral gavage), trastuzumab (5 mg/Kg, once-weekly, *i.p.*), cetuximab (40 mg/Kg, twice-weekly, *i.p.*), BKM120 (20 mg/Kg, once-daily, oral gavage), trametinib (0.3 mg/Kg, once-daily, oral

gavage) or cobimetinib (5 mg/Kg, once-daily, oral gavage). Tumors were measured twice per week by a digital caliper. Tumor volume was calculated using the formula: largest diameter x (smallest diameter)^2 x π/6. Once the endpoints were reached (2 cm in tumor diameter or when mice showed signs of suffering), animals were sacrificed accordingly to Directive 2010/63/EU guidelines (D.lgs 26/2016) and organs were collected for subsequent analyses. For the adjuvant therapy experiments, 3×10^5 LUC-transduced *Kras*-mutant CSphCs resuspended in PBS were injected into the spleen of NOD/SCID mice. The migration of sphere cells was assessed at the time of cell injection and at 30 min immediately after splenectomy up to 12 weeks (every 4 weeks) following *i.p.* injection of VIVO Glo ™ Luciferin (150 mg/kg, Promega) by using *in vivo* imaging system (IVIS Spectrum, PerkinElmer). After 5 days of sphere cells injection, mice were treated for 3 weeks with vehicle or taselisib.

Immunofluorescence/Immunohistochemistry

Cytospun of CD44v6$^+$ and CD44v6$^-$ cell fractions were fixed and permeabilized as previously described.[1] Cells were exposed overnight at 4°C to specific antibodies to detect CD44v6 (2F10, mouse IgG_1, R&D systems) and HER2 (D8F12, rabbit IgG, CST). Subsequently, cells were labeled with secondary antibodies, Rhodamine Red-x Goat anti-Mouse IgG1 and Alexa Fluor-488 Goat anti-rabbit IgG (Life Technologies). Nuclei were counterstained using TOTO-3 iodide (Life Technologies). Cells were examined under a confocal microscope. For CAF-CSphC co-culture experiment, cells were labeled with CD90 (SE10, mouse IgG1, BD Pharmingen) and subsequently with Alexa Fluor-647 Goat anti-mouse IgG1 (Life Technologies). Nuclei were counterstained using Hoechst (33258, Thermofisher). Cell staining were examined by using EVOS Cell Imaging System (Life Technologies).

Immunohistochemical and immunofluorescence analysis were performed on 5-µm-thick paraffin-embedded xenograft sections using antibody specific for CD44v6 (2F10, mouse IgG_1, R&D systems), HER2 (D8F12, rabbit IgG, CST), phospho-AKT XP (Ser473; D9E, rabbit, IgG, CST), Ki67 (T595, $IgG1_k$, Novocastra) and CK20 (Ks20.8, $IgG2a_k$, Novocastra). Stainings were then revealed using byotine-streptavidine system (Dako) and detected with 3-amino-9-ethylcarbanzole (AEC, Dako). Nuclei were counterstained with aqueous hematoxylin (Sigma-Aldrich). Azan-Mallory and H&E stainings were performed using standard protocols.

Statistical analysis

Results are shown as mean ± SD for at least three repeated independent experiments for each group. The mean and SD were obtained by analyzing replicates using Prism 5 (GraphPad Software, La Jolla, CA, USA) applying ANOVA test (one-way or two-way) with Bonferroni multiple comparisons test. P-values less than 0.05 were considered statistically significant. *indicates P<0.05, ** indicates P<0.01 and ***indicates P<0.001. RNA-seq data were normalized with log2-counts per million transformation. Differential expression analyses between diverse conditions were conducted in R (v3.6.1) by using limma package. P-values for log2 fold-change values are corrected with Benjamini-Hochberg procedure, only statistically significant differences were reported. ggplot2 and pheatmap packages were used for plotting the distributions and line plots, and heatmaps with clustergrams and sample annotations, respectively.

SUPPLEMENTAL FIGURE LEGENDS

Supplementary Figure 1. CD44v6 positive cells express high levels of HER2 and MAPK-ERK signaling molecules. (**A**) Dose-response of cetuximab (Cmab) on *Ras/Braf*-wt (CSphC#14, 21) and *Braf*-mutant (CSphC#2, 5) sphere cell lines at the indicated time points. Data are mean ± S.D. of 3 independent experiments. (**B**) Waterfall plot of cetuximab response in *Ras/Braf*-wt xenograft tumors from sensitive CSphCs treated for 4 weeks and analyzed 1 week after treatment suspension. *Braf*- and *Kras*-mutant xenografts served as control. (**C**) Unsupervised clustering of RNA-seq data from sphere cells sensitive and resistant to cetuximab and harboring the indicated mutations. (**D**) Log fold change (logFC) values of a subset of statistically differentially expressed genes, involved in the MAPK pathway, in sensitive versus resistant *Ras/Braf*-wt sphere cells to cetuximab, obtained by limma analysis. (**E**) Statistical distribution of CD44v6 positivity in 47 CSphC lines characterized by the indicated mutational background. Boxes and whiskers represent median ± S.D. of 6 experiments. Dotted lines indicate high (≥70%), medium (69%-31%) and low (≤30%) CD44v6 levels. (**F**) *(Left panel)* Representative immunohistochemical analysis for CD44v6 on tumor xenografts generated by the injection of *Ras/Braf*-wt (CSphC#14) sphere cells and treated with cetuximab for 4 weeks. Scale bars, 200 µm. *(Right panel)* Percentage of CD44v6 positivity in tumor xenografts generated by the injection of *Ras/Braf*-wt (CSphC#14, 21, 33) sphere cells. Data are mean ± S.D. of 3 independent experiments. (**G**) RPPA analysis of CRC spheres (Bulk) and enriched CD44v6$^+$ and CD44v6$^-$ *Braf*- (CSphC#2) mutant cells. (**H**) Immunoblot analysis of HER3, HER2 and EGFR and their relative bar graphs on CD44v6$^-$ and CD44v6$^+$ cells derived from 10 different CR-CSphC lines with different mutational backgrounds (CSphC#1, #2, #3, #9, #11, #15, #16, #21, #33, #37). β-actin was used as loading control. Data are mean ± S.D. of 3 independent experiments performed with 10 different sphere cell lines. (**I**) Bar graphs of immunoblot band densities for HER2 on enriched CD44v6$^+$ and CD44v6$^-$ *Ras/Braf*-wt, *Braf*- and *Kras*- mutant cells. Data are mean ± S.D. of 3 independent experiments performed in *Ras/Braf*-wt (CSphC#14, 21, 33), *Braf*- (CSphC#1, 2, 3, 5) and *Kras*- (CSphC#8, 11, 16) mutant CD44v6 fractions. (**J**) Immunofluorescence analysis for HER2 and CD44v6 on CD44v6$^+$ and CD44v6$^-$ in *Ras/Braf*-wt (CSphC#21), *Braf*- (CSphC#2) and *Kras*- (CSphC#11) mutant cells. Nuclei were counterstained with TOTO-3. Data are representative of 2 independent experiments performed with *Ras/Braf*-wt (CSphC#14, 21), *Braf*- (CSphC#2, 5) and *Kras*- (CSphC#8, 11) mutant sphere cells. Scale bars, 10 µm. **(K)** Browser view of RNA-seq analysis on *Braf*-mutant CD44v6 positive (green) and negative (red) CSphCs. The tracks of expression (RPKM normalized) for *ERRB2* (*Upper panel*) and *Gapdh* (*Lower panel*) are shown. For each cell type, tracks of three different biological replicates are shown.

Supplementary Figure 2. PI3K activation is associated with increased *Erbb2* transcription levels. (**A**) Unsupervised clustering of RNA-seq data from CD44v6^{high} (>70%) and CD44v6^{low} (<30%) cells. **(B)** LogFC values of a subset of statistically differentially expressed genes, enriched for PI3K pathway, in CD44v6^{high} vs CD44v6^{low} cells. Data were computed by limma package in R. **(C)** *(Upper panels)* Schematic diagram of OFP CRISPR Nuclease and donor DNA (pMA-T) vectors. *(Lower panels)* Electropherograms showing the DNA sequence of *Pik3ca*-wt low expressing HER2 CR-CSphC lines (CSphC#23, 5, 15), following targeted genome editing. Red stars indicate the newly introduced point mutation (red letters G→A). crRNA=CRISPR RNA; tracr=trans-activating crRNA; Pol III=terminator. **(D)** Bar graphs of immunoblot band densities for HER2, pAKT and AKT on *Ras/Braf*-wt (CSphC#6, 23), *Braf*- (CSphC#2, 5), *Kras*- (CSphC#10, 15) mutant cells and their corresponding CRISPR/Cas9-*Pik3ca*^{E545K} cells. Data are mean ± S.D. of 3 independent experiments performed with the indicated CSphCs. **(E)** ChIP-qPCR for the histone marks H3K27Ac using amplicons for 2 promoters (Prom1 and Prom2), 3 potential enhancer regions (Enh1, Enh2, and HGE) and negative control (Control) in IMEC, IMEC-MYC and IMEC-MYC-PI3K cells. Enrichment is indicated as % of input. **(F)** Percentage of viable CD44v6 low (≤30%),

medium (31-69%) and high (≥70%) cells treated with miransertib, BKM120 and taselisib for 72 hours. Boxes and whiskers represent median ± S.D. of 3 experimental replicates of 29 CR-CSphC lines. **(G)** Percentage of cell viability in 29 CR-CSphC lines, harboring the indicated mutations, exposed to different doses of PI3K and AKT inhibitors as indicated. Boxes and whiskers represent median ± S.D. of 3 experimental replicates of 29 CR-CSphC lines. **(H)** Percentage of cell viability of CD44v6$^+$ and CD44v6$^-$ *Braf*- (CSphC#2, 3, 4, 5) and *Kras*- (CSphC#8, 9, 10, 11, 16) mutant sphere cells treated with BKM120. Data are mean ± S.D. from 3 independent experiments. **(I)** mRNA expression levels of *Erbb2* in CSphCs and CD44v6$^+$ enriched cells treated as indicated for 48 hours. Data are mean ± S.D. of 3 independent experiments performed with *Ras/Braf*-wt (CSphC#14, 33), *Braf*- (CSphC#1, 5) and *Kras*- (CSphC#10, 11) mutant sphere cell lines. **(J)** *Erbb2* mRNA expression levels in CSphCs treated with vehicle or taselisib in presence of CAF CM. Data are mean ± S.D. of 3 independent experiments using *Ras/Braf*-wt (CSphC#7, 27), *Braf*- (CSphC#2, 3) and *Kras*- (CSphC#12, 20) mutant sphere cell lines. **(K)** *Erbb2* mRNA expression levels in tumor xenografts treated with vehicle or BKM120 for 4 weeks. Mice were sacrificed 1 weeks after treatment suspension. Data are means ± S.D. of 6 mice per group injected with *Ras/Braf*-wt (CSphC#14, 21, 33), *Braf*- (CSphC#1, 2, 5) or *Kras*- (CSphC#8, 11, 16) mutant sphere cells. (**L)** Representative immunofluorescence analysis for HER2 on tumor xenografts generated by the injection of *Braf*- (CSphC#2) mutant sphere cells and treated as in K. Nuclei were counterstained with Toto-3 (blue color). Scale bars, 20 µm.

Supplementary Figure 3. Triple targeting of HER2, MEK and PI3K overcomes the protective effect mediated by CAF-released cytokines. (A) Size of xenograft tumors generated by subcutaneous injection of *Braf*- (CSphC#1, 2, 3, 5) and *Kras*- (CSphC#8, 11, 16) mutant cells, treated for 4 weeks as indicated and monitored up to 10 weeks. "I" indicates the time of cell injection. Time-point 0 indicates the start of treatment. Data are mean ± S.D. of 3 independent experiments (n=6 mice per group). **(B)** RPPA analysis of *Ras/Braf*-wt (CSphC#14), *Braf*- (CSphC#2) and *Kras*- (CSphC#11) mutant cells treated with vehicle or vemurafenib (Vemu) in combination with trastuzumab (Tmab) and PI3K inhibitor (BKM120) (VTB). Total and phosphorilated S6 are indicated. **(C)** Relative band densities of immunoblots for pAKT, AKT, pGSK, GSK3, pERK, ERK and Myc in tumor xenografts-derived cells of mice injected with *Ras/Braf*-wt (CSphC#14, 21, 33), *Braf*- (CSphC#1, 2, 3), *Kras*- (CSphC#8, 11, 16) mutant sphere cells treated with vehicle (Vehicle) or vemurafenib (Vemu) in combination with trastuzumab (Tmab) and PI3K inhibitor (BKM120) (V+T+B). Data are expressed as mean ± S.D. of 3 subcutaneously implanted CSphC lines for each mutational status (n= 6 mice per group). **(D)** (*Left panel*) Representative average of miRNAs equivalent CT values on tumor xenograft-derived cells isolated from mice injected with *Kras*- (CSC#8) mutant CR-CSphCs treated with vehicle (Vehicle) or vemurafenib in combination with trastuzumab plus PI3K inhibitor (Vemu+Tmab+BKM120). (*Right panel*) Clustergrams of miRNAs expression levels of those indicated with arrows in the correlation plot. Data are representative of 3 independent experiments performed with 3 different CR-CSphCs (CSC#8, 11, 16). **(E)** Bar graphs of immunoblot relative band densities for pAKT, AKT, pGSK, GSK, pERK, ERK and Myc in *Ras/Braf*-wt, *Braf*- and *Kras*- mutant sphere cells treated with vehicle (Vehicle) or trastuzumab (Tmab) in combination with trametinib (MEKi) and PI3K inhibitor (BKM120) (T+Mi+B). Data are mean ± S.D. of 3 independent experiments performed with *Ras/Braf*-wt (CSphC#14, 21, 33), *Braf*- (CSphC#1, 2, 3) and *Kras*- (CSphC#8, 11, 16) mutant sphere cultures. **(F)** RPPA analysis of *Braf*- (CSphC#2) and *Kras*- (CSphC#11) mutant cells treated with vehicle or trastuzumab (Tmab) in combination with trametinib (MEKi) and PI3K inhibitor (BKM120) (TMiB) for 24 hours. Total and phosphorilated S6 expression levels are indicated. **(G)** Heatmap of viability percentage of cells with the indicated mutational background treated with vehicle (Vehicle) or trastuzumab in combination with MEKi and BKM120 (T+Mi+B) for 72 and 120 hours. Data are the mean of 3 experimental replicates performed on 30 cell lines. **(H)** (*Left panel*) Representative average of miRNAs equivalent CT values on *Kras*- (CSC#11) mutant CR-

CSphCs, exposed to the indicated therapeutic regimen combinations. Before treating with trastuzumab in combination with MEK inhibitor and BKM120 (Tmab+MEKi+BKM120) for 24 hours, cells were pre-treated with vemurafenib in combination with trastuzumab and BKM120 for 5 days and maintained for 2 days off-drug period. (*Right panel*) Clustergrams of miRNAs expression levels, indicated with arrows in the correlation plot. Data are representative of 3 independent experiments performed with different CR-CSphCs (CSC#8, 11, 16). **(I)** Bar graphs of immunoblot relative band densities for pAKT, AKT, pMEK, MEK, pERK, ERK and Myc in *Braf*- (#1, 2, 3) *Kras*- (CSphC#8,11, 16) mutant sphere cells treated with vehicle (Vehicle) or vemurafenib (V) in combination with trastuzumab (T) and BKM120 (V+T+B) or trastuzumab in combination with MEKi and BKM120 (T+Mi+B) and cultured in FBS-free DMEM or CAF CM for 24 hours. Data are expressed as mean ± S.D. of 3 independent experiments. **(J)** Growth of cells previously untreated (Vehicle) or treated with vemurafenib in combination with trastuzumab and BKM120 (Pre-treated V+T+B) for 5 days and exposed to trastuzumab in combination with MEKi and BKM120 (T+Mi+B) or V+T+B. Data are expressed as mean ± S.D. of 4 independent experiments performed with 15 CR-CSphCs with different mutational backgrounds.

Supplementary Figure 4. The triple targeting of MEK, HER2 and PI3K induces regression of xenograft tumors generated by the injection of CR-CSCs. (A) Subcutaneous size of tumor xenografts generated by sphere cell lines bearing the indicated mutational background and treated as indicated. Time-point "I" indicates cell injection and 0 the start of treatment. Data are mean ± S.D. of 3 independent experiments (n=6 mice per group). **(B)** H&E and immunohistochemical analysis of CD44v6, Ki67 and CK20 on tumor xenografts generated by the injection of *Braf*- (CSphC#2) mutant sphere cells. Mice were treated for 4 weeks with vehicle (Vehicle) or trastuzumab (Tmab) plus trametinib (MEKi) plus BKM120 (BKM120). **(C)** Evaluation of CD44v6 positivity by flow cytometry in CR-CSphCs obtained from *Ras/Braf*-wt, *Braf*- and *Kras*- mutant xenografts treated as indicated, and analyzed 2 weeks after treatment suspension. Data are expressed as mean ± S.D. of 3 independent experiments performed with *Ras/Braf*-wt (CSphC#21, 24, 33), *Braf*- (CSphC#2, 3, 5) and *Kras*- (CSphC#8, 11, 13) mutant cells. **(D)** Cell viability of the indicated sphere cells exposed for 72 and 120 hours to trastuzumab (Tmab) in combination with cobimetinib (Cob) and taselisib (Tas). Data are expressed as mean ± S.D. of 4 independent experiments. **(E)** Subcutaneous outgrowth of *Kras*- (CSphC#8, 9, 11, 13) mutant sphere cell-derived xenograft tumors at the indicated weeks and treated with vehicle (Vehicle) or trastuzumab (Tmab) in combination with cobimetinib (Cob) and taselisib (Tas) for 4 weeks. Data are means ± S.D. of tumor size for each cell line (n= 6 mice per group). "I" indicates the time of cell injection and 0 the start of treatment. **(F)** CD44v6 flow cytometry analysis on cells dissociated from tumor xenografts treated as in (E). Grey histograms represent the relative isotype matched control. **(G)** Representative azan mallory staining and immunohistochemical analysis of CD44v6, CK20 and Ki67 on tumor xenografts obtained from the injection of *Kras*- (CSphC#9, 11) mutant sphere cells treated as indicated. Scale bars, 100 µm.

REFERENCES

1 Todaro M, Alea MP, Di Stefano AB, Cammareri P, Vermeulen L, Iovino F*, et al.* Colon cancer stem cells dictate tumor growth and resist cell death by production of interleukin-4. Cell stem cell 2007;**1**:389-402.

2 Gevensleben H, Garcia-Murillas I, Graeser MK, Schiavon G, Osin P, Parton M*, et al.* Noninvasive detection of HER2 amplification with plasma DNA digital PCR. Clinical cancer research : an official journal of the American Association for Cancer Research 2013;**19**:3276-84.

3 Takegawa N, Yonesaka K, Sakai K, Ueda H, Watanabe S, Nonagase Y*, et al.* HER2 genomic amplification in circulating tumor DNA from patients with cetuximab-resistant colorectal cancer. Oncotarget 2016;**7**:3453-60.

4 Li J, Zhao W, Akbani R, Liu W, Ju Z, Ling S*, et al.* Characterization of Human Cancer Cell Lines by Reverse-phase Protein Arrays. Cancer cell 2017;**31**:225-39.

5 Fagnocchi L, Cherubini A, Hatsuda H, Fasciani A, Mazzoleni S, Poli V*, et al.* A Myc-driven self-reinforcing regulatory network maintains mouse embryonic stem cell identity. Nature communications 2016;**7**:11903.

6 Poli V, Fagnocchi L, Fasciani A, Cherubini A, Mazzoleni S, Ferrillo S*, et al.* MYC-driven epigenetic reprogramming favors the onset of tumorigenesis by inducing a stem cell-like state. Nature communications 2018;**9**:1024.
