# Supplementary material for: PI3K-driven HER2 expression is a potential therapeutic target in colorectal cancer stem cells
Source: Gut. 2021 Jan 12;71(1):119–28. doi: 10.1136/gutjnl-2020-323553 (PMC8666826; doi:10.1136/gutjnl-2020-323553)
Supplement: Supplementary data [file gutjnl-2020-323553supp009.pdf]

Supplementary Table 4: RPPA antibody validation.

Validation Status*	*RPPA Set 131-Present*
Valid = RPPA and WB correlation > 0.7	
Use with Caution = RPPA and WB correlation < 0.7	
Under Evaluation = Antibody has given mixed results and/or evaluated by another lab; We are in the process of (re)validating	
Used for QC = These antibodies are used for tissue sample quality control (QC); WILL NOT report to Datasets containing tissue samples, but WILL report to Datasets containing cell line samples	

THE UNIVERSITY OF TEXAS MD Anderson Cancer Center Making Cancer History

#	Official Ab Name	Ab Name Reported on Dataset	Gene Name	Company	Catalog #	Internal Ab ID	Species	Validation Status*	RPPA Dilution	Storage
1	14-3-3 beta	14-3-3-beta	YWHAB	Santa Cruz	sc-628	882	Rabbit	Valid	1:75	4
2	14-3-3 epsilon	14-3-3-epsilon	YWHAE	Santa Cruz	sc-23957	913	Mouse	Use with Caution	1:50	4
3	14-3-3 zeta	14-3-3-zeta	YWHAZ	Santa Cruz	sc-1019	883	Rabbit	Valid	1:5000	4
4	4E-BP1	4E-BP1	EIF4EBP1	CST	9452	2	Rabbit	Valid	1:100	-20
5	4E-BP1 (phospho S65)	4E-BP1_pS65	EIF4EBP1	CST	9456	3	Rabbit	Valid	1:250	-20
6	53BP1	53BP1	TP53BP1	CST	4937	985	Rabbit	Valid	1:300	-20
7	Acetyl CoA Carboxylase (phospho S79)	ACC_pS79	ACACA, ACACB	CST	3661	13	Rabbit	Valid	1:500	-20
8	Acetyl CoA Carboxylase 1	ACC1	ACACA	Abcam	ab45174	14	Rabbit	Use with Caution	1:20000	-20
9	ADAR1	ADAR1	ADAR	Abcam	ab88574	1198	Mouse	Valid	1:200	-20
10	Akt	Akt	AKT1,2,3	CST	4691	1084	Rabbit	Valid	1:10000	-20
11	Akt (phospho S473)	Akt_pS473	AKT1,2,3	CST	9271	230	Rabbit	Valid	1:150	-20
12	Akt (phospho T308)	Akt_pT308	AKT1,2,3	CST	2965	1154	Rabbit	Valid	1:500	-20
13	AMPK alpha	AMPKa	PRKAA1	CST	2532	39	Rabbit	Use with Caution	1:200	-20
14	AMPK alpha (phospho T172)	AMPKa_pT172	PRKAA1	CST	2535	40	Rabbit	Use with Caution	1:100	-20
15	AMPK alpha 2 (Phospho S345)	AMPK-a2_pS345	PRKAA2	Abcam	ab129081	1351	Rabbit	Valid	1:500	-20
16	Androgen Receptor	AR	AR	Abcam	ab52615	756	Rabbit	Valid	1:100	-20
17	Annexin I	Annexin-I	ANXA1	BD Biosciences	610066	1208	Mouse	Valid	1:5000	-20
18	Annexin VII	Annexin-VII	ANXA7	BD Biosciences	610668	1142	Mouse	Valid	1:30	-20
19	A-Raf	A-Raf	ARAF	CST	4432	1217	Rabbit	Valid	1:150	-20
20	ARID1A	ARID1A	ARID1A	Sigma-Aldrich	HPA005456	1442	Rabbit	Use with Caution	1:1000	-20
21	Atg3	Atg3	ATG3	CST	3415	1612	Rabbit	Valid	1:750	-20
22	Atg7	Atg7	ATG7	CST	8558	1613	Rabbit	Valid	1:1000	-20
23	ATM	ATM	ATM	CST	2873	1363	Rabbit	Valid	1:250	-20
24	ATM (phospho S1981)	ATM_pS1981	ATM	CST	5883	1364	Rabbit	Valid	1:25	-20
25	ATR (Phospho S428)	ATR_pS428	ATR	Abcam	ab178407	1795	Rabbit	Use with Caution	1:1000	-20
26	ATRX	ATRX	ATRX	Abcam	ab97508	1569	Rabbit	Use with Caution	1:1000	-20
27	Aurora B/AIM1	Aurora-B	AIM1	CST	3094	1404	Rabbit	Valid	1:50	-20
28	Axl	Axl	AXL	CST	8661	1271	Rabbit	Valid	1:1000	-20
29	B7-H4	B7-H4	VTCN1	CST	14572	1726	Rabbit	Use with Caution	1:50	-20
30	Bad (phospho S112)	Bad_pS112	BAD	CST	9291	63	Rabbit	Valid	1:50	-20
31	Bak	Bak	BAK1	Abcam	ab32371	71	Rabbit	Use with Caution	1:30	-20
32	BAP1	BAP1	BAP1	Santa Cruz	sc-28383	1207	Mouse	Valid	1:125	4
33	Bax	Bax	BAX	CST	2772	73	Rabbit	Valid	1:100	-20
34	Bcl2	Bcl2	BCL2	Dako	M0887	80	Mouse	Valid	1:50	4
35	Bcl2A1	Bcl2A1	BCL2A1	Abnova	PAB8528	1299	Rabbit	Valid	1:250	-20
36	Bcl-xL	Bcl-xL	BCL2L1	CST	2762	85	Rabbit	Valid	1:100	-20
37	Beclin	Beclin	BECN1	Santa Cruz	sc-10086	87	Goat	Use with Caution	1:250	4
38	beta Actin	b-Actin	ACTB	CST	4970	1169	Rabbit	Use with Caution	1:75	-20
39	beta Catenin	b-Catenin	CTNNB1	CST	9562	75	Rabbit	Valid	1:1500	-20
40	beta Catenin (phospho T41/S45)	b-Catenin_pT41_S45	CTNNB1	CST	9565	1170	Rabbit	Valid	1:30	-20
41	Bid	Bid	BID	Abcam	ab32060	88	Rabbit	Use with Caution	1:30	-20
42	Bim	Bim	BCL2L11	Abcam	ab32158	90	Rabbit	Valid	1:400	-20
43	BiP/GRP78	BiP-GRP78	HSPA5	BD Biosciences	610978	1311	Mouse	Use with Caution	1:750	-20
44	B-Raf	B-Raf	BRAF	CST	14814	2083	Rabbit	Use with Caution	1:500	-20
45	B-Raf (phospho S445)	B-Raf_pS445	BRAF	CST	2696	94	Rabbit	Valid	1:1000	-20
46	BRD4	BRD4	BRD4	CST	13440	1567	Rabbit	Valid	1:200	-20
47	c-Abl	c-Abl	ABL	CST	2862	1565	Rabbit	Valid	1:50	-20
48	c-IAP2	c-IAP2	BIRC3	CST	3130	1615	Rabbit	Use with Caution	1:750	-20
49	Caspase-3 active	Caspase-3	CASP3	Abcam	ab32042	108	Rabbit	Use with Caution	1:250	-20
50	Caspase-7 (cleaved D198)	Caspase-7-cleaved	CASP7	CST	9491	109	Rabbit	Use with Caution	1:75	-20
51	Caspase-8	Caspase-8	CASP8	CST	9746	951	Mouse	**Used for QC**	1:150	-20
52	Caveolin-1	Caveolin-1	CAV1	CST	3238	114	Rabbit	Valid	1:5000	-20
53	CD171 (L1)	CD171	L1CAM	BioLegend	826701	1737	Mouse	Valid	1:1000	4
54	CD26	CD26	CD26	Abcam	ab28340	1308	Rabbit	Valid	1:1000	-20
55	CD29	CD29	ITGB1	BD Biosciences	610467	1206	Mouse	Valid	1:30	-20
56	CD31	CD31	PECAM1	Dako	M0823	127	Mouse	Valid	1:30	4
57	CD44	CD44	CD44	CST	3570	1398	Mouse	Use with Caution	1:50	-20
58	CD49b	CD49b	ITGA2	BD Biosciences	611016	937	Mouse	Valid	1:50	-20
59	cdc2 (Phospho Y15)	cdc2_pY15	CDK1	CST	4539	1783	Rabbit	Use with Caution	1:250	-20
60	cdc25C	cdc25C	CDC25C	CST	4688	1873	Rabbit	Valid	1:500	-20
61	CDK1	CDK1	CDK1	Abcam	ab32384	1658	Rabbit	Use with Caution	1:1000	-20
62	CDKN2A/p16INK4a	p16INK4a	CDKN2A	Abcam	ab81278	1231	Rabbit	Valid	1:500	-20
63	Chk1	Chk1	CHEK1	CST	2360	1203	Mouse	Use with Caution	1:250	-20
64	Chk1 (phospho S296)	Chk1_pS296	CHEK1	Abcam	ab79758	1348	Rabbit	Valid	1:125	-20
65	Chk2	Chk2	CHEK2	CST	3440	146	Mouse	Valid	1:50	-20
66	Chk2 (phospho T68)	Chk2_pT68	CHEK2	CST	2197	147	Rabbit	Use with Caution	1:125	-20
67	c-Jun (phospho S73)	c-Jun_pS73	JUN	CST	9164	155	Rabbit	Valid	1:30	-20
68	c-Kit	c-Kit	KIT	Abcam	ab32363	157	Rabbit	Valid	1:30	-20
69	Claudin 7	Claudin-7	CLDN7	Novus Biologicals	NB100-91714	852	Rabbit	Valid	1:300	-20
70	c-Met	c-Met	MET	CST	3127	726	Mouse	**Used for QC**	1:250	-20
71	c-Met (phospho Y1234/Y1235)	c-Met_pY1234_Y1235	MET	CST	3129	727	Rabbit	Valid	1:100	-20
72	c-Myc	c-Myc	MYC	Santa Cruz	sc-764	1143	Rabbit	Use with Caution	1:125	4
73	COG3	COG3	COG3	ProteinTech	11130-1-AP	1656	Rabbit	Valid	1:750	-20
74	COL6A1	Collagen-VI	COL6A1	Santa Cruz	sc-20649	171	Rabbit	Valid	1:5000	4
75	Connexin 43	Connexin-43	CNST43	CST	3512	1568	Rabbit	Use with Caution	1:150	-20
76	Cox2	Cox2	PTGS2	CST	4842	1218	Rabbit	Use with Caution	1:50	-20
77	Cox-IV	Cox-IV	PTGS3	CST	4850	1116	Rabbit	Valid	1:1000	-20
78	C-Raf (phospho S338)	C-Raf_pS338	RAF1	CST	9427	179	Rabbit	Valid	1:100	-20

79	C-Raf/Raf-1	C-Raf	RAF1	Millipore	04-739	1201	Rabbit	Use with Caution	1:200	-20
80	CREB	CREB	CREB1	CST	9197	181	Rabbit	Use with Caution	1:1000	-20
81	Cyclin B1	Cyclin-B1	CCNB1	Epitomics	1495-1	192	Rabbit	Valid	1:1500	-20
82	Cyclin D1	Cyclin-D1	CCND1	Santa Cruz	sc-718	194	Rabbit	Valid	1:200	4
83	Cyclin D3	Cyclin-D3	CCND3	CST	2936	198	Mouse	Valid	1:1000	-20
84	Cyclin E1	Cyclin-E1	CCNE1	Santa Cruz	sc-247	201	Mouse	Valid	1:30	4
85	Cyclophilin F	Cyclophilin-F	PPIF	Abcam	ab110324	1257	Mouse	Valid	1:50000	4
86	Detyrosinated alpha-Tubulin	D-a-Tubulin	TUBA1A	Abcam	ab48389	1379	Rabbit	Valid	1:500	-20
87	Dimethyl-Histone H3 (Lys4)	DM-Histone-H3	HISTH3	Millipore	07-030	1380	Rabbit	Valid	1:1500	-20
88	Dimethyl-K9 Histone H3	DM-K9-Histone-H3	H3K9ME2	Abcam	ab32521	1397	Rabbit	Use with Caution	1:250	-20
89	DUSP4/MKP2	DUSP4	DUSP4	CST	5149	1406	Rabbit	Valid	1:250	-20
90	E2F-1	E2F1	E2F1	Santa Cruz	sc-251	1261	Mouse	Valid	1:30	4
91	E-Cadherin	E-Cadherin	CDH1	CST	3195	1099	Rabbit	Valid	1:300	-20
92	eEF2	eEF2	EEF2	CST	2332	1060	Rabbit	Use with Caution	1:50	-20
93	eEF2K	eEF2K	EEF2K	CST	3692	1061	Rabbit	Valid	1:50	-20
94	EGFR	EGFR	EGFR	CST	2232	1120	Rabbit	Valid	1:100	-20
95	EGFR (phospho Y1173)	EGFR_pY1173	EGFR	Abcam	ab32578	221	Rabbit	Valid	1:50	-20
96	eIF4E	eIF4E	EIF4E	CST	9742	722	Rabbit	Valid	1:75	-20
97	eIF4E (Phospho S209)	eIF4E_pS209	EIF4E	Abcam	ab76256	1871	Rabbit	Valid	1:500	-20
98	eIF4G	eIF4G	EIF4G1	CST	2498	1124	Rabbit	Use with Caution	1:1000	-20
99	Elk1 (phospho S383)	Elk1_pS383	ELK1	CST	9181	228	Rabbit	Use with Caution	1:50	-20
100	ENY2	ENY2	ENY2	GeneTex	GTX629542	1219	Mouse	Use with Caution	1:1000	-20
101	Epithelial Membrane Antigen	EMA	EMA	Dako	M061329-2	1350	Mouse	Use with Caution	1:1000	4
102	ErbB2/HER2	HER2	ERBB2	Lab Vision	MS-325-P1	1038	Mouse	Valid	1:3000	4
103	ErbB2/HER2 (phospho Y1248)	HER2_pY1248	ERBB2	R&D Systems	AF1768	1075	Rabbit	Use with Caution (likely sees pEGFR)	1:1500	-20
104	ErbB3/HER3	HER3	ERBB3	Santa Cruz	sc-285	911	Rabbit	Valid	1:300	4
105	ErbB3/HER3 (phospho Y1289)	HER3_pY1289	ERBB3	CST	4791	728	Rabbit	Use with Caution	1:50	-20
106	ERCC1	ERCC1	ERCC1	Santa Cruz	sc-17809	1357	Mouse	Valid	1:30	4
107	ERCC5	ERCC5	ERCC5	ProteinTech	11331-1-AP	1355	Rabbit	Use with Caution	1:250	-20
108	ERRFI1/MIG6	MIG6	ERRFI1	Sigma-Aldrich	WH0054206M1	1062	Mouse	Valid	1:50	-20
109	Estrogen Receptor	ER	ESR1	Lab Vision	RM-9101	238	Rabbit	Valid	1:40	4
110	Estrogen Receptor alpha (Phospho S118)	ER-a_pS118	ESR1	Abcam	ab32396	241	Rabbit	Valid	1:1000	-20
111	Ets-1	Ets-1	ETS1	Bethyl	A303-501A	1200	Rabbit	Valid	1:100	4
112	FAK	FAK	PTK2	Abcam	ab40794	252	Rabbit	Use with Caution	1:1000	-20
113	FAK (phospho Y397)	FAK_pY397	PTK2	CST	3283	1227	Rabbit	Valid	1:30	-20
114	Fatty Acid Synthase	FASN	FASN	CST	3180	1156	Rabbit	Valid	1:1000	-20
115	Fibronectin	Fibronectin	FN1	Epitomics	1574-1	262	Rabbit	Valid	1:10000	-20
116	FoxM1	FoxM1	FOXM1	CST	5436	1123	Rabbit	Valid	1:30	-20
117	FoxO3a	FoxO3a	FOXO3	CST	2497	1122	Rabbit	Use with Caution	1:25	-20
118	FoxO3a (phospho S318/S321)	FoxO3a_pS318_S321	FOXO3	CST	9465	270	Rabbit	Use with Caution	1:30	-20
119	FRA-1	FRA-1	FRA1	Santa Cruz	sc-605	1184	Rabbit	Use with Caution	1:100	4
120	G6PD	G6PD	G6PD	CST	8866	1779	Rabbit	Valid	1:1000	-20
121	Gab2	Gab2	GAB2	CST	3239	943	Rabbit	Valid	1:300	-20
122	GAPDH	GAPDH	GAPDH	Life Technologies	AM4300	274	Mouse	Use with Caution	1:50000	-20
123	GATA3	GATA3	GATA3	BD Biosciences	558686	764	Mouse	Valid	1:300	4
124	GCLM	GCLM	GCLM	Abcam	ab124827	1745	Rabbit	Use with Caution	1:500	-20
125	GCN5L2	GCN5L2	KAT2A	CST	3305	1263	Rabbit	Valid	1:30	-20
126	Glutamate Dehydrogenase1/2	Glutamate-D1-2	GLUD	CST	12793	1617	Rabbit	Use with Caution	1:500	-20
127	Glutaminase	Glutaminase	GLS	Abcam	ab156876	1491	Rabbit	Use with Caution	1:250	-20
128	Glycogen Synthase	Gys	GYS1	CST	3886	1035	Rabbit	Valid	1:1500	-20
129	Glycogen Synthase (phospho S641)	Gys_pS641	GYS1	CST	3891	1036	Rabbit	Valid	1:250	-20
130	Granzyme B	Granzyme-B	GZMB	CST	4275	1807	Rabbit	Valid	1:500	-20
131	GSK-3alpha/beta	GSK-3a-b	GSK3A, GSK3B	Santa Cruz	sc-7291	284	Mouse	Valid	1:750	4
132	GSK-3alpha/beta (phospho S21/S9)	GSK-3a-b_pS21_S9	GSK3A, GSK3B	CST	9331	285	Rabbit	Valid	1:200	-20
133	H2AX (phospho S140)	H2AX_pS140	H2AX	Pierce Biotechnology	MA1-2022	1409	Mouse	Use with Caution	1:400	-20
134	Heregulin	Heregulin	NRG1	CST	2573	890	Rabbit	Valid	1:30	-20
135	HES1	HES1	HES1	CST	11988	1582	Rabbit	Valid	1:1000	-20
136	Hexokinase II	Hexokinase-II	HK2	CST	2867	1023	Rabbit	Valid	1:50	-20
137	Hif-1 alpha	Hif-1-alpha	HIF1A	BD Biosciences	610958	1402	Mouse	Use with Caution	1:50	-20
138	Histone H3	Histone-H3	H3F3A, H3F3B	Abcam	ab1791	1250	Rabbit	Valid	1:3000	-20
139	HSP27	HSP27	HSP27	CST	2402	321	Mouse	Use with Caution	1:100	-20
140	HSP27 (phospho S82)	HSP27_pS82	HSBP1	CST	2401	323	Rabbit	Valid	1:75	-20
141	HSP70	HSP70	HSP70	CST	4872	325	Rabbit	Use with Caution	1:150	-20
142	IGF1R (phospho Y1135/Y1136)	IGF1R_pY1135_Y1136	IGF1R	CST	3024	1221	Rabbit	Valid	1:30	-20
143	IGFBP2	IGFBP2	IGFBP2	CST	3922	335	Rabbit	Valid	1:50	-20
144	IGFRb	IGFRb	INSR	CST	3027	336	Rabbit	Use with Caution	1:250	-20
145	INPP4b	INPP4b	INPP4B	CST	4039	1065	Rabbit	Valid	1:25	-20
146	Insulin Receptor beta	IR-b	INSRB	CST	3025	1586	Rabbit	Use with Caution	1:750	-20
147	IRF-1	IRF-1	IRF1	Santa Cruz	sc-497	1316	Rabbit	Use with Caution	1:200	4
148	IRS1	IRS1	IRS1	Millipore	06-248	802	Rabbit	Valid	1:400	-20
149	Jagged1	Jagged1	JAG1	Abcam	ab109536	1413	Rabbit	Valid	1:750	-20
150	Jak2	Jak2	JAK2	CST	3230	1166	Rabbit	Valid	1:750	-20
151	JNK/SAPK (phospho T183/Y185)	JNK_pT183_Y185	MAPK8	CST	4668	888	Rabbit	Valid	1:30	-20
152	JNK2	JNK2	MAPK9	CST	4672	380	Rabbit	Use with Caution	1:30	-20
153	LC3A/B	LC3A-B	LC3AB	CST	4108	1618	Rabbit	Use with Caution	1:500	-20
154	Lck	Lck	LCK	CST	2752	397	Rabbit	Valid	1:100	-20
155	LDHA	LDHA	LDHA	CST	3582	976	Rabbit	Use with Caution	1:250	-20
156	LRP6 (phospho S1490)	LRP6_pS1490	LRP6	CST	2568	1367	Rabbit	Valid	1:500	-20
157	MAPK (phospho pT202_Y204)	MAPK_pT202_Y204	MAPK1, MAPK3	CST	4377	405	Rabbit	Valid	1:30	-20
158	Mcl 1	Mcl-1	MCL1	CST	5453	1222	Rabbit	Valid	1:100	-20
159	MDM2 (phospho S166)	MDM2_pS166	MDM2	CST	3521	1164	Rabbit	Valid	1:50	-20
160	MEK1	MEK1	MAP2K1	Abcam	ab32576	417	Rabbit	Valid	1:1500	-20
161	MEK1 (phospho S217/S221)	MEK1_pS217_S221	MAP2K1 MAP2K2	CST	9154	1076	Rabbit	Valid	1:50	-20
162	MERIT40 (Phospho S29)	MERIT40_pS29	BABAM1	CST	12110	1952	Rabbit	Valid	1:1000	-20
163	Merlin/NF2	Merlin	NF2	Novus Biologicals	22710002	1046	Rabbit	Use with Caution	1:250	-20
164	MIF	MIF	MIF	Santa Cruz	sc-20121	1323	Rabbit	Use with Caution	1:300	4
165	MMP2	MMP2	MMP2	CST	4022	435	Rabbit	Valid	1:75	-20

166	Mnk1	Mnk1	MKNK1	CST	2195	1005	Rabbit	Valid	1:1000	-20
167	Monocarboxylic Acid Transporter 4	MCT4	SLC16A4	Millipore	AB3314P	1633	Rabbit	Valid	1:500	-20
168	MSH6	MSH6	MSH6	Novus Biologicals	22030002	1063	Rabbit	Use with Caution	1:1000	-20
169	MSI2	MSI2	MSI2	Abcam	ab76148	1675	Rabbit	Use with Caution	1:4000	-20
170	mTOR	mTOR	MTOR	CST	2983	444	Rabbit	Valid	1:1000	-20
171	mTOR (phospho S2448)	mTOR_pS2448	MTOR	CST	2971	446	Rabbit	Use with Caution	1:50	-20
172	Myosin heavy chain 11	Myosin-11	MYH11	Novus Biologicals	21370002	1139	Rabbit	Valid	1:5000	-20
173	Myosin IIa (phospho S1943)	Myosin-IIa_pS1943	MYH9	CST	5026	1160	Rabbit	Valid	1:1000	-20
174	Myt1	Myt1	MYT1	CST	4282	1803	Rabbit	Use with Caution	1:2000	-20
175	NAPSIN A	NAPSIN-A	NAPSA	Abcam	ab129189	1274	Rabbit	Use with Caution	1:150	-20
176	N-Cadherin	N-Cadherin	CDH2	CST	4061	452	Rabbit	Valid	1:30	-20
177	NDRG1 (phospho T346)	NDRG1_pT346	NDRG1	CST	3217	1126	Rabbit	Valid	1:100	-20
178	NDUFB4	NDUFB4	NDUFB4	Abcam	ab110243	1345	Mouse	Valid	1:30	4
179	NF-kappaB p65 (phospho S536)	NF-kB-p65_pS536	RELA	CST	3033	457	Rabbit	Use with Caution	1:30	-20
180	Notch1	Notch1	NOTCH1	CST	3268	1064	Rabbit	Valid	1:30	-20
181	Notch3	Notch3	NOTCH3	Santa Cruz	sc-5593	767	Rabbit	Use with Caution	1:300	4
182	N-Ras	N-Ras	NRAS	Santa Cruz	sc-31	1136	Mouse	Valid	1:50	4
183	Oct-4	Oct-4	OCT4	CST	2750	1669	Rabbit	Use with Caution	1:200	-20
184	p21	p21	CDKN1A	Santa Cruz	sc-397	470	Rabbit	Valid	1:150	4
185	p27 KIP 1	p27-Kip-1	CDKN1B	Abcam	ab32034	897	Rabbit	Valid	1:50	-20
186	p27/KIP 1 (phospho T198)	p27_pT198	CDKN1B	Abcam	ab64949	878	Rabbit	Valid	1:30	-20
187	p38 MAPK	p38	MAPK14	CST	9212	478	Rabbit	Valid	1:1500	-20
188	p38 MAPK (phospho T180/Y182)	p38_pT180_Y182	MAPK14	CST	9211	479	Rabbit	Valid	1:50	-20
189	p44/42 MAPK	p44-42-MAPK	MAPK3	CST	4695	1119	Rabbit	Valid	1:2000	-20
190	p53	p53	TP53	CST	9282	481	Rabbit	Use with Caution	1:2500	-20
191	p70 S6 Kinase (phospho T389)	p70-S6K_pT389	RPS6KB1	CST	9205	494	Rabbit	Valid	1:50	-20
192	p70/S6K1	p70-S6K1	RPS6KB1	Abcam	ab32529	493	Rabbit	Valid	1:300	-20
193	p90RSK (phospho T573)	p90RSK_pT573	RPS6K	CST	9346	1178	Rabbit	Use with Caution	1:30	-20
194	PAI-1	PAI-1	SERPINE1	BD Biosciences	612024	499	Mouse	Valid	1:100	-20
195	PAICS	PAICS	PAICS	Sigma-Aldrich	HPA035895	1322	Rabbit	Use with Caution	1:250	-20
196	PAK1	PAK1	PAK1	CST	2602	1811	Rabbit	Valid	1:1000	-20
197	PAK4	PAK4	PAK4	CST	3242	1389	Rabbit	Valid	1:750	-20
198	PAR	PAR	PAR	Trevigen	4336-BPC-100	1370	Rabbit	Use with Caution	1:15000	-20
199	PARK7/DJ1	DJ1	PARK7	Abcam	ab76008	891	Rabbit	Valid	1:5000	-20
200	PARP	PARP	PARP1	CST	9532	2185	Rabbit	Valid	1:1000	-20
201	Paxillin	Paxillin	PXN	Epitomics	1500-1	505	Rabbit	Use with Caution	1:500	-20
202	P-Cadherin	P-Cadherin	CDH3	CST	2130	509	Rabbit	Use with Caution	1:50	-20
203	PCNA	PCNA	PCNA	CST	2586	1383	Mouse	Use with Caution	1:1000	-20
204	Pdcd4	Pdcd4	PDCD4	Rockland	600-401-965	816	Rabbit	Use with Caution	1:750	-20
205	PDGFR beta	PDGFR-b	PDGFRB	CST	3169	1225	Rabbit	Valid	1:100	-20
206	PDHK1	PDHK1	PDHK1	CST	3820	1622	Rabbit	Use with Caution	1:500	-20
207	PDK1	PDK1	PDPK1	CST	3062	515	Rabbit	Valid	1:50	-20
208	PDK1 (phospho S241)	PDK1_pS241	PDPK1	CST	3061	516	Rabbit	Valid	1:50	-20
209	PD-L1	PD-L1	CD274	CST	13684	1724	Rabbit	Use with Caution	1:150	-20
210	PEA-15	PEA-15	PEA15	CST	2780	1017	Rabbit	Valid	1:100	-20
211	PED/PEA-15 (phospho S116)	PEA-15_pS116	PEA15	Invitrogen	44-836G	1018	Rabbit	Valid	1:1000	-20
212	PI3 Kinase p110 alpha	PI3K-p110-a	PIK3CA	CST	4255	808	Rabbit	Use with Caution	1:75	-20
213	PI3K p110 beta	PI3K-p110-b	PIK3CB	Santa Cruz	sc-376412	1330	Mouse	Use with Caution	1:60	4
214	PI3K p85	PI3K-p85	PIK3R1	Millipore	06-195	523	Rabbit	Valid	1:15000	-20
215	PKA RI alpha	PKA-a	PRKAR1A	CST	5675	1667	Rabbit	Valid	1:200	-20
216	PKCalpha	PKCa	PRKCA	CST	2056	1158	Rabbit	Valid	1:200	-20
217	PKC beta II (phospho S660)	PKC-b-II_pS660	PRKCA, PRKCB<br>PRKCD, PRKCE<br>PRKCH, PRKCQ	CST	9371	1137	Rabbit	Valid	1:200	-20
218	PKC delta (phospho S664)	PKC-delta_pS664	PRKCD	Millipore	07-875	932	Rabbit	Valid	1:100	-20
219	PKM2	PKM2	PKM2	CST	4053	1025	Rabbit	Use with Caution	1:300	-20
220	PLC gamma2 (phospho Y759)	PLC-gamma2_pY759	PLCG2	CST	3874	1030	Rabbit	Use with Caution	1:30	-20
221	PLK1	PLK1	PLK1	CST	4513	754	Rabbit	Use with Caution	1:125	-20
222	PMS2	PMS2	PMS2	Novus Biologicals	22510002	1246	Rabbit	Valid	1:1500	-20
223	PRAS40	PRAS40	AKT1S1	Invitrogen	AHO1031	738	Mouse	Use with Caution	1:250	-20
224	PRAS40 (phospho T246)	PRAS40_pT246	AKT1S1	Life Technologies	441100G	739	Rabbit	Valid	1:500	-20
225	PREX1	PREX1	PREX1	Abcam	ab102739	1204	Rabbit	Valid	1:150	-20
226	Progesterone Repector	PR	PGR	Abcam	ab32085	549	Rabbit	Valid	1:50	-20
227	PTEN	PTEN	PTEN	CST	9552	566	Rabbit	Valid	1:500	-20
228	Rab11	Rab11	RAB11A,B	CST	3539	1083	Rabbit	Under Evaluation	1:30	-20
229	Rab25	Rab25	RAB25	CST	4314	1150	Rabbit	Valid	1:30	-20
230	Rad50	Rad50	RAD50	Millipore	05-525	987	Mouse	Valid	1:100	-20
231	Rad51	Rad51	RAD51	CST	8875	1262	Rabbit	Valid	1:30	-20
232	Raptor	Raptor	RPTOR	CST	2280	1128	Rabbit	Valid	1:300	-20
233	Rb	Rb	RB1	CST	9309	552	Mouse	**Used for QC**	1:100	-20
234	Rb (phospho S807/S811)	Rb_pS807_S811	RB1	CST	9308	557	Rabbit	Valid	1:500	-20
235	RBM15	RBM15	RBM15	Novus Biologicals	21390002	1138	Rabbit	Valid	1:5000	-20
236	Rheb	Rheb	RHEB	R&D Systems	MAB3426	847	Mouse	Use with Caution	1:75	-20
237	Rictor	Rictor	RICTOR	CST	2114	1129	Rabbit	Use with Caution	1:100	-20
238	Rictor (phospho T1135)	Rictor_pT1135	RICTOR	CST	3806	1130	Rabbit	Valid	1:200	-20
239	RIP	RIP	RIP	CST	4926	1624	Rabbit	Use with Caution	1:250	-20
240	Rock-1	Rock-1	ROCK1	Santa Cruz	sc-5560	1334	Rabbit	Use with Caution	1:1000	4
241	RPA32	RPA32	RPA32	CST	2208	1368	Rat	Use with Caution	1:500	-20
242	RPA32 (Phospho S4/S8)	RPA32_pS4_S8	RPA32	Bethyl	A300-245A	1375	Rabbit	Use with Caution	1:250	4
243	RSK	RSK	RPS6KA1<br>RPS6KA2<br>RPS6KA3	CST	9347	759	Rabbit	Use with Caution	1:150	-20
244	S6 (phospho S235/S236)	S6_pS235_S236	RPS6	CST	2211	600	Rabbit	Valid	1:2500	-20
245	S6 (phospho S240/S244)	S6_pS240_S244	RPS6	CST	2215	601	Rabbit	Valid	1:1000	-20
246	S6 Ribosomal Protein	S6	RPS6	CST	2317	1874	Mouse	Valid	1:1000	-20
247	SCD	SCD	SCD	Santa Cruz	sc-58420	1127	Mouse	Valid	1:30	4
248	SDHA	SDHA	SDHA	CST	11998	1339	Rabbit	Valid	1:250	-20
249	SF2/ASF	SF2	SRSF1	Invitrogen	32-4500	1131	Mouse	Valid	1:150	-20
250	Shc (phospho Y317)	Shc_pY317	SHC1	CST	2431	1031	Rabbit	Valid	1:30	-20

251	SHP-2 (phospho Y542)	SHP-2_pY542	PTPN11	CST	3751	1180	Rabbit	Use with Caution	1:75	-20
252	SLC1A5	SLC1A5	SLC1A5	Sigma-Aldrich	HPA035240	1313	Rabbit	Use with Caution	1:15000	-20
253	Slfn11	Slfn11	SLFN11	Santa Cruz	sc-136891	1411	Goat	Use with Caution	1:250	4
254	Smac/Diablo	Smac	DIABLO	CST	2954	610	Mouse	**Used for QC**	1:150	-20
255	Smad1	Smad1	SMAD1	Abcam	ab33902	922	Rabbit	Valid	1:750	-20
256	Smad3	Smad3	SMAD3	Abcam	ab40854	796	Rabbit	Valid	1:150	-20
257	Smad4	Smad4	SMAD4	Santa Cruz	sc-7966	920	Mouse	Valid	1:30	4
258	Snail	Snail	SNAI1	CST	3895	616	Mouse	**Used for QC**	1:50	-20
259	SOD1	SOD1	SOD1	CST	4266	1818	Mouse	Valid	1:500	-20
260	SOD2	SOD2	SOD2	CST	13141	1328	Rabbit	Valid	1:2500	-20
261	Sox2	Sox2	SOX2	CST	2748	1670	Rabbit	Valid	1:200	-20
262	Src	Src	SRC	Millipore	05-184	621	Mouse	Valid	1:200	-20
263	Src (phospho Y527)	Src_pY527	SRC, YES1, FYN FGR	CST	2105	626	Rabbit	Valid	1:30	-20
264	Src Family (phospho Y416)	Src_pY416	SRC, LYN, FYN LCK, YES1, HCK	CST	2101	623	Rabbit	Valid	1:500	-20
265	Stat3	Stat3	STAT3	CST	4904	1197	Rabbit	Use with Caution	1:3000	-20
266	Stat3 (phospho Y705)	Stat3_pY705	STAT3	CST	9131	637	Rabbit	Valid	1:30	-20
267	Stat5a	Stat5a	STAT5A	Abcam	ab32043	638	Rabbit	Valid	1:250	-20
268	Stathmin 1	Stathmin-1	STMN1	Abcam	ab52630	718	Rabbit	Valid	1:75	-20
269	Syk	Syk	SYK	Santa Cruz	sc-1240	1033	Mouse	Valid	1:3000	4
270	Tau	Tau	TAU	Millipore	05-348	646	Mouse	Use with Caution	1:100	-20
271	TAZ	TAZ	WWTR1	CST	4883	1848	Rabbit	Valid	1:300	-20
272	TFAM	TFAM	TFAM	CST	7495	1333	Rabbit	Valid	1:300	-20
273	TIGAR	TIGAR	C12ORF5	Abcam	ab137573	1107	Rabbit	Valid	1:100	-20
274	Transferrin Receptor	TFRC	TFRC	Novus Biologicals	22500002	1140	Rabbit	Valid	1:15000	-20
275	Transglutaminase II	Transglutaminase	TGM2	Lab Vision	MS-224-P1	908	Mouse	Valid	1:150	4
276	TRIM25	TRIM25	TRIM25	Abcam	ab167154	1756	Rabbit	Use with Caution	1:2000	-20
277	TSC1/Hamartin	TSC1	TSC1	CST	4906	1125	Rabbit	Use with Caution	1:200	-20
278	TSC2/Tuberin (phospho T1462)	Tuberin_pT1462	TSC2	CST	3617	671	Rabbit	Valid	1:30	-20
279	TTF1	TTF1	NKX2-1	Abcam	ab76013	1081	Rabbit	Valid	1:30	-20
280	Tuberin	Tuberin	TSC2	Abcam	ab32554	670	Rabbit	Valid	1:2500	-20
281	TUFM	TUFM	TUFM	Abcam	ab173300	1842	Rabbit	Valid	1:300	-20
282	Twist	TWIST	TWIST2	Santa Cruz	sc-81417	1353	Mouse	Use with Caution	1:30	4
283	Tyro3	Tyro3	TYRO3	CST	5585	1080	Rabbit	Valid	1:30	-20
284	UBAC1	UBAC1	UBAC1	Sigma-Aldrich	HPA005651	1270	Rabbit	Valid	1:250	-20
285	Ubiquityl Histone H2B	Ubq-Histone-H2B	H2BFM	Millipore	05-1312	1604	Mouse	Use with Caution	1:500	4
286	UGT1A	UGT1A	UGT1A1	Santa Cruz	sc-271268	1267	Mouse	Valid	1:75	4
287	ULK1 (phospho S757)	ULK1_pS757	ULK1	CST	6888	1626	Rabbit	Use with Caution	1:1000	-20
288	VASP	VASP	VASP	CST	3112	678	Rabbit	Valid	1:250	-20
289	VDAC1/Porin	Porin	VDAC1	Abcam	ab14734	1254	Mouse	Valid	1:300	-20
290	VEGF Receptor 2	VEGFR-2	KDR	CST	2479	688	Rabbit	Valid	1:12000	-20
291	VHL/EPPK1**	VHL-EPPK1	EPPK1	BD Biosciences	556347	693	Mouse	Under Evaluation (Targets EPPK1)	1:1000	4
292	Vimentin	Vimentin	VIM	Dako	M0725	1393	Mouse	Use with Caution	1:250	4
293	Wee1	Wee1	WEE1	CST	4936	1802	Rabbit	Use with Caution	1:2000	-20
294	Wee1 (Phospho S642)	Wee1_pS642	WEE1	CST	4910	2058	Rabbit	Use with Caution	1:75	-20
295	WIPI1	WIPI1	WIPI1	CST	12124	1627	Rabbit	Use with Caution	1:1000	-20
296	WIPI2	WIPI2	WIPI2	CST	8567	1628	Rabbit	Use with Caution	1:1000	-20
297	XBP1	XBP1	XBP1	Santa Cruz	sc-32136	1044	Goat	Use with Caution	1:200	4
298	XPA	XPA	XPA	Santa Cruz	sc-56813	1359	Mouse	Valid	1:75	4
299	XPF	XPF	XPF	Abcam	ab73720	2015	Rabbit	Use with Caution	1:100	-20
300	XRCC1	XRCC1	XRCC1	CST	2735	906	Rabbit	Use with Caution	1:30	-20
301	YAP	YAP	YAP1	Santa Cruz	sc-15407	780	Rabbit	Under Evaluation	1:200	4
302	YAP (phospho S127)	YAP_pS127	YAP1	CST	4911	782	Rabbit	Under Evaluation	1:750	-20
303	YB1 (phospho S102)	YB1_pS102	YBX1	CST	2900	835	Rabbit	Valid	1:50	-20
304	ZAP-70	ZAP-70	ZAP70	CST	2705	1828	Rabbit	Use with Caution	1:1000	-20
