# Supplementary figures and images for: PI3K-driven HER2 expression is a potential therapeutic target in colorectal cancer stem cells
Source: Gut. 2021 Jan 12;71(1):119–28. doi: 10.1136/gutjnl-2020-323553 (PMC8666826; doi:10.1136/gutjnl-2020-323553)

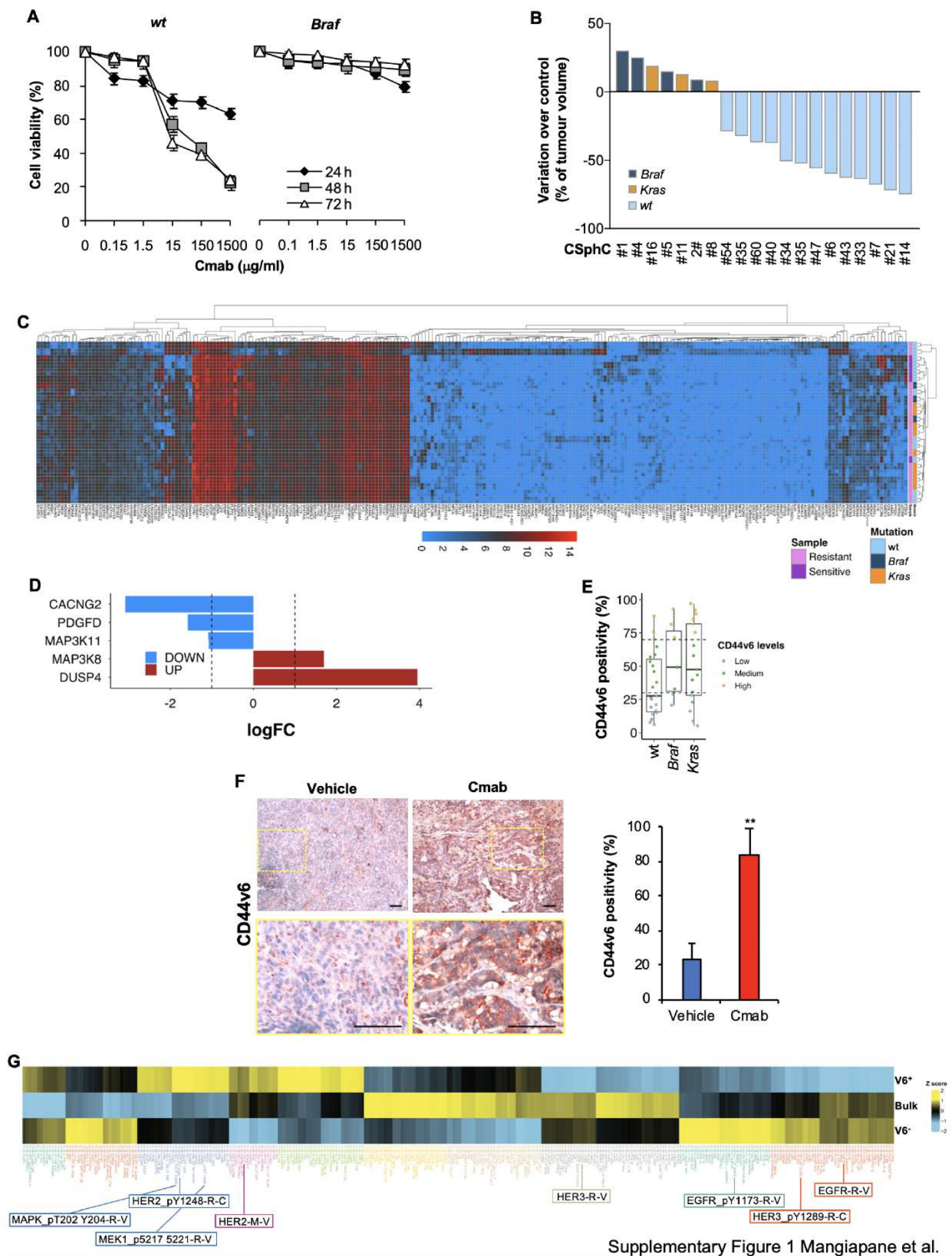

Supplementary Figure 1 Mangiapane et al.

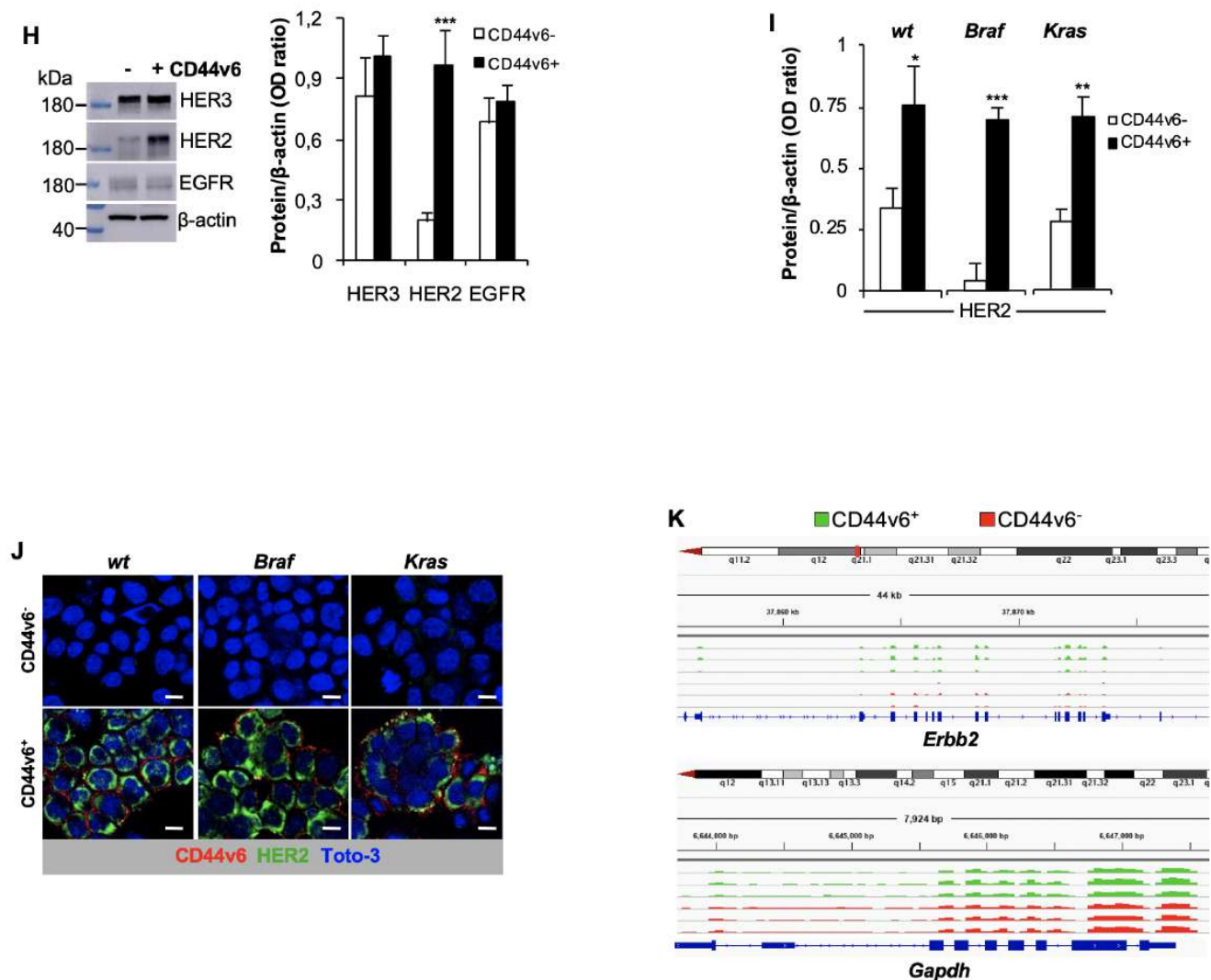

Supplementary Figure 1 Mangiapane et al.

Supplement: Supplementary data [file gutjnl-2020-323553supp002.pdf]

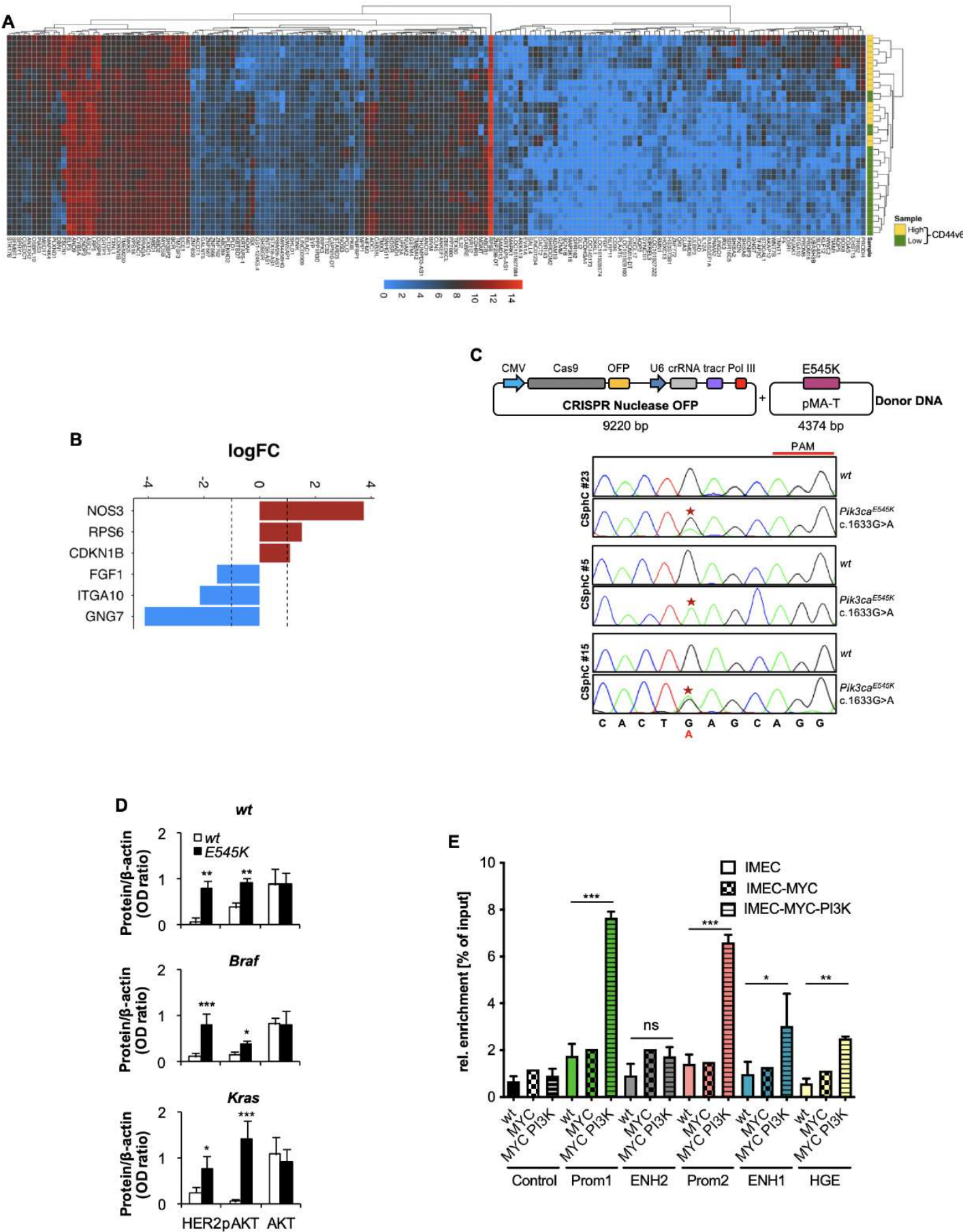

Supplementary Figure 2 Mangiapane et al.

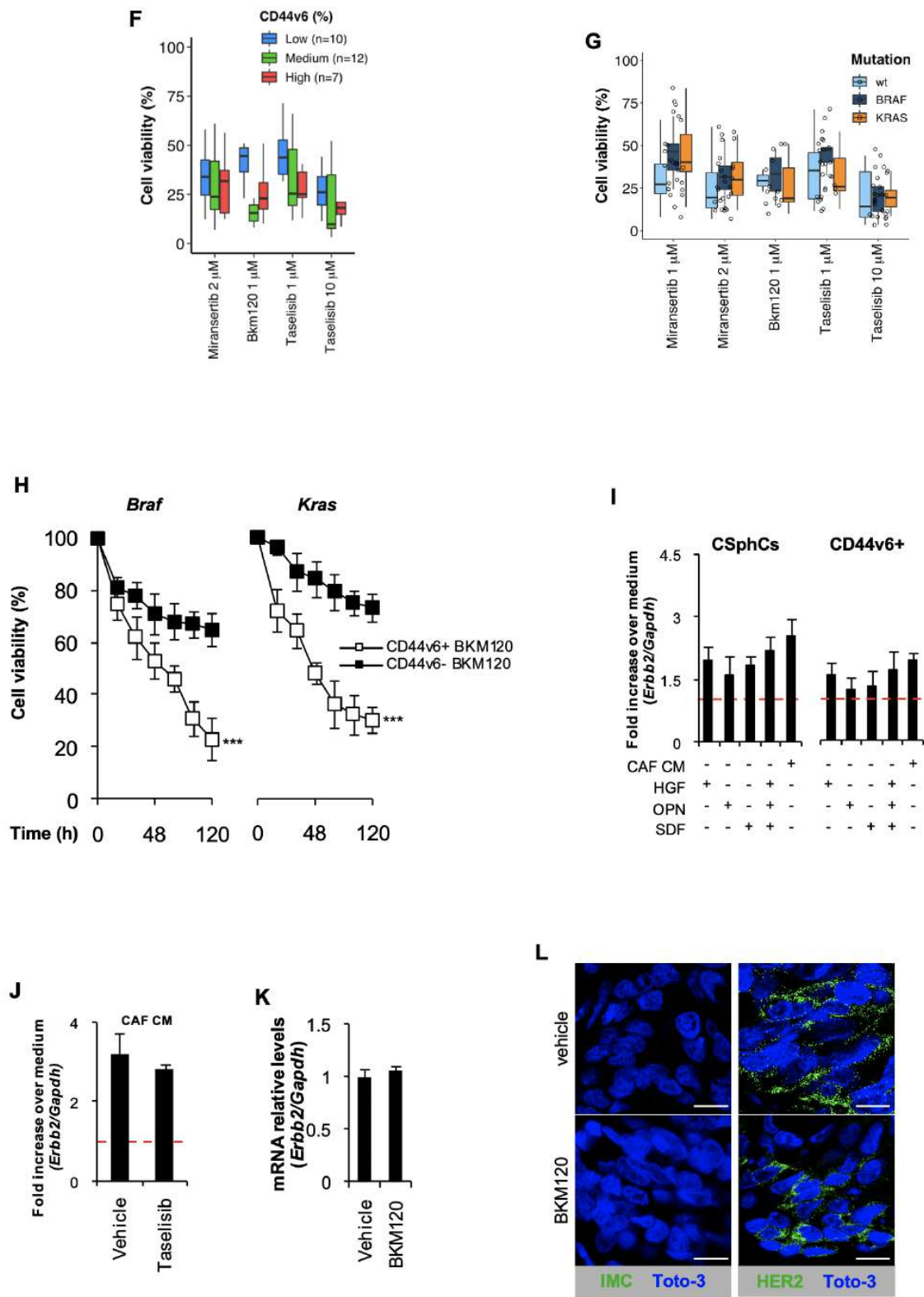

Supplementary Figure 2 Mangiapane et al.

Supplement: Supplementary data [file gutjnl-2020-323553supp005.pdf]

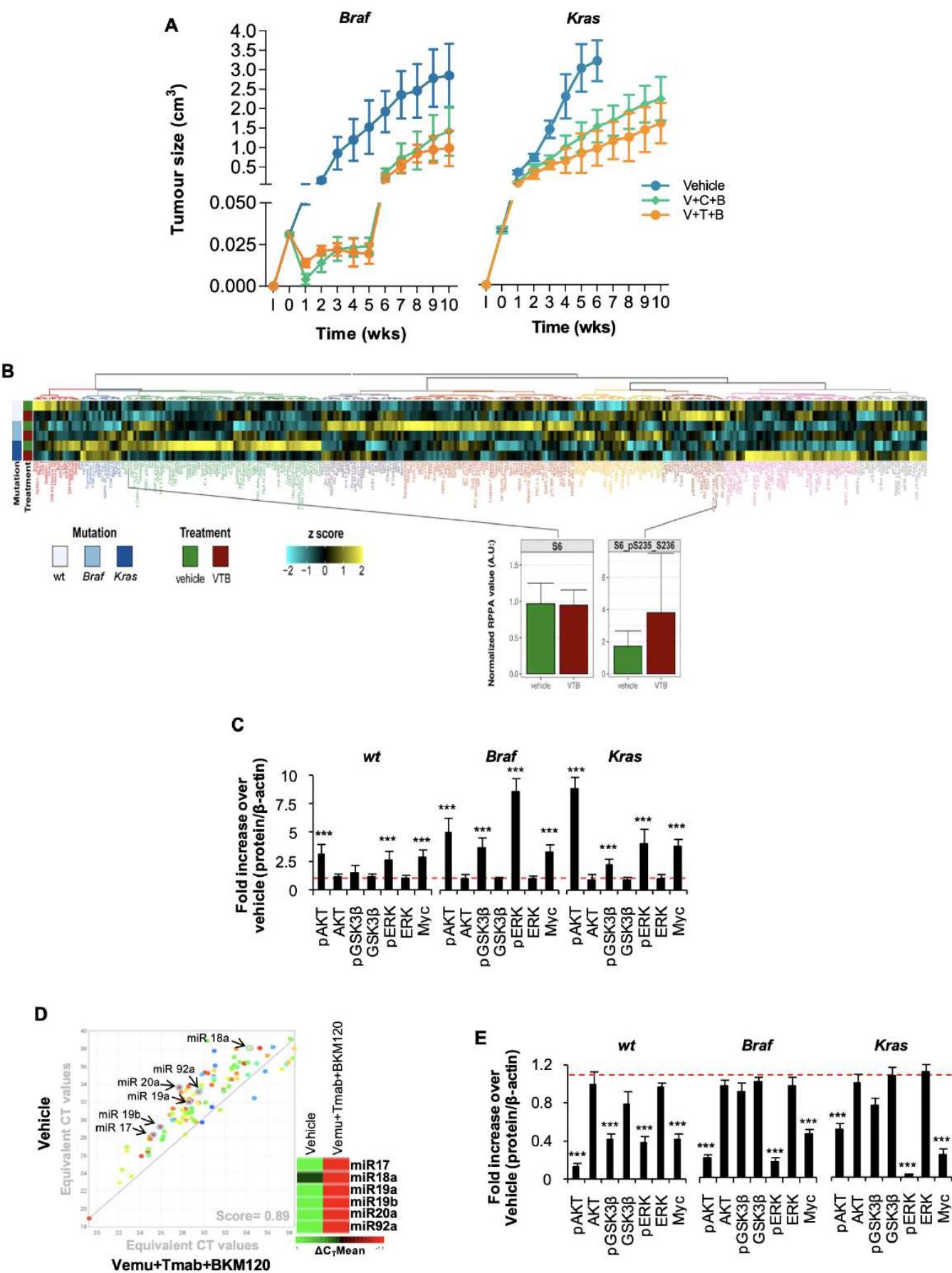

Supplementary Figure 3 Mangiapane et al.

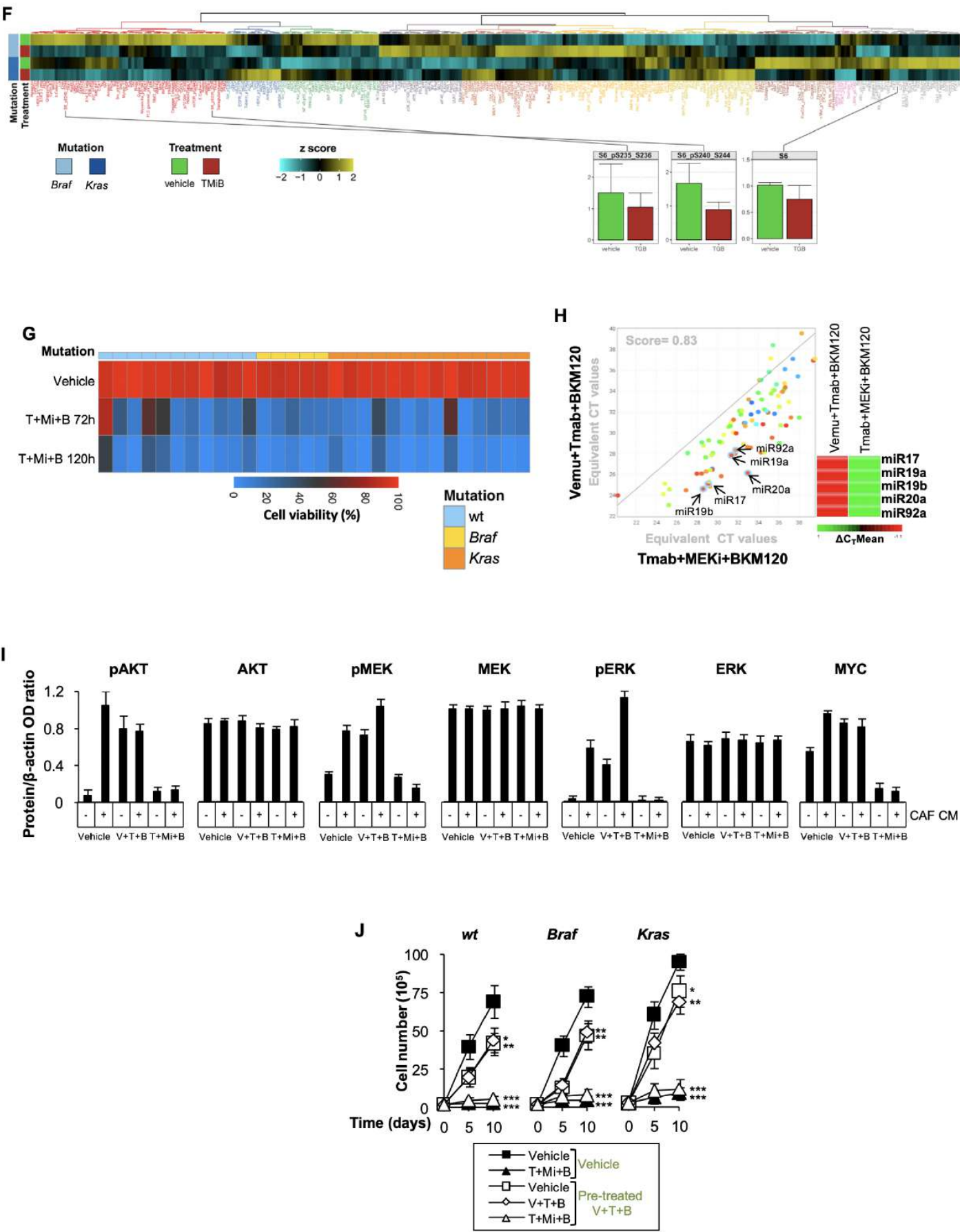

Supplementary Figure 3 Mangiapane et al.

Supplement: Supplementary data [file gutjnl-2020-323553supp007.pdf]

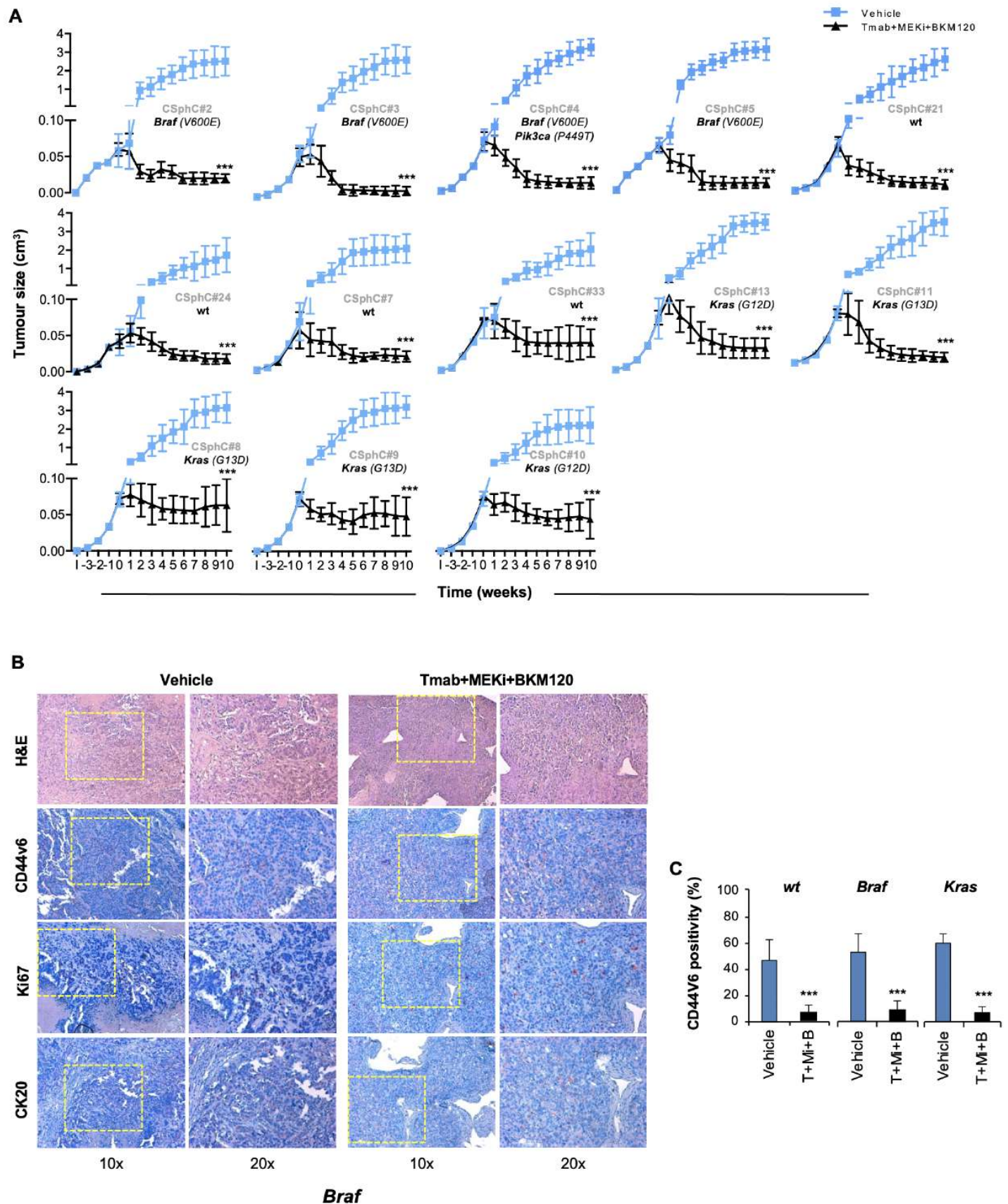

Supplementary Figure 4 Mangiapane et al.

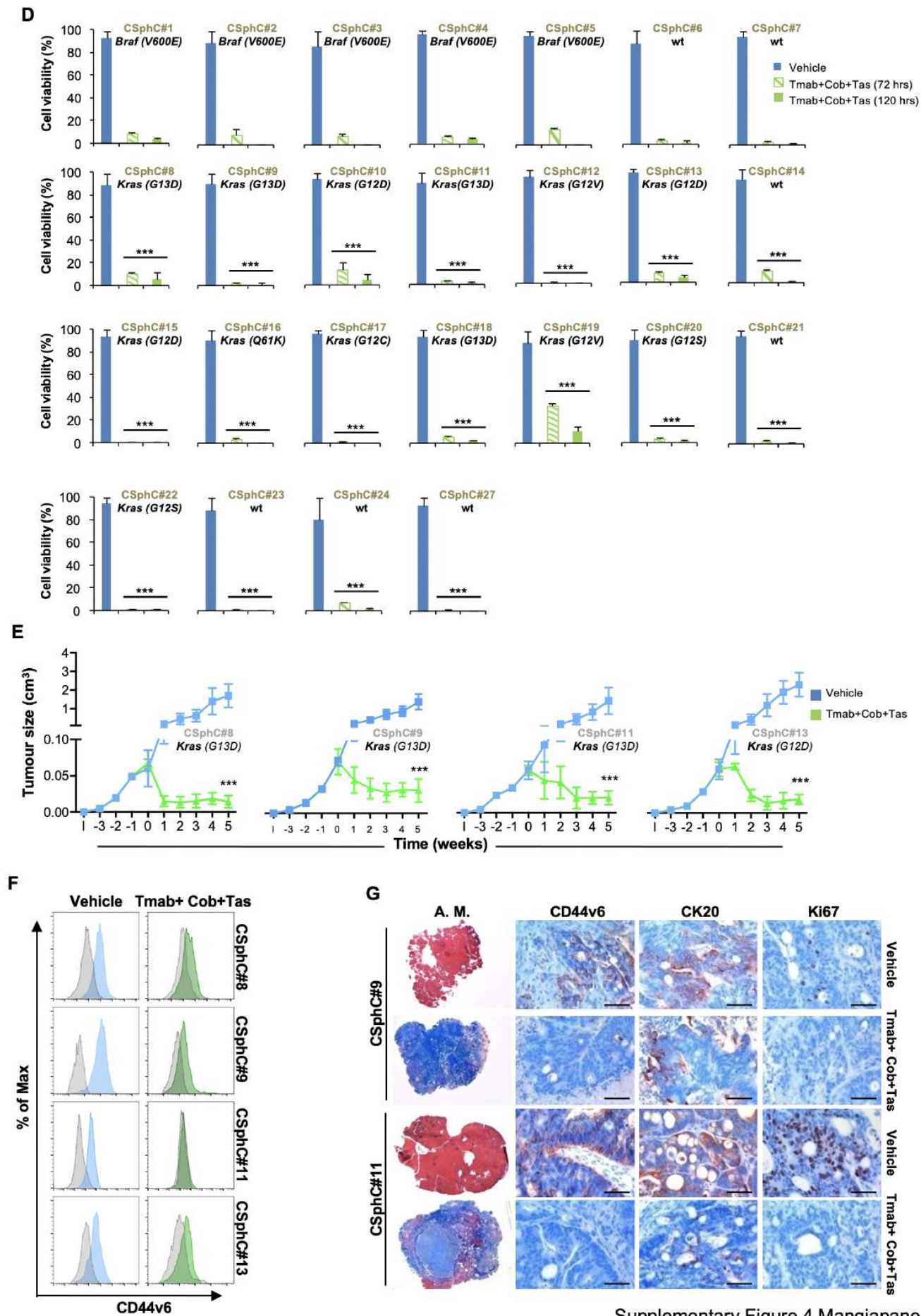

Supplementary Figure 4 Mangiapane et al.

Supplement: Supplementary data [file gutjnl-2020-323553supp008.pdf]
