# Supplementary material for: PI3K-driven HER2 expression is a potential therapeutic target in colorectal cancer stem cells
Source: Gut. 2021 Jan 12;71(1):119–28. doi: 10.1136/gutjnl-2020-323553 (PMC8666826; doi:10.1136/gutjnl-2020-323553)
Supplement: Supplementary data [file gutjnl-2020-323553supp003.pdf]

Supplementary Table 1. Cancer sphere cells and their mutational profiles, ERBB2 status and CD44v6 expression.

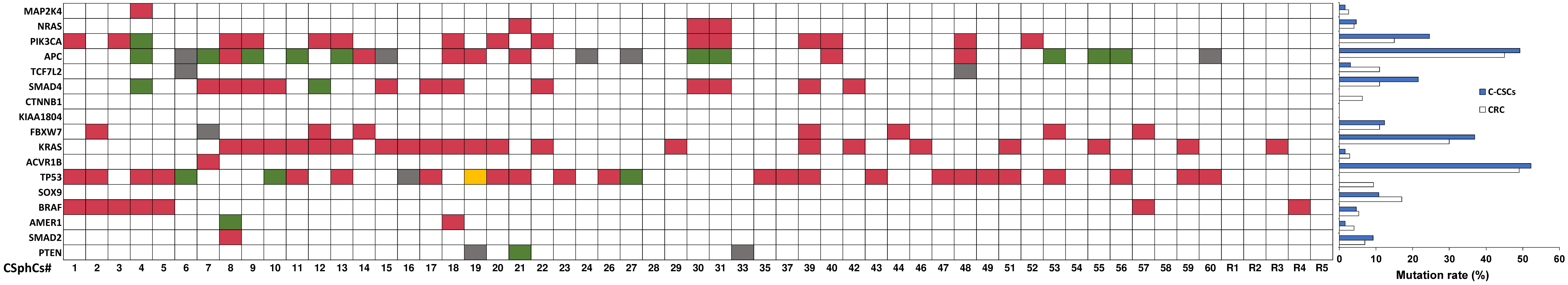

CSphCs#	1	2	3	4	5	6	7	8	9	10	11	12	13	14	15	16	17	18	19	20	21	22	23	24	26	27	28	29
ERBB2 status	0.7-1.25	0.7-1.25	0.7-1.25	0.7-1.25	0.7-1.25	0.7-1.25	0.7-1.25	> 1.25	0.7-1.25	0.7-1.25	0.7-1.25	0.7-1.25	0.7-1.25	0.7-1.25	0.7-1.25	0.7-1.25	0.7-1.25	> 1.25	0.7-1.25	0.7-1.25	0.7-1.25	0.7-1.25	0.7-1.25	0.7-1.25	NA	0.7-1.25	0.7-1.25	0.7-1.25
CD44V6 %	Low	Medium	Medium	High	Low	Medium	Medium	High	High	High	Low	Medium	High	Low	Low	Medium	High	Medium	Low	Medium	High	Medium	Low	Low	Low	Medium	NA	Low
Age	68	41	85	59	68	76	62	57	53	69	77	76	63	47	69	79	75	57	63	68	60	71	78	51	58	60	60	66
Gender	♂	♂	♀	♀	♂	♂	♂	♀	♀	♂	♀	♂	♂	♀	♂	♀	♂	♀	♀	♂	♀	♀	♂	♀	♀	♂	♂	♀
Site	S	L	R	R	R	R	R	R	S	L	R	R	R	R	L	R	L	L	S	R	R	M	M	R	S	L	L	R
Grade	G3	G3	G3	G2	G3	G2	G2	G3	G2	G3	G2	G2	G3	G2	G2	G2	G2	G3	G2	G2	G2	G2	G2	G2	G2	G3	G2	G2
TNM	T2N1M0	T3N0M0	T3N2M0	T2N0M0	T4N1M0	T2N1M0	T3N1M0	T3N2M1	T2N1M0	T4N3M1	T3N1M0	T3N2M0	T4N2M0	T3N0M0	T3N1M1A	T3N0M0	T2N0M0	T3N2M2	T4N2M1	T3N1M0	T3N1Mx	T3N0M0	T3N0M0	T3N0M0	T2N0MX	T3N2M0	T1N0M0	T3N0M0
Stage	IIIA	IIA	IIIC	IIA	IIIB	IIIA	IIIB	IV	IIIA	IV	IIIB	IIIC	IIIC	IIA	IVA	IIA	I	IV	IVa	IIIB	IIIB	IIA	IIA	IIA	I	IIIC	I	IIA

CSphCs#	30	31	33	35	37	39	40	42	43	44	46	47	48	49	51	52	53	54	55	56	57	58	59	60	R1	R2	R3	R4	R5
ERBB2 status	0.7-1.25	0.7-1.25	0.7-1.25	0.7-1.25	0.7-1.25	NA	NA	NA	NA	> 1.25	NA	NA	NA	0.7-1.25	NA	> 1.25	NA	0.7-1.25	0.7-1.25	0.7-1.25	0.7-1.25	0.7-1.25	0.7-1.25	NA	NA	NA	NA	NA	NA
CD44V6 %	Low	Low	High	Low	Low	NA	Low	NA	NA	Low	NA	NA	NA	Medium	NA	Medium	NA	Low	Medium	High	High	Medium	High	NA	Low	Low	Low	High	NA
Age	69	67	58	87	82		49			65			73	78		57	74	66	63	52	79	39	78	61	65	61	62	38	54
Gender	♀	♂	♂	♀	♂		♂			♂			♀	♀		♂	♂	♀	♀	♀	♂	♀	♂	♀	♂	♀	♀	♂	♀
Site	R	R	R	R	R		S			S			R	S		R	S	S	R	S	L	L	M	L	S	RT	RT	RT	L
Grade	G2	G2	G2	G3	G3		G2			G3			G2	G2		G3	G2	G2	G2	G3	G2	G2	G3	G2					
TNM	T2N1M0	T3N1M0	T2N0M0	T4N3M1	T3N2M0		T2N1M0			T4N2M0			T3N0M0	T2N1M0		T4N2M0	T3N0Mx	T2N1M0	T2N1M0	T3N2bMx	T3bN2aMx	T3N2bM0	T3N1M0	T3N2bM0	T3N0M0	T3N1M1	T3N1M0	T4N1M1	T3N0M1
Stage	IIIA	IIIB	I	IVB	IIIC		IIIA			IIIC			IIA	IIIA		IIIC	IIA	IIIA	IIIA	IIIC	IIIC	IIIC	IIIB	IIIC	IIA	IV	IIIB	IV	IV

CD44v6: High >70%; Medium 30-70%; Low <30%; *NA*

***ERBB2*/*RPP30* ratio**: 0.7-1.25 9.7%; > 1.25; *NA*

Site: Sigma S; Left L; Right R; Meso M; Rectum RT; *NA*

Gender: Male; Female; *NA*

Missense SNV; Frameshift Del; Frameshift Ins; Multiple mutation

COSMIC-reported mutation of the analyzed genes (*upper panel*). Mutation types (missense SNV, frameshift deletion or insertion ,and multiple mutations) are indicated. Percentage of mutation rate in CRC patients reported in TGCA database (white bars) and in C-CSCs (blue bars) are indicated on the right. ERBB2 status and CD44v6 expression of CSphCs and clinical data related to CRC patients are reported (*lower panel*).
