# Supplementary material for: PI3K-driven HER2 expression is a potential therapeutic target in colorectal cancer stem cells
Source: Gut. 2021 Jan 12;71(1):119–28. doi: 10.1136/gutjnl-2020-323553 (PMC8666826; doi:10.1136/gutjnl-2020-323553)
Supplement: Supplementary data [file gutjnl-2020-323553supp004.pdf]

Supplementary Table 2. Statistically differentially expressed genes in cetuximab resistant versus sensitive cancer sphere cells.

Gene	Resistant WT	Resistant WT	Resistant KRAS	Resistant KRAS	Resistant KRAS	Resistant KRAS	Resistant BRAF	Resistant WT	Resistant WT	Resistant KRAS	Sensitive WT	Resistant WT	Resistant BRAF	Resistant KRAS	Resistant BRAF	Sensitive WT	Sensitive WT	Resistant BRAF	Sensitive WT	Resistant KRAS	Resistant WT	Sensitive WT	Sensitive WT	Resistant WT
NPY1R	0	0	0	0	0	0	0	0	0	0	0	0	2.254475	0	0	0	0	0	0	0	3.25138	0	0.588633	2.994073
ADAM23	0	0	0	0	0	0	0	0	0	0	0	0	2.254475	0	0	0	0	0	0	0	2.885979	0.902111	0	4.807247
FOXD3-AS1	0	1.518444	0	0.858862	2.062176	0.855986	0	0	0	1.600378	0	0	0	0	0	0	0	0	0	0	1.646481	0	0	4.807247
ADORA1	0.995829	0	2.32209	0	1.182986	2.336277	1.096298	0	0	1.600378	6.6268	0	3.094778	0	0	3.032428	6.729631	0.701667	0	1.562827	0	5.293779	6.856378	1.455124
S1PR3	0	0	0	0	0	0.855986	1.096298	0	0	0	0	1.505082	0	0	0	0	0.697815	0.701667	0	0	3.404373	0	0.588633	1.853078
HOXA3	6.530895	6.204139	3.700626	6.512706	7.80304	9.354616	6.814156	6.424652	6.859617	7.644997	6.817619	7.966386	7.932324	6.923004	7.612989	8.477765	7.02664	7.953003	6.870105	8.502034	1.646481	8.624121	6.445485	3.279323
ADD2	0	2.998749	1.585097	0.858862	0	0	0	1.498706	0	0	2.266443	2.225526	2.254475	0	0	0	0	0	0	0	4.173569	0	1.005502	6.125178
NIPAL4	0	0	1.585097	0	0	0	0	0	0	0	0	0	0	0	0	1.483087	0.697815	0	1.042062	0	5.258786	0.902111	0	4.33341
ARHGAP18	8.580383	7.856558	8.484017	7.758928	8.223633	6.742942	5.674527	7.8058	7.643805	7.05631	8.539049	8.486112	7.154162	8.200954	7.78592	9.883431	8.396197	7.311508	7.779511	7.434464	7.457107	8.367521	8.227692	4.452965
OR2W3	0	0	0	0	4.021973	0	0	0	0	2.340407	0	1.505082	0	0	0	0	0	0	0	0	3.542686	0	0	2.827067
KC6	0	0	0	0	0	0	0	0	0	0	0	0	0	0	0	3.941454	3.279269	0	0	1.562827	0	4.129873	0	
RAB39A	0	0	0	0	0	0	0	0	0	0	0	1.505082	0	0	0	0	0	0	0	0	1.646481	0	0	5.163338
TRIM58	0	0	2.585131	0	4.976873	4.463354	3.490888	0	0	0	0	3.349371	0	0	0	0	0	4.927897	0.613139	0	5.674439	0	0	5.826234
CYP4F8	0.995829	1.262201	1.000101	0	1.182986	5.096339	0	1.498706	1.275828	3.72174	1.182979	0.940508	0	0	0	2.456453	0	0	5.080411	3.140335	9.598994	1.452909	3.009615	4.93596
FOXQ1	5.63567	8.847614	6.820379	7.703694	7.320195	10.16281	3.630002	7.345106	6.454571	9.40457	9.400046	7.773807	9.862174	6.570842	8.464087	11.00808	8.045079	5.744596	6.789519	9.647948	4.408818	8.794698	8.726919	3.721657
APBA2	0.995829	0	0	0	0	0.855986	0	0	0	0	1.182979	2.894221	0	0	0	0	0	0	0	0	0	0	0	8.308961
RIPPLY2	0	0	0	3.631162	0	0	0	0	0	0	0	0.940508	0	0	0	0	0	0	0	1.562827	3.404373	0	0	3.62313
HOXA2	4.162037	2.501323	1.000101	3.722914	4.498115	5.430278	5.706321	4.046148	3.927307	4.97652	4.326017	5.71357	6.602527	4.615666	6.861729	6.056029	4.7616	5.014317	3.831837	5.897958	0	6.39925	4.139641	1.455124
CACNG2	0	0	0	0	0	0	0	0	0	0	0	0	0	0	0	0	0	0	0	0	5.922658	0	0	
NUDT16	8.782828	8.876522	8.515901	8.029862	9.233449	8.417887	9.333464	9.128568	8.734682	8.713004	7.60072	8.474974	8.51903	8.00513	9.004572	7.459904	8.010695	8.91682	9.36936	8.677555	9.457509	8.340708	8.508805	9.766577
NKX2-1-AS	0	0.552086	0	0	0	0	0	0	0	0	0	0	0	0	0	0	0	0	0	0	0	0	0	5.803917
IFNGR1	8.3404	8.963973	8.661979	8.995252	8.914401	9.231718	7.778118	9.197489	7.922639	8.157406	10.20765	8.34642	8.129792	8.145651	8.986959	8.262689	8.43561	8.51147	8.030973	7.498232	7.645439	9.451999	9.318167	6.889614
PERP	10.81502	10.69188	10.6902	11.43679	10.52189	11.92337	10.0303	9.992845	10.43463	10.72088	11.52884	10.48956	10.14287	11.25335	11.06256	11.14019	10.46586	10.13215	10.04719	10.59696	10.84365	11.50839	10.88173	5.953335
PTPRM	0	0	0	1.393533	0	2.550894	2.742064	1.498706	0	0	6.896659	0	1.528973	0	2.854499	5.680126	6.064729	4.632417	0	0	0	4.560027	5.580608	0
SLC66A1L	0	0	1.000101	0	0	0	0	0	0	0	0	0.940508	0	0	0	0	0	0	0	0	1.646481	0	0	0
UNC5D	0	0	0	0	0	0	0	0	0	0	0	0	0	0	0	0	0	0	0	0	3.892341	1.452909	0	
INA	1.579399	0.552086	0	0	0	0	0	0	0	0	0	0	0	0	0	0	0	0	0	0	0	0	0	7.132824
IRX6	0	0.552086	0	0	0	0	0	0	0	0	0	0	0	0	0	0	0	0	0	0	0	0	0	2.994073
FLJ40288	0	0	0	0	0	0	0	0	0	0	0	0	0	0	0	0	0	0	0	0	3.404373	0	0	0
NKX2-8	0	1.924946	0	0	0	0	0	0	0	0	0	0	0	0	0	0	0	0	0	0	0	0	0	7.030823
NPR1	0	2.912049	1.585097	1.393533	3.108153	0	1.096298	0	0	0	1.182979	0.940508	0	0	0	0	0	0	0.613139	0	1.046383	0	0	6.813463
YTHDC2	8.546257	7.83904	7.644057	7.163712	8.710745	8.000837	8.685931	6.345879	8.325575	8.573747	8.037343	7.508454	8.24188	8.426518	7.376446	7.810798	8.419973	9.065904	8.039709	8.302163	0	8.124201	8.33513	8.123176
PRR16	0	1.735965	0	0	0	0	0	0	0	0	0	0	0	0	0	0.924276	0	0	0	0	0	0	0	7.333116
CREBL2	7.553933	7.978906	7.467806	7.555582	7.572124	6.927059	6.432191	7.261736	7.271545	7.849509	6.385074	7.456549	7.383278	7.674872	7.043115	6.788206	5.863646	7.608652	7.737168	7.674189	7.997843	7.306084	6.625581	8.231652
PRIMA1	0	0	0	0	0.709495	0	0	0	0	0	0	0	0	0	0	0	0	0	0	0	0	0	0	3.517377
CNNM1	0	0	0	0	0	0	0	1.902589	0	0	0	0	1.528973	0	0	0	0	0	0	0	0	0	0.588633	7.621704
SIX3-AS1	0	1.518444	0	0	0.709495	0	0	0	0	0	0	0	0	0	0	0	0	0	0	0	1.046383	0	0	2.164658
APBB2	8.948765	8.368972	8.661979	6.781665	7.878973	7.319755	7.283322	7.829155	8.38812	9.195449	9.728585	7.878952	8.149089	8.802275	8.807946	9.492019	9.246728	7.971123	8.170098	9.129118	6.705128	8.146489	9.603426	7.317447
RAB8B	6.036167	4.73559	5.930936	4.35942	6.801291	4.834906	4.76422	5.331155	5.350447	5.307877	6.942084	5.0519	3.094778	3.890746	4.780742	4.756715	6.606103	5.873499	5.620356	5.521974	4.08578	6.028813	5.711212	0.903733
MIR4787	0	0	0	0	0	0	0	0	0	0	0	0	0	0	0	0	0	0	0	0	0	0	0	1.853078
ITGA7	5.969059	5.923011	7.257588	4.46948	7.523144	6.897959	5.181089	5.652459	6.254235	4.97652	4.02196	6.146889	7.09569	5.261129	5.795315	4.311239	4.693868	4.803975	6.711474	5.325042	5.922658	7.008017	4.1803	9.084011
ZYG11A	0	0.552086	0	0	0	0	0	0	0	0	0	0	0	0	0	0	0	0	0	0	0	0	0	5.803917
ANXA3	7.7861	8.177821	8.467807	8.779831	8.061571	8.507311	7.636779	10.31578	7.422614	7.887149	10.78599	7.579506	10.73301	7.908767	8.85725	9.656716	5.93872	8.301241	9.257718	6.932722	5.612958	9.087459	9.93949	0
SCARNA11	0	0	0	0	0	0	0	0	0	0	0	0	0	0	1.35509	0	0	0	0	0	1.046383	0	0	1.455124
C5orf63	6.161688	7.003083	5.672623	5.436546	6.751127	7.370069	5.76788	6.584155	6.280852	7.202813	5.088808	5.738622	5.893845	6.439043	6.41175	6.900874	5.981957	6.386855	5.20991	6.536879	0	6.457459	4.987916	4.976433
PLCXD3	0	0	0	0	0	0	1.711982	0	0	0	0	0	0	0	0	0.924276	0	1.525643	0	0	1.046383	0	0	2.164658
FZD10	0.995829	0	0	4.799555	2.604511	0	7.645005	0	0	0	9.67802	1.505082	3.827882	6.729708	5.428596	10.64373	3.680197	7.561593	0.613139	0	1.046383	8.470052	7.432984	0
LINC00867	0	0	1.000101	0	0	0	0	0.935796	1.275828	0	0	0	0	1.252965	1.35509	0	1.166132	1.171698	0	0	1.046383	0	0	1.455124
PAQR9	0	0	0	0	0	0	0	0	0	0	0	0.940508	0	0	0	0	0	0	0	0	0	0	0	2.420752
NDST3	0	1.262201	1.000101	0	0	0	0	0	0	0	0	0	0	0	0	0	0	0	0.613139	0	0	0	0	1.455124
LAMC2	8.008487	7.98157	9.346715	8.711474	9.326314	8.986545	7.103984	8.204417	7.102787	8.275652	11.50508	7.44137	8.978737	8.564695	7.976473	7.769845	9.274526	8.236798	7.931205	8.265983	7.113008	5.293779	9.168311	1.455124
RNF150	0	0	0	0.858862	0	0	0	0	0	0	0	0	0	0	0	0	0	0.701667	0	0	0	0	0	2.827067
IL17RE	8.664092	5.934054	7.960203	6.670829	8.232801	6.440612	7.196438	8.951003	8.614264	7.982971	7.552717	7.544417	7.522957	7.048915	6.740832	7.489034	7.697101	7.76223	6.636663	8.111446	8.835949	7.668388	7.044323	9.338086
SEPTIN8	10.06446	9.348333	9.833092	9.206684	10.12144	9.35283	9.315539	9.631971	10.09357	10.10119	9.463729	9.45227	10.17617	9.251851	9.736905	9.582641	9.656731	9.09594	9.95629	10.22996	9.091148	9.397286	9.626599	8.15438
HOXA5	0.995829	6.283991	2.32209	6.525504	8.118738	6.2694	7.240534	5.936741	1.275828	4.97652	6.530952	6.201969	8.354222	6.838337	7.893823	8.616222	7.335282	7.956645	5.524021	5.325042	0	5.683423	6.47029	2.164658
OOEP	0	0	0	0	0	0	0	0	0	0	3.636225	0	0	0	0	0	1.519108	0	0	0	0	5.012422	4.625246	
NKX2-1	0	3.713841	0	0	0	0	0	0	0	0	0	0	0	0	0	0	0	0	0	0	0	1.452909	0	9.759347
POLK	6.786138	6.700873	7.098232	6.260504	6.470243	6.964969	6.066242	4.960104	6.199481	6.70934	7.000508	6.355614	7.399482	7.018452	6.302073	6.186234	6.651444	7.022755	6.137002	7.064946	5.733406	6.606391	6.494677	4.509221
BVES-AS1	0	0	0	0	0	0	0	0	0	0	0	0	0	0	0	0	0	0	0.613139	0	0	0	0	2.164658
CHST2	0	0	1.000101	0	0	0	0	0	0	0	0	0.940508	0	0	0	0	0	0	0	0	0	0	0	5.532529
PTGER3	0	0	0	0.858862	0	0	0	0	0	0	0	0.940508	0	0	0	0	0	0	0	0	0	0	0	2.638167
PAK3	0	0	0	0	0.709495	0	0	0	0	0	0	1.505082	0	0	0	0	0	0	0	0	0	0	0	3.517377
PHF21B	0	0	0	0.858862	0	0	0	0	0	0	0	0	0	0	0	0	0	0	0.613139	0	0	0	0	6.032247
IL24	0.995829	1.518444	0	0	0.709495	0	1.096298	1.902589	1.275828	0	1.824117	0	0	0	0	0	1.519108	0.701667	0.613139	0	2.395442	0	2.008245	2.994073
WSCD2	0	0	2.32209	0.858862	1.538883	0	0	0	0	0	0	0	2.734961	0	0	0	0	0	0.613139	0	0	0	0	8.316863
RNF180	0	0	0	0.858862	0	0	0	0	0	0	0	0.940508	0	0	0	0	0	0	0	0	0	0	0	2.827067
LCK	4.7993	6.384019	3.170104	5.68492	4.279598	6.493448	5.737429	6.638046	3.927307	2.340407	11.27444	5.608768	4.673298	4.019381	2.504466	8.068907	9.013116	4.668399	1.640777	3.429162	1.646481	0	10.38275	0
SUOX	8.584125	8.133002	8.033624	7.504785	8.627456	9.377637	8.900397	8.548416	8.700586	9.520882	6.849756	8.540548	7.566665	7.063908	8.191654	8.418434	7.756409	8.795972	9.081365	9.378229	8.920849	7.592489	7.561672	9.921009
POGLUT2	5.799151	5.877974	5.20965	7.555582	5.061638	6.040008	5.608757	6.439903	6.280852	5.953424	5.192692	6.725049	5.644747	5.585938	5.320242	6.131864	5.516876	5.3385	4.664532	6.11689	5.258786	6.9176	5.368268	3.900568
TBC1D16	9.669376	9.853337	10.58422	9.401506	9.826724	10.43933	9.754294	9.643544	9.808108	10.5199	10.46919	10.27087	9.221378	10.43495	10.40409	9.994248	9.660063	10.1086	9.443656	10.31432	9.836052	10.30899	10.56461	7.801414
GOLGA6L7	0	0.552086	0	0	0	0	1.096298	0	0	0	0	0	0	0	0	0	0	0	0	0	0	0	0	1.853078
NOVA1	0	0	0	0	0	0	0	0	0	0	0	0	0	0	0	0	0	0	0	0	0	0	0	5.662243
GASK1A	1.579399	1.262201	0	1.782752	0	0	1.711982	0.935796	1.275828	3.928426	1.182979	4.206711	0	0	0	0	0	0	0	0	5.133031	0.902111	2.709739	3.900568
ALDH1A1	8.898554	10.98555	10.88666	13.71688	9.765919	9.589643	9.143318	12.62976	9.033607	7.923833	5.033919	10.94864	8.008256	6.906464	11.26976	14.63456	10.23292	9.301819	11.3844	8.209953	5.996736	12.12385	5.385759	3.62313
NKX6-1	0	0.552086	0	0	0	0	0	0	0	0	0	0.940508	0	0	0	0	0	0	0	0	0	0	0	2.827067
GRID1	0	0	0	0	0	0	0	0	0	0	0	0	0	0	0	0.924276	0	0	0	0	0	1.452909	0	2.638167
FCMR	2.315249	2.615475	2.32209	2.556218	1.824127	0.855986	3.981368	2.885695	3.252214	2.827144	1.824117	1.909816	2.734961	0	1.35509	0	3.74856	2.730629	2.234835	2.778832	5.644026	0	3.710581	4.761658
PRR19	3.314412	4.296324	4.248119	4.620269	3.708121	3.308878	4.934959	5.999847	3.252214	4.269784	2.266443	3.886361	4.442534	3.592931	5.529378	3.941454	3.876272	2.860731	4.664532	0	5.088553	3.514195	4.054714	8.880459
MAP3K11	9.766443	9.670644	10.09296	9.917001	10.39219	10.16281	9.618857	9.933472	10.13063	9.387161	8.902946	9.804883	10.08142	9.511759	9.680495	9.719512	9.588431	9.072633	10.16833	9.480442	10.01604	9.573774	8.760827	10.4616
SCN4A	0.995829	0	1.000101	0	1.824127	2.084058	0	0	0	1.600378	1.824117	1.505082	0	0	0	0	0	3.617639	0	1.562827	1.046383	0	1.815209	7.059364
RPL39L	7.171613	0.552086	0	6.693688	1.182986	0	5.351624	0	6.671263	0	0	5.307667	0	0	0	0	0	5.584323	1.640777	0	6.465294	0	0.588633	6.51686
ARHGEF38	6.733192	5.669137	6.044593	4.667222	6.222782	6.299384	4.702501	4.628574	5.191185	5.804005	7.24967	5.763247	4.673298	6.368335	6.213973	5.753983	7.512838	5.810487	5.927019	6.270876	4.895512	5.261467	6.542246	2.827067
SLC39A4	8.267798	9.534885	9.197418	10.38735	8.677557	10.20896	10.10525	8.669809	8.758552	9.210374	9.330577	9.207329	10.34046	8.76147	9.978869	7.054919	7.067357	9.620423	9.920472	8.770875	9.394962	6.471652	9.168311	10.94278
KCP	3.314412	6.029811	5.04459	2.341426	3.964428	3.527314	4.570528	5.229504	4.056163	1.600378	4.02196	1.505082	3.827882	1.252965	1.35509	2.675001	0	4.26041	3.160882	2.295325	4.408818	0	1.815209	7.011477
HBQ1	0	0	2.32209	0	0	0.855986	1.096298	0.935796	0	0	0	3.21301	0	1.252965	0	1.483087	0	0.701667	3.243874	0	3.25138	4.505888	0	6.503074
CAVIN1	7.28632	6.477559	5.357749	7.219908	7.99128	8.058958	7.628506	5.67841	7.752401	6.652187	9.666789	6.566188	9.600033	5.095899	4.511837	7.8839	6.408987	7.493019	7.148419	5.521974	5.176179	9.075893	8.700955	3.900568
OLFM1	0.995829	0	0	0.858862	0	0	0	0	0	1.600378	1.824117	0	0	0	1.35509	0	0	0	4.723561	0	0	0	0.588633	7.945993
PPFIA3	8.036072	8.566404	8.614911	8.752875	7.863316	8.5201	7.127659	9.973324	8.511249	8.26616	7.851447	8.815353	8.719505	7.093435	7.865186	7.963888	6.078074	6.606783	8.30183	8.328714	9.768933	9.326818	8.466279	9.766577
STAMBPL1	7.621045	7.610341	7.248128	7.791081	8.108813	7.536667	6.814156	8.107461	6.945309	6.131254	8.606435	6.966852	7.034748	7.604836	7.78592	7.422645	7.813374	6.922026	7.782984	6.409999	7.376156	6.938188	8.490733	4.059719
NXPE1	0	1.735965	0	0	0	0	0	0	0	0	0	0.940508	0	0	0	0	0	0	1.867074	0	7.606537	0	0	0
TCERG1L	0	0	0	0	0	0	0	0	0	0	3.108141	0	0	0	0	0	0.697815	0	0	0	0	0	0	4.452965
ERCC6L2	6.82461	6.882855	7.570056	7.053158	6.480543	6.675949	6.541984	5.915077	6.523063	7.120927	7.097382	6.86814	6.485954	7.258932	6.84227	7.445116	6.642489	6.739561	6.856983	7.186063	5.581206	6.717755	6.947532	6.01292
CD6	2.315249	2.377357	2.585131	2.556218	2.445361	3.051365	3.336916	0	0	1.600378	2.878187	2.225526	0	2.707797	0	0	1.519108	4.21247	1.372257	1.562827	2.885979	0	2.816774	5.505126
B4GALT1	8.977505	9.158949	10.68583	8.99755	8.324277	9.77445	8.01869	9.080721	8.528012	8.926896	9.660008	8.791609	9.309771	8.756864	8.866912	9.342435	8.956436	9.099239	9.720361	9.295396	8.334203	9.424902	8.959328	6.544044
SMOC1	1.993739	0	6.066288	9.926681	3.964428	3.051365	2.742064	0	1.942141	0	10.19676	6.810233	0	3.592931	7.008616	10.15963	8.486539	1.171698	0	1.562827	3.542686	6.414024	10.54138	0
SLC2A1	9.493495	10.82724	12.61698	11.93819	10.71417	11.74217	9.975904	11.04171	9.189244	9.40889	13.74379	10.76955	11.34095	10.46773	10.52954	11.87265	10.04862	10.16558	9.431536	9.503932	9.173307	12.21446	10.65573	9.397045
SMOX	8.963206	7.029065	6.129482	7.898288	6.121119	7.896809	7.04304	7.770043	9.22395	8.518853	6.530952	6.77434	7.28203	6.987333	7.455415	7.474542	7.183036	8.08865	7.185633	8.991872	2.661459	7.874715	4.868587	5.826234
STAC2	0	0	1.585097	0	0	0	0	0	0	0	0	0	0	0	0	0	0	0	0	0	1.046383	0	0	2.827067
CDK6	9.868172	9.466178	9.224203	10.46912	8.779094	9.264388	8.653679	9.490827	9.091138	8.87788	10.02092	9.65832	8.511594	8.221158	9.242114	9.672673	9.433525	9.673399	9.741936	9.259044	10.30192	9.031074	9.465267	3.143737
MVB12B	7.90457	8.05422	7.077016	7.340688	7.314448	7.911444	7.967132	7.377151	8.112425	8.157406	7.149951	7.699197	7.034748	7.063908	7.566312	8.702691	8.377429	8.332415	6.983124	8.209953	7.250573	9.028676	7.621952	6.070141
CHSY3	0	0	1.000101	0	0	2.084058	0	0	0	0	0	0	0	0	0	0	0	0	0	0	0	0	0	2.164658
DOCK3	3.899095	2.092015	4.000189	1.393533	4.855501	4.034737	2.742064	5.157526	3.452812	4.666002	2.878187	4.343641	4.007772	0	3.751148	1.884869	3.18374	3.617639	2.654399	2.778832	4.173569	4.266393	3.009615	7.829304
WBP11P1	0	0.552086	0	0	1.182986	0	0	0	0	0	0	0	0	0	1.252965	0	0	0	0.701667	0	1.646481	0	0	0.903733
PRSS1	0.995829	0	2.32209	0.858862	0	3.18586	4.702501	6.880894	1.275828	0	6.530952	1.505082	3.094778	6.074651	2.854499	4.174478	6.530457	5.777918	0	0	1.046383	5.658825	6.837435	0
LINC02159	0	0.950401	0	0	0	0	0	0	1.275828	0	0	3.21301	0	0	1.35509	0	0	0	0	0	2.395442	0	0	3.403254
FOXN2	7.171613	6.707323	7.807556	6.132506	7.462084	6.519157	6.292554	5.626033	6.749579	6.96539	8.321401	6.9018	6.29127	6.923004	6.48886	7.246208	7.06065	7.259377	7.048071	7.367747	6.629528	7.775237	7.424547	5.662243
EFS	0	0	0	0	0	0	0	0	0	0	0	0	0	0	0	0.924276	0	0	0	0	1.646481	0	0	0
PNMA6A	0	0	0	0	0	0	0	0	0	0	0	1.505082	0	0	0	0	0	0	0	0	0	0	0	3.279323
CLDN18	0	0	0	0	0	0	0	0	0	0	0	0	2.254475	1.252965	0	0	0.697815	0	0	0	0	0.902111	0.588633	8.072734
A4GALT	0	2.241729	2.32209	1.393533	4.32603	4.353316	0	0	0	0	8.741034	0.940508	0	1.913269	0	3.032428	3.279269	0.701667	4.807808	0	0	7.065293	7.506778	3.143737
SIX2	0	1.518444	0	0	0.709495	2.336277	0	0	0	3.480412	1.824117	0	0	0	1.35509	2.198785	0	0	0	3.875805	5.088553	0	1.328528	4.93596
ANTXR1	0	0.950401	0	3.05701	0.709495	0	0	0	0	0	0	1.909816	0	0	0	0	0	0	0	0	1.046383	0	0	8.758112
S100A11	10.92387	11.25139	11.42437	11.88859	11.18989	13.08242	11.54735	12.35646	11.2324	11.81869	11.93208	11.26275	13.80459	11.09905	13.47441	12.59801	11.51065	9.946799	11.00473	11.25497	11.28666	10.99133	11.52026	4.452965
HOXA9	2.578004	8.39518	6.507994	7.517653	8.226695	8.397239	8.092729	0	1.942141	2.827144	8.483802	7.06915	8.511594	8.554115	9.200691	8.91344	9.589596	9.060836	7.765534	3.140335	1.646481	5.887756	7.947576	0
ZFHX3	7.120989	7.212576	6.129482	6.53819	7.973159	7.124217	6.692438	6.210148	6.749579	8.005965	8.274819	7.537296	6.657462	7.271904	6.513674	7.703045	7.522632	8.101867	8.556411	7.741609	6.482585	7.398128	7.643923	4.761658
PGGT1B	6.515299	6.726505	6.741667	5.876911	6.134226	5.632261	5.990795	6.020284	6.280852	5.75055	6.362976	5.902774	5.126991	7.107976	5.911907	6.691274	7.151822	6.588118	6.385692	6.195936	1.646481	5.325383	6.603327	6.01292
PID1	0	4.684193	1.585097	0.858862	1.538883	0	0	0	1.275828	1.600378	4.498102	0	4.673298	1.252965	3.135971	0.924276	0	0	1.867074	1.562827	4.672592	1.452909	4.097802	7.743963
ZNF274	7.811862	8.274389	8.303982	9.018072	8.938884	7.949761	7.793001	6.74014	8.051896	8.926896	4.23161	9.154786	8.28613	7.018452	8.01174	7.652444	1.166132	8.024157	8.304249	8.91218	7.614402	7.329651	1.328528	8.462885
HOXA-AS3	0.995829	2.819803	1.000101	3.631162	5.380411	4.104329	3.630002	3.05373	1.275828	3.928426	3.480731	4.133043	4.31165	4.442918	5.668465	4.43615	4.693868	3.192664	2.654399	3.875805	0	3.276196	3.917144	0.903733
RTN4RL1	0	4.226183	1.585097	0	7.782375	0	0	1.498706	1.275828	0	6.449407	2.225526	0	0	2.041335	0	0	0	3.467553	0	7.891824	0.902111	6.899634	7.011477
FOXL2	0	2.819803	0	0	0	0	0	0	0	0	0	4.468693	0	0	0	0	0	0	0.613139	0	1.646481	0	0	5.163338
RUNX1	8.396812	7.506902	9.137193	8.191164	7.619496	8.913301	8.409985	8.640405	8.859128	7.757664	9.854694	8.568762	9.40113	9.383174	8.709357	10.09952	9.294672	9.077659	8.42698	7.780599	8.825823	7.579442	9.10372	4.714581
EGFL6	0.995829	0	0	0.858862	0	0	0	0	0	0	0	0	0	0	0	0	0	0	1.042062	0	0	0	0	2.420752
EXOC2	8.487524	8.173169	8.055483	7.970263	7.651754	7.370069	7.724786	7.576618	8.112425	8.083672	8.112115	8.452437	7.729348	7.584185	7.416471	7.652444	8.62026	8.085326	7.277138	8.048942	7.09063	7.630938	7.985354	6.900172
LIFR	0	0	0	0	0	0	0	0	0	0	0	0	0	0	0	0.924276	0	0	0	0	0	0	0	3.900568
KIF5C	0.995829	2.241729	0	1.393533	3.964428	0	0	0	1.942141	0	0	5.091333	1.528973	0	0	0.924276	0	2.730629	1.042062	0	3.784938	4.711227	0	8.276913
CXCR4	0.995829	3.812881	3.907079	7.035372	5.486424	4.104329	0	1.498706	1.275828	0	10.22613	4.690333	4.673298	4.909268	8.088072	6.016562	11.859	0	8.400092	1.562827	1.046383	4.266393	9.111626	1.853078
SLIT2	0	0.552086	2.32209	1.782752	0	4.234187	0	0	0	0	0	3.062403	0	0	0	0	0	1.171698	4.723561	0	4.895512	0	0	3.279323
SPATA2L	6.591645	6.944221	8.022569	7.908094	7.40915	8.176714	7.196438	8.530718	7.853392	7.44078	7.213078	7.867641	8.060146	7.383678	7.845774	6.943587	6.956116	6.731079	8.395561	6.978157	7.598629	6.958487	7.576979	8.491095
LINC02561	0.995829	0	0	0.858862	0	2.336277	1.711982	0	0	0	2.604499	1.505082	0	0	0	1.483087	2.722119	1.525643	1.640777	2.295325	0	0.902111	1.005502	0
HPCA	1.579399	2.819803	2.32209	1.393533	1.824127	2.336277	2.968703	1.902589	0	1.600378	0	1.505082	0	0	4.051486	0	2.420704	2.250446	3.160882	2.295325	2.069027	0.902111	2.178475	5.78125
TOP3A	8.546257	7.550607	7.303981	8.317956	8.173726	7.93073	8.844346	8.736174	8.624692	7.730311	8.372069	8.382632	9.05744	7.823235	8.83773	8.224228	9.131449	8.595638	7.921783	7.367747	7.385378	8.219792	8.81024	6.857469
HTRA3	0	0	1.000101	0.858862	0	0	0	0	0	0	0	0.940508	0	0	0	0	0	0	0	0	0	0	0	4.133161
ARMCX1	2.800198	0.552086	0	0	2.604511	0	0	0	0	0	0	0	1.528973	0	0	1.483087	0	0	0	0	1.046383	0	0	6.557446
NMNAT3	7.451125	6.439318	6.170124	6.512706	7.181873	7.020028	7.218655	6.638046	7.323679	7.688285	6.530952	6.725049	5.589316	7.078747	6.782267	6.627209	6.970499	7.452319	7.796793	8.319918	4.672592	7.027363	6.170701	5.662243
GHR	0	0	1.000101	0	0	0	0	0	0	0	0	0.940508	0	0	0	0	0	0	0	0	0	0	0	2.420752
SEC14L5	2.800198	0.950401	1.585097	2.341426	1.538883	1.778203	1.096298	2.476311	0	0	1.182979	1.909816	2.254475	0	0	0	0	2.428861	1.042062	0	2.395442	1.850557	2.178475	3.517377
AACSP1	0	0	0	0	0	0	1.096298	1.498706	0	0	0	0	0	1.913269	0	3.44295	7.498021	0	0	2.295325	0	4.711227	1.328528	0.903733
LINC00239	4.576958	3.812881	5.954395	3.428046	5.997471	6.2694	3.873483	5.229504	3.252214	4.880253	7.376417	5.608768	5.53167	5.036331	4.696556	3.941454	3.74856	3.885616	4.436547	5.521974	0	4.199747	5.697271	2.164658

Gene	Resistant KRAS	Resistant KRAS	Resistant WT	Resistant KRAS	Resistant BRAF	Resistant KRAS	Sensitive WT	Sensitive WT	Resistant WT	Resistant WT	Resistant KRAS	Resistant WT	Resistant KRAS	Resistant WT	Sensitive WT	Resistant KRAS	Sensitive WT	Resistant BRAF	Resistant KRAS	Resistant WT	Resistant KRAS	Resistant KRAS	Resistant WT	Resistant KRAS
NPY1R	0	0	0	0	0	0	0	0.923506	0	0	0	1.37396	0	3.998289	0	0	0	0	0	0	1.672125	0	0	0
ADAM23	0	0	1.223673	0	0	0	2.202074	0	0	0	0	3.399289	0	5.252738	0	0	0	1.297861	0	0	0	0	2.508614	0
FOXD3-AS1	1.174725	0	1.223673	0	0	0.626961	0	0	0	0	0	1.37396	0	5.601355	0	0	0	0	0	0.864812	0	0.706894	0	0.904236
ADORA1	3.467277	0	0	1.531951	0	0	3.036118	0	0	0	0	0	0	0	6.581452	1.504553	5.650519	0	0	0	4.027495	0	2.1757	0
S1PR3	0	0	0	0	1.603113	0	0	0	0	0	0	3.163346	0	2.089751	0.677882	0.68926	1.872301	0	0	0	0.789892	0	0	0
HOXA3	7.632466	9.176953	6.422876	4.012044	8.181864	6.074797	8.266036	7.458285	7.485262	7.540448	8.140331	2.529929	6.763102	4.686572	6.775601	7.630892	8.446862	7.899408	7.875632	6.683931	7.871983	8.238252	7.336797	7.45377
ADD2	0	1.289558	2.32372	0	0	1.8959	0.782644	0	2.523927	0	0	5.233107	0	4.792091	0	0	0	4.420952	0	0	0	0	3.006495	0
NIPAL4	1.174725	0	0	1.531951	0	0	3.269538	0	0	0	0	5.405552	0	1.203326	0	0.68926	0	0	0	0	1.297421	0	1.119477	0
ARHGAP18	9.236831	7.433789	8.037489	8.560185	8.344469	7.271416	10.64453	7.750311	8.600818	8.348208	7.157732	6.494118	8.068475	5.011888	8.55868	7.543606	8.490214	7.836353	7.220463	7.445036	9.06425	7.043117	8.467514	7.672934
OR2W3	0	0	0	0	0	0	0	0	0	0	0	4.890086	0	3.140156	0	1.504553	0	0	0	0	1.297421	0	2.1757	3.722657
KC6	0	0	0	0	0	0	3.157544	0	0	0	0	0	0	0	4.497766	0	2.936006	0	0	0	0	0	0	0
RAB39A	0	0	0	0	0	0.626961	0	0	0	0	0	0	0	4.95243	0.677882	0	0	0	0	0	0	0	2.508614	0
TRIM58	0	0	0	2.738832	0	0	0	1.482042	0	0	0	6.182523	0	5.676772	0	5.064373	0	0	0	0	0	0	3.913817	4.134181
CYP4F8	0	0	2.32372	3.626509	0	0	1.956475	5.822643	0	0	1.145263	10.35652	0	6.526521	0	6.055994	1.582975	0	0	0	1.672125	0	1.119477	2.995019
FOXQ1	9.702976	8.767536	8.795567	7.467069	8.674532	6.451953	10.55412	7.465623	8.652837	5.638553	8.309688	3.602014	6.832185	4.216178	8.401227	9.741448	8.550335	10.04783	9.729956	5.720537	10.11599	10.52636	7.603572	7.302681
APBA2	0	0	0	0	0	1.062548	0	0.923506	0	0	0	2.529929	1.046499	7.21145	0	0.68926	0	2.426512	0.80896	0	0	1.179238	3.375938	0
RIPPLY2	0	3.474011	0	0	0	4.203034	0	0	0	0	2.212753	3.937937	0	2.634778	0	0	0	0	0	0	0	0	0	0
HOXA2	4.217616	6.012556	3.971724	0	6.768356	3.756926	5.41874	4.021855	3.022755	3.235214	5.399286	0	4.256536	0	4.153326	4.364474	6.048724	6.317713	5.270432	4.933558	5.596383	6.033272	5.311014	5.265917
CACNG2	0	0	0	0	0	0	0	0	0	0	0	6.415589	0	0	0	0	0	0	0	0	0	0	0	0
NUDT16	8.829586	8.79125	8.812807	8.516137	8.482391	8.425905	7.59481	9.656694	9.369493	7.746735	8.471123	9.250468	8.474986	9.497243	7.968737	8.688497	8.894869	8.228921	8.84215	8.81424	8.746917	8.884736	8.617196	9.325512
NKX2-1-AS	0	0	0	0	0	0.626961	0	0	0	0	0	0	0	7.014755	0	0	0	0	0	0	0	0	0	0
IFNGR1	8.601063	8.361544	8.943772	8.678147	9.238983	8.663277	8.357585	8.378451	9.151774	9.304348	8.050862	7.140363	8.295817	7.728174	8.48089	9.065212	9.787641	8.542851	8.327911	8.520468	8.40302	8.469429	8.353172	8.502612
PERP	10.30754	10.59628	10.49175	10.77075	11.61631	9.99812	11.50405	10.14321	10.90733	10.55156	10.21361	10.04924	11.23998	8.020067	10.57805	11.93735	11.74814	10.96741	10.24095	10.29327	10.98287	10.88582	10.54139	10.21756
PTPRM	4.962547	0	0.737862	0	5.235867	0	5.62231	0	0	0	0	1.37396	0	1.850158	6.429114	0.68926	4.245102	2.426512	2.250883	0	3.743308	2.261144	0	0
SLC66A1L	1.174725	0	0	0	0	0	0	0	0	0	0	2.064752	0	2.295164	0	0	0	0	0	0	1.672125	0	1.119477	0
UNC5D	0	0	0	0	0	0	0	0	0	0	0	3.602014	0	0.723645	0	0	0	1.297861	0	0	0	0	0	0
INA	0	0	0	0	0	0	0	0	0	0	0	0	0	6.409799	0.677882	0	0	0	0	0	0	0	0	0
IRX6	0	0	0	0	0	0.626961	0	0	0	0	0	0	0	2.634778	0	0	0	0	0	0	0	0	0	0
FLJ40288	0	0	0	0	0	0	0	0	0	0	0	3.602014	0	0	0	0	0	1.297861	0	0	0	0	0	0
NKX2-8	0	0	1.223673	0	0	0	0	0	0	0	0	0	0	7.022003	0	0	0	0	0	0	0.789892	0	1.742164	0
NPR1	0	0	3.060865	0	0	2.092833	0	1.883682	0	0	0	2.064752	0	4.611679	0	0	0	0	0	0	0.789892	0	1.742164	1.85386
YTHDC2	7.37271	7.30792	7.882835	7.820946	7.903543	7.849862	7.827617	7.982869	8.627062	7.757998	7.465324	0	8.256145	8.134838	8.544844	7.795044	7.776203	8.127222	8.053577	7.926149	7.488577	8.034161	7.637953	8.446049
PRR16	0	0	1.586456	0	0	0	0	0	0	0	0	0	0	7.95339	0	0	0	0	0	0	0	0	0	0
CREBL2	7.623294	7.766064	7.88691	7.497594	7.85357	7.568906	7.610847	8.543567	8.318983	7.029192	7.082329	7.677732	7.550474	8.896287	6.325029	6.976605	7.030463	7.628748	7.921099	7.16864	7.784063	8.131704	7.594847	6.983036
PRIMA1	0	0	0	0	0	0	0	0	0	0	0	0	0	2.089751	0	0	0	0	0	0	0	0	0	0
CNNM1	0	0	0	0	0	0	0	0	0	1.424024	0	0	1.646635	7.224076	0	0.68926	0	0	0	0	0	0	0	0
SIX3-AS1	0	0	0	0	0	0	0	0	0	0	0	0	0	1.850158	0	0	0	0	0	0	1.297421	0	0	0.904236
APBB2	8.489394	8.135654	8.248578	8.911418	8.942964	6.691115	9.71817	8.522613	8.627062	8.825963	8.010753	6.27439	8.756664	8.066376	9.626297	7.250551	7.652861	8.41921	7.398183	8.200328	8.61366	7.589334	7.850786	7.750889
RAB8B	6.786627	4.078085	4.439542	5.131416	6.003993	5.36733	5.724111	6.254073	5.971978	6.230097	4.657484	3.937937	4.731851	3.669513	7.08058	5.090541	5.126386	4.843691	5.724771	4.894264	6.913453	5.52018	4.865146	6.327533
MIR4787	0	0	0	0	0	0	0	0	0	0	0	0	0	2.295164	0	0	0	0	0	0	0	0	0	0.904236
ITGA7	6.476136	6.546124	5.674622	7.251163	5.235867	6.051307	4.302122	6.931412	6.474142	7.65332	5.556148	6.616188	5.79028	10.28277	4.89675	6.108161	6.587012	6.419558	6.222861	7.092566	5.701166	6.727368	5.869414	6.47617
ZYG11A	0	1.959423	1.223673	0	0	0	0	0	0	0	0	0	0	5.277176	0	0	0	1.969853	0	0	0	0	0	0
ANXA3	9.955891	7.421685	7.772618	8.793354	10.11855	6.807856	9.795043	9.107441	10.91268	9.467602	7.002767	5.458739	7.448558	3.875617	5.972193	7.116891	9.750238	11.46027	3.934234	8.558669	10.22105	3.770376	7.085961	7.73327
SCARNA11	0	0	0	0	0	0	0	0	0	0	0	0	0	1.850158	0	0	0	0	0	0	0	1.534489	0	0
C5orf63	6.434793	6.194296	6.178798	5.702593	6.56601	5.049378	6.63921	5.238996	6.267222	6.52199	5.075546	0	6.09016	6.075482	5.765037	6.844446	5.611788	6.824199	7.277514	7.021403	6.737377	7.278707	5.080449	6.558516
PLCXD3	0	0	0	0	0	0	0	0	0	0	0	0	0	3.24299	0	0	0	0	0	0	0	0	0	0
FZD10	0	5.213865	0	1.531951	5.155655	2.560415	11.29663	0.923506	0	0	5.269324	0	6.260239	0.723645	3.405533	1.786523	8.131454	4.915177	0.80896	1.401742	0	0	3.669801	2.165499
LINC00867	0	0	0.737862	0	0	0	0	0	0	0	1.145263	3.163346	0	1.850158	0	0	0	1.969853	0	1.401742	0	0	0	0
PAQR9	0	0	0.737862	0	0	0	0	0	0	0	0	0	0	2.089751	0	0	0	0	0	0	0	0	0	0
NDST3	1.813523	0	0.737862	0	0	0	0	0	0	0	0	1.37396	0	2.474941	0	0	0	0	0	0	0	0	0	0
LAMC2	9.829994	8.019987	8.129797	8.900037	8.805156	7.256086	9.224274	7.843377	8.813821	8.953902	6.666539	7.073735	8.521742	3.24299	9.91776	8.90487	5.650519	9.288401	8.133012	7.451819	9.924747	8.659633	7.541359	8.981937
RNF150	0	0	0	0	0	0	0	0	0	0	0	0	0	2.909484	0	0	0	0	0	0	1.297421	0	0	0
IL17RE	7.928945	8.556806	6.0097	8.16318	7.85357	7.016326	7.517521	7.227427	8.767383	8.078592	8.044254	9.669621	7.590903	10.12426	7.337563	6.828986	7.652861	7.334948	7.454932	7.820064	8.249073	8.054891	7.46681	7.417456
SEPTIN8	10.07673	8.904304	9.430597	9.962839	9.575926	9.491916	9.855742	9.791263	10.20088	9.741688	9.446715	8.972751	9.291068	7.705843	9.652338	9.567211	9.331668	9.982763	9.788782	10.09633	9.782732	9.628185	9.421742	9.783439
HOXA5	5.227566	6.106286	5.979485	2.258076	8.674532	6.460895	8.116801	6.572215	6.025574	2.596932	5.175686	0	7.188931	3.140156	7.364068	4.804677	5.282496	9.13216	6.830258	1.79215	6.073264	7.455417	6.567037	7.957225
OOEP	0	0	0	0	0	0.626961	0	0	0	0	0	0	0	0	0.677882	0	4.026947	1.297861	0	0	0	0	0	0
NKX2-1	0	0	3.461468	0	0	0	0.782644	0	0	0	0	0	0	10.51563	0	0	0	0	0	0.864812	0	0	0	0
POLK	6.476136	5.766799	6.650875	6.634765	6.821314	6.504793	6.132309	6.28758	7.595697	7.047666	5.944699	4.329247	6.645192	5.252738	6.806852	6.611291	6.461937	7.596492	6.94035	6.600938	5.976372	7.341922	6.455796	5.994396
BVES-AS1	0	0	0	0	0	0	0	0	0	0	0	0	0	2.089751	0	0	0	0	0	0	0	0	1.119477	0
CHST2	0	0	0	0	0	0	0	0	1.755198	0	0	0	0	5.370989	0	0	0	0	0	0	1.672125	0	2.508614	0
PTGER3	0	0	0	0	0	0	0	0.923506	0	0	1.145263	0	0	4.649611	0	0	0	0	0	0	0	0	0	0
PAK3	0	0	0	0	0	0	0	0	0	0	0	0	0	3.998289	0	0	0	1.297861	0	0	1.297421	0.706894	0	0
PHF21B	0	0	2.117201	0	0	2.420743	0	0	0	0	0	0	0	6.474633	0.677882	0	0	1.969853	0	0	0	0	0	0
IL24	0	0	0	1.531951	0	0	0	0.923506	1.755198	0	0	3.937937	0	2.295164	1.485101	0	0	0	0	2.099096	0	0	1.119477	0
WSCD2	0	0	0	2.738832	0	0	0	1.482042	0	0	0	0	0	7.119834	0	0	0	2.426512	0	0	0	0	0	0
RNF180	0	0	0	0	0	0	0	0	0	0	0	0	0	3.875617	0	0	0	0	0	0	0	0	1.742164	0
LCK	3.890688	7.397168	6.124605	3.832116	3.194075	5.477181	8.388923	1.883682	5.971978	2.12653	6.782248	0	3.892552	0	8.502525	6.681839	0.735773	4.983288	7.999495	0	6.088799	8.387358	4.805896	4.937006
SUOX	9.162798	9.43054	8.000352	8.447434	8.171703	8.235814	8.186229	9.66787	9.058432	8.333227	9.210402	9.074227	7.660936	9.78934	7.605345	8.537358	6.702102	7.154698	7.35907	8.487843	9.066213	7.876184	8.37372	8.705987
POGLUT2	6.348372	5.877285	5.405909	5.207251	5.859558	7.505497	6.376986	4.434598	6.655073	5.777855	6.463593	3.779731	5.088772	2.295164	5.915978	6.407803	6.79146	4.843691	5.91657	6.36263	6.119375	5.871302	7.216823	5.47825
TBC1D16	10.16154	9.478071	10.08572	10.75819	10.17559	10.08455	10.38724	9.849917	9.96406	9.665984	9.634395	9.669621	10.33821	7.784666	9.874167	10.64728	9.938068	9.201713	9.735057	9.553605	10.12072	10.073	10.48467	9.77772
GOLGA6L7	0	0	0	0	0	0	0	0	0	0	0	0	0	4.055923	0	0	0	0	0	0	0	0	0	0
NOVA1	1.174725	0	0	2.738832	0	2.092833	0.782644	0	0	0	0	0	0	5.974488	0	0	0	0	0	0	0	0	0	0
GASK1A	1.813523	0	0	0	0	0	0.782644	0	0	0	0	5.105511	0	2.089751	0	0	0.735773	0	0	0	0	0	3.913817	0
ALDH1A1	11.59488	12.42037	11.6168	11.35595	11.88706	12.46232	14.00043	11.94933	14.04287	10.77728	12.61814	5.037216	7.188931	3.029425	10.27352	9.479576	13.01249	7.386165	7.704164	11.07055	10.92455	7.649632	11.64336	10.01737
NKX6-1	1.174725	0	1.223673	0	0	0	0	0	0	0	0	0	0	2.778659	0	0	0	0	0	0	0	0	0	0
GRID1	0	0	0	0	0	1.667771	0.782644	0	0	1.424024	0	2.064752	0	4.216178	0	0	0	1.969853	0	0	0	0	0	0.904236
FCMR	2.254743	0	2.664852	2.258076	0	2.092833	2.202074	2.197512	3.392717	1.424024	0	6.833125	1.646635	4.449111	3.321471	0	0	0	0	3.545268	2.772216	0.706894	2.508614	2.827997
PRR19	3.762878	1.289558	4.860144	3.832116	3.194075	3.813873	3.269538	3.939932	1.755198	2.596932	4.91096	5.653809	4.085992	8.097586	3.944384	4.276026	4.520709	3.820461	4.361112	3.735147	4.151027	4.124281	3.530338	3.624124
MAP3K11	10.28736	9.954122	9.979995	10.06828	9.079658	9.601101	9.678141	10.32464	8.888042	9.981564	10.33092	10.98426	9.700612	10.55114	9.368	10.02623	9.766859	9.56612	10.0268	9.84262	10.28144	10.18773	9.910792	10.45015
SCN4A	2.592156	0	1.876099	3.626509	0	0.626961	0	1.883682	1.755198	0	1.145263	1.37396	0	6.306735	0	0.68926	0	1.969853	0	0	0	0.706894	1.742164	0.904236
RPL39L	4.708737	5.604948	0	0	0	6.330361	0	0.923506	1.755198	3.854658	6.203824	7.486093	0	5.695026	0	0.68926	0	0	0	2.099096	5.334964	0	5.311014	0
ARHGEF38	5.492252	6.694348	5.336085	6.330095	7.186823	5.441485	5.643253	6.444353	6.311049	6.440451	5.730916	5.170719	6.067418	3.593705	7.364068	6.042652	4.855101	4.51599	5.91657	5.365763	5.959572	6.442792	5.869414	5.420704
SLC39A4	5.749934	10.94602	9.722767	9.604582	9.153797	10.07803	6.759181	9.851318	7.762821	10.14705	10.67257	10.06219	9.397468	11.68115	6.165411	9.68889	6.596961	9.870347	8.093841	9.578609	4.948917	8.524965	8.901717	8.799257
KCP	4.116654	1.959423	5.917086	5.851823	2.343681	1.396666	2.595018	2.863351	4.484237	3.676189	0	5.037216	1.046499	8.045187	1.485101	3.512449	2.319542	5.110561	2.002808	3.545268	3.28501	1.179238	2.508614	1.85386
HBQ1	1.813523	3.650335	0	3.386649	1.603113	0	1.660309	2.455122	0	0	0	5.170719	0	5.348102	0	1.504553	4.193577	0	0.80896	0	0	0	2.1757	0
CAVIN1	6.079962	6.753489	6.541375	4.44688	5.311853	5.185075	7.523181	6.336426	3.392717	5.588961	5.82644	5.105511	6.394204	3.513692	6.775601	8.823344	9.791978	9.420176	9.233156	5.697895	8.283231	8.967243	6.084126	7.570821
OLFM1	0	0	1.586456	0	0	0	0	3.853076	0	0	0	1.37396	0	7.56903	0	0	0.735773	1.297861	0	0	0	0.706894	0	0
PPFIA3	8.489394	9.233441	8.649693	8.415817	6.921732	8.716517	7.762112	8.091202	8.992671	8.697312	9.713575	11.01899	7.37638	9.54331	5.44576	8.523018	9.264589	7.993241	8.041275	8.801021	8.152588	8.182826	8.713035	7.785495
STAMBPL1	7.000082	6.674083	7.781402	7.303932	7.66363	6.572364	8.210989	7.637892	8.160311	7.723942	6.250498	7.407114	7.146162	3.810133	8.125682	7.038405	7.059536	7.618076	6.251621	7.135324	7.831004	5.329185	6.719303	7.654331
NXPE1	4.116654	0	2.117201	0	0	0	0	1.482042	0	0	0	5.292909	0	0.723645	0	0	0	0	0	2.567208	2.215594	0	1.119477	0
TCERG1L	0	0	0	0	0	0.626961	0	0	1.755198	0	0	2.064752	0	4.405436	1.765176	0	0	0	0	0	0	0	0	0
ERCC6L2	6.802966	7.184411	6.770404	7.497594	7.573891	7.064055	7.56768	6.931412	6.845928	6.719271	6.813672	5.405552	6.81863	6.566729	7.048256	6.646996	6.66475	6.208128	7.153463	6.094456	6.93939	7.084031	6.928208	6.403764
CD6	0	1.289558	2.117201	2.258076	1.603113	3.571211	0	2.197512	0	1.424024	1.775568	4.329247	2.395624	4.722609	1.137227	2.560629	1.872301	2.772849	0	1.79215	1.969301	1.179238	2.1757	2.421632
B4GALT1	9.954063	8.823809	9.41657	10.48489	8.765169	8.238417	10.05644	10.00401	9.133583	9.375543	8.436217	8.222401	9.04847	7.968476	9.200147	9.962124	9.481509	9.43241	9.259945	8.301415	9.852645	9.392007	9.059068	8.368276
SMOC1	3.622635	9.009139	0	5.702593	7.393299	9.201764	9.232935	0	1.755198	2.596932	9.228003	0	4.173782	1.562693	9.483712	1.786523	6.048724	1.297861	0.80896	1.79215	5.483388	0	6.766685	4.453997
SLC2A1	11.51778	11.40458	10.91837	12.16679	11.96928	12.20262	12.87112	9.126083	10.15952	11.74184	10.74873	9.390496	10.14267	8.328513	10.11089	12.30524	12.41847	12.0202	10.46086	9.922914	11.19734	10.39636	10.79382	10.28159
SMOX	6.208183	8.283433	7.49675	6.187592	7.166501	7.474924	7.349288	8.02321	7.630706	9.241543	7.759634	3.163346	7.300401	6.13017	7.074173	7.924338	8.11077	7.031428	6.711066	8.793032	7.139117	7.369766	6.885919	6.108131
STAC2	0	0	0	0	0	0	0.782644	0	0	0	0	0	0	1.203326	0	0	0	0	0	0	0.789892	0	1.119477	0
CDK6	9.406825	8.930168	9.471879	9.297068	9.704827	9.478769	10.4495	9.601143	9.332757	8.223757	8.379598	9.73036	7.727726	3.593705	10.05586	9.778138	9.205663	8.263572	9.698966	8.635147	9.737866	9.680258	9.835019	9.209242
MVB12B	7.844641	7.902965	8.077265	7.251163	7.265367	8.35812	9.189101	6.962864	8.008868	6.573896	8.287477	7.204048	6.910941	6.734962	8.463835	8.219368	8.851892	5.741765	7.629432	7.08386	8.033217	7.536266	7.836053	7.967309
CHSY3	0	0	0	2.258076	0	1.062548	0	0	0	0	0	2.064752	0	2.778659	0	0	0	0	2.250883	0	0.789892	0.706894	1.119477	0
DOCK3	4.00809	4.078085	2.117201	4.44688	3.932237	3.432632	2.903526	2.673628	5.971978	3.854658	3.841017	4.210226	2.661649	9.396271	2.80692	3.512449	2.936006	5.436314	3.703894	4.679934	3.662562	3.701929	3.669801	4.334438
WBP11P1	0	1.289558	0	0	0	0	0	0.923506	0	0	0	2.064752	0	0	0	0	0	0	0	0.864812	0	0.706894	0	0
PRSS1	0	0	1.586456	2.258076	2.830649	1.8959	3.80375	0	6.12714	0	0	2.064752	3.992512	0	7.048256	4.276026	6.077435	0	2.002808	0.864812	3.819772	0.706894	1.119477	0
LINC02159	3.095078	0	1.223673	0	0	0	0	0	0	0	2.212753	1.37396	0	4.111343	1.137227	0	0	0	0.80896	0	3.28501	1.534489	3.375938	0
FOXN2	7.788308	6.430075	7.128068	7.733882	7.649056	6.604999	7.187614	7.38278	7.066527	7.029192	6.155589	5.653809	6.54997	6.306735	7.326824	6.299793	7.303857	7.124854	7.161063	6.525793	7.225978	7.511484	7.158808	7.357381
EFS	0	0	0	0	0	0	0	0	0	0	0	3.163346	0	0	0	0	0	0	0	0	0	0	0	0
PNMA6A	0	0	0	0	0	0	0	0	0	0	0	0	0	3.029425	0	0	0	0	0	0	0	0	2.1757	0
CLDN18	0	0	0	0	0	0.626961	0	0	0	0	0	0	1.646635	6.221152	0	0	1.582975	1.297861	0	0	0	0	0	0
A4GALT	2.865422	1.959423	1.876099	1.531951	0	2.092833	4.248461	3.441472	0	1.424024	2.547809	0	2.395624	1.203326	2.925559	4.487741	7.736249	0	3.325297	0	2.215594	3.204722	0	2.827997
SIX2	4.311973	0	1.223673	0	1.603113	1.8959	1.660309	0	0	0	0	4.965526	0	3.140156	0	3.348635	1.872301	0	0	0	4.515282	0	1.742164	0
ANTXR1	0	0	0	0	0	6.10933	1.287213	1.482042	0	0	0	0	0	8.69106	0	0	0	2.426512	0	0	0	0	4.541079	1.455811
S100A11	12.63997	12.07687	11.32623	11.69907	12.391	10.90256	11.2307	10.92296	10.92507	12.83433	12.20531	12.31165	11.41963	4.722609	11.32514	12.57716	11.91923	13.58349	11.10407	12.13143	12.00033	10.94856	10.7493	11.36921
HOXA9	5.178314	7.198669	8.503408	6.634765	9.305546	7.903322	9.007197	7.962267	0	1.424024	6.29571	2.529929	8.567031	2.295164	9.627392	8.789797	5.743027	8.207724	9.095171	2.352056	7.422681	9.289324	6.396784	8.456825
ZFHX3	6.971455	7.937379	7.155354	6.149652	7.124976	7.075745	8.50788	8.640763	7.426704	6.293392	7.016335	6.388433	7.260856	6.341914	7.776914	6.698949	6.825709	6.878863	7.667282	6.683931	7.175947	8.071941	7.970286	7.629146
PGGT1B	5.968492	4.59171	6.830886	6.839279	6.003993	6.12066	6.389435	6.516782	6.512189	6.440451	5.972809	3.779731	6.280156	6.318557	7.048256	5.552568	6.034152	5.335687	6.603617	6.023388	6.207396	6.2405	5.953719	6.263507
PID1	0	0	4.299807	0	0	0	3.157544	0	0	1.424024	0	4.080496	1.046499	8.686506	0.677882	1.153748	1.872301	4.843691	3.526979	2.567208	3.172872	2.261144	0	1.455811
ZNF274	7.780078	8.855648	8.381612	8.104893	8.251052	8.227978	7.642395	8.631007	8.909583	8.427938	8.887607	7.560972	6.89811	9.620547	0	7.567093	7.549062	7.908195	6.877154	8.290106	7.239959	7.729168	8.91229	8.669395
HOXA-AS3	4.116654	5.213865	3.461468	1.531951	6.049094	3.697638	5.643253	2.863351	1.755198	0	3.958134	0	1.646635	0.723645	4.743672	3.432866	3.056269	3.486777	5.66188	0.864812	4.26481	5.597574	4.611978	5.265917
RTN4RL1	1.174725	3.273097	4.56693	2.258076	1.603113	0	0	5.627106	0	0	4.349777	8.135241	0	7.843127	0	0.68926	0	1.297861	4.957818	0	1.297421	4.175662	2.778926	8.002057
FOXL2	0	0	2.32372	0	0	1.062548	0	0	0	0	0	0	0	5.989356	0	0	0	0	0	0	0.789892	0	4.466515	0
RUNX1	8.406277	7.656711	8.007856	9.3186	8.863132	7.379116	9.916143	8.374559	7.613308	8.239913	8.395261	8.798035	8.749539	4.890416	9.249318	9.263694	7.727218	9.549366	8.377684	7.605703	8.89785	8.189091	8.14182	7.022256
EGFL6	0	0	0	0	0	0	0	0.923506	0	0	0	0	0	3.741536	0	0	0	0	0	0	0.789892	0	1.742164	0
EXOC2	8.110572	7.67722	7.92308	7.891606	8.065957	7.979971	8.464413	7.311272	7.9955	7.834468	7.860876	7.021665	7.260856	6.77841	8.69019	7.984885	7.648084	8.001476	9.209529	7.587341	8.550551	8.999681	8.551077	7.424792
LIFR	0	0	0	0	0	0	0	0	0	0	0	0	0	4.265871	0	0	0	0	0	0	0	0	3.006495	0
KIF5C	0	2.415109	2.32372	0	0	1.396666	2.595018	0.923506	0	2.596932	0	3.779731	0	8.748995	0.677882	0	4.982005	1.969853	0	0.864812	1.969301	0	6.203716	0
CXCR4	1.174725	7.170012	2.809275	2.738832	8.065957	6.691115	6.7104	8.393918	0	8.600529	7.205896	1.37396	5.298601	0	11.55676	3.915185	3.45672	6.559996	4.690098	4.122842	0.789892	3.770376	3.669801	5.663304
SLIT2	3.293148	1.959423	1.223673	3.386649	0	2.092833	1.660309	4.493216	0	1.424024	0	4.080496	0	4.449111	0	4.132274	2.660454	0	0	0.864812	2.425909	0	1.119477	0
SPATA2L	7.405182	7.538384	7.182134	8.191464	6.921732	7.505497	7.284111	8.666462	7.040728	7.119284	8.102665	8.766742	7.31012	8.635434	6.710992	8.36047	7.102075	7.5183	7.339109	7.761218	7.675081	7.290409	8.111555	7.709438
LINC02561	0	0	1.876099	1.531951	0	0.626961	2.595018	0	0	0	2.547809	0	1.646635	0	2.80692	1.153748	2.319542	0	1.324175	0	0.789892	1.534489	1.119477	1.455811
HPCA	0	1.289558	2.32372	2.258076	3.932237	1.062548	0	4.172941	3.686908	0	0	3.602014	0	6.06148	1.999619	1.504553	0	4.768477	2.002808	0.864812	1.672125	2.439889	0	2.639075
TOP3A	7.796492	7.656711	7.559373	6.815245	8.119799	8.209527	7.941902	7.762278	8.652837	7.927182	8.134121	7.597003	7.668511	7.029216	9.276083	7.894942	8.452353	8.72794	9.202181	8.710604	8.451891	8.751391	8.217661	8.345263
HTRA3	0	0	0	0	0	0	0	0	0	0	0	0	0	3.029425	0	0	0.735773	1.969853	0	0	0.789892	0	1.119477	0
ARMCX1	0	1.289558	0	0	0	0	3.036118	0	0	2.951088	0	0	0	7.839029	0	0	0	0	0	0.864812	0	0	1.119477	1.455811
NMNAT3	5.532003	7.017514	6.20515	6.549924	7.35789	6.2501	6.73011	7.584971	6.512189	6.599166	5.82644	3.399289	7.533981	5.765817	6.799102	6.797559	6.472784	5.530379	7.960829	7.075101	6.727487	8.464273	6.751063	7.060438
GHR	0	0	0	0	0	0.626961	0.782644	0	0	0	0	0	0	3.029425	0	0	0	1.297861	0	0	0	0	0	0
SEC14L5	1.813523	1.289558	1.586456	0	0	1.667771	1.660309	2.673628	1.755198	0	1.775568	2.881125	0	4.611679	0	1.153748	0.735773	0	1.703032	1.79215	0.789892	2.872407	0	0.904236
AACSP1	0	0	0	0	0	1.667771	0.782644	0.923506	3.022755	0	0	0	0	0	7.197204	0	4.026947	1.969853	1.703032	0	0.789892	2.872407	0	0
LINC00239	8.057082	4.901622	3.060865	6.661972	3.72551	4.873027	3.269538	4.93507	4.62978	7.855592	5.075546	0	5.581427	2.778659	3.484965	5.643755	3.056269	6.537519	5.454868	5.627754	5.226789	6.727368	5.129604	5.506184

IL20RA	7.613741	7.893799	7.303981	7.57418	7.731537	8.696647	7.586412	9.228559	7.733255	7.261315	6.849756	7.890175	8.120046	7.814396	7.008616	8.816227	6.67798	7.866587	7.971335	7.14681	8.003843	8.448686	6.850091	2.638167	8.155803	8.20085	7.899068	7.759298	7.558376	7.87684	8.913331	8.261066	9.343349	7.700783	7.650747	8.033736	7.992043	0.723645	6.408889	8.474161	8.446862	8.263572	8.432092	7.55621	8.203422	9.024417	7.367856	7.609965
RERG	4.451459	0	1.000101	0	0	0	0	0	3.927307	0	0	0	0	0	1.35509	0	0	0	0.613139	0	4.61103	0	0	0	0	0	0	0	0	0	0	0	0	0	0	4.63675	0	1.203326	0	0	0	0	0	0	0	0	0	0
TM4SF1	7.759869	10.90576	7.340051	9.218555	9.430925	10.17196	7.067727	10.06101	7.515198	8.781232	12.26114	7.640592	11.72135	8.690787	7.669289	8.258466	8.518704	7.205291	8.540083	8.770875	7.622224	7.290155	11.02221	0	10.46985	9.44257	10.98642	7.746646	8.415077	8.885438	7.562192	8.494193	8.43246	9.680854	9.239618	7.039231	9.583277	0.723645	8.97276	9.395484	7.832028	12.19733	2.250883	8.236126	8.997919	3.102226	7.122844	9.057038
MCTP1	3.99217	2.615475	3.170104	3.890574	3.480744	5.1301	5.135108	4.334272	2.396196	4.666002	3.636225	3.349371	4.31165	0	4.409778	4.100908	7.346353	5.069183	3.160882	3.875805	0	3.718435	3.533593	2.827067	4.217616	2.760887	3.62655	3.386649	4.418177	2.804754	4.134752	3.555893	3.022755	3.472485	4.586489	1.37396	2.069199	2.634778	7.499341	6.133554	4.678958	4.319208	5.091095	3.735147	3.577028	4.750806	3.20301	3.624124
ARHGAP29	6.121049	6.788661	4.000189	5.136334	7.376431	5.701264	5.310842	5.571683	5.074321	5.75055	10.02445	3.588618	8.458445	7.018452	5.754258	3.854591	6.299313	6.262891	6.281507	5.45927	1.046383	6.066706	7.738852	4.059719	6.079962	5.322014	6.641257	2.258076	7.542693	5.611737	4.302122	6.054425	8.361287	6.952835	4.789782	2.064752	6.375811	3.741536	6.314195	5.571272	5.795813	9.070872	5.898497	5.214455	5.552241	6.674768	4.021913	6.35851
ANXA5	9.608206	9.851154	8.876718	9.978794	9.34757	8.87399	9.552625	9.432932	9.382897	9.946365	11.61083	9.582512	10.50301	8.918191	11.25879	10.02316	10.82319	9.136647	8.568536	9.77567	8.988981	9.790245	10.67986	8.988298	9.898123	9.433557	9.518594	8.768536	11.41676	9.765303	10.01386	8.526127	9.725807	9.727435	9.833399	8.949714	9.138503	8.625951	10.50907	9.079981	9.750238	10.5357	9.693736	9.920471	10.16077	9.376994	9.672451	9.647382
RGL2	8.631912	9.334856	9.226614	8.354277	9.518499	9.434475	8.355885	9.606854	8.786685	9.451388	9.233436	9.910624	9.988062	7.645272	9.021973	9.506337	8.178064	8.58391	8.936067	9.739846	9.474893	7.046453	9.052622	10.13725	8.721994	10.10177	9.146045	9.360721	9.074213	8.805205	9.555074	9.095666	10.16252	9.016201	9.764716	10.10243	7.998068	10.9983	8.378006	8.793785	7.13659	10.25489	8.779344	8.980588	8.775997	9.292236	9.715346	8.846458
ZNF32	6.811899	6.859877	7.170125	5.794718	5.862224	6.040008	5.467476	5.870748	7.349056	7.70243	7.261663	6.822002	7.71647	7.487412	7.025969	7.437664	7.249418	5.761353	6.674553	6.955618	6.675362	7.241288	7.976724	4.33341	6.232515	6.88273	6.688719	7.420035	7.303091	6.0631	7.355646	6.931412	5.916313	6.412214	7.016335	6.892318	7.357757	4.95243	7.532435	6.265747	6.738512	7.563499	6.463131	6.897709	6.707501	6.819088	6.474942	5.80498
GFPT2	0	0	1.585097	0.858862	0	0	0	3.340484	0	0	0	4.583765	2.254475	1.913269	0	0.924276	0	0	0.613139	1.562827	2.885979	3.276196	0	6.570724	4.311973	0	0.737862	2.258076	1.603113	0	0	0	3.022755	0	0	2.881125	1.046499	5.252738	0.677882	0.68926	3.167274	1.297861	0	0	0.789892	0	3.530338	0
ZNF556	0	0	0	0	0	0	0	0	0	0	5.241957	0	0	0	2.041335	0	1.166132	0	0	0	0	2.824072	6.044239	0	0	0	0	0	1.603113	0	0	0	0	0	0	0	1.046499	0	0.677882	0	3.904107	0	0	0.864812	0	0	0	0
NFE2L3	10.49548	10.21143	9.698906	9.986914	10.33445	9.945378	9.227895	8.945671	10.1721	9.762815	10.10986	9.177866	8.906872	9.910492	9.29317	10.46651	9.044047	9.180914	9.842727	9.658452	10.12466	10.24949	9.84307	7.123843	10.5971	9.6199	10.12679	10.02178	9.480372	10.25553	9.974116	9.730124	8.537659	10.37079	9.662713	9.284009	9.7988	6.77841	9.305099	10.21944	9.891333	10.11388	9.281024	9.697512	10.06585	9.484048	9.273804	10.04739
CORO6	4.240015	5.471091	4.907086	3.314649	5.402246	3.884591	4.175589	3.05373	3.628893	5.577163	4.02196	4.789568	5.589316	1.252965	3.751148	3.318652	4.048859	4.557642	4.694348	4.722804	5.515526	6.13963	4.987916	8.763904	4.311973	4.901622	5.515261	4.677675	2.343681	5.227612	2.411883	3.939932	4.883069	4.803913	4.657484	5.825617	3.892552	7.732599	3.765065	4.564432	5.282496	4.51599	3.934234	3.440052	2.215594	4.940986	4.744108	4.395456
TRIM36	5.576783	6.720139	5.170121	3.05701	5.677601	4.353316	6.313349	3.964133	5.012136	2.827144	7.890592	3.886361	5.046956	6.044732	6.538068	4.174478	6.686718	6.652413	4.364195	3.669699	0	4.330096	6.550024	3.517377	3.762878	3.474011	7.750423	4.315976	7.124976	1.8959	4.302122	5.095022	5.195186	5.371556	3.958134	0	5.548961	5.068992	6.694378	3.727825	5.041502	4.209741	6.193516	5.502697	4.948917	4.940986	3.20301	5.420704
ARL15	6.773082	5.601485	6.672625	5.463398	5.217548	5.92931	5.351624	5.298061	6.566982	6.251575	6.067455	6.255023	5.408972	5.095899	5.623576	6.933027	6.695403	5.904003	5.347623	6.11689	5.218074	6.13963	5.502593	4.665916	7.000082	5.766799	5.312039	6.490458	5.699038	5.404884	6.628748	5.728185	5.285832	5.904882	4.789782	4.080496	5.644247	5.176836	6.581452	5.729519	5.510152	5.89107	6.003684	6.162186	6.841917	6.582599	5.980761	5.01685
KRBA1	0	0	0	0	0.709495	0	0	0	1.275828	0	0	5.435741	0	0	0	6.518392	6.19293	0	0	1.562827	6.871876	0	0	7.556457	0	0	0	0	0	0	6.117419	0	0	0	0	7.172557	0	7.719283	5.930238	0	0	0	0	0	0	0	5.810342	0
LURAP1	0	0.552086	1.000101	1.393533	1.538883	0	0	0	0	0	0	0	3.094778	1.252965	1.35509	0	0	0	0.613139	0	1.046383	0	0	3.721657	1.174725	0	0	2.258076	0	0	0	0.923506	0	2.596932	0	0	0	3.938256	0	0	0	0	0	1.401742	0	0	0	1.455811
HOXA1	3.899095	4.547068	0	5.102569	5.241971	4.750599	2.473063	0.935796	4.056163	4.109178	0	5.435741	5.408972	2.707797	4.607151	5.339857	3.453219	2.860731	4.077886	4.491695	0	5.194588	3.257759	1.853078	4.562851	5.516661	4.720973	3.626509	5.45278	5.674584	6.19038	4.099375	2.523927	3.676189	4.433072	0	2.661649	0	3.035178	4.740019	4.95131	5.170221	4.307334	3.902917	4.688413	4.681401	5.779874	4.334438
VTCN1	0	0.950401	0	0	0	0	0	0	0	0	4.13059	1.505082	0	0	0	2.198785	0.697815	0	0	0	0	0	2.709739	0	0	2.415109	0.737862	0	0	0	3.470398	0	0	0	1.775568	0	0	0	2.201237	0	0	0	0	0	0	0	1.119477	0
BLVRA	6.220577	4.71012	6.087662	3.533175	7.99128	7.639876	8.717477	9.975942	6.805647	6.843038	7.57211	6.339329	9.879607	7.769371	8.358465	7.005376	7.662083	8.26938	7.558212	6.566929	3.542686	7.120382	7.721737	4.85144	6.052892	4.860144	5.536127	8.171703	[illegible]	4.640943	7.361977	6.809841	8.371672	5.588961	3.713546	2.064752	8.27115	4.921756	7.653415	7.849693	7.235442	9.845402	6.053534	5.986497	6.35602	6.582599	6.314148	7.863153
NUDT16L1	8.068493	7.924576	8.092958	8.645589	8.002044	8.507311	8.262736	9.572669	8.720168	8.157406	6.9271	7.647223	8.259743	8.36126	8.862089	8.271098	8.13721	7.758062	8.93138	7.949795	8.924017	7.60542	7.920831	8.945615	7.987304	8.090494	8.226234	8.104893	7.951842	8.256504	8.443671	9.005409	8.48123	7.527343	8.719446	9.467124	8.329658	8.806793	8.106973	8.428728	8.15858	7.881672	8.304083	8.52369	8.442853	7.973756	7.357577	8.249354
EEPD1	9.459264	8.594516	9.194958	9.656933	9.627949	9.370593	8.748348	8.145168	9.640955	10.16642	9.889894	8.815353	9.335266	10.14006	8.671266	9.968625	9.044047	7.361821	8.614095	10.14536	8.630242	8.98729	9.622916	6.583882	9.454148	9.281116	8.756009	9.561889	8.474146	9.698826	9.776206	8.611293	7.344735	9.721694	9.171515	8.772005	10.2639	5.928942	8.97276	9.05029	8.255986	10.02579	9.503583	9.327061	9.166616	9.743345	8.5099	9.779152
GLI3	0	0	0	0	1.182986	0	0	0	0	0	0	0	0	0	1.35509	0	0	0	0	0	0	0	0	3.143737	0	0	0	0	0	0.626961	0	0	0	0	0	0	0	2.474941	0	0	0.735773	1.297861	0	0	0	0	0	0
TMEM154	5.546413	6.674777	5.523759	4.46948	3.841959	4.353316	3.336916	0.935796	5.246249	4.545628	5.380398	6.00955	6.455277	0	4.780742	8.2669	4.947582	5.246279	4.571198	4.056113	4.334584	8.598481	5.076746	0	6.881999	5.841388	6.929012	6.839279	4.549533	5.289155	7.958707	5.095022	1.755198	6.05853	3.958134	2.529929	0	2.634778	5.716493	4.406744	7.685865	5.818348	3.212791	5.603595	7.232985	4.817024	5.472139	2.165499
RNASE1	4.898816	0.552086	9.185077	7.965574	7.425235	6.709834	6.939834	11.21128	4.569372	8.78788	6.362976	5.834697	1.528973	0	7.505758	9.567358	8.590304	5.991824	8.936067	9.838609	1.646481	9.845785	4.843489	1.455124	8.682799	9.478071	0	9.136618	7.678058	6.998013	9.902093	8.776759	9.410362	6.293392	9.10322	2.529929	1.046499	2.909484	9.04498	7.166939	9.782201	4.209741	8.540951	5.651515	8.496251	7.324955	4.805896	7.002779
HOXA4	3.314412	3.157929	0	3.314649	5.444949	5.830096	2.742064	3.340484	2.396196	4.414289	3.776579	3.793777	6.024969	2.707797	5.623576	5.402437	3.876272	2.980064	2.062641	4.611873	0	4.848074	2.008245	1.455124	4.903157	5.373192	2.940548	2.738832	3.484125	3.107741	5.18165	3.181178	2.523927	3.854658	4.511819	0	3.892552	2.295164	3.56025	3.727825	3.541594	5.818348	4.251473	2.920068	3.486101	4.225275	4.216473	4.7627
B4GALT5	9.637315	8.815131	10.62865	9.166396	8.539062	9.4209	9.02539	8.751535	9.287677	9.008858	11.48091	9.338068	8.807146	9.918762	8.725378	9.297861	9.920209	9.217806	9.357762	8.935402	9.096773	9.848507	10.53601	8.95324	9.487386	8.925889	8.769316	10.29592	8.989982	9.158446	10.10065	9.137613	8.644296	9.163227	8.529066	8.569289	9.707983	8.948576	10.11791	9.922611	9.64388	8.89196	9.696353	8.693537	9.458824	9.22218	9.701189	9.033236
CECR2	0.995829	0.950401	7.644057	0	6.896672	0.855986	2.742064	2.695475	1.275828	0	2.266443	3.21301	1.528973	1.252965	0	2.456453	1.802476	1.809634	6.549626	0	6.705128	0	2.468424	7.807036	5.409304	0	0.737862	7.338069	2.830649	0	2.411883	6.237022	2.523927	2.951088	0	6.519372	0	7.242809	1.765176	0.68926	1.582975	0	0	1.79215	2.918484	0	2.508614	6.089787
C19orf81	0	0	0	0	1.182986	1.778203	0	0	0	0	0	2.703815	2.734961	0	0	0.924276	0	0	1.640777	0	0	0	0	5.532529	1.174725	0	0	0	0	0	0	1.482042	0	0	0	0	0	5.370989	0	2.02229	0	0	0	0	0.789892	0	1.119477	0
CD177	0	0	0	0	0	0	3.336916	0	0	0	1.182979	2.703815	0	0	0	0	0	3.090277	0	0	3.080217	1.850557	1.005502	4.203045	1.813523	0	0	1.531951	0	2.912975	0	0	0	1.424024	1.145263	3.937937	0	2.634778	0	0	3.056269	0	0.80896	0.864812	0	2.59891	2.1757	0
MSX1	4.946113	5.310885	6.78156	6.460348	4.498115	6.581494	7.1852	7.583501	5.447623	6.498538	7.056658	6.593909	6.325597	5.91844	6.822545	7.958691	7.405775	7.186802	7.272204	5.989555	1.046383	6.729615	7.341832	7.29362	9.162798	4.754972	5.47251	6.607035	6.003993	6.644781	8.934724	7.119924	5.798095	5.86377	5.022743	0	5.972686	6.853465	6.967587	6.740859	5.650519	6.687968	4.069608	5.720537	8.96673	5.027432	6.180578	4.270725
SERPING1	0	1.735965	0	0	0	0	0	1.096298	0	0	0	0	4.407522	0	0	0	0	0	0	0	0	0	0	5.9122	0	0	0	0	0	0.626961	0	0	0	0	0	0	0	8.733625	0	0	0	0	0	0	0	0	5.651128	0
FHL1	1.579399	7.521618	1.000101	0.858862	1.538883	0	2.742064	0	1.275828	0	1.182979	0	0	0	9.484183	0	1.802476	2.046811	0	0	8.045161	0.902111	1.592294	6.143067	0	0	7.356843	3.098801	9.708346	0.626961	0	0	0	0	0	7.073735	1.046499	5.765817	0.677882	0	2.113183	3.820461	0	0	0	0	1.119477	0.904236
CEMIP2	10.83636	9.613405	12.5471	10.07769	9.590401	11.13801	9.505966	9.35864	10.5195	9.943391	10.73205	9.928425	8.807146	10.05444	11.12125	11.10534	11.00928	9.954106	11.05946	9.70311	9.03072	8.871638	10.06626	8.095881	10.46215	10.90856	9.606482	12.2261	11.78077	9.090928	11.81099	10.73156	11.05157	10.28191	10.72723	8.085381	9.822762	8.271875	11.66678	11.31665	8.864302	8.909571	9.781404	9.529764	10.60618	9.485321	9.965965	9.735569
B4GALT7	8.146494	8.654165	8.413829	7.714911	8.499094	8.255304	8.355885	9.123854	8.560964	9.118417	7.647176	8.6403	8.070303	7.957753	6.513674	7.579837	6.276341	8.227783	8.483573	8.840184	8.570853	8.261234	8.10634	9.09094	7.87587	7.226767	8.729021	8.730489	7.060358	7.360123	7.813829	8.033122	8.123915	8.643673	7.710397	9.521256	8.033701	9.11881	6.39867	8.20147	8.335392	7.817819	7.067109	8.92318	8.235179	7.290409	8.65546	8.599801
KRBOX4	7.068524	6.400036	6.714445	5.876911	7.291232	6.837936	6.313349	6.65121	7.010377	6.592676	0	5.203552	6.391893	4.974198	6.054013	5.402437	0	6.643402	6.047426	6.653496	5.972464	6.580431	0	5.99333	6.7701	7.359593	5.979485	6.363625	6.2556	5.834503	5.512778	6.983457	7.257826	7.527343	7.107903	6.519372	5.67466	6.96297	1.137227	5.455226	6.348729	6.965605	5.804575	7.71122	6.088799	6.287969	5.080449	6.745903
MGST2	8.821387	8.846153	8.839405	8.782499	8.777007	9.429399	10.03187	9.061128	8.989911	9.45974	8.762295	8.739816	9.279446	8.138587	11.0855	9.429532	8.941911	8.670736	9.19783	9.056842	8.027598	8.614559	9.112939	6.951832	9.658772	9.38139	8.838286	8.787189	9.776978	9.123675	8.585674	9.40519	8.452166	9.88983	9.372163	8.409627	8.687507	6.803865	8.751707	8.971512	8.849813	9.441519	8.598057	10.29798	9.267325	8.51253	8.58231	9.004146
HDDC2	8.39255	8.465365	7.693688	8.769109	8.468374	6.764599	9.647799	9.992845	8.424385	8.684783	8.675303	8.610074	9.03168	8.946783	9.512159	9.157497	8.976172	8.748436	8.260073	7.926806	7.439506	9.395426	9.184641	7.878187	8.539011	8.067372	8.424298	7.969894	9.189485	8.046825	8.849435	8.151591	9.102744	8.587962	8.195048	7.620533	9.074365	7.487597	8.94318	6.333053	9.608661	9.24744	8.746885	8.865934	8.616342	8.894353	8.667992	8.653871
LINC01647	0	0.950401	0	2.088965	0	0	2.968703	0.935796	0	0	0	0	0	0	1.35509	0.924276	0	3.617639	0	0	2.069027	0	0	3.982337	0	0	0	0	0	0.626961	0	0	0	0	0	1.37396	0	1.203326	0	1.504553	0	0	1.703032	0	0	0.706894	0	0
LOC102724	0	0	0	0	0	0	0	0	0	0	0	1.505082	0	0	0	0	0	0	0	0	0	0	0	1.853078	0	0	0	0	0	0	0	0	1.755198	0	0	0	0	2.778659	0	0	0	0	0	0	0	0	0	0
B3GNTL1	6.036167	7.407404	6.83309	6.028457	7.503076	7.604286	8.180235	8.028953	7.258212	7.757664	6.406839	6.621108	7.703477	6.906464	6.48886	6.82294	7.542023	7.613275	6.830375	7.401491	5.76201	7.337422	6.588297	5.163338	6.835101	7.155468	7.356843	7.303932	6.216596	6.530504	6.299947	6.530841	7.898285	7.473702	6.382092	6.519372	6.985619	4.265871	7.074173	6.602225	6.850873	7.574581	6.511496	6.84716	6.860136	6.683669	6.248908	6.558516
DIRAS1	0.995829	1.262201	0	1.393533	3.636238	1.778203	0	0	0	3.190442	0	1.505082	0	1.913269	2.041335	1.884869	0.697815	0	1.042062	4.056113	1.646481	2.635241	0.588633	6.387768	1.174725	1.289558	1.876099	0	0	0.626961	1.956475	0	0	0	0	2.881125	1.046499	6.075482	1.137227	2.832801	1.582975	2.772849	0	0	1.297421	1.179238	0	2.165499
MAP3K8	6.121049	6.60048	6.30398	6.275734	6.107892	6.480419	5.539845	7.617433	6.306987	7.522781	8.078608	6.070017	6.485954	4.841277	6.087455	6.560166	6.460848	6.202818	5.725175	7.14681	4.946247	7.592489	8.145371	6.19544	6.702032	7.80386	6.499402	6.363625	7.166501	7.121578	6.508351	6.111671	7.365668	7.083919	6.683648	5.037216	5.374673	5.765817	6.727418	6.133554	7.403594	5.660888	7.391737	6.486699	5.779837	7.546061	5.353013	6.230396
SLC35B4	7.824573	6.971275	6.6867	7.720487	6.742594	7.312422	7.973679	7.244468	8.090023	7.3358	6.663425	7.06915	7.609088	8.292909	7.06006	7.140023	7.906082	7.766387	6.850376	7.24301	6.177482	7.383189	7.234912	6.62265	6.684501	7.184411	6.905099	6.578762	6.847081	7.691733	7.511838	6.111671	6.845928	7.083919	7.182015	6.388433	7.491901	5.521776	7.999494	7.324078	6.850873	7.552331	7.277514	8.006657	7.030693	7.230928	7.336797	7.286664
TCTA	7.892548	7.438756	9.095598	7.57418	6.406852	7.517662	7.150952	8.6371	8.325575	7.222578	6.986122	7.613762	6.684164	7.87516	7.845774	7.304721	6.851205	7.467717	9.075712	6.474833	8.602831	7.592489	7.935751	8.649666	7.35065	7.549551	7.569551	8.917075	7.720497	6.675835	6.815621	8.97745	7.982007	7.083919	7.229388	9.095373	7.533981	8.93328	6.694378	7.594782	8.134873	7.154698	6.886354	7.926149	7.62801	6.950275	7.347224	7.410082
NME5	0	0.950401	2.32209	0	0	0	0	0	0	4.414289	0	0	0	0	0	0	0	0	0	1.562827	0	0	0	3.721657	2.254743	0	0.737862	1.531951	0	0.626961	0.782644	0	0	0	0	1.37396	0	4.532683	0	0	0	0	0	0	0.789892	1.179238	0	0
RHOBTB3	9.546246	10.485	9.665537	8.211106	10.03148	8.707869	10.83122	8.569371	9.005954	8.494672	8.783247	8.209767	9.3971	9.864134	10.17245	10.07691	8.617977	11.19725	10.73259	9.035509	9.713261	8.588747	8.798861	3.721657	9.979444	8.471311	10.48706	9.747961	10.29273	8.428186	10.63017	11.05833	10.09831	8.720526	8.003958	8.926302	9.494428	3.029425	8.713052	8.990745	9.17776	9.401627	9.727399	8.749657	10.4749	9.548811	8.135818	9.541691
BIRC7	0	0	1.000101	0	0	2.084058	0	0	0	0	6.530952	0	0	0	0	0	0.697815	0	0	2.295325	0	3.619926	6.230005	0	1.813523	0	0	0	0	1.062548	1.287213	0	0	2.12653	0	0	0	0	0	0.68926	4.245102	0	0	0	0	0	0	0
KIRREL2	0	2.092015	4.170116	0.858862	3.108153	1.389562	0	0	1.275828	0	1.182979	1.505082	0	0	0	1.483087	0	0	0	0	0	0.902111	0	9.643163	2.592156	2.415109	0.737862	4.44688	0	2.912975	0	0	2.523927	0	1.775568	0	0	10.26226	1.137227	0	0	0	0	0.864812	2.609429	0	1.119477	0
TGFBI	12.59527	9.459506	10.72897	9.375234	11.4227	12.69303	7.611815	9.356632	12.27948	12.89323	12.27007	12.31883	8.807146	13.42427	8.914273	13.81824	11.2312	7.487994	10.62881	12.62243	11.3201	11.33875	12.29667	4.452965	13.43676	8.968114	9.364911	11.04066	9.258317	7.870142	14.50741	11.10313	8.743592	11.56076	8.902298	10.09405	13.63566	5.348102	10.90713	12.20924	11.23522	9.376519	10.87949	11.63644	14.14614	11.30428	11.74987	11.75518
IVD	9.453138	9.73441	9.346715	9.786187	10.29851	9.514943	10.15506	9.102449	9.896821	9.524867	8.799793	9.522471	9.902529	9.775831	9.843512	9.769338	9.677702	10.02347	9.62392	9.317659	10.02049	6.764619	9.691329	10.8028	9.861538	9.898437	9.844064	9.529018	9.760124	10.05901	9.866914	9.682113	8.703046	9.353438	9.79061	9.881652	9.854657	10.63862	9.791633	9.518648	6.348729	9.701177	9.672623	9.595551	9.912795	9.671336	9.453459	10.15387
TMEM54	8.726376	11.26266	10.66643	9.147892	10.08815	10.87728	9.952896	10.90002	9.171572	9.812569	9.597502	9.170981	9.996077	8.22783	11.34514	9.648671	8.654069	9.413371	11.2096	9.723264	10.68932	7.825833	9.571302	10.38943	10.9515	9.784904	11.39545	10.75662	10.63538	9.510338	9.337827	10.93754	8.888042	9.33851	10.23262	11.86273	8.694945	10.31927	8.449055	10.92732	8.240201	9.521004	10.30409	9.465868	10.22545	9.928844	8.873138	10.26329
DALRD3	6.910579	6.965905	7.426465	7.219908	7.709814	8.045752	7.433344	7.345106	7.386304	6.397825	7.46207	6.479676	7.636692	6.215588	6.861729	7.054919	5.436266	7.247532	6.958763	6.787146	7.859099	8.021823	7.37286	8.722861	7.316913	7.457696	6.913114	7.746646	6.921732	7.44369	7.257193	6.664377	7.926736	7.010478	7.016335	8.085381	6.240044	8.884405	5.82732	7.977892	7.881709	7.435625	6.966608	7.021403	7.440953	6.912914	6.670312	7.609965
PPP1R1A	0.995829	1.262201	2.32209	1.782752	0.709495	1.389562	1.711982	0	3.628893	2.340407	0	3.21301	0	0	0	0	0	0.701667	0	3.429162	1.046383	0	2.468424	7.105713	1.813523	1.959423	1.223673	3.386649	0	0	0	0	0	0	0	0	0	7.21145	0	1.504553	0	2.772849	0	2.099096	2.772216	0	3.20301	0
SESTD1	6.295533	6.6482	6.629556	5.916316	6.428293	6.974292	7.04304	4.628574	5.956968	6.681046	8.195759	7.078991	6.391893	7.395579	6.991053	5.975985	7.565901	7.22958	6.126104	7.333195	5.411035	7.190706	7.377239	6.446573	7.518356	6.106286	6.43406	6.975636	7.444835	6.349748	6.326086	5.017261	7.066527	6.786811	6.179908	4.63675	6.948763	5.730856	7.523057	6.740859	7.122883	6.419558	7.398183	6.512879	7.281109	7.711869	7.347224	6.610913
HOXA7	0	3.607497	1.585097	4.667222	5.506723	3.422226	4.702501	0	1.275828	0	4.326017	5.0519	0	5.036331	4.696556	6.321935	5.953277	5.014317	4.664532	0	0	2.991024	4.219845	0	3.622635	5.15659	3.275027	0	5.808011	4.672079	7.201801	4.549545	0	2.12653	2.819529	0	4.334798	0	5.796515	3.348635	1.582975	3.486777	6.535083	1.401742	6.39403	6.794646	6.133158	5.01685
RTN4	9.903047	10.67799	10.9832	10.05138	10.46726	9.471159	10.1186	8.673039	9.763979	9.993145	11.31673	10.34309	10.55999	10.2523	10.99367	10.6805	10.56338	9.880278	10.74733	9.807483	10.37665	10.16794	10.6081	8.861676	10.19288	9.021222	10.79204	11.03416	10.96155	10.12801	10.99545	10.71395	9.245102	9.988752	9.256868	9.859574	10.52924	8.059347	10.54774	10.07701	10.39541	10.7041	10.06781	9.936276	10.41684	9.691334	10.49291	10.73472
CPAMD8	0	2.241729	4.087653	2.088965	4.231623	2.550894	2.142139	0	0	0	0	0	5.749601	0	0	0	1.166132	0.701667	0.613139	0	2.885979	0.902111	0	3.143737	3.095078	3.039605	1.223673	2.258076	0	1.062548	0	0	0	1.424024	0	3.163346	0	5.459073	0	3.061685	0	5.110561	0	0	1.297421	0	1.119477	2.165499
FGL2	0	0.552086	0	0	0	0	0	0	0	0	4.23161	0	0	0	0	3.318652	0	0	1.042062	1.562827	0	2.161949	3.533593	0	1.813523	0	1.223673	0	2.830649	0	4.248461	0.923506	1.755198	0	0	0	0	0	1.137227	1.153748	0	2.426512	0	0	1.297421	2.261144	0	0
SFXN5	7.635544	7.986882	7.40108	8.444642	7.143497	7.213534	6.066242	9.02366	7.939444	6.988663	2.878187	7.711903	7.77974	7.805503	7.530285	8.316494	3.532913	5.486934	8.201583	7.617888	7.832375	7.668388	2.330721	8.264709	7.780078	8.082828	8.070114	7.542204	6.821314	8.450805	8.38271	8.318928	7.235256	7.473702	8.140331	8.563231	7.574867	8.009166	3.321471	7.630892	7.150168	7.721402	7.256383	7.881636	7.528995	6.858926	7.963524	7.475129
RHOXF1P1	0	0	0	0	0	0	0	0	0	0	0	0	0	0	0	0	0	0	0	0	0	0	0	2.638167	0	0	0	0	0	1.8959	0	0	0	0	0	0	0	2.089751	0	0	0	0	0	0	0	0	1.742164	0
GPR20	0	1.924946	1.000101	0.858862	0	0	1.096298	0	0	0	0	0	0	0	0	0	0.697815	2.046811	0.613139	0	1.646481	0.902111	0	3.143737	0	0	0	1.531951	0	0	1.287213	0	0	0	0	0	0	2.634778	0	0.68926	0	0	0	0	0.789892	0	0	0
SH3TC2	7.401086	4.968243	5.000195	3.533175	5.604141	7.046789	6.899146	5.26419	6.928573	7.389245	7.835486	6.9018	9.165621	4.137479	5.075565	7.271576	6.686718	6.239161	5.681182	7.000349	5.612958	5.929485	6.905709	1.853078	6.496372	4.19648	4.683977	5.41342	6.049094	1.667771	7.336486	3.853076	5.858415	5.249189	4.789782	5.233107	3.080416	1.203326	7.299619	6.749097	6.276279	9.34774	6.132972	5.697895	7.680217	6.473912	6.436392	5.360767
LINC01783	0.995829	0.950401	0	0	1.182986	0	0	0	0	0	0	0	0	0	0	0	0	0	1.372257	0	0	0	0	2.420752	0	0	1.876099	0	0	0	0	0	0	0	0	0	0	2.778659	0	0.68926	0	0	0	0	0	0	0	0
WDR17	0	1.518444	0	0	0	0	0	0	0	0	0	4.206711	0	0	0	2.864755	6.501035	0	0	0	1.046383	4.662567	0	0	1.813523	0	0.737862	0	0	0	4.498789	0	0	2.12653	0	0	0	0	6.335782	0	3.541594	2.426512	0	0	2.215594	0	4.611978	0
COL4A1	3.69273	0.950401	2.000151	0.858862	0	2.73768	1.096298	0	4.056163	0	6.067455	3.473948	0	0	1.35509	7.254713	0.697815	2.046811	0	0	1.046383	8.962379	0	0	1.813523	0	2.504345	2.738832	0	2.560415	8.047794	0	0	2.12653	0	0	1.046499	0.723645	0	3.432866	8.984761	1.297861	0	2.567208	1.672125	0	3.796962	0.904236
DYNC2LI1	6.746611	5.900668	6.475933	6.670829	6.801291	7.115816	6.137939	6.74014	6.332657	6.622738	7.354185	6.387644	6.630256	6.392291	6.243941	6.150216	6.252997	6.853348	6.200713	6.761388	5.996736	7.60542	6.98244	5.091455	6.476136	6.88273	6.0097	6.110688	6.945781	6.960674	6.564325	6.612436	6.96044	6.952835	5.795296	6.150553	6.240044	5.730856	6.258772	7.110511	7.437212	7.435625	6.425763	6.486699	6.041685	7.168889	6.376564	6.504144
BORCS5	6.313679	5.360842	6.930937	6.923788	6.067468	5.94836	6.066242	6.597818	6.254235	6.326553	5.088808	5.902774	4.87219	6.820791	6.861729	5.491495	5.283359	6.30921	6.725982	6.270876	6.447794	6.121741	4.258335	7.37156	5.939224	6.611522	5.211633	6.790804	6.2556	6.652607	6.008647	6.111671	5.798095	5.98375	6.666539	6.985876	7.135269	7.230347	5.649118	6.333053	6.636086	5.335687	7.461102	6.333548	5.907962	7.486269	6.314148	6.144134
FKBP14	6.719646	7.741841	7.149947	7.196091	7.403748	7.949761	6.912837	7.834935	6.691244	7.423805	7.162799	6.786404	7.742112	7.713418	7.612989	7.467242	7.607909	6.884276	6.581887	7.14681	7.021333	7.491632	6.055212	5.477192	7.614063	7.08046	7.686393	7.158685	7.951842	7.479332	7.616153	7.253096	8.09913	7.314995	6.798045	6.771398	7.483336	5.748442	7.944353	7.409712	7.727218	7.691173	6.625756	6.92721	7.030693	7.136839	7.09836	7.060438
TJP2	10.82612	9.722518	11.84452	10.5465	10.3387	10.00573	9.268394	8.71125	10.39253	10.00175	10.56304	10.55801	8.686868	10.38359	9.359691	10.24749	10.4595	10.07297	10.64321	10.25556	10.08417	10.40271	9.769194	7.556457	10.74397	10.56085	9.989502	11.63957	10.31829	10.59865	11.03313	10.6348	10.32364	10.5467	10.57394	9.66397	9.952538	7.780398	10.45954	10.4479	10.09987	8.814619	10.80383	9.930218	11.04242	10.29036	10.69012	10.14945
CDHR5	7.553933	6.367822	8.644057	7.917835	5.192705	9.218047	6.373993	8.910521	7.988714	6.362629	5.830353	7.627239	1.528973	6.803029	7.601461	8.198005	2.722119	6.861143	9.357762	6.761388	8.124406	3.514195	5.076746	8.731755	10.07336	9.835963	6.612012	8.493597	7.339854	5.792617	8.572087	11.07402	8.160311	9.446873	9.204488	9.026584	6.299801	11.84721	1.999619	8.718173	3.904107	2.772849	6.450783	8.19225	9.407097	7.149744	7.594847	5.533588
KRT15	6.546324	5.911883	5.954395	6.06398	5.217548	8.668204	5.608757	7.107916	5.889388	5.804005	9.787843	5.011358	10.8745	5.88506	8.918925	7.140023	6.299313	6.074604	6.46567	6.566929	4.61103	2.824072	7.016523	0.903733	6.666754	4.407587	5.994671	5.536127	8.900527	5.459443	7.187614	7.04353	4.761976	7.579061	5.763465	5.105511	6.260239	0	6.889457	8.379167	5.330992	10.31889	6.265788	5.394209	7.307904	7.084031	3.375938	4.80829
TMEM241	6.664154	6.788661	6.629556	6.854476	6.259132	6.675949	5.674527	6.137172	6.357878	6.212573	6.094529	6.418978	6.455277	7.258932	5.754258	6.460742	7.512838	6.274611	6.863559	6.11689	5.704224	7.102251	6.377173	6.032247	6.303139	7.017514	6.59218	6.45978	5.808011	7.345712	6.326086	6.444353	6.655073	6.468146	6.360977	4.965526	7.614627	5.676772	7.470358	6.407803	6.606842	6.058291	5.898497	6.88774	5.798852	6.087936	6.530902	6.745903
ORMDL2	7.046992	7.910668	7.066289	7.903199	7.56246	7.432284	8.213804	8.400354	7.386304	7.538636	7.213078	6.977414	6.29127	6.484327	7.835969	6.587358	5.717808	7.149099	7.786449	6.50619	7.622224	6.764619	7.759125	8.592225	7.586012	6.165552	7.88691	7.251163	7.015613	6.75794	6.7104	7.543966	7.189027	6.742138	7.414989	8.23007	7.167705	8.633069	6.116364	7.529328	7.491556	6.317713	6.003684	7.40366	8.159961	5.823779	6.914249	7.768296
SPHK2	8.349224	8.364897	9.32213	8.257871	7.077177	9.058444	8.050003	7.261736	9.05304	8.706	7.028856	8.878704	7.228611	7.205849	7.480806	8.376182	7.213589	7.008789	7.899558	8.50979	8.7741	7.027363	8.135065	9.353533	8.242219	8.435642	8.390251	9.155885	7.060358	8.049791	8.312537	7.998129	7.647898	7.34518	8.74412	9.430983	7.683544	9.219975	6.994981	8.697038	7.652861	6.26396	8.057654	8.78768	8.936999	7.763158	9.13626	7.762517
TCEAL9	9.279372	7.949278	8.771691	5.323843	7.76141	5.967161	6.899146	7.745703	8.881041	8.017325	0	7.850505	8.782645	1.252965	5.835235	7.07427	0	6.262891	5.168022	8.446543	8.500452	2.635241	0	8.268789	9.094748	3.474011	7.989021	8.684795	6.921732	7.05227	6.376986	4.802275	7.869262	10.76058	2.547809	8.634306	0	8.187361	0	6.546599	2.113183	9.059084	0	9.280199	8.242143	0	7.970286	7.721403
ADGRE1	1.993739	0	0	0	1.538883	0	0	0	2.741038	0	0	1.909816	0	0	0	0	0	0	0	0	0	0	0.588633	6.051318	0	0	0	0	0	0	0	0	0	3.235214	0	0	0	4.360398	0	0	0	1.969853	0	1.79215	0	0	0	0
DNAH14	7.313627	6.806797	6.108723	6.923788	7.279483	6.440612	6.75458	0.935796	6.928573	6.465742	2.604499	7.219089	2.734961	4.531876	2.854499	7.015422	2.971314	7.317186	2.38865	6.156955	7.508656	0.902111	2.468424	7.41638	6.897299	7.582543	6.60213	5.851823	3.932237	6.915853	6.575264	3.760654	0	7.735384	6.463593	7.524018	5.613179	8.158053	2.378102	5.778633	1.582975	3.663245	6.069776	7.209232	6.502399	7.117262	6.751063	6.901245
DSEL	1.579399	0.950401	1.000101	0.858862	0	0	0	0	0	0	0	0.940508	0	1.252965	0	0	1.166132	0	2.527635	0	0	0	0	4.452965	0	1.289558	0.737862	0	0	0.626961	0	2.863351	0	0	0	0	0	5.096718	0	0	0	1.297861	0	1.79215	0	0	0	0.904236
CPM	0.995829	0.552086	0	1.782752	0	3.527314	1.096298	2.217783	0	0	1.182979	0.940508	0	0	5.577246	0	1.166132	2.046811	1.640777	0	2.069027	3.140645	1.328528	9.328346	2.254743	0	1.586456	1.531951	6.049094	2.266084	0	1.482042	0	2.12653	2.212753	2.064752	0	9.024521	2.201237	3.512449	3.904107	0	0.80896	0.864812	0.789892	1.819318	1.742164	0
TAC3	0	0.552086	1.000101	1.393533	2.997752	2.550894	3.873483	3.20422	0	2.827144	0	1.909816	0	0	0	2.864755	0	5.761353	2.527635	2.778832	1.046383	2.635241	0	12.04618	3.467277	5.15659	0.737862	0	2.343681	0.626961	2.411883	5.204327	5.285832	0	1.775568	2.064752	0	11.58165	1.765176	1.504553	3.62175	0	0	2.099096	2.215594	1.179238	2.1757	2.995019
TOP2A	11.6418	10.57638	11.48905	10.68485	10.59422	9.487531	10.76577	10.00827	10.70537	11.16068	11.7861	11.23331	10.6148	11.71351	10.04632	11.11589	11.25636	10.86795	11.11975	10.98558	10.85282	11.22134	11.64216	10.54806	10.59944	10.09607	10.45448	11.37766	9.729281	10.71814	11.61157	10.47401	10.4417	10.72515	10.33634	10.33357	11.28072	9.350941	11.49665	10.7024	11.16423	10.50562	11.51549	10.64505	11.00658	10.8983	11.45493	10.83794
PLPP2	9.58971	9.967911	10.33894	9.353866	10.75186	10.81248	8.525878	9.570938	10.04975	10.99387	11.00247	10.13359	10.29354	10.11148	10.1918	10.29362	11.1768	8.416808	10.80953	10.87883	10.62751	8.926764	11.52323	5.015801	10.88974	8.578775	10.12507	10.72633	9.199521	8.803448	9.932186	10.71395	9.025926	10.60243	8.934815	10.98313	10.64608	4.686572	11.16327	10.42687	9.301564	10.16793	10.31691	10.18855	10.525	10.35307	9.864356	10.95594
NR3C1	0.995829	5.758818	3.32211	4.415499	0	0	0	0	1.942141	0	0	0	0	0	1.35509	0	0.697815	0	0	0	1.046383	0	0	5.686843	6.025303	2.415109	5.556781	2.738832	1.603113	3.279313	0	1.482042	1.755198	0	0	3.937937	0	6.820589	0	0.68926	1.872301	3.486777	0	0	4.842975	1.179238	0	1.85386
CPD	9.928293	9.534885	9.881316	8.703049	9.236491	8.484654	9.241522	8.442609	8.973687	8.87788	10.27132	9.128963	8.25084	10.08783	8.900227	8.929467	10.57225	9.908761	9.957827	9.088262	9.202297	8.866276	10.05537	8.202091	8.187263	7.697441	9.545913	9.803991	9.544773	8.948786	10.07583	10.01154	9.090222	8.703151	7.561012	7.806411	9.505045	8.107842	10.98006	8.840793	8.96569	8.952681	9.892862	8.399393	8.64026	9.545152	9.578215	9.0758
C11orf74	5.079355	4.684193	4.907086	6.96195	5.968493	6.544416	5.855558	6.677183	5.246249	5.75055	5.506709	6.464739	6.684164	4.694855	5.623576	6.867987	4.826295	5.486934	2.527635	6.075681	3.66889	6.875521	6.010812	4.133161	4.841216	6.84697	5.53617	5.649211	4.980469	7.010248	5.942599	3.555893	7.116782	6.719271	6.000381	2.881125	4.731851	4.532683	5.035132	5.974026	6.842533	6.467906	4.957818	6.111689	4.688413	5.52018	6.356057	5.782312
IGF2R	10.74487	9.879278	10.76673	9.66996	10.48974	11.06898	9.268394	8.112228	10.23811	9.175306	10.43517	10.53942	9.620916	11.00895	9.951887	10.0936	10.48665	9.493283	9.871559	9.46057	9.744056	9.693653	10.54577	8.248275	10.02717	9.709746	9.873116	10.51318	10.49167	9.222944	11.47624	9.630819	9.359092	9.764205	9.293549	9.132673	10.81601	8.567705	10.84121	11.06814	9.723239	9.97023	10.82611	9.950712	10.7669	10.30413	11.07754	10.56167
FBLL1	0	0	0	0	0	0	2.473063	6.099252	1.275828	0	0	0	2.254475	4.531876	3.37139	0	0.697815	2.046811	2.062641	0	3.784938	1.452909	1.005502	3.813884	1.174725	0	1.586456	0	2.343681	0	0	1.482042	5.195186	0	0	4.63675	3.251582	3.810133	1.765176	0	1.220689	1.297861	0.80896	1.401742	1.672125	0	0	2.165499
FKBP4	10.3904	11.45725	11.15312	11.27947	10.60426	10.13606	11.47882	11.57902	10.69304	10.241	10.68273	11.35474	10.09641	11.58103	11.07407	10.0829	10.86324	10.22266	11.64801	10.00425	11.67438	10.17012	11.1639	11.8851	10.2946	10.85106	11.28127	10.78628	10.52796	11.18482	10.39421	11.34711	10.39918	10.25831	11.5579	12.28613	11.4419	11.05549	10.8581	10.92551	10.28358	9.966028	10.77626	11.0343	10.97662	10.24071	11.46454	11.26066
MSH2	9.394671	9.294724	8.879785	10.3016	9.872415	7.826477	9.398161	8.235339	8.932311	9.003156	9.215049	9.193805	8.591349	9.759806	9.267868	8.763559	8.988592	9.493283	9.047111	9.183471	9.513257	10.02007	9.384919	6.846593	8.168469	8.386668	9.147744	8.658019	8.900527	9.945733	9.13468	8.22693	8.711247	7.96655	8.913218	8.605116	9.630586	6.2085	9.064506	8.20147	10.18478	8.635719	8.600854	9.125487	8.92839	8.163867	9.371544	10.01008
TTC37	9.637315	9.029792	9.301698	8.150433	9.459844	10.0114	8.844346	8.332514	8.863537	9.170226	9.355973	9.002805	8.75142	9.639857	7.806149	8.658502	8.03485	9.4213	9.609322	9.496144	8.426437	9.075893	8.893603	7.202724	9.242832	9.3175	8.916122	8.847681	8.75159	9.360706	9.360136	9.770677	9.503024	8.98299	8.510009	7.39352	9.105396	7.710337	8.289513	9.534224	8.424688	9.13216	8.698091	8.558669	9.077937	9.062449	9.246211	8.790765
DUSP4	7.606399	7.938352	7.85195	8.497563	7.255692	8.500874	6.627499	5.599114	7.434517	6.681046	12.09512	6.387644	10.644	7.732311	9.126217	6.900874	12.05521	7.41574	6.376525	6.787146	6.020607	3.514195	11.81773	4.133161	6.648787	7.321013	7.824536	8.16318	9.65464	7.560611	7.7233	6.612436	5.858415	7.995387	6.904034	5.942252	7.637967	3.593705	12.24011	8.722363	4.602002	10.44954	9.034402	7.876604	7.260681	9.047011	5.980761	7.523768
MCCC1	8.442881	8.510327	8.092958	8.674605	9.070847	7.663123	7.851042	8.408129	8.260196	7.716438	8.002031	8.292456	7.46254	7.933468	8.544552	6.82294	8.425204	8.312655	8.733783	7.891619	9.048245	7.704891	8.254121	9.34583	7.293975	9.021222	8.316628	7.844886	8.600717	8.412139	7.208842	8.83174	8.783029	7.153802	8.956092	8.674204	7.992043	8.915875	8.322324	7.576382	7.873547	7.817819	8.416301	7.438222	7.590308	8.265187	8.273402	9.082774
CEP135	5.664236	5.733759	6.129482	6.06398	6.147214	5.096339	7.055436	4.872893	6.021524	6.561974	7.57211	6.306195	6.736131	6.484327	6.822545	6.653178	7.290124	7.399775	6.674553	6.978157	5.04266	6.764619	8.165763	6.357441	5.275191	5.91231	6.243797	6.363625	6.626776	6.074797	6.161637	6.166732	6.512189	5.537603	5.399286	4.080496	6.156312	6.420809	7.631763	4.740019	5.958997	6.514686	6.413089	6.194895	5.81762	6.074463	6.567037	6.089787
BEX3	9.934169	6.938749	9.370889	5.8364	9.190176	1.389562	8.30488	9.479801	10.01648	8.445059	0	9.27525	9.010735	1.252965	0	1.483087	0	7.963902	7.962173	8.577779	8.280843	5.6338	0	9.876257	8.529223	2.760887	7.01345	9.514165	1.603113	7.691733	3.269538	7.860178	8.361287	9.819005	0	8.645818	1.646635	9.585506	0	2.02229	5.467405	8.905189	0.80896	10.35155	8.671547	0.706894	9.235024	9.004146
MRPL23-AS	2.800198	0	2.000151	3.314649	4.757263	5.632261	7.017922	1.902589	2.396196	4.414289	6.294575	6.272283	3.382531	4.246636	2.041335	6.504194	2.242534	9.026591	2.527635	4.491695	0	6.59347	5.314487	0	4.962547	7.421685	0	3.386649	5.45278	5.940595	5.781912	3.939932	5.285832	4.286632	4.725149	0	4.995478	2.089751	1.999619	4.982893	5.669501	4.091281	6.992397	0.864812	2.609429	7.940722	5.54642	1.85386
SDR42E1	6.515299	6.713746	7.229019	6.980658	4.687846	5.632261	0	6.74014	6.083315	6.868353	0	6.371718	0	1.252965	6.330284	0	0	0	5.189118	6.075681	6.644968	5.473783	0.588633	6.981963	6.232515	6.012556	6.822399	7.119967	5.699038	6.359344	0	5.238996	6.025574	5.98375	6.73379	6.388433	0	6.924808	0	5.625973	6.119457	1.297861	1.703032	6.094456	7.022627	1.819318	6.81256	4.334438
MIR4489	0	0.552086	0	0.858862	0.709495	0	0	0.935796	0	1.600378	0	0	0	0	0	0	0	0	0	0	1.646481	0	1.005502	0	0	0	1.223673	0	0	0	0	0	0	0	0	1.37396	0	1.562693	0	0	0	0	0	0	0.789892	1.179238	0	0
OLIG1	0	0	0	0	0	0	0	0	0	0	0	0.940508	0	0	0	0	0	0	0	0	0	0	0	3.721657	0	0	0	0	0	0	0	0	0	0	0	0	0	0.723645	0	0	0	0	0	0	0	0	1.119477	0
UGT1A10	7.830887	9.283975	8.607532	7.171875	7.200685	5.509372	4.880261	7.41623	8.104997	4.414289	0	8.60327	7.792067	7.75096	6.822545	2.456453	6.501035	4.162882	9.167592	4.491695	8.798469	1.850557	0.588633	6.647928	7.415845	3.807436	9.197814	8.560185	8.695959	7.25094	4.134752	10.22006	9.450105	6.05853	4.91096	8.252835	7.250798	6.87764	6.608307	5.916674	1.582975	8.45563	1.324175	6.90761	8.238665	1.534489	8.953824	7.516919
PRSS22	6.499533	6.249046	8.704105	7.212013	5.115502	8.326197	2.473063	7.154884	6.523063	6.432183	10.48718	4.789568	9.343666	3.592931	5.262852	7.781665	7.231615	4.306808	7.708232	7.16657	5.922658	5.989898	9.059449	1.455124	6.666754	8.399069	6.218147	8.691412	7.444835	6.889602	8.193347	8.196383	6.435064	8.847211	7.4454	6.519372	4.843145	1.850158	7.326824	7.420068	5.611788	9.910259	5.479392	6.857413	5.411084	7.168889	4.97681	4.937006
HAUS6	7.621045	6.911073	7.925013	7.720487	6.317751	7.872082	7.534222	6.409238	7.146863	7.730311	9.051965	8.083458	6.927083	8.20772	7.518074	7.510501	7.598679	7.571128	7.121238	7.972424	7.483112	8.494585	8.762502	7.219682	7.415845	7.155468	7.005975	7.796602	7.761724	7.909867	7.517521	6.488245	7.040728	7.403719	7.484976	6.182523	7.763537	6.485161	7.358806	7.585611	8.114238	7.639342	7.907608	7.485259	7.680217	8.331818	7.990384	6.780628
MIR7-3HG	0	0	0	0	0	0.855986	0	0	0	0	0	1.505082	0	0	0	0	0	0	0	0	0	0	0	5.84821	1.174725	3.273097	0	0	0	0	0	0	1.755198	2.596932	4.349777	0	0	5.676772	0	0	0	0	0.80896	3.326555	0.789892	0	1.742164	0
HS6ST3	0	0.552086	0	0	0	0	0	0	0	0	0	0	0	0	0	0	0	0	1.867074	0	1.046383	0	0	1.853078	0	0	0.737862	0	0	0	0	2.197512	0	0	0	0	0	1.850158	0	0	0	0	0	0	0	0	1.742164	0
DOCK4	5.161804	3.764211	3.585147	3.967622	4.53811	6.004044	4.499719	2.217783	5.074321	3.480412	5.65957	3.97336	3.622323	3.217207	3.751148	5.95526	6.19293	4.595514	1.867074	2.778832	0	5.386587	5.436993	3.62313	2.592156	1.289558	3.971724	3.626509	4.884194	3.868658	7.076519	0.923506	5.669318	3.676189	0	2.064752	2.661649	3.875617	6.314195	6.37624	3.904107	3.486777	7.378759	4.428113	4.090583	6.7614	3.006495	4.334438
CLEC18A	0	0	0	2.341426	0.709495	0	1.096298	1.498706	0	0	0	0.940508	0	1.252965	0	0	2.242534	0	1.372257	1.562827	2.069027	0.902111	0.588633	0.903733	1.174725	0	0	0	0	0.626961	0	2.197512	0	0	1.145263	2.529929	0	3.029425	0	0.68926	0	1.297861	1.324175	0	0	0.706894	0	1.85386
GALNT14	0	0	1.000101	0	0	0	0	0	1.942141	0	0	2.484401	0	0	0	0	0	0	0	0	0	1.452909	0	6.01292	0	0	0.737862	0	0	0	0	0	0	0	0	0	0	4.164713	0	0	0	2.426512	0	0	0	0	3.20301	0
PDGFD	0	0	0	0	0	0	0	0	0	0	0	2.484401	0	0	0	0	0	0	0	0	0	0	0	2.638167	0	0	0	0	0	0	0	0	0	0	0	0	0	3.669513	0	0	0	0	0	0	0	0	2.778926	0
P2RY4	1.579399	0	1.585097	0	0.709495	0	0	0	1.275828	0	0	0	0	0	0	0	0	0	0.613139	0	1.646481	0	1.005502	2.164658	1.174725	0	0	1.531951	0	0.626961	0	1.482042	0	0	0	0	0	2.089751	0	0	0	0	0.80896	0	0	0	0	1.85386
PRPH2	0.995829	0	1.000101	0	0.709495	0	0	0.935796	0	0	0	0	2.254475	0	0	0	0	0	0	1.562827	1.046383	0	0	3.143737	1.174725	0	0	0	0	0	1.660309	1.482042	0	1.424024	1.145263	0	0	4.265871	0	0	0.735773	1.969853	0	0.864812	1.672125	0	1.119477	0
SLC6A20	4.451459	4.809408	6.870565	6.923788	5.265986	3.716984	6.926399	3.877173	5.191185	2.827144	6.608133	7.493813	0	1.252965	9.307431	7.496225	3.608434	6.520824	5.964208	3.140335	2.885979	9.198294	6.296245	0	9.771	5.56148	5.53617	6.815245	10.25954	7.641492	7.757317	6.270924	6.875419	8.939135	5.075546	0	1.046499	3.029425	3.137054	4.866561	9.200778	1.969853	5.183548	8.832544	8.214086	4.570602	7.79855	5.87092
LRRN2	1.579399	0	1.000101	0	1.538883	1.778203	0	0	0	0	0	2.894221	0	0	0	0	0	0	1.640777	0	0	1.850557	0.588633	4.93596	3.762878	0	0	0	1.603113	0.626961	0.782644	1.883682	0	2.596932	2.212753	1.37396	1.046499	6.364899	0	0.68926	0	0	1.324175	1.401742	2.215594	2.872407	2.778926	0.904236
PRAME	7.313627	6.514812	8.29022	0	6.896672	0.855986	0	1.902589	7.146863	0	0	8.068715	0	0	0	2.675001	0	1.809634	2.062641	0	7.385378	6.457459	0	10.30852	3.467277	0	6.698027	8.264283	0	1.667771	0.782644	2.863351	3.93115	8.978183	0	7.732749	0	10.08932	1.137227	0	5.098642	0	0.80896	8.395881	1.969301	0	7.872606	5.849273
ZBTB42	6.546324	6.894209	6.6867	6.760169	7.225392	6.238779	5.310842	6.155765	7.010377	6.131254	6.986122	5.763247	6.545418	6.268321	6.41175	5.729782	5.188109	6.019948	7.5378	6.625214	6.177482	5.08807	7.490702	8.316863	6.835101	6.303867	6.805275	7.233136	6.330581	6.728621	6.473694	7.082233	6.267222	5.18388	6.22735	6.771398	6.177705	8.83793	5.08697	6.690419	5.257623	6.394763	4.922482	6.178633	7.054622	5.671027	6.60229	7.294695

Gene	logFC	AveExpr	t	P.Value	adj.P.Val	B
NPY1R	-2.8308465	0.35534252	-9.0514428	5.43E-12	9.05E-08	16.3992157
ADAM23	-3.69829	0.55695958	-8.9251651	8.36E-12	9.05E-08	16.01761
FOXD3-AS1	-3.3572608	0.5380456	-7.4170589	1.60E-09	1.15E-05	11.3139419
ADORA1	5.11210713	1.5179653	6.98030002	7.50E-09	3.02E-05	9.91360934
S1PR3	-2.1480583	0.4497599	-6.9489188	8.38E-09	3.02E-05	9.81259937
HOXA3	4.57443995	6.96897532	6.84053883	1.23E-08	3.81E-05	9.46343855
ADD2	-4.5741629	1.06792712	-6.780912	1.52E-08	4.12E-05	9.27116381
NIPAL4	-3.2561997	0.64570034	-6.7153688	1.92E-08	4.62E-05	9.05968831
ARHGAP18	3.03439463	7.82512322	6.40248033	5.83E-08	0.00012617	8.04908709
OR2W3	-3.5999987	0.64516224	-6.2924272	8.61E-08	0.00016946	7.69353019
KC6	2.74273904	0.48968207	6.22208382	1.10E-07	0.0001993	7.46633516
RAB39A	-2.8558271	0.35584973	-6.0654656	1.92E-07	0.00029734	6.96087408
TRIM58	-5.8399919	1.3562472	-6.0103914	2.34E-07	0.00033714	6.78331173
CYP4F8	-6.3993226	1.93781528	-5.8864939	3.62E-07	0.00048934	6.38433306
FOXQ1	5.19789517	7.81570074	5.82106508	4.55E-07	0.00057989	6.1739566
APBA2	-4.3647126	0.73941284	-5.767635	5.50E-07	0.00066089	6.00235253
RIPPLY2	-3.4000544	0.61717734	-5.7067367	6.81E-07	0.00073095	5.80699716
HOXA2	4.79913498	4.3546396	5.69504725	7.09E-07	0.00073095	5.76952937
CACNG2	-3.0845617	0.25704681	-5.6398526	8.61E-07	0.00084669	5.59275839
NUDT16	-1.4455434	8.68354024	-5.6074694	9.64E-07	0.00090713	5.48916081
NKX2-1-AS1	-3.204668	0.29161915	-5.5569538	1.15E-06	0.00103734	5.32773719
IFNGR1	1.68688118	8.52857097	5.49920899	1.41E-06	0.00121833	5.14350269
PERP	2.45283453	10.5855639	5.44236445	1.72E-06	0.00142817	4.96246502
PTPRM	4.82880485	1.8294013	5.39823271	2.00E-06	0.00160355	4.82215205
SLC66A1L	-1.5015991	0.24819443	-5.3840043	2.10E-06	0.00162468	4.77696033
UNC5D	-1.8728865	0.22851607	-5.3711046	2.20E-06	0.00164056	4.73600883
INA	-3.3009206	0.34066646	-5.3205732	2.62E-06	0.00183113	4.57578075
IRX6	-1.4072127	0.14183121	-5.3202183	2.62E-06	0.00183113	4.57465653
FLJ40288	-1.7515967	0.17300516	-5.2569452	3.26E-06	0.00220841	4.37447393
NKX2-8	-3.5132066	0.41111463	-5.2456236	3.40E-06	0.00222698	4.33870958
NPR1	-3.4861969	0.80815271	-5.229863	3.59E-06	0.00224988	4.28895096
YTHDC2	4.04501021	7.65972754	5.22096899	3.70E-06	0.00224988	4.26088603
PRR16	-3.706092	0.40694174	-5.2174761	3.74E-06	0.00224988	4.24986714
CREBL2	-1.4590121	7.46033928	-5.1400299	4.89E-06	0.00278363	4.0059931
PRIMA1	-1.401782	0.13159631	-5.1016557	5.58E-06	0.00309475	3.8854798
CNNM1	-3.6378658	0.47137277	-5.0935283	5.73E-06	0.00310279	3.85998454
SIX3-AS1	-1.2652998	0.1977249	-5.0834403	5.94E-06	0.00313378	3.82835333
APBB2	2.06098669	8.2578706	5.04547449	6.76E-06	0.00348448	3.70945426
RAB8B	2.84775987	5.24356305	5.0123116	7.57E-06	0.00381256	3.60578713
MIR4787	-1.0370603	0.10525995	-4.99696	7.98E-06	0.00392668	3.55785986
ITGA7	-2.9762491	6.11268209	-4.9800961	8.46E-06	0.00400871	3.50525713
ZYG11A	-2.7702733	0.34971101	-4.9778937	8.52E-06	0.00400871	3.49839068
ANXA3	5.12890261	7.9828304	4.95331358	9.26E-06	0.00426664	3.42181613
SCARNA11	-1.0879162	0.15085925	-4.9410155	9.66E-06	0.0043566	3.38354345
C5orf63	3.16615249	5.92239792	4.93337941	9.92E-06	0.00438017	3.35979276
PLCXD3	-1.4979732	0.22116524	-4.9039466	1.10E-05	0.00447953	3.26834532
FZD10	7.39981787	3.08087884	4.89343225	1.14E-05	0.004484	3.23571559
LINC00867	-1.7329863	0.43598625	-4.8920099	1.14E-05	0.004484	3.23130318
PAQR9	-1.1276259	0.12893486	-4.8872392	1.16E-05	0.004484	3.21650576
NDST3	-1.3260062	0.2235594	-4.8791622	1.19E-05	0.00450095	3.1914633
LAMC2	3.75429723	8.07829934	4.84847663	1.32E-05	0.00469523	3.09643476
RNF150	-1.4341378	0.1790521	-4.8411299	1.36E-05	0.0046975	3.07370952
IL17RE	-1.9970411	7.74596668	-4.8388146	1.37E-05	0.0046975	3.06655
SEPTIN8	1.08981128	9.57134295	4.82670212	1.42E-05	0.00481775	3.0291116
HOXA5	5.59873836	5.65879064	4.81849456	1.46E-05	0.00487727	3.0037591
OOEP	2.43722884	0.44630526	4.76099075	1.78E-05	0.0055631	2.82651003
NKX2-1	-4.7893004	0.63647194	-4.7604276	1.78E-05	0.0055631	2.82477758
POLK	1.5863908	6.44946851	4.75773449	1.80E-05	0.0055631	2.81649298
BVES-AS1	-1.0636023	0.12472971	-4.757608	1.80E-05	0.0055631	2.81610397
CHST2	-2.7258796	0.39125134	-4.743169	1.89E-05	0.00575826	2.771713
PTGER3	-1.8219445	0.23241492	-4.7345532	1.94E-05	0.00584547	2.74524521
PAK3	-1.8789164	0.27150871	-4.7142647	2.08E-05	0.00603919	2.68298069
PHF21B	-3.0419848	0.44092831	-4.7127309	2.09E-05	0.00603919	2.67827703
IL24	-2.0510825	0.72295216	-4.6995699	2.19E-05	0.00607139	2.63793766
WSCD2	-3.8591744	0.62816703	-4.6994794	2.19E-05	0.00607139	2.63766065
RNF180	-1.675671	0.21342119	-4.6838313	2.31E-05	0.00631794	2.58974696
LCK	6.63418441	4.75975518	4.67025937	2.41E-05	0.00638293	2.54823341
SUOX	-1.8423017	8.41391029	-4.6623664	2.48E-05	0.00638293	2.52410913
POGLUT2	2.21790342	5.73977115	4.66034646	2.49E-05	0.00638293	2.51793768
TBC1D16	1.37663385	9.91201125	4.65991605	2.50E-05	0.00638293	2.51662277
GOLGA6L7	-1.4772502	0.15744552	-4.6589501	2.51E-05	0.00638293	2.51367193
NOVA1	-2.8113521	0.3838701	-4.6468748	2.61E-05	0.00647822	2.47680071
GASK1A	-3.2680597	0.93651131	-4.6441687	2.63E-05	0.00647822	2.46854236
ALDH1A1	6.16555299	9.89117186	4.63837098	2.69E-05	0.00653093	2.45085432
NKX6-1	-1.4014317	0.19784831	-4.6100051	2.95E-05	0.0068717	2.36442439
GRID1	-1.8347955	0.37593356	-4.598822	3.06E-05	0.00702766	2.33040043
FCMR	-3.5711295	2.20764232	-4.5969083	3.08E-05	0.00702766	2.32458093
PRR19	-3.2566384	4.03545179	-4.562381	3.46E-05	0.00772441	2.21973317
MAP3K11	-1.0834493	9.84857997	-4.5622918	3.46E-05	0.00772441	2.21946265
SCN4A	-3.4916949	1.10696003	-4.5448586	3.67E-05	0.00810209	2.16663147
RPL39L	-6.4672393	2.28590624	-4.527159	3.89E-05	0.00850602	2.11306777
ARHGEF38	2.15107741	5.76392025	4.51205068	4.09E-05	0.0088224	2.0674063
SLC39A4	-3.1934741	8.9959626	-4.5101257	4.12E-05	0.0088224	2.06159238
KCP	-4.2616955	3.19591949	-4.504567	4.19E-05	0.0088984	2.04480949
HBQ1	-3.5879611	1.31532871	-4.4941141	4.34E-05	0.00912257	2.01327034
CAVIN1	3.80442294	6.78127611	4.47517121	4.62E-05	0.00961946	1.95618398
OLFM1	-3.8286805	0.77115651	-4.4615023	4.84E-05	0.00994337	1.91504773
PPFIA3	-2.2545804	8.36895589	-4.4535165	4.96E-05	0.00994337	1.89103666
STAMBPL1	2.17016716	7.19237229	4.45333501	4.97E-05	0.00994337	1.89049101
NXPE1	-3.4057728	0.66218364	-4.4527772	4.98E-05	0.00994337	1.88881456
TCERG1L	-2.0343968	0.39325924	-4.4509395	5.01E-05	0.00994337	1.88329179
ERCC6L2	1.124768	6.79175335	4.4349051	5.28E-05	0.01016093	1.83514073
CD6	-3.0827909	1.94830827	-4.4327249	5.32E-05	0.01016093	1.82859879
B4GALT1	1.61786979	9.07858298	4.4277763	5.40E-05	0.01016093	1.8137545
SMOC1	7.54411875	4.17864604	4.4275006	5.41E-05	0.01016093	1.81292768
SLC2A1	2.66962578	10.8095518	4.42489897	5.46E-05	0.01016093	1.80512633
SMOX	2.61295575	7.17379319	4.42290058	5.49E-05	0.01016093	1.79913509
STAC2	-1.1713635	0.19487265	-4.4101372	5.73E-05	0.01050725	1.76089471
CDK6	2.97438133	9.08310792	4.40290317	5.87E-05	0.01060772	1.73923988
MVB12B	1.60826006	7.67171351	4.40038311	5.91E-05	0.01060772	1.73169942
CHSY3	-1.7520173	0.38083331	-4.3996128	5.93E-05	0.01060772	1.72939498
DOCK3	-3.4186856	3.76343558	-4.3968513	5.98E-05	0.01061673	1.72113457
WBP11P1	-1.1537414	0.25186336	-4.3881982	6.16E-05	0.01083418	1.6952639
PRSS1	5.05491465	2.51629242	4.37559705	6.42E-05	0.01120083	1.65762551
LINC02159	-2.6788465	0.72398867	-4.3702319	6.53E-05	0.01130857	1.64161334
FOXN2	1.39271362	6.94783698	4.36605299	6.62E-05	0.01137365	1.62914685
EFS	-1.0869223	0.11946048	-4.3517345	6.94E-05	0.01182629	1.58646824
PNMA6A	-1.577187	0.2081152	-4.3422562	7.16E-05	0.01197385	1.5582472
CLDN18	-3.1020299	0.5238399	-4.3408167	7.19E-05	0.01197385	1.55396353
A4GALT	4.48011823	2.25162688	4.32440961	7.59E-05	0.01253748	1.505178
SIX2	-3.4220435	1.22771867	-4.3141449	7.85E-05	0.01286682	1.47469472
ANTXR1	-4.462987	0.88383882	-4.3030239	8.14E-05	0.01321615	1.44170156
S100A11	3.4349556	11.4202202	4.2977116	8.28E-05	0.01321615	1.4259539
HOXA9	6.53208007	6.42587108	4.2972371	8.29E-05	0.01321615	1.4245477
ZFHX3	1.71298396	7.19986588	4.29678744	8.30E-05	0.01321615	1.42321514
PGGT1B	2.01140602	6.05230926	4.28939363	8.51E-05	0.01343974	1.40131218
PID1	-4.2107872	1.90940691	-4.2824469	8.70E-05	0.01364774	1.38074777
ZNF274	-3.7022239	7.58808481	-4.267302	9.14E-05	0.01423417	1.33596247
HOXA-AS3	3.74906605	3.34278812	4.25959324	9.37E-05	0.0143098	1.31319218
RTN4RL1	-5.9390232	2.40181774	-4.2585687	9.40E-05	0.0143098	1.31016716
FOXL2	-3.1997936	0.61132263	-4.2574723	9.44E-05	0.0143098	1.30693026
RUNX1	2.29587715	8.39343799	4.24155602	9.94E-05	0.014866	1.25998099
EGFL6	-1.540572	0.26072089	-4.2395529	0.00010003	0.014866	1.25407744
EXOC2	1.15275581	7.88781237	4.23880806	0.00010027	0.014866	1.25188268
LIFR	-1.9260751	0.25202519	-4.2331704	0.00010212	0.01503735	1.23527539
KIF5C	-4.4113433	1.66823454	-4.2107772	0.0001098	0.01595178	1.16940238

CXCR4	6.83209364	4.5676605	4.20820784	0.00011072	0.01597773	1.16185381
SLIT2	-3.6360151	1.36019626	-4.2049737	0.00011189	0.01603886	1.15235504
SPATA2L	-1.2797971	7.59004816	-4.200845	0.00011339	0.01614752	1.14023343
LINC02561	2.05484984	0.9357421	4.18732794	0.00011845	0.01666257	1.10058392
HPCA	-3.4408292	1.90921927	-4.1870838	0.00011855	0.01666257	1.09986831
TOP3A	1.33624821	8.15929407	4.18273698	0.00012022	0.01678906	1.08712991
HTRA3	-1.6986749	0.30368859	-4.1567704	0.00013072	0.01809887	1.01115504
ARMCX1	-3.2958137	0.73184535	-4.1554615	0.00013127	0.01809887	1.00733078
NMNAT3	1.791105	6.69902852	4.13473064	0.00014033	0.01922464	0.94683308
GHR	-1.2647138	0.21038025	-4.1255782	0.00014451	0.01967377	0.92016684
SEC14L5	-2.4003937	1.354019	-4.1224151	0.00014599	0.01975037	0.91095697
AACSP1	3.39750711	1.0342514	4.11480886	0.0001496	0.02011268	0.8888232
LINC00239	3.11094836	4.72751961	4.10856988	0.00015262	0.02023775	0.87068184
IL20RA	2.82663003	7.63713584	4.10711835	0.00015333	0.02023775	0.86646294
RERG	-2.6127765	0.4541292	-4.1005775	0.00015658	0.02054117	0.84746016
TM4SF1	5.11843121	8.27769904	4.09259936	0.00016063	0.02093117	0.82430015
MCTP1	3.12211935	3.76723356	4.08915095	0.00016241	0.02093117	0.81429597
ARHGAP29	3.57140734	5.7357417	4.08907557	0.00016245	0.02093117	0.81407733
ANXA5	1.51182058	9.69140923	4.08170258	0.00016633	0.02130389	0.79270062
RGL2	-1.6673966	9.12525869	-4.0787689	0.0001679	0.02137815	0.78419991
ZNF32	1.63578912	6.71332562	4.07389671	0.00017053	0.02158667	0.77008799
GFPT2	-3.391938	1.28407794	-4.0631932	0.00017646	0.02219891	0.73911336
ZNF556	2.48229863	0.52946142	4.0614974	0.00017742	0.02219891	0.73420941
NFE2L3	1.53270999	9.65271878	4.05782048	0.00017951	0.02220833	0.72357956
CORO6	-2.7123538	4.51526204	-4.0577621	0.00017955	0.02220833	0.72341092
TRIM36	3.56214658	4.80772613	4.05247436	0.0001826	0.02245771	0.70813198
ARL15	1.47197696	5.81900562	4.03656463	0.00019209	0.02349194	0.66221665
KRBA1	-4.2351707	1.43486218	-4.0345313	0.00019334	0.02351165	0.65635464
LURAP1	-2.1765741	0.61078471	-4.025968	0.00019868	0.02392264	0.63168129
HOXA1	3.46451686	3.80279225	4.02475284	0.00019945	0.02392264	0.62818218
VTCN1	1.92607041	0.49816795	4.02161101	0.00020145	0.02392264	0.61913717
BLVRA	3.57140694	6.69478855	4.02074249	0.00020201	0.02392264	0.61663737
NUDT16L1	-1.0895272	8.27666679	-4.0185567	0.00020342	0.02392264	0.61034723
EEPD1	1.83594768	9.07429432	4.01696569	0.00020445	0.02392264	0.60576978
GLI3	-1.3126977	0.22536144	-4.0153464	0.0002055	0.02392264	0.60111181
TMEM154	4.32907388	4.80521437	4.01525572	0.00020556	0.02392264	0.60085098
RNASE1	6.35714395	6.54508211	4.00133885	0.00021485	0.02473721	0.56085517
HOXA4	3.0868159	3.57552386	3.99693464	0.00021787	0.02492827	0.54821151
B4GALT5	1.22627262	9.3442515	3.99557589	0.00021881	0.02492827	0.54431213
CECR2	-5.2243574	2.57218563	-3.992038	0.00022128	0.02497476	0.53416186
C19orf81	-2.6103452	0.59285338	-3.9911187	0.00022192	0.02497476	0.53152509
CD177	-2.5770808	0.94496684	-3.9887611	0.00022359	0.02497476	0.52476435
MSX1	3.45730809	6.18185507	3.98841395	0.00022383	0.02497476	0.52376888
SERPING1	-3.6614562	0.58674372	-3.9804019	0.00022959	0.02535513	0.50080784
FHL1	-5.7230795	1.89641827	-3.9645045	0.00024142	0.02652724	0.4553143
CEMIP2	2.14486589	10.1539445	3.95902962	0.00024564	0.02675485	0.43966689
B4GALT7	-1.5231127	8.13781303	-3.9586029	0.00024597	0.02675485	0.43844761
KRBOX4	-3.2393336	5.88350536	-3.9552799	0.00024856	0.02690187	0.42895612
MGST2	1.3328235	8.96869021	3.94182705	0.00025934	0.02792858	0.39056926
HDDC2	1.49233391	8.56104398	3.93671317	0.00026355	0.02824187	0.37599373
LINC01647	-2.0416279	0.54189495	-3.9317705	0.00026769	0.02854373	0.36191477
LOC10272451	-1.1579343	0.16441702	-3.9236962	0.00027458	0.02913501	0.33893441
B3GNTL1	1.43766648	6.86232027	3.91648367	0.00028088	0.02965814	0.31842587
DIRAS1	-2.9373097	1.38645929	-3.9135078	0.00028352	0.02970981	0.30996952
MAP3K8	1.69842541	6.49409238	3.91284144	0.00028411	0.02970981	0.30807641
SLC35B4	1.15864423	7.18428878	3.91027639	0.00028641	0.02980626	0.3007906
TCTA	-1.5308926	7.65380646	-3.8890475	0.00030615	0.03170733	0.24058185
NME5	-2.3092447	0.55794165	-3.8854626	0.0003096	0.03191289	0.23043053
RHOBTB3	2.82568023	9.1726568	3.8815895	0.00031338	0.03203214	0.21946813
BIRC7	2.82637661	0.70171578	3.88124655	0.00031372	0.03203214	0.21849771
KIRREL2	-4.3881807	1.27101332	-3.874893	0.00032002	0.03237036	0.20052725
TGFBI	4.39678253	10.799605	3.8719204	0.00032302	0.03252092	0.19212464
IVD	-1.4971313	9.60190882	-3.8584737	0.00033689	0.03333775	0.15415476
TMEM54	-1.8996306	9.89400387	-3.8580902	0.00033729	0.03333775	0.15307283
DALRD3	-1.3486666	7.24140557	-3.8550329	0.00034053	0.03338738	0.14444951
PPP1R1A	-3.5323335	1.29279194	-3.8547034	0.00034088	0.03338738	0.14352057
SESTD1	1.60339875	6.66878818	3.84892781	0.00034708	0.03384172	0.12724004
HOXA7	4.7991735	3.39791217	3.84406384	0.00035239	0.03420514	0.11353885
RTN4	1.37009346	10.1896715	3.82474009	0.00037425	0.03556938	0.05919263
CPAMD8	-3.4045033	1.37469655	-3.8243952	0.00037466	0.03556938	0.05822379
FGL2	2.32893651	0.78070505	3.82113356	0.00037848	0.03577531	0.04906496
SFXN5	-2.7197387	7.29239315	-3.8181592	0.000382	0.03595086	0.04071618
RHOXF1P1	-1.1819795	0.17429129	-3.812633	0.00038862	0.03641556	0.02521335
GPR20	-1.4953565	0.43465404	-3.8092095	0.00039277	0.03655673	0.01561494
SH3TC2	3.46705262	5.81034897	3.80489117	0.00039808	0.03655673	0.00351421
LINC01783	-1.2998529	0.25554673	-3.803135	0.00040025	0.03655673	-0.0014051
WDR17	3.28896937	1.02308455	3.8031215	0.00040027	0.03655673	-0.0014427
COL4A1	4.55935745	1.88571838	3.80235926	0.00040122	0.03655673	-0.0035774
DYNC2LI1	1.08329585	6.54345859	3.80088719	0.00040306	0.03655673	-0.0076995
BORCS5	-1.4416956	6.2416512	-3.7991562	0.00040523	0.03655673	-0.0125455
FKBP14	1.12947331	7.21837905	3.79804499	0.00040663	0.03655673	-0.0156558
TJP2	1.6080605	10.1622655	3.79774049	0.00040701	0.03655673	-0.016508
CDHR5	-4.4553342	7.22438874	-3.7921815	0.00041409	0.03672973	-0.03206
KRT15	3.90441104	6.18694089	3.790911	0.00041572	0.03672973	-0.0356128
TMEM241	1.14916036	6.44190848	3.78575332	0.00042242	0.03716989	-0.0500292
ORMDL2	-1.4743587	7.24106846	-3.7812559	0.00042835	0.03753877	-0.062592
SPHK2	-1.6019688	8.11231124	-3.7705297	0.00044281	0.03834019	-0.0925222
TCEAL9	-6.1227669	6.04169953	-3.7674806	0.000447	0.03854923	-0.1010224
ADGRE1	-2.5293499	0.54543836	-3.7654842	0.00044977	0.03863396	-0.1065858
DNAH14	-4.3395128	5.49195218	-3.7632429	0.0004529	0.03874875	-0.11283
DSEL	-2.2416542	0.61120133	-3.7579198	0.00046041	0.03923617	-0.1276523
CPM	-4.0062083	1.76739428	-3.7542514	0.00046565	0.03952752	-0.1378608
TAC3	-5.0223887	2.25378987	-3.7476702	0.00047521	0.04018091	-0.1561619
TOP2A	1.14043951	10.8307109	3.74215716	0.00048335	0.04064046	-0.1714796
PLPP2	2.58673492	9.92642491	3.72072147	0.00051633	0.04284538	-0.2309254
NR3C1	-4.0516734	1.55748241	-3.7204445	0.00051677	0.04284538	-0.2316924
CPD	1.50987222	9.17054785	3.71929749	0.00051859	0.04284538	-0.234868
C11orf74	2.1844838	5.56672771	3.71687062	0.00052248	0.04300206	-0.2415855
IGF2R	1.48876432	10.0328197	3.71367966	0.00052763	0.04325285	-0.2504145
FBLL1	-3.2436648	1.43089565	-3.7125123	0.00052952	0.04325285	-0.2536434
FKBP4	-1.1629284	10.8951765	-3.7103842	0.00053299	0.0433728	-0.2595284
MSH2	1.55115351	8.94370949	3.70787269	0.00053712	0.04354486	-0.2664713
TTC37	1.07839024	8.9117357	3.69957414	0.00055097	0.04450123	-0.2893945
DUSP4	3.94613625	7.64952058	3.69058545	0.00056636	0.04557432	-0.3141932
MCCC1	-1.1693012	8.21096545	-3.6886739	0.00056969	0.04567216	-0.3194626
CEP135	1.54942214	6.24017357	3.68647468	0.00057354	0.04581113	-0.3255236
BEX3	-7.1153773	6.03534368	-3.6844012	0.00057719	0.04593337	-0.331236
MRPL23-AS1	4.52759871	4.04152834	3.6788084	0.00058715	0.04655511	-0.3466358
SDR42E1	-5.2123089	4.55688841	-3.6766625	0.00059102	0.04669065	-0.3525412
MIR4489	-1.0200959	0.2799595	-3.6722245	0.00059909	0.04691123	-0.3647484
OLIG1	-1.1113257	0.13552683	-3.6708969	0.00060153	0.04691123	-0.3683985
UGT1A10	-4.678879	6.35786076	-3.6704164	0.00060241	0.04691123	-0.3697196
PRSS22	3.77339233	6.59431739	3.67035342	0.00060253	0.04691123	-0.3698927
HAUS6	1.20848016	7.52075077	3.66920485	0.00060465	0.04691123	-0.3730498
MIR7-3HG	-2.8812456	0.70215316	-3.6654739	0.00061158	0.04727947	-0.3833017
HS6ST3	-1.1874047	0.24679828	-3.6610583	0.00061988	0.04775065	-0.3954274
DOCK4	3.34989573	4.05446995	3.6576551	0.00062635	0.04788347	-0.4047675
CLEC18A	-1.6663687	0.70950795	-3.6571562	0.00062731	0.04788347	-0.4061364
GALNT14	-2.3627944	0.48801185	-3.6566678	0.00062824	0.04788347	-0.4074761
PDGFD	-1.57692	0.24106264	-3.6535875	0.00063417	0.04816587	-0.415925
P2RY4	-1.3495348	0.41974685	-3.6507413	0.0006397	0.04824708	-0.4237281
PRPH2	-1.8144872	0.60756075	-3.6471643	0.00064671	0.04853941	-0.4335301
SLC6A20	5.18395901	5.51784718	3.64523518	0.00065052	0.04853941	-0.4388144
LRRN2	-2.7659756	1.08848742	-3.6442166	0.00065254	0.04853941	-0.441604
PRAME	-6.8601215	3.40197432	-3.6412341	0.0006585	0.04868672	-0.4497692
ZBTB42	-1.6132845	6.44833662	-3.6409722	0.00065902	0.04868672	-0.450486
