# Supplementary material for: PI3K-driven HER2 expression is a potential therapeutic target in colorectal cancer stem cells
Source: Gut. 2021 Jan 12;71(1):119–28. doi: 10.1136/gutjnl-2020-323553 (PMC8666826; doi:10.1136/gutjnl-2020-323553)
Supplement: Supplementary data [file gutjnl-2020-323553supp006.pdf]

Supplementary Table 3. Statistically differentially expressed genes in CD44v6^{high} vs CD44v6^{low} cells.

Gene	High	High	Low	Low	High	High	High	High	Low	Low	High	High	Low	Low	Low	Low	High	High
SHISA6	0	0.701667	0	0	8.34414	8.01094	0	0	0	0	8.341392	7.8355	1.782752	0	0	0	0	0
F5	5.76788	6.936854	0	1.603113	3.108141	3.179692	3.293148	1.672125	2.336277	3.163809	7.424792	7.811223	2.088965	2.266084	2.002808	0	6.156955	5.999978
FRMD6	4.934959	4.703506	3.751148	3.72551	5.65957	4.402779	3.095078	2.918484	2.084058	2.560629	5.329838	5.923904	1.393533	0	0	0.706894	1.562827	0
LOC44017	0	0	0	0	2.266443	1.592294	3.095078	2.609429	0	0	2.827997	2.604511	0	0	0	0	0	0
TFAP2C	0	1.809634	3.135971	2.830649	7.647176	7.296315	5.968492	4.688413	3.422226	2.703124	2.639075	3.776591	0	1.396666	0	0	2.778832	3.190442
ABCB1	7.800385	7.334086	3.751148	1.603113	0	1.005502	6.496372	7.826378	9.275744	9.21546	4.510256	3.396224	8.814137	8.597899	11.36486	11.14865	4.921952	5.695038
GLI2	0	1.809634	0	0	5.465822	5.277486	0	1.969301	0.855986	0	6.952905	6.636057	0	0	0	0	0	0
AQP5	5.181089	4.111529	2.504466	1.603113	11.91983	11.45081	3.467277	2.425909	0.855986	1.504553	8.650745	8.575553	2.908593	1.396666	0	2.872407	5.393717	6.131254
MBD1	9.26306	9.054052	9.416716	9.544773	9.711197	7.947576	9.636151	9.28766	9.869956	9.724837	8.424253	8.321415	9.972966	9.791237	9.310368	9.357658	8.532811	8.87788
MPP1	7.620185	7.667621	1.35509	0	1.182979	1.005502	5.275191	7.116559	4.750599	6.001869	1.455811	1.182986	7.686703	7.379116	4.957818	4.364633	5.748641	5.066762
LINC00909	6.393655	7.361821	6.861729	7.08222	5.088808	5.752246	5.07446	4.948917	6.675949	5.729519	3.814889	4.577026	7.266393	6.985674	6.020493	6.769783	6.156955	5.577163
GABRE	1.096298	2.58762	7.949446	8.440689	8.539049	7.772484	9.212573	8.345985	9.555885	8.856128	3.98335	3.904493	8.299447	8.682518	5.842881	6.60151	7.660318	7.982971
CTDP1	8.503432	8.091966	8.20695	7.998577	8.238867	7.762476	8.271869	8.460873	8.805124	8.755452	7.590526	7.225392	8.63677	8.760637	8.886377	8.958101	7.4667	7.490538
LINGO1	0	0.701667	5.948767	5.384036	7.875061	7.872023	1.174725	1.672125	0	0.68926	4.204068	6.121119	1.782752	2.687751	0	0	2.295325	0
LINC01234	2.142139	1.171698	1.35509	1.603113	1.182979	0	3.622635	4.98257	3.18586	2.832801	7.982303	7.79893	3.314649	3.107741	1.324175	1.534489	7.891619	8.21774
SHLD1	5.429883	5.584323	4.780742	5.581157	3.90448	2.916414	5.939224	5.529653	4.99004	5.284376	4.564402	3.776591	6.419793	5.674584	6.307476	6.534209	6.11689	6.251575
PRDM8	5.135108	4.395355	6.822545	6.945781	7.365344	6.647498	4.311973	3.819772	3.18586	3.163809	7.310624	7.951109	1.782752	0	4.603501	4.531694	4.611873	2.827144
ISX	2.473063	3.192664	6.631719	7.265367	3.108141	3.179692	7.547741	8.174594	3.422226	2.402502	2.827997	3.396224	8.234678	9.792123	5.952049	6.087936	6.837321	6.212573
TXNL1	9.163501	9.591431	9.110035	9.516951	7.958483	7.932779	8.090745	8.567282	8.770755	8.871302	8.011833	7.823413	9.629039	9.266947	9.170526	8.859428	8.446543	8.611717
HOXC13	1.096298	0	0	0	0	0	1.174725	0	0.855986	0	3.901577	4.231623	0	0	0	0	4.491695	3.480412
SHROOM2	4.880261	6.061133	0	0	4.976859	3.402258	6.7701	6.930796	4.034737	5.214704	5.87092	5.862224	0.858862	0	2.250883	2.439889	2.295325	0
NOS3	6.64401	6.559661	3.37139	3.932237	7.794796	7.225229	7.721114	7.014516	6.675949	6.556021	2.995019	2.062176	5.638549	6.478615	8.093841	8.566461	6.70845	6.326553
FGF1	0	0	0	0	0	0	0	0	0.855986	0	2.165499	2.062176	0	0	0.80896	0	0	0
CAB39L	4.425255	4.478779	3.37139	4.113021	5.192692	4.054714	8.143024	8.806851	6.73199	6.874879	4.715622	4.790757	10.08095	9.238628	8.327911	8.578442	8.131693	8.061893
PRDM16-D	0	0	5.075565	3.72551	4.02196	4.654402	0	0.789892	0	0	3.98335	2.997752	0	0	0	0	3.429162	3.72174
AOAH	1.711982	1.525643	6.019777	8.270225	4.23161	3.533593	8.805459	7.638604	6.629495	7.313007	3.814889	1.824127	7.438664	7.350531	7.107003	7.622115	7.000349	6.817271
CLN5	6.692438	6.140135	7.06006	6.921732	6.362976	6.725124	7.547741	7.690436	6.868259	7.26211	5.232862	5.938921	7.686703	7.38852	7.823905	7.880061	6.270876	6.764315
SMAD4	8.01869	8.576828	7.589839	8.279717	8.201997	7.437184	7.468011	7.669926	7.586157	8.015939	6.636415	7.194442	8.170942	8.444057	7.995249	8.780725	7.728375	7.630274
PNMA2	1.096298	3.192664	3.37139	4.418177	7.273558	5.518537	1.174725	0	1.778203	1.504553	8.372076	8.942614	0	2.560415	0	1.534489	0	1.600378
DKKL1	0	0	0	0	1.824117	2.594121	2.254743	0.789892	1.778203	0	1.455811	2.266453	0.858862	0	0	0	2.295325	0
KIRREL3	1.711982	1.525643	5.320242	6.135275	1.182979	2.816774	4.903157	3.051277	2.550894	3.163809	4.80829	4.414637	0	0	1.703032	0.706894	0	0
AOC1	5.351624	5.042011	7.307167	6.596713	8.002031	7.862685	10.44793	9.881919	9.281389	9.196007	4.7627	5.546483	9.639378	9.803591	7.352447	8.157492	9.922352	10.25546
ELAC1	6.559498	6.845512	5.375436	5.311853	3.480731	4.893255	3.293148	5.907962	5.257757	5.81047	5.01685	4.414637	6.53819	6.310711	5.804575	5.990871	5.393717	5.905317
IRX3	4.499719	4.351761	6.538068	6.049094	8.085372	7.538401	0	0	0.855986	0	2.165499	4.279598	0	0	0	0	2.295325	1.600378
UBASH3B	2.968703	1.525643	5.795315	6.71338	8.499081	8.185871	2.592156	4.26481	3.051365	2.703124	8.286071	7.718542	1.393533	2.266084	0.80896	0.706894	3.429162	2.827144
WNT7B	1.711982	4.00299	6.2733	5.699038	9.322011	7.76582	2.592156	1.969301	4.56559	2.832801	6.311791	6.480543	2.341426	2.266084	0	2.74213	2.295325	3.72174
SAMD13	2.968703	2.860731	3.751148	4.273652	1.182979	1.592294	4.903157	4.647038	3.80322	2.402502	1.85386	0	2.556218	4.021536	3.325297	4.319659	2.295325	3.928426
SHISA2	2.142139	3.288264	5.754258	5.641301	10.39626	10.72694	0	0	0	0	2.995019	2.604511	0	0.626961	0.80896	0	0	1.600378
ADAM19	3.490888	2.980064	1.35509	0	3.90448	1.592294	7.557404	5.976372	9.080197	8.560946	5.298232	4.07731	0	2.420743	0	0	4.921952	5.999978
SOX8	5.674527	5.425179	8.079787	7.814921	9.728585	8.640312	6.052892	5.99298	0	0	4.80829	5.604141	2.556218	4.702557	0	0	0	1.600378
PKIB	4.880261	5.761353	6.1521	7.08222	0	0.588633	5.019589	5.07906	4.834906	6.655786	2.421632	2.062176	5.973476	5.579256	7.503567	7.272821	1.562827	2.340407
RBM26-AS	4.702501	6.376016	3.37139	1.603113	3.306431	3.402258	4.708737	4.604443	5.608506	4.63725	2.639075	4.498115	5.566072	6.074797	4.992309	5.947185	4.921952	5.577163
PRODH	9.771303	9.868726	7.723475	6.366658	6.094529	6.772434	4.637637	5.596383	4.034737	4.229682	9.121666	9.053704	2.088965	4.951259	1.703032	2.057079	4.921952	3.480412
WWC2	3.490888	4.632417	1.35509	5.518396	7.994864	6.437121	3.762878	5.226789	6.641249	6.195147	5.232862	5.764459	2.088965	2.092833	1.324175	1.179238	4.921952	5.448794
AKAP12	3.490888	3.377921	0	3.484125	8.799793	4.50235	5.07446	6.134423	3.80322	3.659544	5.092505	5.358241	4.46948	1.396666	0	0.706894	0	3.928426
GJA3	5.268875	5.858002	0	2.830649	5.423742	4.791945	1.174725	2.772216	5.457127	4.487741	4.134181	4.07731	2.743137	2.804754	0.80896	1.179238	1.562827	0
STX18-AS1	3.630002	5.095853	1.35509	1.603113	4.577013	4.097802	5.451374	4.090583	5.850492	4.982893	3.144696	3.396224	5.590637	5.2065	4.992309	4.970381	4.611873	5.380055
PLEKHO2	4.499719	4.05828	4.051486	3.484125	6.733997	5.881161	5.847682	6.316981	5.767118	5.455226	7.537369	7.8355	4.799555	4.60912	3.703894	4.608488	5.012084	5.577163
GSTM4	8.886587	8.957379	9.208312	8.62326	6.530952	6.947532	7.771801	7.942079	5.483486	5.552568	6.247047	5.830366	8.539177	8.297015	6.347992	7.481172	7.915172	8.197908
ZNF772	4.880261	6.227148	0	0	2.266443	1.592294	4.116654	1.672125	0	1.786523	4.134181	5.033933	3.631162	2.560415	2.002808	2.261144	0	0
SFMBT1	7.162459	7.399775	6.67636	7.038159	7.354185	7.510769	6.325933	7.04669	6.075097	6.407803	8.245216	7.656304	6.760169	6.487394	7.436262	6.752967	5.45927	5.953424
ECE1	9.305195	9.847303	10.20899	9.95044	11.93349	11.23438	10.12139	9.500073	10.67772	10.20529	10.27653	10.39763	9.330355	8.780417	9.329042	8.985354	8.478513	8.061893
ME1	9.225154	9.847303	9.366518	10.57305	10.17631	10.61692	9.661014	9.282603	9.225875	9.376999	9.089714	9.1012	8.542329	8.138851	8.484522	8.016656	7.315603	7.3358
PLD1	4.987659	5.727643	5.795315	6.71338	8.820211	7.453864	8.211947	7.617338	8.39029	8.124406	8.48166	9.269535	5.032571	4.203034	5.969467	6.563437	7.961154	7.784507
DACT2	0	0	0	0	0	0	0	0	3.18586	2.560629	6.661474	7.365358	3.809178	2.266084	0.80896	0	9.008391	8.596649
THUMPD3-	6.953146	7.205291	6.019777	7.08222	7.701019	7.277699	8.713376	7.858452	9.0319	8.140152	6.013985	6.271048	7.289086	7.633605	7.688473	7.883928	7.450672	6.988663
NUAK1	5.608757	6.114279	5.711999	6.768356	7.387406	6.843777	2.254743	3.051277	4.409384	5.090541	5.560481	6.530965	3.05701	4.203034	1.324175	1.534489	3.429162	1.600378
PCLO	5.504114	6.845512	3.37139	3.194075	1.182979	0	6.476136	6.603193	6.56924	6.418172	0.904236	2.266453	5.973476	5.512014	8.703306	9.183483	5.897958	4.880253
TIAM1	6.393655	6.047535	2.041335	2.343681	8.770712	8.087758	5.127322	4.948917	0.855986	0	6.490225	6.550651	0	0	0	0.706894	0	0
DUSP6	12.05187	12.52411	9.339014	10.54184	12.33572	11.12114	10.87242	10.46377	11.24155	11.06278	11.7872	12.21968	9.704134	9.975678	9.899692	10.49194	11.34985	10.63315
KRTAP5-1	0	2.58762	5.623576	5.155655	5.192692	4.59549	7.659637	5.890341	8.510519	6.724241	2.421632	4.07731	4.841099	5.227612	4.412957	6.101284	6.861769	6.622738
IDNK	5.911183	6.286236	5.711999	5.155655	3.480731	5.420117	5.36597	4.98257	5.430278	4.276026	4.510256	5.088821	5.81571	5.953324	5.765225	6.957633	5.695225	5.514406
SNHG11	7.162459	7.161776	7.723475	6.502571	6.751114	7.135026	7.270667	6.697403	8.139902	7.63978	6.911726	6.896672	8.078438	7.992359	7.852349	8.105448	7.926806	8.157406
SH3BGR	4.346738	4.632417	3.573728	3.72551	3.90448	3.964473	3.293148	1.969301	4.409384	4.080987	3.144696	3.396224	4.756778	5.459443	4.511373	5.329185	2.778832	4.109178
CNDP2	9.927831	9.50328	10.39744	10.13956	8.895268	9.536489	9.815973	10.06977	10.68342	10.47111	9.209242	8.929517	11.70811	11.59337	11.14728	10.69762	9.217022	9.528842
ANXA13	6.912837	7.870454	7.263944	6.921732	0	0.588633	3.293148	4.027495	2.336277	3.348635	0	0	3.191573	2.804754	4.463004	4.408246	4.216369	4.269784
GPR162	0	0.701667	0	2.343681	3.636225	3.257759	0	0.789892	1.389562	0.68926	2.995019	3.396224	1.393533	2.092833	0.80896	1.179238	0	3.480412
HS3ST3B1	2.968703	4.00299	5.795315	5.518396	1.824117	2.330721	1.174725	0.789892	2.336277	2.224885	4.937006	4.651844	1.782752	3.432632	3.325297	2.872407	0	0
QKI	0	0.701667	0	0	0	0	0	0.789892	0.855986	0.68926	6.698265	7.249683	4.756778	5.927752	0	1.534489	0	0
NUAK2	6.75458	6.994686	6.937041	7.649056	7.070361	7.424547	8.277727	8.445872	7.010996	7.61295	6.780628	6.865574	7.965574	7.593509	7.683204	7.66769	7.949795	8.05088
RRAGA	7.636779	8.172477	8.802922	9.153797	8.807995	9.060811	9.32433	9.577818	9.178259	9.876356	7.66676	7.035871	9.24781	8.991067	9.017755	8.86139	9.500043	9.434539
MIR4458H	5.82692	5.095853	0	0	6.198017	6.022041	5.227566	4.370271	6.328757	5.678675	0	0	6.335095	6.668134	5.479392	5.055134	6.596365	6.791036
ANO9	8.01869	8.62569	10.35173	10.47524	10.74493	10.07302	12.39624	11.24751	12.67706	11.57247	9.159537	9.798187	11.15682	12.35249	10.72655	11.27403	11.74352	11.34216
PIAS3	7.865194	8.124711	6.861729	7.526838	8.995628	8.319288	8.283561	8.156279	8.81294	8.959138	8.764983	9.055427	7.709313	7.531196	7.138142	7.380754	8.09091	7.730311
KLF12	5.225649	6.429417	3.909118	5.155655	5.862211	4.565106	3.622635	5.872501	6.357544	6.57468	6.343105	6.500924	1.393533	0	4.251473	4.491707	4.36059	5.066762
CHST15	0	0	6.84227	6.969436	8.951883	7.976724	0	3.486101	4.793368	4.1818	7.873914	7.354199	5.032571	2.266084	0	0	6.306934	6.397825
ANO10	6.45108	6.386855	8.144769	7.284352	7.794796	7.665565	8.40092	8.13399	7.862072	7.674796	6.196508	5.712974	6.952504	7.064055	7.391737	7.45021	6.681235	6.498538
ZNF566	5.883639	6.559661	6.12014	6.330581	6.094529	6.160576	5.36597	5.28189	6.126181	5.116244	7.133898	7.446407	5.48976	4.50921	4.511373	5.479868	6.474833	6.131254
CXCL17	1.096298	0.701667	2.041335	1.603113	7.492923	6.129767	0	0	0.855986	1.153748	1.85386	3.776591	0.858862	0	0	0	1.562827	0
SALL4	1.096298	2.428861	0	0	0	0	2.865422	3.389056	4.234187	2.402502	5.16439	6.294588	0	2.804754	0	0	4.611873	4.109178
MYO5B	8.448093	9.401394	9.161974	9.610188	10.21538	8.265976	9.248809	9.64372	9.830713	10.2187	8.364466	8.594644	9.335815	9.303446	9.84176	9.390648	8.494237	8.486521
ASCL2	11.0775	10.63323	11.5828	9.926469	9.148784	10.2416	11.20461	11.27196	11.20736	11.32873	8.729876	7.80304	12.5446	11.73947	11.01514	10.90925	11.87018	12.16548
LOC10192	0	1.809634	0	0	1.824117	1.592294	0	0	0	0.68926	1.455811	2.445361	0	0	0	0	0	0
GNG7	0	0	0	0	7.070361	6.453801	3.890688	1.672125	3.308878	3.432866	3.98335	5.465835	0	1.062548	0	0	6.075681	3.480412
CXXC1	9.633401	9.052351	10.30629	9.617692	9.102854	8.359678	9.722434	9.146344	9.8473	9.351526	8.516413	8.363761	9.9753	9.87474	9.653892	9.678033	9.083073	9.356181
OSBPL10	7.661318	8.147199	6.60887	7.510806	10.1905	8.785745	8.187263	8.608281	8.4213	8.591045	8.609491	8.820225	7.163712	6.588774	7.880243	7.423891	7.000349	6.988663
TEX30	8.345828	7.036588	7.912603	6.534639	6.956913	7.394622	7.316913	7.590308	6.878227	7.104103	6.071207	5.62286	8.808912	8.651609	7.677916	7.465774	6.70845	7.474142
STARD5	2.968703	2.980064	7.093364	7.444835	5.088808	5.881161	7.508426	7.446993	5.375032	5.533618	5.055173	5.695396	5.436546	6.424789	6.425763	6.935447	7.450672	7.141838
LOC10192	3.164525	2.730629	2.504466	2.830649	3.306431	4.219845	1.174725	3.577028	0.855986	0	2.995019	4.021973	0	0	1.324175	0	3.429162	3.928426
SCN1B	0	0	0	3.194075	3.108141	2.008245	3.762878	0.789892	2.73768	2.402502	5.01685	5.192705	0	0	0	0.706894	2.778832	0
PCDHGA4	0	1.171698	0	0	2.266443	0.588633	1.813523	0.789892	0	0	0	1.182986	0	0	0	0	0	0
TRIB2	8.180235	7.930954	5.577246	6.768356	8.779081	8.366301	2.592156	2.215594	5.632261	5.414373	7.482178	7.59126	0.858862	2.266084	0	0	3.875805	5.066762
AQP2	1.711982	0	0	0	4.790744	4.219845	1.813523	1.672125	2.084058	0	2.639075	3.396224	0.858862	0	0	1.534489	6.566929	6.622738
SLC4A3	6.183831	6.354089	3.135971	2.830649	7.943669	7.697431	2.592156	3.28501	7.411842	6.45892	5.199032	6.222782	0	2.266084	1.703032	4.015681	4.056113	4.666002
ZNF567	6.576801	7.142718	5.795315	5.808011	7.285356	6.811785	5.227566	5.552241	4.952792	4.132274	7.550843	7.451651	4.841099	5.001152	4.069608	5.74092	6.156955	6.045077
INTS6-AS1	3.336916	4.26041	2.041335	3.194075	2.878187	2.916414	4.637637	3.892388	5.809407	5.064373	2.995019	3.210702	4.620269	4.702557	4.412957	4.645405	5.521974	5.804005
ACOT1	4.934959	4.557642	3.135971	2.830649	4.722959	5.697271	4.116654	4.647038	2.903032	4.132274	6.418539	6.134226	4.920762	4.325587	4.690098	4.531694	2.778832	4.545628
SH3BP2	9.320683	10.29996	9.796258	10.15253	11.58183	9.846232	9.950401	9.829715	9.247184	9.164018	7.703418	7.843502	9.216583	9.277108	9.021935	9.214523	9.804333	9.835213
LOC101927	1.711982	0	2.041335	2.830649	0	2.468424	0	0	5.608506	6.288533	0	0	4.571737	1.062548	6.265788	5.947185	1.562827	0
SYP	4.175589	2.860731	5.668465	3.194075	3.90448	4.368001	3.890688	4.879161	5.584354	4.740019	4.395456	3.396224	6.229553	6.290789	5.745139	5.947185	5.252935	5.307877
CDHR5	6.373993	6.861143	7.601461	7.339854	5.830353	5.076746	10.07336	9.407097	9.218047	8.718173	5.533588	5.192705	7.917835	5.792617	6.450783	7.149744	6.761388	6.362629
MGMT	8.365874	7.585314	8.58609	7.461613	7.162799	7.190817	7.852512	7.108961	0	0	3.144696	0	8.567299	8.787542	8.121372	8.297438	8.894515	8.926896
IL12A	4.570528	3.823142	1.35509	1.603113	3.108141	2.816774	1.813523	0	0	0	2.165499	1.538883	0	1.062548	0	0	0	2.827144
SPRY1	6.871369	7.930954	7.680291	8.667318	8.31566	8.2204	7.828769	7.700582	6.357544	6.867331	8.424253	8.597012	7.465477	7.535435	6.374384	6.851046	8.265983	7.971336
DBN1	7.778118	9.045526	4.607151	5.311853	9.9374	9.54235	6.183435	7.446993	8.46825	7.621949	8.989378	8.789487	5.323843	4.640943	7.305215	8.803361	8.293203	8.470079

Gene	Low	Low	Low	Low	Low	Low	Low	Low	High	High	Low	Low	High	High	Low	Low	High	High
SHISA6	2.894221	1.742164	0	0	0	0	0	0	6.535551	5.784451	0	0.923506	0	0	0	0	4.936645	5.53536
F5	1.505082	1.119477	2.523927	1.902589	1.579399	0	0	1.79215	7.197204	7.405775	1.042062	2.455122	0	1.562693	6.192034	5.39321	0	0
FRMD6	0.940508	1.742164	0	0	0	0	0	0	6.357051	6.078074	2.234835	2.197512	3.279323	3.24299	0	1.262201	2.675001	2.411883
LOC44017	0.940508	3.375938	0	0	0	0	0	0	2.201237	1.166132	0	0	0	0	0	0	0.924276	2.757503
TFAP2C	5.203552	4.805896	0	0	0	0	0	0	5.299629	5.436266	0	0	1.455124	3.140156	3.774666	3.492687	4.803844	3.56123
ABCB1	8.164265	8.59111	10.15652	8.252716	8.746553	7.947773	9.002062	9.569283	0	1.166132	10.23578	10.52758	9.744776	9.224695	9.277585	9.07788	7.149177	8.315801
GLI2	0.940508	0	0	0	0	0	0	0	3.484965	3.74856	1.867074	0	4.563366	3.338979	3.908982	2.819803	0.924276	0
AQP5	2.703815	2.1757	3.022755	0	2.992693	2.741038	3.854658	4.252798	12.13821	12.07781	0.613139	0.923506	1.455124	1.562693	3.060865	2.241729	5.096602	3.646681
MBD1	9.264509	9.206671	9.390072	9.311741	9.555807	9.673964	9.848281	9.589413	8.255939	8.265233	9.188694	9.198333	10.32429	10.71328	9.527758	9.583911	8.690203	9.176586
MPP1	7.108121	6.766685	5.285832	5.571683	7.499487	7.175516	6.851329	7.617815	0.677882	0.697815	7.905943	8.028174	2.638167	3.140156	4.089571	4.330156	6.338034	7.173287
LINC00909	6.089623	5.84018	7.406644	6.624761	6.733192	6.691244	5.777855	6.90761	4.608323	4.297292	6.231543	6.352348	6.544044	7.236592	7.162096	7.099419	6.255679	5.855546
GABRE	7.565573	6.827534	8.821417	8.063827	9.092324	9.150077	9.180072	9.2878	3.321471	3.532913	8.974546	9.4354	6.446573	6.932522	7.92308	7.868119	9.843107	9.886808
CTDP1	8.437214	8.667992	8.172242	9.158839	8.782828	9.24438	9.016201	8.614138	7.499341	7.421565	8.519411	8.362817	9.053595	8.880423	8.916122	8.674061	8.403213	8.687482
LINGO1	2.484401	1.742164	0	0	0	0	0	0	6.727418	6.429955	1.640777	2.863351	5.19798	4.405436	1.223673	1.262201	0	0
LINC01234	7.008645	7.272596	0	0	0.995829	2.396196	2.12653	0	5.631771	5.953277	0	1.883682	3.982337	3.593705	0.737862	2.092015	5.274438	6.461954
SHLD1	4.836738	4.611978	6.07725	6.542374	6.201213	5.299289	6.52199	6.43287	4.458947	4.728132	6.339266	6.148611	4.269699	4.360398	5.405909	4.603492	5.86925	6.161637
PRDM8	1.505082	0	0	0	0.995829	0	1.424024	0	7.901568	8.193474	1.042062	0.923506	2.994073	4.982465	0.737862	0.950401	2.675001	3.56123
ISX	8.958176	9.166017	9.576689	8.882887	7.591602	6.430999	7.514119	8.406393	0	2.039219	9.272618	9.785411	1.455124	1.850158	5.711856	7.044432	8.485013	9.553692
TXNL1	8.711625	8.642817	9.121326	9.551762	9.392538	9.109816	9.141891	9.278292	8.103831	8.311164	9.032595	8.637518	9.000615	9.255771	9.189588	9.415863	8.50654	8.175487
HOXC13	0	0	1.755198	0.935796	0	0	0	0	2.201237	2.242534	0	0	5.448706	6.18286	2.32372	0.552086	6.627209	7.121989
SHROOM2	4.468693	5.223158	0	0	1.993739	3.252214	2.951088	1.401742	6.710992	6.051259	0	0	4.85144	5.370989	0.737862	0	6.460742	7.180468
NOS3	5.763247	4.805896	7.485262	7.498652	6.530895	6.500592	7.723942	7.417584	4.378028	4.102086	7.175098	6.320327	8.202091	8.052285	3.171916	3.080534	6.400692	5.210001
FGF1	0	0	0	0	0	0	0	0	1.485101	0.697815	0	0	0	0	0	0	0	0
CAB39L	9.138407	9.384258	7.189027	7.823351	9.971794	9.773382	9.002062	8.840319	5.232167	4.888213	7.082302	7.657248	3.721657	4.405436	7.469833	7.375356	10.10893	10.38102
PRDM16-D	0	0	0	0	0	1.275828	2.596932	0	4.535567	4.385808	1.867074	0.923506	0	0	0	0	0	0
AOAH	6.509094	7.476342	4.322347	5.515205	9.186417	8.482872	9.498148	8.078533	0	0	6.933983	7.072654	2.420752	3.741536	2.809275	2.501323	7.407468	7.187614
CLN5	7.297051	7.513852	7.714685	8.177373	7.892548	7.664167	7.136646	7.471976	6.563266	6.530457	7.22702	7.45091	5.477192	6.409799	6.952535	6.922207	6.77644	7.076519
SMAD4	7.992657	8.240216	8.836491	8.248392	8.344819	8.104997	8.333227	7.861403	7.430784	7.249418	8.25508	8.577828	9.154053	9.827072	8.27058	8.589286	7.684277	8.113053
PNMA2	2.225526	3.20301	0	0.935796	1.993739	2.396196	4.013459	0.864812	5.578442	4.976375	1.042062	0	6.51686	6.812251	1.586456	2.912049	0	1.287213
DKKL1	0	2.778926	0	0	0	0	0	0	1.485101	0.697815	0	0	0.903733	2.089751	0	1.262201	2.675001	1.287213
KIRREL3	0	2.1757	0	0	0.995829	1.275828	0	0	6.177417	5.981957	3.072822	3.317187	0	0	3.702508	3.367943	4.244478	3.56123
AOC1	9.12184	9.139264	7.559816	7.03948	10.37103	10.7066	10.48195	10.36867	1.999619	2.85209	8.504761	9.322741	7.925467	8.779254	8.731289	8.555719	10.51777	10.79599
ELAC1	5.880437	5.651128	6.025574	6.978121	6.635586	6.609603	5.18388	6.499848	3.699971	3.18374	6.491374	6.237022	6.635344	6.869627	5.674622	5.771186	4.936645	5.210001
IRX3	4.468693	4.541079	0	0	0.995829	1.275828	2.12653	0	6.507293	6.843411	0.613139	0.923506	6.342036	5.799951	5.884845	5.934054	0	0.782644
UBASH3B	2.484401	3.006495	0	0	3.69273	1.275828	0	1.401742	7.757051	7.20753	5.057651	4.656005	2.638167	3.24299	2.32372	2.912049	0	0
WNT7B	2.225526	0	0	0	0	1.275828	2.12653	2.099096	4.419055	4.826295	2.062641	2.197512	4.059719	2.089751	6.796636	7.118905	0.924276	0.782644
SAMD13	3.473948	3.530338	5.098461	3.464982	4.849916	4.480308	4.156501	4.122842	0	0	5.862825	6.28758	1.455124	2.295164	5.994671	5.695334	4.244478	4.671834
SHISA2	2.225526	1.119477	0	0	0	0	1.424024	0	7.79261	7.648729	0	0	0	0	0	0	0	0
ADAM19	3.349371	4.216473	0	0	0.995829	0	0	1.401742	2.80692	2.579269	0	0	4.714581	4.649611	4.892936	5.071862	6.911671	7.733101
SOX8	0.940508	1.119477	0	0	0	0	1.424024	0.864812	8.131865	8.146741	2.062641	1.883682	0	0	6.124605	6.593532	7.645993	6.852057
PKIB	4.468693	4.865146	6.585399	5.777782	5.161804	5.074321	4.888334	3.735147	1.999619	2.85209	5.620356	6.09284	3.517377	4.216178	4.683977	4.330156	3.318652	4.452078
RBM26-AS	4.468693	4.304724	5.735143	4.872893	6.036167	6.171305	5.98375	6.36263	2.535633	2.971314	4.752193	5.017261	3.813884	4.722609	5.237397	4.946595	4.707993	4.897785
PRODH	2.484401	3.20301	6.875419	6.020284	4.7993	3.785802	4.516222	3.821469	9.525405	9.213857	4.723561	4.976751	8.649666	7.788921	0	0	3.941454	3.470398
WWC2	0.940508	0	0	0	0	0	1.424024	1.401742	5.84248	5.260129	3.599468	3.555893	6.446573	6.596165	2.117201	2.819803	5.912896	6.806366
AKAP12	0	2.508614	0	0	0	1.275828	1.424024	0	5.061284	4.658771	1.640777	0	5.297177	3.338979	4.892936	5.642456	3.032428	1.660309
GJA3	2.225526	1.742164	0	0	0	1.275828	0	0	4.677584	3.608434	0.613139	0	5.091455	5.277176	0.737862	1.262201	1.483087	2.202074
STX18-AS1	3.793777	5.02956	5.371118	4.872893	4.515572	4.879096	5.18388	4.482117	3.887052	3.608434	4.694348	5.017261	4.133161	5.040722	1.223673	2.912049	4.605318	4.862499
PLEKHO2	4.583765	4.466515	4.139968	5.893083	4.7993	5.074321	4.714242	4.122842	6.201133	6.252997	3.884518	4.656005	6.889614	6.546765	6.110732	6.266624	6.400692	6.981041
GSTM4	7.718214	7.315712	8.694798	8.992968	9.081774	9.168012	8.490045	8.62618	5.523065	5.816653	8.323457	9.368474	8.123176	7.57397	8.443795	8.55214	6.573826	5.743637
ZNF772	6.00955	6.292727	0	0	0.995829	1.942141	0	0	6.829854	6.205145	0	0.923506	1.853078	3.741536	5.405909	5.031303	1.483087	1.660309
SFMBT1	6.86814	7.00924	6.987702	7.009127	7.41796	7.258212	6.808639	6.877702	8.357046	8.081978	7.386202	6.962864	8.105037	8.283985	7.042969	6.818762	6.867987	7.488882
ECE1	8.382632	8.528346	9.655301	9.898019	8.47951	8.59318	8.782504	8.507506	10.79958	10.61064	9.440361	9.098029	9.489089	9.625314	9.186285	8.958576	8.897234	9.102905
ME1	8.402363	9.074841	8.412481	8.186444	8.47951	8.394228	8.820601	8.471249	10.00456	10.20278	7.782984	8.526127	6.721209	5.639556	9.869002	10.00307	8.422215	7.380804
PLD1	6.833675	7.04811	4.883069	4.731317	6.546324	5.854372	6.991519	6.00506	8.51677	8.2298	6.291296	6.809841	3.403254	3.669513	7.653281	7.815347	6.203911	7.12943
DACT2	2.225526	3.530338	0	0	3.799599	3.927307	3.472485	0.864812	6.617149	6.387709	0	1.482042	2.164658	0	0	0	6.475372	6.596896
THUMPD3-	7.039215	6.735271	9.181593	8.698625	7.957464	8.246757	7.937125	7.772098	5.614213	5.574508	6.613442	7.557765	6.01292	7.554107	7.188752	6.800777	7.353053	7.005509
NUAK1	2.894221	3.530338	1.755198	0.935796	3.314412	2.396196	2.951088	2.099096	7.985905	7.894814	4.326611	4.802275	3.813884	3.875617	5.336085	4.856601	0	2.202074
PCLO	5.967783	5.433513	6.815821	4.197391	7.249089	6.691244	6.468146	6.178633	0	0	3.160882	4.309693	6.01292	7.5838	4.347905	3.607497	2.864755	4.302122
TIAM1	6.237554	6.376564	3.022755	0	4.746844	5.133936	2.951088	2.352056	8.905783	8.636136	0	1.482042	7.049913	5.480278	5.979485	5.998593	4.494771	5.18165
DUSP6	10.143	9.484493	8.442347	7.353184	9.322576	8.319169	9.124592	8.271059	12.63153	12.80138	10.01359	10.78679	11.35284	9.792522	9.58281	9.787128	9.406816	9.021264
KRTAP5-1	6.306195	5.84018	7.212327	6.245296	5.692247	6.083315	7.566305	6.917443	0.677882	1.166132	4.664532	5.369898	3.517377	4.449111	5.711856	5.819626	6.113275	4.897785
IDNK	5.203552	5.223158	6.721536	5.777782	5.51539	5.956968	5.732899	6.178633	3.321471	3.680197	5.490425	6.254073	2.994073	4.686572	4.924998	4.603492	5.654643	5.18165
SNHG11	7.850505	7.577237	7.280049	8.199944	7.719606	8.212601	7.579061	8.142812	6.526193	6.429955	7.296708	7.343492	7.379127	7.498031	7.397404	7.142899	7.878406	7.616153
SH3BGR	4.133043	3.669801	5.669318	4.628574	4.576958	4.283767	5.18388	4.894264	3.699971	3.279269	4.780268	4.892149	3.982337	3.938256	3.461468	3.551234	2.456453	3.646681
CNDP2	10.57984	10.7175	10.88031	10.95214	10.89025	10.97357	10.02183	10.52558	9.641545	9.910917	10.71133	10.60687	10.99604	10.81849	10.27642	10.18971	9.798718	9.989531
ANXA13	3.349371	5.810342	3.686908	5.081767	5.201326	5.989607	4.013459	6.273563	0	2.039219	2.527635	4.847911	0	0	0.737862	0.552086	3.032428	3.80375
GPR162	0.940508	0	0	0	0	0	0	0	3.035178	1.802476	0.613139	0	6.733069	7.036392	1.223673	1.924946	0	0
HS3ST3B1	3.473948	3.669801	0	0	1.579399	1.275828	0	0.864812	5.765037	5.683295	0	0	2.164658	3.029425	1.586456	2.377357	0	0
QKI	5.203552	5.311014	0	0	0.995829	0	1.424024	0	7.27748	6.926911	1.042062	0.923506	6.835634	7.384517	0	1.735965	0	1.956475
NUAK2	8.60327	8.604212	7.613308	7.088692	8.499464	8.645324	8.032961	8.32377	5.683199	5.032292	8.316284	8.550485	8.532401	9.112034	7.246986	7.059638	8.609605	8.572087
RRAGA	9.963382	9.83131	9.145736	9.033747	9.4613	9.542649	9.048665	9.049277	8.497745	8.535731	9.312818	9.153603	8.1806	8.704638	8.037489	8.133002	8.787205	9.20151
MIR4458H	5.880437	5.980761	6.987702	7.270294	5.664236	5.624301	6.87221	5.869801	4.201154	3.680197	5.011024	5.627106	5.264863	5.897759	5.211633	4.968243	0	0
ANO9	11.4196	11.16561	10.0635	10.78455	10.55485	11.32883	12.71443	12.02261	7.815838	7.635251	10.86343	11.59181	9.203907	9.949018	10.14817	10.12914	11.86225	11.27121
PIAS3	7.354902	7.388198	8.073912	6.345879	7.475509	7.633515	7.403719	7.039525	8.749698	8.719399	7.793353	8.018229	7.087352	8.131491	7.042969	7.243974	7.005376	7.703495
KLF12	4.740804	5.02956	0	0	2.315249	0	0	2.099096	6.065589	5.555552	1.640777	0.923506	5.091455	5.277176	0	0.950401	4.174478	4.965868
CHST15	6.089623	6.335255	0	0	0	0	0	0	5.185373	3.453219	0.613139	0.923506	2.994073	1.203326	5.748153	5.29384	4.893722	5.291857
ANO10	7.219089	7.541359	6.394899	8.159059	8.186432	8.177617	8.333227	8.267219	5.299629	5.394206	8.690055	8.644001	7.211228	7.071748	7.114229	7.039328	6.964479	6.939315
ZNF566	6.089623	6.007304	6.025574	4.827228	5.606527	5.191185	5.732899	5.30713	6.516774	6.606103	5.490425	5.799602	6.41747	6.566729	5.748153	6.249046	5.729782	5.12322
CXCL17	1.505082	0	0	0.935796	0	0	0	0	4.89675	4.693868	2.527635	2.197512	0	0	0	0	0	0
SALL4	5.946437	4.922058	0	0	0	0	0	0	4.153326	4.342229	2.878727	3.661903	3.279323	3.513692	4.860144	3.812881	3.557383	4.192726
MYO5B	8.824158	9.503265	9.868299	8.772769	10.08705	9.768688	9.534624	8.790359	8.171422	7.722818	10.01433	9.81012	10.08962	10.25996	9.740893	9.5996	8.582833	9.431098
ASCL2	11.54472	11.37947	8.930808	10.35352	11.47244	12.41482	12.36676	12.62707	8.594938	8.409454	11.47629	10.86933	5.803917	5.202583	9.433386	9.255647	12.93713	12.38119
LOC10192	0	0	0	0	0	0	0	0	0.677882	0.697815	0.613139	0	0.903733	1.562693	0	0	0	0
GNG7	1.505082	1.119477	0	0	0	0	0	0	5.112207	4.7616	2.654399	4.706428	5.953335	7.39013	1.223673	1.924946	2.456453	2.411883
CXXC1	9.173279	9.02375	9.676398	10.45834	8.991663	9.64864	9.988752	9.956685	8.679677	8.765495	9.21593	9.262931	10.55392	10.49624	9.361986	9.411919	9.312194	8.976582
OSBPL10	7.288593	7.736439	7.746954	7.714688	8.196246	8.036358	7.566305	7.445036	9.508872	9.24525	8.104989	8.110054	6.697193	6.409799	7.772618	7.833153	7.07427	8.210989
TEX30	7.711903	7.272596	6.585399	7.998375	7.576652	8.074893	7.500772	8.204349	1.137227	0.697815	7.001129	6.983457	7.720326	5.748442	6.669921	7.089576	8.039674	7.180468
STARD5	5.608768	5.509757	6.875419	6.74014	6.862083	6.928573	7.187514	6.333548	3.944384	4.048859	5.836326	6.166732	6.070141	6.233693	6.318116	6.400036	3.663403	4.07435
LOC10192	0.940508	1.119477	0	1.902589	0	1.275828	0	0.864812	4.419055	3.081433	2.062641	2.673628	0.903733	0	1.223673	0.950401	0	0
SCN1B	1.505082	2.1757	0	0	0.995829	0	0	0.864812	1.765176	1.166132	0	0	2.638167	3.428979	1.586456	0.950401	1.483087	0
PCDHGA4	0	1.119477	0	0	0	0	0	0	1.485101	2.039219	0	0	1.455124	1.203326	0	0	0	2.411883
TRIB2	3.886361	3.796962	5.527909	5.936741	0	2.741038	1.424024	0	7.348224	6.763065	1.372257	0	6.868264	6.992789	3.060865	2.092015	1.483087	1.660309
AQP2	1.505082	1.119477	0	0	2.578004	2.396196	1.424024	1.401742	6.177417	5.909157	1.372257	1.482042	0	0	0.737862	0.950401	3.44295	1.956475
SLC4A3	4.133043	3.913817	0	0.935796	1.579399	3.628893	4.013459	2.567208	6.30328	5.574508	1.640777	2.455122	6.01292	6.79543	2.809275	2.092015	1.483087	0.782644
ZNF567	6.089623	5.779874	6.474142	4.12375	5.945978	5.538665	4.516222	5.115416	7.22639	6.819773	5.270546	5.953659	5.611748	6.536679	5.979485	6.249046	5.240581	5.030882
INTS6-AS1	4.2768	4.021913	6.222023	4.917157	4.162037	5.133936	4.516222	4.933558	2.378102	2.722119	3.661162	4.434598	4.133161	5.252738	2.504345	3.231384	4.174478	4.302122
ACOT1	5.0519	4.922058	4.139968	3.964133	5.349403	4.653256	4.618627	5.045399	5.649118	5.909157	5.290205	4.434598	2.420752	2.778659	4.683977	5.051725	4.023383	4.248461
SH3BP2	8.448646	8.183152	9.846127	9.451862	9.241958	9.628055	10.10577	9.969739	8.706853	8.921697	9.537116	9.977302	9.913212	9.60856	8.193703	8.059272	9.883431	9.541194
LOC101927	3.349371	3.530338	4.484237	5.626033	4.849916	5.582118	1.424024	3.203361	0	0	5.146613	4.706428	0	0	1.223673	0	4.244478	4.671834
SYP	6.798368	6.982732	4.322347	5.753573	6.991712	7.361579	5.732899	6.805406	4.103859	4.102086	6.081672	5.974383	7.431016	8.052285	4.646007	5.010588	4.43615	3.80375
CDHR5	7.627239	7.594847	8.160311	8.910521	7.553933	7.988714	9.446873	8.19225	1.999619	2.722119	9.357762	11.07402	8.731755	11.84721	6.612012	6.367822	8.198005	8.572087
MGMT	7.362981	7.418181	8.644296	10.49531	7.29548	8.273511	8.078592	8.4612	6.235992	6.376951	7.968287	7.774146	8.256516	7.801612	7.662819	7.769847	8.46316	8.028025
IL12A	1.505082	2.1757	0	1.902589	0	0	0	0	1.999619	1.802476	0.613139	0	3.517377	3.998289	0	0.552086	0	0
SPRY1	7.640592	7.085961	7.681678	6.174121	7.131257	6.113245	8.105294	6.993785	8.710989	8.757223	6.291296	6.866374	9.789474	10.23679	6.707275	7.252821	6.879033	7.718374
DBN1	7.815612	8.295109	6.267222	5.193964	3.314412	5.012136	5.371556	6.419093	9.74096	9.777194	4.505443	4.172941	9.813411	10.0772	6.839323	7.114058	6.811454	6.887596

TMX3	8.005973	8.536023	7.493336	7.410683	6.406839	6.510708	7.457727	7.267522	7.567796	7.154589	6.213552	6.396011	7.979593	7.973736	7.912119	8.343098	7.261504	6.764315	7.227963	6.982732	8.798507	7.408499	8.272444	7.780652	7.527343	7.755748	6.960657	6.970499	7.247277	7.487415	7.89412	8.094152	8.059321	8.094149	7.467242	6.720289
STK17B	7.207589	7.963902	7.845774	8.010027	8.81615	7.314695	7.936371	7.022627	7.959183	8.231178	7.846861	8.002044	6.933424	6.713735	6.791611	7.130343	7.498232	7.353836	6.890667	6.636702	7.257826	7.107916	6.849699	5.854372	7.616668	6.877702	8.253105	8.121183	7.277138	6.809841	7.37156	7.21145	7.61939	7.547015	7.422645	7.053235
GPR143	3.630002	4.162882	0	0	0	0	4.116654	4.318504	4.875281	4.447811	4.510256	4.917463	0	0	0	1.534489	3.140335	0	0	1.742164	1.755198	0	1.993739	2.741038	1.424024	0	2.677644	2.85209	0	0	1.455124	2.778659	0	0	6.131864	6.628748
KCNIP3	0	3.090277	2.504466	3.484125	5.546469	5.868776	2.254743	1.297421	6.2694	5.988015	4.134181	5.217548	0	0	1.324175	2.872407	5.582066	4.269784	1.909816	3.006495	2.523927	1.498706	1.579399	2.396196	0	0	4.806866	4.7616	3.777159	1.482042	3.900568	4.890416	1.223673	0.552086	2.675001	2.903526
ANTXR2	8.98059	8.968245	9.013299	9.360254	8.543968	7.614553	7.921482	7.784063	7.081715	7.256342	9.004146	9.10952	5.68492	5.441485	7.378759	7.423891	7.482552	7.744052	6.029989	6.396784	5.916313	4.872893	5.746689	5.012136	5.86377	5.74283	8.880301	8.667374	7.395308	8.183091	6.41747	7.327154	5.53617	6.194987	6.740554	8.182657
CAPN10-D	5.797702	5.657762	5.87408	5.311853	5.65957	5.220141	7.000082	5.925371	7.62811	7.166939	3.98335	4.823492	6.550765	7.483726	6.374384	6.356365	6.44278	7.099708	6.183842	6.180578	6.474142	6.858368	6.3316	6.823862	6.933097	6.794776	4.608323	3.74856	5.168022	4.549545	5.19798	5.695026	6.621826	6.415877	6.431029	6.618208
SDR16C5	2.968703	2.428861	4.051486	5.581157	8.151324	7.875122	1.174725	1.297421	4.034737	3.972596	1.85386	2.266453	0	0	0	0	3.140335	2.827144	0	0	0	0	0.995829	2.396196	1.424024	1.401742	5.596438	5.260129	0	0	0	0.723645	1.223673	0.950401	2.456453	3.56123
MED17	8.202701	8.006695	7.712799	8.054943	7.973145	8.56746	7.270667	7.643872	8.156379	8.155727	8.177341	7.980435	7.758928	7.641492	7.719686	7.589334	7.715018	7.317536	7.821487	8.211966	7.698276	7.270294	7.957464	7.694182	7.010478	7.17685	9.287206	9.062627	7.390762	7.657248	8.189235	8.193794	8.449317	8.381127	8.228552	8.550077
DMAC1	8.516942	8.091966	9.481041	9.024267	8.463176	8.840682	9.707322	10.00062	9.55122	9.494352	7.583987	7.156403	9.105903	8.789318	9.317186	9.219122	10.1798	10.45663	9.994915	9.65367	8.264296	9.913125	9.697289	10.06514	9.062358	10.15465	7.905182	7.856606	9.031136	8.256843	8.264709	7.95339	7.368549	7.853653	9.62262	9.715701
ZNF850	0	1.171698	4.299946	4.884194	6.490756	6.40317	4.841216	4.728633	3.884591	3.915185	7.060438	7.035871	5.169327	4.401863	5.378692	5.807583	5.45927	5.380055	6.607573	6.248908	3.686908	3.784633	5.121169	5.624301	5.043662	4.428113	6.346456	6.155652	4.723561	4.656005	1.853078	3.513692	5.103713	4.924617	4.244478	4.862499
UPF3A	7.516398	7.532602	7.204222	7.558376	6.172832	6.249243	8.586978	8.550551	8.685337	8.111685	7.262301	6.904343	9.069226	8.846732	8.348027	8.121914	8.209953	8.340402	8.626944	8.289713	8.23046	7.5417	8.69557	8.953147	8.340737	8.707773	6.867393	6.738062	7.353873	7.820667	6.941647	7.043533	8.378721	8.418956	9.056521	8.724803
SMN1	4.702501	4.162882	4.409778	3.484125	2.604499	2.008245	1.813523	0.789892	0	0	0	0	4.667222	3.432632	3.429659	3.770376	2.295325	2.827144	0	0	3.392717	3.340484	3.799599	1.942141	2.951088	3.545268	1.485101	1.519108	2.770919	1.482042	3.143737	2.778659	3.060865	2.998749	5.018839	3.56123
ABCB9	7.603397	6.747993	5.795315	6.003993	7.365344	7.607116	7.198394	7.116559	7.34872	6.821193	7.544122	7.325918	6.760169	6.750666	4.922482	5.027432	7.022204	7.078172	6.479676	6.653605	5.371118	5.801591	6.499533	6.357878	6.851329	6.162186	8.165406	8.038268	6.158553	6.034827	7.059364	7.014755	6.499402	6.176508	5.371486	6.132309
DYNC1I1	0	2.046811	2.041335	0	0	0	1.174725	3.486101	1.389562	2.560629	3.280294	2.878198	0	0	2.002808	1.534489	0	0	2.225526	1.742164	0	0	0	0	0	0	6.388377	5.800642	4.165646	0.923506	0	0	0.737862	0	1.884869	3.56123
ULBP3	2.968703	3.885616	2.854499	3.194075	5.289596	4.332364	3.293148	4.688413	1.389562	1.786523	4.80829	4.917463	0.858862	0.626961	0	1.534489	2.295325	1.600378	3.349371	3.20301	0	2.217783	3.799599	1.275828	1.424024	0.864812	3.999524	4.202988	2.878727	2.673628	4.807247	4.360398	3.971724	1.924946	2.198785	4.07435
GALNT5	4.346738	3.757841	4.299946	3.72551	8.176883	7.979606	6.516328	7.894256	6.897959	6.158508	3.144696	2.604511	6.165586	4.021536	4.647449	4.225275	3.669699	4.97652	5.635691	6.084126	6.721536	7.078983	6.773082	6.454571	7.360039	6.486699	3.631802	3.368864	7.042286	5.845322	0.903733	0.723645	6.960291	6.99783	7.384398	7.605521
ZBED6CL	7.240534	6.764711	6.41175	6.049094	4.917449	5.965003	6.392229	7.04669	6.827684	5.945635	6.279782	5.546483	7.390507	7.425533	9.04471	9.379735	7.106459	7.2803	5.27379	5.509757	7.014458	8.168245	7.951682	8.149006	7.514119	8.011255	5.944359	6.217258	7.099117	7.192475	6.01292	7.092553	8.460299	8.026114	7.793388	7.374556
LRP5	10.26189	10.52331	10.71882	9.988563	10.47177	9.978919	11.56824	11.45579	11.59883	11.67601	11.67093	12.05865	10.78736	10.78687	10.80079	11.04712	10.40652	10.39264	10.83417	10.81745	9.987883	9.288761	10.41056	10.77607	11.07166	10.84248	11.70383	11.78489	10.53099	10.50015	10.25091	10.40494	11.34635	11.0886	11.34724	11.84444
MBD2	10.31053	9.692091	10.55516	9.803543	8.189495	8.319288	9.551648	9.24843	9.834558	9.656411	8.471069	8.277788	9.870109	9.701658	9.069151	9.003241	8.864584	9.268576	9.083243	8.76062	9.121326	9.421453	9.660542	9.615039	10.69177	10.64579	7.56021	7.883458	9.04422	9.007924	9.572313	9.255771	10.11815	9.969253	9.632444	8.479048
RPS6	13.99843	13.87494	14.84145	14.65062	13.96851	13.57579	14.49537	14.25492	15.1151	14.85288	12.56946	12.71571	14.09896	14.01351	13.31496	13.87092	14.93178	15.00137	14.39392	14.28788	14.7556	14.83799	14.44337	14.46781	15.16617	14.86757	12.89639	13.01755	13.80456	14.19043	13.50493	14.02612	13.55223	13.80419	14.88191	14.56037
RIPK3	4.499719	4.867266	5.577246	5.45278	6.530952	6.78567	7.771801	7.545977	7.341533	6.969572	3.518364	5.335724	5.590637	5.77838	4.558172	6.047134	7.279763	7.222578	7.271529	7.357577	6.267222	6.040435	7.046992	7.203611	7.220456	7.101219	3.56025	3.608434	5.795641	6.689662	6.460907	7.887455	6.445158	6.351441	7.005376	7.173287
PLXND1	7.611815	8.230794	8.063074	7.649056	9.796113	9.592169	7.028152	6.368802	7.549199	6.852115	9.610863	10.15398	4.110374	3.503585	8.016352	7.860569	7.529089	7.3358	5.373125	5.080449	7.212327	7.454279	4.162037	4.569372	3.676189	4.053193	9.400373	9.192542	5.666214	4.549545	8.340313	7.664755	7.638854	7.203478	4.849483	5.210001
TNNT1	0	3.377921	0	0	9.834761	8.934288	4.637637	2.609429	4.56559	4.276026	5.80498	7.855423	3.967622	2.804754	2.002808	3.47464	1.562827	2.827144	2.484401	1.119477	1.755198	3.05373	0	0	2.951088	1.79215	4.743672	4.509164	2.234835	1.883682	6.19544	7.692277	3.546369	3.367943	2.675001	1.956475
FSCN1	9.951239	9.064217	12.92077	11.64506	10.96994	10.3833	6.476136	7.04669	11.86505	12.28254	12.01268	11.77386	10.87934	9.502781	4.003509	4.015681	9.322071	9.319941	5.811271	6.314148	1.755198	2.885695	2.578004	1.942141	7.591706	3.902917	11.63856	11.7644	4.96284	4.242936	7.141749	5.913435	11.09301	10.94587	6.627209	5.41874
ZNF236-DT	2.968703	4.05828	1.35509	0	1.824117	2.709739	3.622635	3.389056	3.18586	3.348635	2.421632	2.062176	2.743137	4.640943	3.212791	3.300417	2.295325	3.72174	3.793777	3.913817	3.686908	3.964133	3.99217	3.785802	4.405989	3.821469	1.765176	2.039219	3.243874	3.181178	3.403254	3.338979	4.145071	3.231384	2.675001	3.646681
JCAD	4.702501	4.21247	4.051486	1.603113	5.335711	5.486471	0	0.789892	0	1.504553	0.904236	2.062176	2.341426	0.626961	0	0	0	2.340407	0.940508	1.119477	0	0	0	0	0	0	3.887052	3.279269	0	0	2.420752	2.778659	3.546369	3.157929	1.884869	3.373461
AIFM3	5.504114	7.103828	5.320242	5.859558	6.801278	6.550024	10.1408	9.455832	8.908445	8.36047	4.97748	6.449421	6.844297	8.331552	8.033015	8.755618	9.197945	9.048157	8.898292	8.58231	6.267222	5.486115	9.022329	9.382897	9.905108	9.2668	3.631802	2.85209	8.099429	8.536616	5.091455	5.562114	8.05208	7.657741	9.558797	8.80366
PXDN	3.981368	3.377921	4.780742	4.418177	4.917449	4.534069	1.174725	0	0	1.504553	3.901577	4.498115	0.858862	0.626961	2.647	0.706894	3.875805	4.269784	0.940508	0	3.686908	5.298061	0	0	1.424024	0.864812	10.31283	9.790429	0	0	0	0	0	0	4.100908	2.595018
TMEM42	5.855558	6.480912	6.84227	6.56601	7.188158	6.905709	7.383616	6.785836	8.259124	7.61295	5.994396	6.470243	6.659263	7.230169	6.625756	7.070521	7.297795	6.917687	6.289339	6.376564	8.442347	7.913515	7.151576	6.859617	7.029192	6.762407	5.20896	5.394206	7.372435	7.657248	6.889614	7.564073	6.024574	6.53671	6.640252	6.438184
GS1-124K5	4.76422	4.478779	2.854499	2.830649	3.636225	4.534069	3.293148	3.28501	6.652909	5.916674	3.144696	3.636238	3.533175	4.245052	4.886259	5.135183	4.491695	5.695038	4.638033	4.466515	5.971978	5.936741	3.99217	5.191185	5.371556	5.30713	2.80692	1.519108	5.556853	4.603757	3.62313	3.810133	3.843386	4.363213	4.023383	3.80375
TM7SF3	9.966089	9.742544	10.30629	9.938504	9.21813	9.89743	7.780078	8.96252	7.906582	7.887498	10.57162	10.29487	9.698494	9.226881	9.323973	8.701867	8.256795	8.518853	8.909366	9.108945	9.199193	9.477955	9.938561	9.469329	8.399453	9.127606	10.69907	10.19973	9.860917	9.677381	8.930244	8.383011	9.171326	9.31493	7.872892	8.524907
MKKS	8.99675	8.769296	9.173698	8.850448	7.308666	7.798836	8.432769	8.326461	8.08931	8.242892	8.390928	8.198895	9.826821	9.747143	9.656583	9.410891	9.765988	10.10119	8.448646	8.383885	8.184075	9.409882	9.438741	9.509271	9.071414	9.460834	8.009603	8.336371	9.079953	8.873713	8.332539	7.926604	8.687568	8.688805	9.669496	9.270437
MAP4K4	10.21845	10.07297	9.490447	9.300892	8.973921	8.653007	6.132626	8.235179	8.805124	9.320077	9.309748	8.975756	8.325294	7.870142	8.444601	7.703141	8.430288	8.648712	8.164265	8.278859	8.798507	9.075847	7.068524	6.710952	6.972306	7.543567	10.37279	10.10186	7.896355	7.428556	9.106979	9.07418	8.015321	8.108842	8.054365	8.591074
GZMM	0	0	2.041335	3.932237	3.306431	1.328528	1.174725	1.297421	0.855986	0	3.144696	2.445361	1.393533	0	1.324175	2.439889	1.562827	0	0	0	0	0	0	0	1.424024	0	3.321471	2.242534	0.613139	1.482042	2.420752	3.998289	0	0	3.182626	0
ELF3	8.612324	9.274355	10.92827	11.66963	12.71215	11.13089	11.90836	11.36859	12.09479	11.96789	9.90383	10.06694	11.95809	12.21951	10.91588	11.33533	11.0294	10.78875	11.6802	11.19549	11.08034	10.49713	11.66319	11.80633	13.48234	11.84595	9.652338	9.902502	11.217	11.74778	11.13828	11.21104	11.95097	11.901	11.77661	11.60791
PLAAT5	2.968703	2.046811	0	0	1.824117	0	0	0	0	0	5.586882	5.033933	2.088965	1.8959	0	0	0	0	0	0	0	0	0	0	0	0	3.137054	2.039219	2.878727	1.883682	0	0	2.32372	0	0	0
TMEM230	8.66181	8.56733	8.457707	8.307824	7.53306	7.808596	7.965695	7.950606	8.591515	8.4364	8.431555	8.372083	10.02346	9.833162	8.581161	9.16135	9.113933	9.609871	8.653533	8.505251	9.199193	8.360836	9.297721	9.157277	9.002062	9.339914	7.640463	7.848841	9.342543	9.490394	8.820582	9.002704	8.957401	9.092739	9.01206	8.722344
CUEDC1	6.64401	7.383631	6.782267	6.626776	9.132548	9.214313	7.44737	7.204748	7.680317	7.460763	8.092359	8.391316	4.712695	5.025466	7.436262	7.906915	8.718291	8.486521	6.146889	6.314148	6.311049	5.626033	5.898676	6.083315	6.324027	6.111689	8.911185	8.967234	6.895996	7.413458	6.178193	6.992789	7.064719	6.309656	7.304721	7.8458
NR1I2	6.137939	5.693125	7.204222	8.530892	3.108141	2.178475	9.869318	9.518831	9.393361	8.964454	5.586882	5.52674	4.841099	6.478615	6.163561	6.835156	8.718291	8.794499	8.867391	8.717061	8.958631	7.945925	8.994478	8.705507	9.521466	8.406393	0.677882	0	8.663922	9.602808	8.136632	8.886393	8.457561	8.272216	7.445116	7.323569
ZNF792	4.987659	5.63975	5.428596	6.656225	6.294575	6.277628	4.962547	5.976372	5.163089	6.055994	7.635484	7.414531	5.48976	5.477181	5.934418	6.216167	6.195936	5.307877	5.988818	6.686828	4.761976	4.267454	6.934229	5.818486	4.968088	5.182192	7.09331	6.811808	5.710659	5.776187	3.900568	4.792091	5.932941	6.129248	5.339857	5.237806
CD96	0	0	0	0	0	0	1.174725	0.789892	0	0	1.85386	0.709495	1.393533	0	0	0	0	1.600378	0.940508	1.119477	0	0	0	0	0	0	5.780861	5.112303	0.613139	0.923506	1.455124	1.203326	0.737862	0	0.924276	0
KDSR	8.219323	8.935397	8.176194	8.811714	7.859361	7.432984	7.478221	7.517562	8.160469	8.108487	6.992941	6.490769	8.060963	8.473075	8.3939	8.657376	7.418072	7.011566	7.779854	7.963524	8.992671	8.828878	8.290881	8.020651	7.65332	7.40366	7.143134	7.351858	7.633249	8.091202	8.487599	8.751178	8.441026	8.541349	7.619893	7.894651
PPP1R3D	4.499719	4.05828	3.751148	4.549533	6.6268	6.580724	7.796492	7.153962	6.878227	7.097666	5.128895	5.846383	6.09865	6.165109	6.425763	6.252514	7.4667	7.242076	6.77434	6.900154	6.753654	5.120144	6.141511	6.382666	6.262092	6.348162	3.699971	3.680197	6.754567	7.110593	5.054124	5.581868	6.650875	6.836527	6.150216	6.720289
RASGEF1A	0	0	0	2.830649	7.905957	7.093051	0	0	0	0.68926	2.165499	2.445361	0	0	0	0	0	2.827144	0	0	0	0	0	1.275828	2.596932	0	5.255006	4.947582	0.613139	2.863351	9.322471	9.197744	0.737862	0	0	0.782644
LOC101927	2.142139	0.701667	1.35509	0	0	1.005502	3.467277	2.609429	3.527314	2.224885	1.455811	0	3.533175	3.279313	2.810563	2.74213	0	0	1.909816	0	2.523927	3.784633	2.315249	3.019148	1.424024	3.545268	0	0.697815	2.527635	0.923506	1.455124	1.203326	0	0.950401	1.884869	2.202074
EVA1A	2.473063	1.171698	0	0	6.340534	6.837435	4.00809	5.386154	6.796486	7.004399	0	0	7.273997	5.875207	7.176144	6.153476	3.140335	2.827144	6.146889	5.582174	0	0.935796	7.171613	6.823862	5.86377	6.36263	0	0	6.482857	6.073761	0	0	3.62655	2.998749	7.288244	7.975319
PP7080	7.394839	6.929459	6.562057	5.45278	7.441129	7.458004	7.44737	6.455237	7.940277	7.963805	6.343105	6.374081	9.293419	10.88148	7.735044	8.340287	9.382459	8.884099	8.437214	8.604212	7.926736	7.763996	7.726395	8.134485	7.628989	7.56249	4.925509	4.79431	7.121238	7.494606	6.160737	6.803865	7.723329	7.359061	8.29191	8.032
MAP3K15	0	1.171698	0	0	4.790744	2.916414	0	0	0	0	0	2.266453	0.858862	0.626961	0.80896	0	0	0	0	1.742164	0	0	0	0	0	0	2.535633	2.722119	0	0	4.203045	4.95243	1.586456	0	0	0
BCR	9.233361	9.139854	9.455654	9.360254	9.810473	9.587453	9.20337	9.757439	9.194306	9.699556	10.17577	9.727518	9.257432	9.561118	10.06781	9.322471	7.498232	7.538636	9.442774	9.741274	8.635705	8.751535	8.773025	9.128256	8.122825	8.484539	10.68652	10.7661	8.521491	8.147035	9.312625	9.175164	8.615717	8.63906	8.344656	8.898165
OSR1	4.638023	5.446059	7.78592	7.427861	6.490756	6.14011	5.127322	3.819772	5.1301	5.306872	5.638277	5.862224	1.393533	1.667771	4.003509	3.835722	2.778832	0	3.062403	3.006495	2.523927	0.935796	2.578004	0	2.12653	3.735147	8.100682	7.975472	5.328737	5.095022	3.900568	3.140156	1.223673	2.615475	3.032428	3.470398
IL32	5.087613	4.737779	10.57445	10.09724	9.293086	9.337396	9.92263	8.875633	9.680739	9.710144	4.715622	5.506723	10.38735	10.22108	4.132812	6.047134	10.18223	10.68572	8.068715	7.929231	8.264296	9.501769	9.128652	9.803526	11.4029	10.74507	2.201237	1.166132	7.873731	7.327472	5.359711	6.893535	8.316628	8.175497	8.619518	7.799907
ANKEF1	6.99236	7.097243	6.41175	6.795078	5.730327	6.277628	6.434793	6.592336	6.581494	6.681839	6.461976	6.025879	8.203162	7.568906	7.083199	6.858926	7.064946	7.034111	5.946437	5.779874	7.212327	6.313124	7.874325	7.515198	6.808639	7.676898	5.780861	5.953277	7.377039	7.013807	6.744831	6.306735	7.741448	7.738695	6.799877	7.540029
NLRP11	3.164525	1.809634	1.35509	0	1.824117	1.005502	0	0	0	1.153748	1.85386	0.709495	1.393533	0	0	0.706894	0	1.600378	1.909816	1.119477	0	0	1.579399	0	0	0	3.887052	3.608434	0	0	0	0	0	0	0	1.660309
KRTAP5-A	0	0	3.37139	3.932237	4.23161	2.709739	4.903157	4.26481	3.18586	3.25918	0	2.266453	3.428046	1.667771	4.132812	4.273239	4.216369	4.109178	3.97336	3.375938	4.761976	1.498706	3.899095	3.452812	5.371556	3.203361	0	0	4.122433	5.518408	1.853078	3.669513	4.987063	4.657791	0	3.269538
GRTP1	7.452218	8.191149	8.358465	8.191953	7.665348	8.044364	8.411615	7.853913	9.506935	9.257933	8.078471	8.346971	9.300882	9.257996	8.406743	9.055608	8.592462	8.486521	9.005388	8.821719	8.412481	8.257028	8.580383	8.818827	9.133267	9.259087	7.086959	7.231615	8.468553	9.128397	8.13216	8.295994	8.699202	8.62209	9.056521	8.986859
VPS16	8.79345	8.010204	8.751692	7.82792	6.881193	7.160653	8.524304	8.41231	8.058958	8.300078	8.553699	8.511704	9.382286	9.060366	9.20586	8.990744	8.846323	8.985912	8.117281	7.783271	8.207454	9.30551	8.901557	9.217075	8.963664	9.482524	7.137	7.575342	8.918806	9.042683	7.767219	8.250435	8.122902	8.091685	9.262449	8.77785
CGAS	0	1.171698	7.234392	7.32159	8.009163	8.077032	1.174725	0	1.389562	2.224885	7.115881	7.049771	6.57559	5.595588	4.069608	4.319659	6.861769	6.432183	4.583765	4.12247	5.371118	4.334272	4.079602	4.807673	3.676189	2.754399	8.306012	8.651839	6.329798	6.473762	1.455124	1.562693	7.162096	7.180478	1.483087	1.660309
MARK1	0	3.288264	0	2.830649	1.824117	0.588633	2.592156	3.662562	0.855986	2.402502	0	0	0.858862	2.266084	7.067109	7.788134	2.295325	0	5.967783	6.670312	6.07725	3.579584	5.719725	5.246249	5.777855	5.276895	0.677882	1.519108	5.635805	5.518408	4.269699	6.047341	1.586456	0	3.318652	0.782644
PMFBP1	4.934959	6.152891	4.299946	2.830649	2.878187	2.008245	6.555431	4.98257	6.299384	5.306872	3.624124	3.306443	6.349561	6.849308	6.251621	6.228385	6.909457	6.498538	6.289339	6.942033	5.735143	3.05373	7.057798	7.203611	6.830142	6.773278	3.405533	3.813829	5.767866	5.305931	3.517377	4.313908	1.876099	2.241729	7.833688	7.954524
KBTBD7	0	0.701667	5.529378	6.092828	6.094529	6.087637	6.684501	6.645826	6.075097	6.288533	5.638277	5.167427	6.378065	7.05227	6.830258	6.665811	7.333195	7.05631	7.192137	7.696217	6.435064	4.872893	7.089739	6.671263	6.851329	6.836835	1.137227	0	6.394801	6.809841	5.78125	6.047341	6.08258	6.558282	6.369703	6.690416
ST3GAL1	3.981368	4.306808	0	1.603113	10.4653	9.685162	4.483975	4.090583	6.917424	7.30744	3.814889	3.841959	0.858862	2.687751	0.80896	0.706894	7.5593	7.44078	4.468693	3.669801	0	0	2.992693	4.653256	0	1.401742	7.823499	7.697101	2.062641	2.455122	4.714581	5.581868	5.336085	5.977399	0.924276	2.202074
BIVM	7.38505	7.636168	7.043115	5.957437	6.47023	6.752348	7.614063	7.360047	8.28183	7.626428	5.9544	6.654369	8.199174	8.605984	7.318868	7.860569	7.544274	7.60037	8.236395	8.123737	7.778516	7.164097	8.126102	8.134485	8.199178	8.451081	6.759718	6.738062	6.718746	7.719952	4.714581	5.324845	6.467101	6.770293	7.467242	7.361977
CYB5A	10.63146	10.2841	11.2366	11.58316	9.002792	9.492767	9.433307	9.133294	9.55122	9.784164	8.946695	8.881746	11.84331	11.92073	10.59806	10.72321	9.586931	9.324521	9.767334	9.619665	9.777491	10.45834	10.15152	10.34603	10.16831	10.33146	9.224942	9.197136	9.962429	10.65018	10.08268	9.283178	10.68786	10.64225	9.236918	9.033214
TTC32	6.292554	6.460534	6.213973	5.754553	5.335711	4.964827	6.232515	5.907962	6.983556	6.749097	4.510256	5.312851	5.729847	6.165109	6.658341	7.117262	7.401491	6.791036	5.834697	5.953719	7.165345	6.892026	6.3493	6.545189	6.354026	6.525793	5.232167	5.059458	5.230407	6.014959	4.563366	5.459073	6.151956	5.923011	6.764577	5.601059
CDKN1B	8.543587	9.706224	8.120743	9.046679	8.226682	7.821506	8.529223	9.1239	7.93073	9.03015	7.868544	8.026853	8.914849	9.401917	8.152206	8.670868	8.963914	8.914797	9.944664	9.869791	9.210808	8.159059	9.388263	9.569885	8.804397	8.768794	7.480084	7.648729	8.637306	8.47977	10.34375	10.59372	8.289176	8.243653	9.075851	9.468249
SWSAP1	6.137939	5.446059	5.320242	4.980469	4.65183	5.160422	5.968492	6.104168	5.632261	5.514416	3.901577	3.841959	6.486765	6.039417	5.091095	5.459282	5.521974	6.530605	6.146889	5.617063	5.527909	6.715286	5.664236	6.05275	6.293392	6.333548	4.867406	5.032292	5.456028	5.238996	5.9122	5.695026	4.924998	4.458067	6.489855	6.596896
PRDM16	2.473063	4.306808	6.538068	6.534639	7.52313	6.81824	2.865422	1.969301	2.084058	2.02229	7.031897	7.823413	2.088965	3.013642	1.324175	1.179238	3.140335	3.190442	3.588618	3.530338	0	2.217783	1.579399	1.942141	5.115474	2.920068	7.384927	7.301547	6.179787	6.336426	6.294807	3.513692	1.586456	2.377357	2.456453	1.287213
ITGA10	6.559498	5.147757	6.538068	6.366658	6.42828	5.368268	2.865422	3.577028	4.515377	4.132274	5.199032	5.953782	3.809178	3.921438	5.213095	4.880336	3.875805	1.600378	2.703815	2.508614	4.62978	3.685748	4.240015	4.385382	2.596932	3.545268	6.516774	6.366112	2.770919	2.673628	5.686843	5.324845	1.223673	2.377357	3.032428	2.411883

Gene	logFC	AveExpr	t	P.Value	adj.P.Val	B
SHISA6	-6.89870189	1.950197	-16.3044265	6.64E-11	1.44E-06	13.2263035
F5	-5.98865649	2.96825621	-11.6081004	7.35E-09	4.56E-05	9.8886489
FRMD6	-5.37781882	1.88885254	-11.555572	7.81E-09	4.56E-05	9.84094619
LOC440173	-2.19996893	0.54999223	-11.4904469	8.43E-09	4.56E-05	9.78141911
TFAP2C	-4.17150142	1.15926421	-10.40154	3.18E-08	0.00013774	8.71975856
ABCB1	7.26216532	7.71477681	9.33979736	1.29E-07	0.00046666	7.5526942
GLI2	-5.05003206	1.41809754	-9.17754864	1.62E-07	0.00050072	7.36180863
AQP5	-8.22905877	4.18878574	-8.98657014	2.12E-07	0.00057282	7.13263306
MBD1	1.19900121	9.2159608	8.82246496	2.67E-07	0.00064315	6.93175532
MPP1	5.69004741	5.27115897	8.68177849	3.27E-07	0.0007089	6.75658619
LINC00909	2.32291245	6.06656678	8.59717004	3.70E-07	0.0007288	6.64990754
GABRE	4.76709454	7.26087774	8.41194588	4.86E-07	0.00087706	6.41282585
CTDP1	1.3251893	8.42809785	7.96245828	9.57E-07	0.0015943	5.81681014
LINGO1	-5.12275412	2.02857441	-7.85985378	1.12E-06	0.00165937	5.67655876
LINC01234	-5.45129611	2.75309823	-7.76700857	1.30E-06	0.00165937	5.54827785
SHLD1	1.82622551	5.75168719	7.74973831	1.33E-06	0.00165937	5.5242718
PRDM8	-6.56391285	2.91625888	-7.74664245	1.34E-06	0.00165937	5.51996369
ISX	6.06143201	6.61193395	7.54846766	1.83E-06	0.00208196	5.24113641
TXNL1	1.12007982	8.90262	7.47880838	2.04E-06	0.00218251	5.14169027
HOXC13	-2.9199932	0.95424778	-7.4560739	2.12E-06	0.00218251	5.10907146
SHROOM2	-4.86148093	2.47773826	-7.40375614	2.30E-06	0.00220459	5.03370195
NOS3	3.73482395	6.18544548	7.39307771	2.34E-06	0.00220459	5.01826608
FGF1	-1.53523411	0.45122185	-7.31472762	2.66E-06	0.00239736	4.90446439
CAB39L	3.72376131	7.69951067	7.13895401	3.54E-06	0.00306496	4.64564891
PRDM16-DT	-3.42034115	1.41036369	-7.02355195	4.28E-06	0.00356491	4.4730705
AOAH	5.97428541	5.890468	6.95379118	4.81E-06	0.0038548	4.36771982
CLN5	1.55983309	7.23625125	6.91869144	5.10E-06	0.00394135	4.31441983
SMAD4	1.20166919	8.02901666	6.8697541	5.53E-06	0.00406092	4.2397786
PNMA2	-5.68896275	2.70065466	-6.85966334	5.63E-06	0.00406092	4.22434016
DKKL1	-1.40472307	0.42275256	-6.78677905	6.36E-06	0.00435374	4.11234611
KIRREL3	-4.42294272	2.02836833	-6.78001836	6.44E-06	0.00435374	4.10191449
AOC1	5.31877341	7.77930321	6.76105974	6.65E-06	0.00435936	4.07262261
ELAC1	2.19664696	5.72628457	6.7067342	7.29E-06	0.00463957	3.98836799
IRX3	-4.45438075	1.60816458	-6.67766414	7.66E-06	0.0047356	3.94308801
UBASH3B	-5.97067964	3.2642889	-6.54886543	9.55E-06	0.00558527	3.74083538
WNT7B	-4.08348349	2.44680818	-6.52276863	9.99E-06	0.00568845	3.69953059
SAMD13	3.91537875	3.39999914	6.49776932	1.04E-05	0.0057871	3.65986012
SHISA2	-5.02188865	1.4938009	-6.47502145	1.08E-05	0.00586892	3.62367503
ADAM19	-3.28890638	1.22375277	-6.42089665	1.19E-05	0.00628966	3.53724404
SOX8	-5.54826487	2.51156081	-6.36706727	1.31E-05	0.00674381	3.45081768
PKIB	3.43821249	4.91253883	6.32617079	1.41E-05	0.0070755	3.38484467
RBM26-AS1	2.46494119	5.00974016	6.31211371	1.44E-05	0.00708722	3.36210618
PRODH	-5.03539624	5.45211088	-6.29186137	1.49E-05	0.00715913	3.32929058
WWC2	-4.13612098	2.42289182	-6.25821084	1.58E-05	0.00725109	3.27461995
AKAP12	-4.13322762	1.94277935	-6.24963249	1.61E-05	0.00725109	3.26065403
GJA3	-3.33895597	1.62016038	-6.2259285	1.68E-05	0.00725835	3.22200159
STX18-AS1	1.47224129	4.61328246	6.19519879	1.77E-05	0.00726806	3.17175888
PLEKHO2	-2.37297171	5.17702106	-6.18181207	1.81E-05	0.00726806	3.14982451
GSTM4	2.59663933	7.80176225	6.18166875	1.81E-05	0.00726806	3.14958953
ZNF772	-4.35769452	2.28250738	-6.1443299	1.94E-05	0.0076248	3.08825697
SFMBT1	-1.07303603	7.28035897	-6.08266708	2.16E-05	0.00835819	2.98648296
ECE1	-1.44779798	9.43524749	-6.03007048	2.38E-05	0.00902193	2.89919433
ME1	-1.24489659	8.66588911	-5.98273623	2.59E-05	0.00948971	2.82026365
PLD1	-2.8009986	6.52369196	-5.98273008	2.59E-05	0.00948971	2.82025337
DACT2	-5.05538336	2.96638477	-5.94964371	2.75E-05	0.00990477	2.76487068
THUMPD3-AS	2.00322489	7.37085714	5.93192536	2.83E-05	0.0099872	2.73514116
NUAK1	-4.26809332	3.79197153	-5.92689401	2.86E-05	0.0099872	2.72669007
PCLO	5.41092554	4.85086658	5.81996199	3.47E-05	0.01176059	2.54613679
TIAM1	-5.9460642	3.18615053	-5.80530469	3.57E-05	0.01176059	2.5212486
DUSP6	-3.05121781	10.0715333	-5.79943905	3.61E-05	0.01176059	2.51127931
KRTAP5-1	3.77545401	4.91732938	5.77754158	3.75E-05	0.01176059	2.47401487
IDNK	1.85978011	5.54502149	5.77649964	3.76E-05	0.01176059	2.47223987
SNHG11	1.12576917	7.53546332	5.7595998	3.88E-05	0.01176059	2.4434265
SH3BGR	1.53378955	4.53038239	5.7532041	3.92E-05	0.01176059	2.43251061
CNDP2	1.46954999	10.5249676	5.75268497	3.93E-05	0.01176059	2.4316243
ANXA13	3.86434144	3.40806077	5.75197733	3.93E-05	0.01176059	2.43041608
GPR162	-2.29991572	1.08228759	-5.74574138	3.98E-05	0.01176059	2.41976553
HS3ST3B1	-3.99820158	2.26064427	-5.73987058	4.02E-05	0.01176059	2.40973313
QKI	-5.65438155	2.79729875	-5.72746155	4.11E-05	0.01187083	2.38851021
NUAK2	1.90793219	7.52137226	5.71824457	4.18E-05	0.01191446	2.37273117
RRAGA	1.22145813	8.85012019	5.69806885	4.34E-05	0.01207733	2.33814551
MIR4458HG	4.06836452	5.02161117	5.68682401	4.43E-05	0.01207733	2.31884211
ANO9	2.68395399	10.6151688	5.68295139	4.46E-05	0.01207733	2.31218968
PIAS3	-1.36045649	7.80203461	-5.67348184	4.54E-05	0.01213802	2.2959131
KLF12	-4.69001395	2.5987819	-5.66295154	4.63E-05	0.01220912	2.27779707
CHST15	-5.23040109	2.0438753	-5.65658433	4.69E-05	0.01220912	2.26683485
ANO10	2.15842191	7.26964566	5.64363132	4.80E-05	0.01220912	2.24451489
ZNF566	-1.59489699	5.72962267	-5.61020436	5.10E-05	0.01261666	2.18679672
CXCL17	-3.26195032	1.35880461	-5.59102844	5.29E-05	0.01272156	2.15360884
SALL4	-4.20985135	1.83124486	-5.57235436	5.48E-05	0.01302502	2.12123581
MYO5B	1.32982143	9.21070332	5.53734451	5.84E-05	0.0137492	2.06040107
ASCL2	3.00896507	10.6410507	5.5070282	6.18E-05	0.01423815	2.00757276
LOC10192867	-1.26812215	0.36812549	-5.43235609	7.11E-05	0.01619831	1.87686484
GNG7	-4.12880049	1.73414798	-5.42074031	7.27E-05	0.01638248	1.85645782
CXXC1	1.11710554	9.4191657	5.40783388	7.44E-05	0.01661102	1.83375995
OSBPL10	-1.38118865	8.01006792	-5.37563634	7.91E-05	0.01742214	1.77702897
TEX30	4.32849288	6.62864654	5.3150353	8.87E-05	0.01890606	1.66984114
STARD5	1.82678696	6.05604323	5.30868937	8.97E-05	0.01890606	1.65858598

LOC10192810	-2.78739745	1.53882211	-5.30727298	9.00E-05	0.01890606	1.65607309
SCN1B	-3.07125459	0.98177489	-5.27025925	9.65E-05	0.01991119	1.59030237
PCDHGA4	-1.17682644	0.29420661	-5.26974517	9.66E-05	0.01991119	1.58938751
TRIB2	-5.61893883	3.08197761	-5.25060735	0.00010015	0.02026117	1.55530262
AQP2	-3.44316715	1.94809312	-5.24007176	0.00010218	0.02047908	1.53651623
SLC4A3	-3.75777976	3.0065658	-5.23126536	0.0001039	0.02055907	1.52080113
ZNF567	-2.04623448	5.72748846	-5.18923623	0.00011255	0.02137104	1.44564886
INTS6-AS1	1.87033795	4.22923893	5.16522358	0.00011783	0.02189936	1.40260058
ACOT1	-1.36411603	5.00467304	-5.16466968	0.00011795	0.02189936	1.40160664
SH3BP2	1.24680628	9.22897211	5.16281115	0.00011837	0.02189936	1.39827129
LOC10192788	4.40583245	3.30437434	5.15738031	0.0001196	0.02194008	1.38852228
SYP	2.10361367	5.57711646	5.12856354	0.00012638	0.02298756	1.33672428
CDHR5	4.3042722	7.09021206	5.11895465	0.00012872	0.02307851	1.31942689
MGMT	4.45762972	7.28263203	5.10263695	0.00013281	0.02356348	1.2900236
IL12A	-1.57842949	0.69279706	-5.09168709	0.00013562	0.02386769	1.27027237
SPRY1	-1.65708645	7.37955421	-5.08347686	0.00013778	0.02405102	1.25545212
DBN1	-3.79674429	6.47669661	-5.05503582	0.00014551	0.02519803	1.20404271
TMX3	1.23868987	7.5641969	5.04985183	0.00014697	0.02524843	1.19466049
STK17B	-1.12077543	7.21521687	-5.04470006	0.00014843	0.02529905	1.18533298
GPR143	-2.95198905	1.52537127	-4.99548771	0.00016319	0.0267614	1.0960534
KCNIP3	-3.27554818	2.27338778	-4.98900989	0.00016525	0.02682975	1.08427763
ANTXR2	-2.69349491	6.89521402	-4.97430629	0.00017001	0.02725902	1.05752806
CAPN10-DT	2.10062314	5.86639852	4.93420081	0.00018372	0.02847998	0.98442266
SDR16C5	-3.2260709	1.32466703	-4.93296172	0.00018416	0.02847998	0.9821607
MED17	-1.07981933	7.81703762	-4.92347137	0.00018758	0.02847998	0.96482945
DMAC1	1.6141531	8.83615942	4.92322235	0.00018767	0.02847998	0.96437453
ZNF850	-1.830786	5.27651452	-4.92317654	0.00018769	0.02847998	0.96429085
UPF3A	1.39279413	7.98762036	4.92191609	0.00018815	0.02847998	0.9619881
SMN1	2.45929355	2.59552225	4.91440761	0.00019091	0.02869693	0.94826645
ABCB9	-1.71028129	6.48571732	-4.8922912	0.00019928	0.02954501	0.9078072
DYNC1I1	-3.86800701	1.68587244	-4.88700078	0.00020134	0.02964708	0.89811983
ULBP3	-2.96917364	2.25518615	-4.88148821	0.0002035	0.02976383	0.88802191
GALNT5	2.88106225	5.34826486	4.86161858	0.00021152	0.03072855	0.85159298
ZBED6CL	1.86476656	7.39554535	4.84758908	0.00021737	0.03136863	0.82584155
LRP5	-1.23534821	10.8780622	-4.8285357	0.0002256	0.03212655	0.79082958
MBD2	1.52288767	9.19029692	4.8171599	0.00023066	0.03255552	0.76990435
RPS6	1.51954191	13.9394349	4.81503039	0.00023162	0.03255552	0.76598547
RIPK3	2.27260357	5.71014589	4.81089306	0.00023349	0.03260764	0.75837004
PLXND1	-4.18660184	6.44948687	-4.78720191	0.00024455	0.03393262	0.71472248
TNNT1	-3.56826748	3.05210917	-4.77421429	0.00025084	0.03458343	0.69076581
FSCN1	-6.94214738	6.59076611	-4.74821862	0.00026393	0.03615799	0.64275409
ZNF236-DT	1.59284998	3.26668834	4.73547628	0.0002706	0.03683885	0.61919082
JCAD	-2.2858179	0.81882003	-4.73031429	0.00027335	0.03689488	0.60963974
AIFM3	3.68321833	7.24011199	4.72832415	0.00027442	0.03689488	0.60595662
PXDN	-5.7829441	2.78852944	-4.71527349	0.00028153	0.03761782	0.58179247
TMEM42	1.46421949	6.86511569	4.67330718	0.00030573	0.04035222	0.5039554
GS1-124K5.4	2.20084619	4.42737506	4.66471855	0.00031094	0.04079104	0.48800078
TM7SF3	-1.09952003	9.61668084	-4.65779187	0.0003152	0.0411018	0.47512741
MKKS	1.07182729	9.0378199	4.64448021	0.00032358	0.04148482	0.45037226
MAP4K4	-1.87022311	8.2873717	-4.62986384	0.00033303	0.04148482	0.42316798
GZMM	-2.06544875	1.23942908	-4.62882017	0.00033372	0.04148482	0.42122458
ELF3	1.76600294	11.2059059	4.62731361	0.00033471	0.04148482	0.41841902
PLAAT5	-3.22033217	1.53402257	-4.61228921	0.00034478	0.04198779	0.39042645
TMEM230	1.15918736	8.94262603	4.60340097	0.00035088	0.04243078	0.37385476
CUEDC1	-2.27839198	6.88172981	-4.59794929	0.00035468	0.0426517	0.36368611
NR1I2	4.9785876	6.68181643	4.59415131	0.00035735	0.0427354	0.35660009
ZNF792	-1.69405006	5.96824551	-4.58195094	0.00036606	0.04325261	0.3338269
CD96	-3.11994829	1.02416863	-4.580234	0.00036731	0.04325261	0.33062078
KDSR	1.21364309	7.90490793	4.5797434	0.00036767	0.04325261	0.32970459
PPP1R3D	1.7290905	5.8856795	4.5699012	0.00037489	0.0437546	0.31131919
RASGEF1A	-3.09092452	1.38516867	-4.56457254	0.00037886	0.0438548	0.30136084
LOC10192732	2.16397452	2.16138729	4.55131077	0.00038894	0.0445305	0.27656386
EVA1A	5.51609268	4.13706951	4.54961821	0.00039024	0.0445305	0.27339775
PP7080	2.52484631	7.50288574	4.54432657	0.00039435	0.0445305	0.26349728
MAP3K15	-1.68981942	0.61368671	-4.54258117	0.00039572	0.0445305	0.26023106
BCR	-1.44120814	9.25807198	-4.53859002	0.00039886	0.0445305	0.25276109
OSR1	-4.12552237	3.800022	-4.53500761	0.0004017	0.0445305	0.24605473
IL32	5.33888817	7.40159429	4.53167788	0.00040436	0.0445305	0.23982019
ANKEF1	1.23663083	6.98297141	4.53095326	0.00040494	0.0445305	0.23846328
NLRP11	-2.20805807	0.85866666	-4.53038666	0.00040539	0.0445305	0.23740222
KRTAP5-AS1	3.21090457	2.97479175	4.52978045	0.00040588	0.0445305	0.23626696
GRTP1	1.15393359	8.55145433	4.52796777	0.00040734	0.0445305	0.23287208
VPS16	1.11210794	8.77851702	4.52000256	0.00041383	0.04470061	0.21795044
CGAS	-2.91527082	5.59442265	-4.51702682	0.00041627	0.04470061	0.21237417
MARK1	4.51841599	3.93805934	4.51514268	0.00041783	0.04470061	0.20884298
PMFBP1	2.57971577	5.47226891	4.51348291	0.00041921	0.04470061	0.205732
KBTBD7	3.58828146	5.67694377	4.50816051	0.00042366	0.04480021	0.19575408
ST3GAL1	-4.24203523	2.61283559	-4.50741878	0.00042428	0.04480021	0.19436334
BIVM	1.32975882	7.5239564	4.4952983	0.00043461	0.04507416	0.17162941
CYB5A	1.51495998	10.1988496	4.49461033	0.00043521	0.04507416	0.17033857
TTC32	1.36695082	6.05389601	4.48639081	0.00044237	0.04538161	0.15491252
CDKN1B	1.09045764	8.57389574	4.47033869	0.00045671	0.04641244	0.12476713
SWSAP1	1.45241657	5.50012109	4.46628169	0.0004604	0.04656963	0.11714418
PRDM16	-4.56068759	3.96493007	-4.45483686	0.000471	0.04737476	0.09563102
ITGA10	-2.14628199	4.3992137	-4.43970856	0.0004854	0.04819688	0.06717436
